# Cytochrome P450- and Dehydrogenase-Mediated Regiospecific and Stereoselective Formation of β- and γ‑Lactones in Drimane-Type Sesquiterpenoid Biosynthesis

**DOI:** 10.1021/jacs.6c13983

**Published:** 2026-08-28

**Authors:** Meiting Wu, Jingjing Wu, Daniel J. Janzen, Wen-Bing Yin, Shu-Ming Li

**Affiliations:** † 9377 Philipps-Universität Marburg, Fachbereich Pharmazie, Institut für Pharmazeutische Biologie und Biotechnologie, Robert-Koch-Straße 4, Marburg 35037, Germany; ‡ State Key Laboratory of Microbial Diversity and Innovative Utilization, 85387 Institute of Microbiology, Chinese Academy of Sciences, Beijing 100101, China

## Abstract

Lactone-containing natural products are important candidates for drug discovery. Drimane-type sesquiterpenes (DTSs), characterized by a bicyclic *trans*-decalin scaffold, can bear both β- and γ-lactone moieties. While γ-lactone-containing DTSs have frequently been reported, β-lactone-containing derivatives are rare, and their biosynthesis remains unexplored. Here, we identified a biosynthetic gene cluster (*dri*) in *Aspergillus ustus* and confirmed ustidrimane A (**1**), a β- and γ-lactone-containing DTS, as its product. Heterologous gene expression, precursor feeding, and enzymatic investigation provided evidence for the formation of both lactone rings. In both cases, the reaction cascade is initiated by regiospecific (and stereoselective) methyl hydroxylation, followed by regiospecific and stereoselective oxidation of one hydroxymethyl group to an aldehyde. The resulting hemiacetal was proven to be subsequently oxidized to a lactone. The β-lactone formation is catalyzed by two cytochrome P450 enzymes (DriE and DriF), followed by two oxidation steps catalyzed by two dehydrogenases (DriG and DriH). These findings differ entirely from the known β-lactone formation in fatty acid-, PKS-, and NRPS-derived metabolites. The subsequent γ-lactone formation is catalyzed by a P450 (DriJ) and a dehydrogenase (DriD). DriJ has been shown to be involved in both methyl hydroxylation and hemiacetal formation, while DriD is responsible for the hemiacetal oxidation and also contributes moderately to its formation. Collectively, these findings establish a sequential P450/dehydrogenase-mediated oxidative cascade for the construction of two distinct lactone motifs within a single DTS scaffold. Moreover, they provide the first insight into the β-lactone formation in terpenes, thus unveiling a new strategy for the construction of this structural motif.

## Graphical abstract

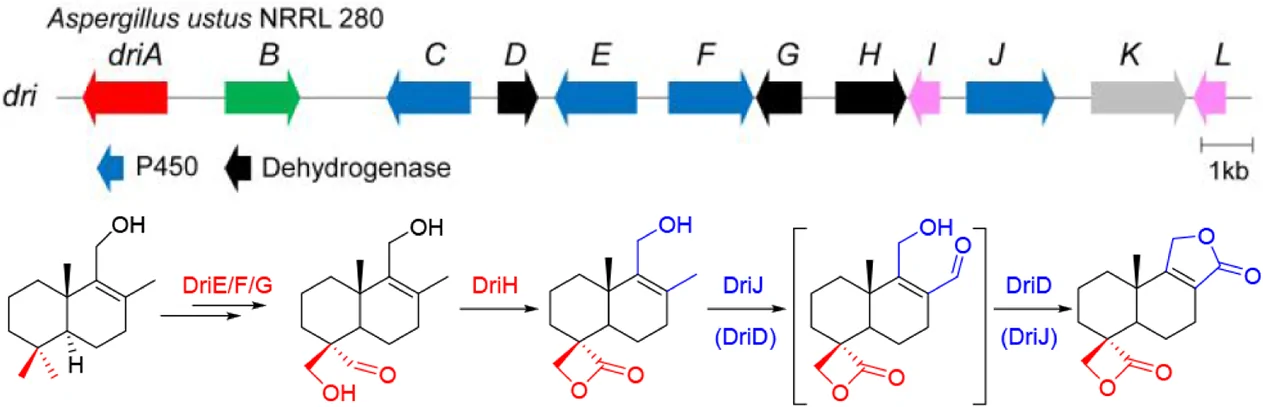

## Introduction

Lactone-containing natural products have long attracted attention in medicinal chemistry.^1^ γ-Lactones are regarded as privileged structures in diverse natural products with various activities, including anticancer, anti-inflammatory, and antibiotic effects.^2,3^ In contrast, β-lactones occur less frequently in nature with a highly strained four-membered ester ring. Several β-lactone-containing natural products exhibit interesting biological activities,^4,5^ such as the lipid and cholesterol metabolism inhibitors lipstatin,^6^ ebelactone A,^7^ hymeglusin,^8^ and vibralactone,^9^ as well as anticancer agents like salinosporamide A^10^ and neuroactive anisatin^11^ (Figure 1A). Orlistat, a lipstatin derivative with a saturated chain, is used clinically as an antiobesity drug.^12^

**Figure 1:**
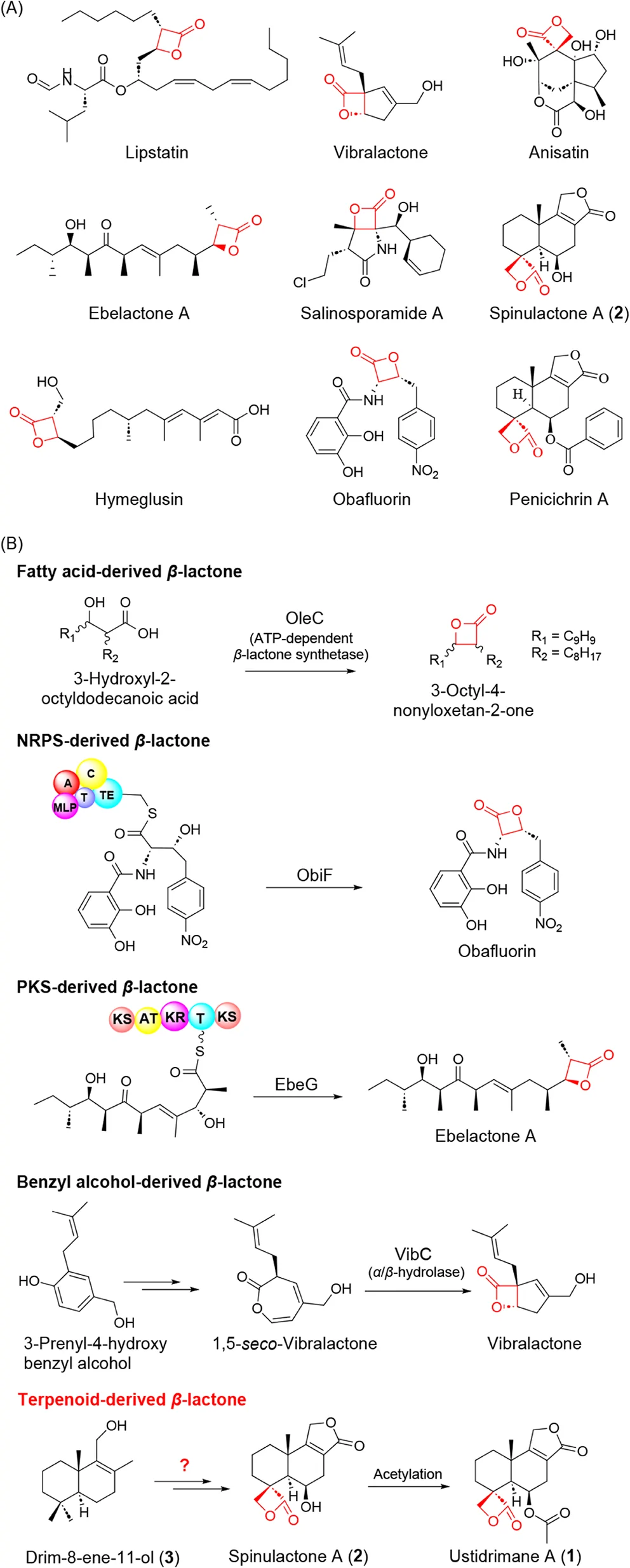
(A) Representative β-lactone-containing natural products. (B) Known biosynthetic strategies for β-lactone formation.

The biosynthesis of β-lactone formation remains less understood and is reported only for nonterpenoid-derived pathways. These include ATP-dependent β-lactone formation by OleC in the fatty acid derivative 3-octyl-4-nonyloxetan-2-one,^13^ and NRPS thioesterase-mediated β-lactonization during obafluorin release (Figure 1B).^14^ The TE-independent β-lactone formation in ebelactone A biosynthesis is contributed by the PKS EbeG,^15^ and the *α/β*-hydrolase VibC catalyzes cyclization, thereby generating the fused bicyclic β-lactone scaffold in vibralactone (Figure 1B).^16^ Hemiacetal oxidation has been reported for the formation of lactones, e.g., in the five-membered γ-lactones in the biosynthesis of momilactone A^17^ and meroaspochalasin.^18^ However, to the best of our knowledge, β-lactone formation in terpenoids has not been reported prior to this study.

Drimane-type sesquiterpenes (DTSs) are characterized by a *trans*-decalin core and are distributed across various organisms, including plants, fungi, bacteria, and marine organisms.^19–23^ Several members of this family, such as spinulactone A (**2**)^24^ and penicichrin A^25^ (Figure 1A), contain both β- and γ-lactones. Considerable progress has been made in elucidating DTS biosynthetic pathways.^19,26–30^ However, genes and enzymes responsible for the biosynthesis of the dual-lactone drimane structures have not been reported, which raised our interest in investigating the biosynthesis of these metabolites.

*Aspergillus ustus* has been repeatedly reported as a notable fungal source of structurally diverse DTSs.^31–34^ Genome mining based on the known terpene cyclase AstC from the *ast* cluster associated with 14-deacetyl astellolide A,^28^ led to the identification of a candidate biosynthetic gene cluster (*dri*) in *Aspergillus ustus* NRRL 280, showing a significant sequence identity between the two clusters (Figure S1). In the Ast pathway, the formation of the γ-lactone ring in 14-deacetyl astellolide A was proposed to be derived from the scaffold drim-8-ene-11-ol with the involvement of a P450 enzyme and a dehydrogenase (**3**, Figure 1B).^28^ However, the detailed enzymatic mechanism of the γ-lactone formation has not been fully elucidated. Cultivation of the strain NRRL 280 led to the detection of compound **1**, with a typical UV absorption characteristic of DTS-type metabolites and a [M + H]^+^ ion at *m*/*z* 321.1336 [M + H]^+^ (Figures S2–S4). **1** is likely an acetylated analog of spinulactone A from *Penicillium spinulosum*.^24^ Herein, we report the identification of the *dri* cluster and the catalytic mechanisms underlying the formation of both β- and γ-lactone rings from the putative precursor **3** (Figure 1B).

## Results and Discussion

### Identification of *Dri* Cluster for Ustidrimane A Biosynthesis

The identified *dri* cluster of *A. ustus* NRRL 280 contains 12 open reading frames, designated *driA–driL* (GenBank accession number: PZ445384). They code for one terpene cyclase (TC, DriA), two phosphatases (DriI and DriL), four cytochrome P450 enzymes (P450s, DriC, DriE, DriF, and DriJ), three dehydrogenases (DriD, DriG, and DriH), one acetyltransferase (DriB), and one transporter (DriK). Among them, nine share 42% or higher amino acid sequence identities with those from the Ast pathway.^28^ Interestingly, the *dri* cluster contains three dehydrogenase genes, while the *ast* cluster includes only one (Figure S1). The two additional dehydrogenases, DriG and DriH, could be involved in the β-lactone formation.

Large scale fermentation of NRRL 280 in rice medium resulted in the isolation of compound **1**. Structural elucidation (see Supporting Information for details) led to the identification of ustidrimane A, an acetylated derivative of spinulactone A (**2**)^24^ and an analog of 14-deacetyl astellolide A^28^ (Figures 1 and S1). Heterologous expression of the complete *dri* cluster in *A. nidulans* LO8030 generated the recombinant strain MW11, which produced **1** after cultivation in liquid minimal medium (LMM) for 7 days (Figure 2Bii), confirming their biosynthetic relationship. In addition, a minor peak with a [M + H]^+^ ion at *m*/*z* 279.1226 was also detected (Figure 2Bii), indicating the presence of spinulactone A (**2**), the deacetylated derivative of **1**.^24^

**Figure 2:**
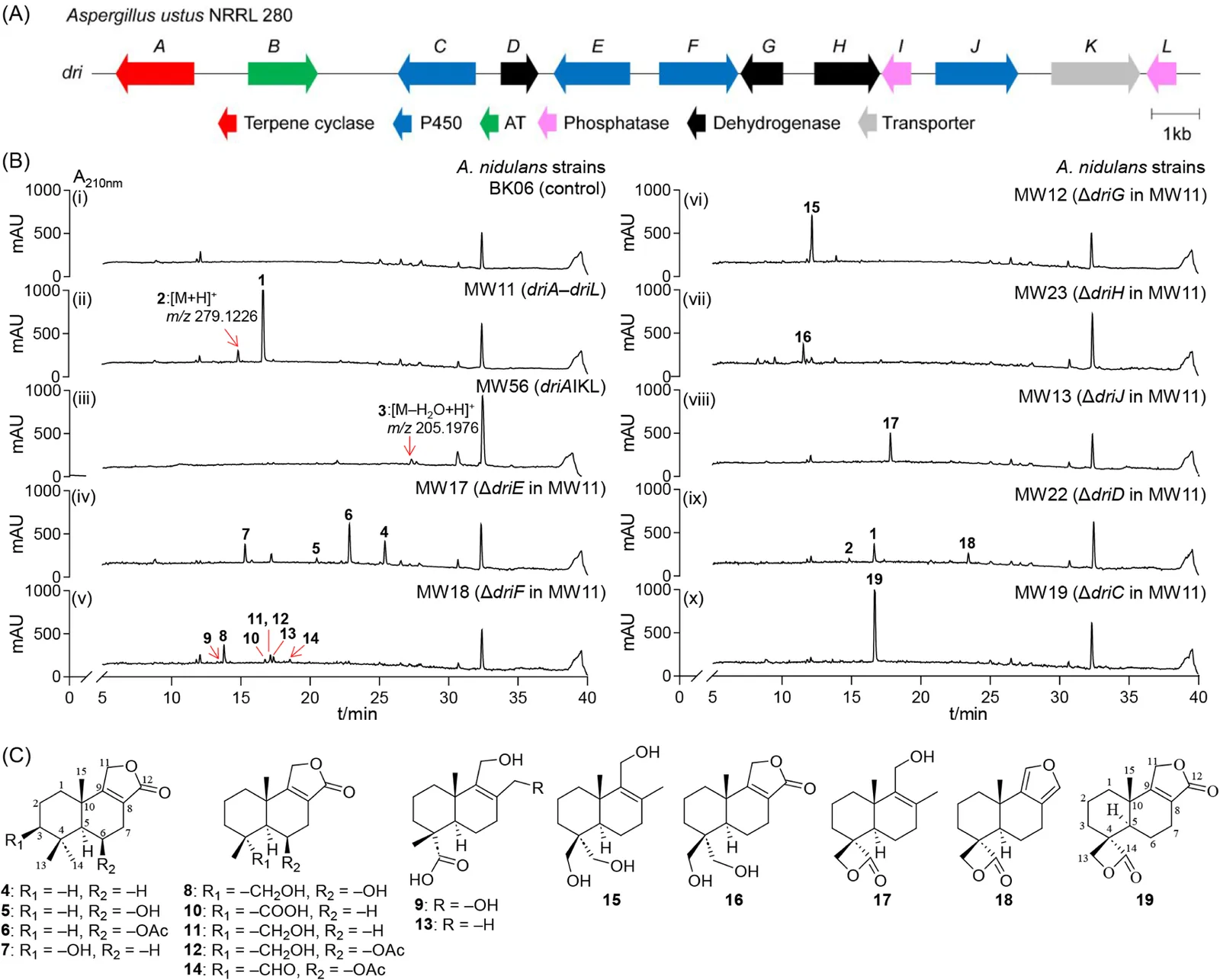
(A) The identified *dri* cluster of *A. ustus* NRRL 280. (B) LC-MS chromatograms of the metabolites from *A. nidulans* transformants. Absorptions at UV 210 nm are displayed. (C) Structures of the accumulated metabolites.

### DriA, DriI, and DriL Are Responsible for the Formation of the Scaffold Drim-8-ene-11-ol (3)

Because the TC AstC and the two phosphatases AstI and AstK were proposed to be involved in the formation of the scaffold drim-8-ene-11-ol (**3**),^28^ their homologous counterparts in the *dri* cluster, *driA*, *driI*, and *driL*, were therefore coexpressed with the transporter gene *driK* in *A. nidulans* LO8030. Cultivation of the resulting recombinant strain MW56 in LMM for 7 days led to the production of **3** (Figures 1B and 2Biii), supporting their conserved role in the scaffold formation.

### Single-Gene Deletions Prove Involvement of DriE, DriF, DriG, and DriH in the β-Lactone Ring Formation

Downstream modifications of **3**, including hydroxylation, oxidation, and lactone ring formation, can be expected by the three dehydrogenases and four P450 enzymes encoded by the *dri* cluster. Therefore, we deleted *driC*–*driH* and *driJ* individually in *A. nidulans* MW11. Each individual deletion of *driE–driH* resulted in the abolishment of the production of **1** and the accumulation of metabolites lacking the β-lactone (Figure 2Biv–Bvii, See SI for UV, MS, and NMR data and spectra as well as for structural elucidation), suggesting that all the four genes are involved in its formation. An array of metabolites bearing γ-lactone rings was detected among the accumulated intermediates in the Δ*driE* and Δ*driF* mutants.

The deletion of the P450 gene *driE* in *A. nidulans* MW11 led to the detection of **4**–**7**, carrying two unmodified methyl groups at C-4 (Figure 2Biv and C), suggesting that DriE likely catalyzes the initial methyl hydroxylation, which triggers the formation of the β-lactone. It is also plausible that **4** undergoes DriE-mediated downstream modifications to yield **5**–**7** via another pathway or by other host enzymes.

Disrupting another P450 gene, *driF*, in *A. nidulans* MW11 resulted in the accumulation of **8**–**14** (Figure 2Bv). The NOESY spectra of **8**–**10** and **12**–**14** proved their configuration at C-4, *i.e* with a nonmodified C-13 *β*-methyl group and C-14 residues with different oxidation stages and α-orientations (Figure 2C). Along with the metabolites found in the Δ*driE* mutant, these results proved that DriF functions as the C-13 hydroxylase following hydroxylation at C-14 by DriE. The presence of differently oxidized C-14 substituents suggests that the hydroxylated DriE product was further oxidized by other enzymes in the absence of DriF.

Cultivation of the Δ*driG* and Δ*driH* mutants resulted in the accumulation of **15** and **16**, respectively (Figure 2Bvi and Bvii). Both compounds are hydroxylated at C-13 and C-14 (Figure 2C). This indicates that the two dehydrogenases may catalyze downstream oxidations of the dihydroxymethyl moiety to form the β-lactone ring.

### Deletion of *DriD* and *DriJ* Abolishes the γ-Lactone Ring Formation

The deletion of the P450 gene *driJ* resulted in the sole formation of **17** (Figure 2Bviii). Compound **17** differs from **3** only by the β-lactone ring, suggesting that *driJ* catalyzes initial methyl hydroxylation at C-12, which triggers subsequent γ-lactone formation.

Disrupting the dehydrogenase gene *driD* led to the accumulation of **18**, as well as **1** and **2**, but with significantly lower yields than in MW11 (Figure 2Bii and Bix). Notably, **18** contains a β-lactone ring and a furan ring between C-8 and C-9, rather than a γ-lactone ring. This structure is distinct from those of other DTS products in the pathway. This suggests that DriD is involved in the γ-lactone ring formation, and that its substrate is converted via a shunt pathway to form the furan motif in its absence. The production of **1** and **2** in the Δ*driD* mutant implies that the function of DriD can be partially complemented by another enzyme in the pathway or by a host enzyme, although with lower efficiency.

### *β*-Lactone Precedes γ-Lactone Ring Formation

Deleting *driE* and *driF* resulted in the production of much more metabolites with a γ-lactone than those with a β-lactone ring in the Δ*driD* and Δ*driJ* mutants (Figure 2B and C). This indicates that either the γ-lactone-forming enzymes have broad substrate flexibility, or the γ-lactone ring is formed prior to the β-lactone ring.

To prove the reaction order, we fed **4**, a less modified metabolite in the Δ*driE* mutant, to *A. nidulans* MW74, harboring the β-lactone forming genes *driE–driH*. Subsequent LC-MS analysis revealed no detectable conversion (Figure 3A), thus excluding it as a precursor for the β-lactone. In contrast, the putative precursor for the first step of the γ-lactone formation (**17**) was fed to the MW50 strain harboring *driJ*, which led to accumulation of **18**, **19**, and **23** (Figure 3B). In **19**, the γ-lactone ring is already installed. Compound **23** with a dihydroxymethyl feature is likely the direct product of **17** with DriJ, which is then converted to **18** and **19** by the same or other host enzymes. These results demonstrate that β-lactone formation precedes γ-lactone formation in the pathway.

**Figure 3:**
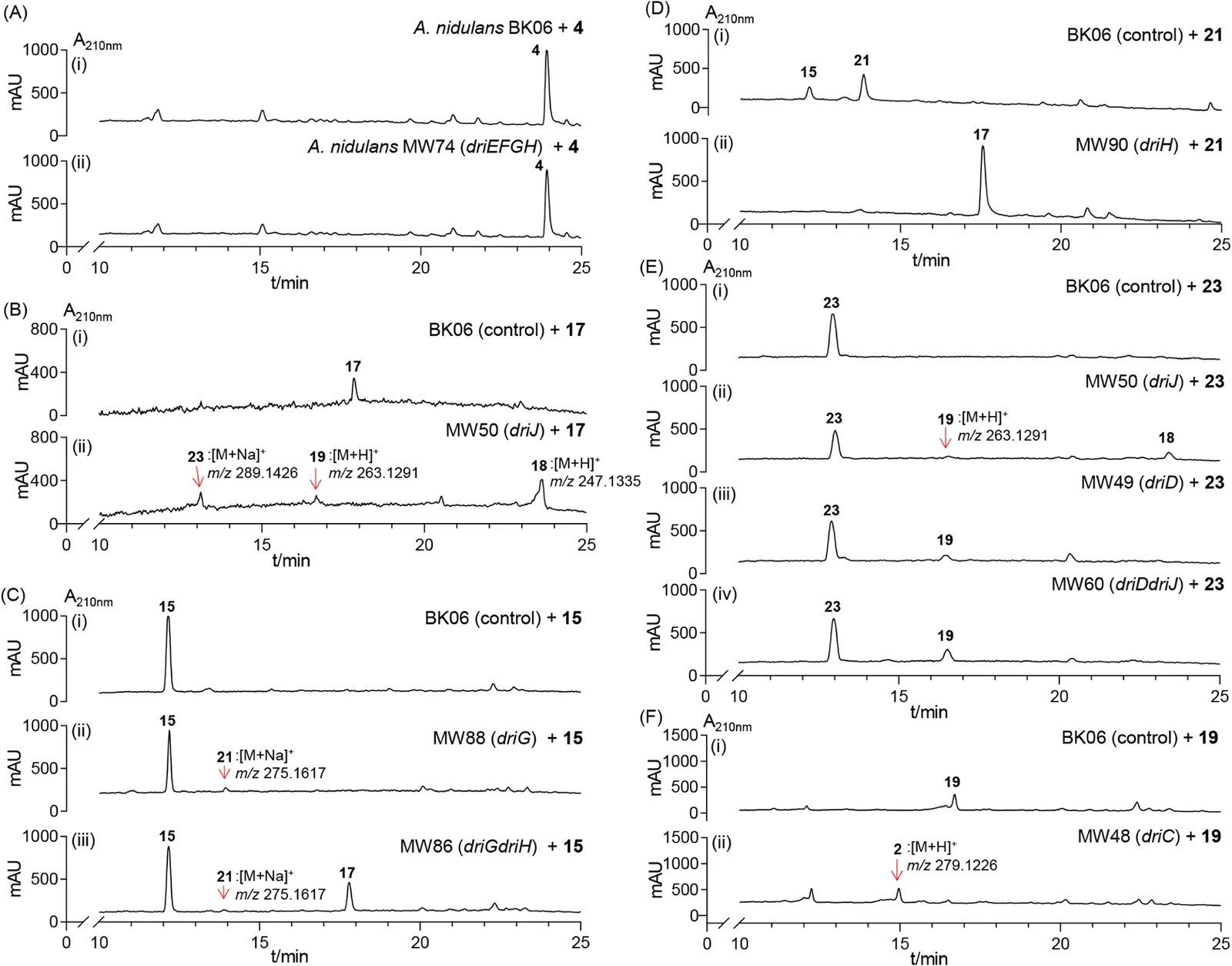
LC-MS analysis of the *A. nidulans* transformants cultures after feeding with compounds **4** (A), **17** (B), **15** (C), **21** (D), **23** (E), and **19** (F). Absorptions at UV 210 nm are displayed. See Scheme 1 for the corresponding reactions and structures.

### Double Mutant Analysis Reveals Reaction Order during β-Lactone Ring Formation

To identify the genuine intermediates involved in β-lactone formation and to establish the biosynthetic sequence, we individually deleted *driE*–*driH* in the previously generated Δ*driJ* strain, in which the γ-lactone formation was blocked. In the Δ*driJ*Δ*driE* mutant, the formation of compound **17** was abolished and **3**—the product of the TC DriA and the two phosphatases DriI and DriL—accumulated as the major product (Figure 4Aii). This indicates that compound **3** is the natural substrate of DriE. The conclusion is also supported by the detection of **20** after heterologous coexpression of *driA*, *driI*, *driL*, *driK*, and *driE* in *A. nidulans* LO8030 (Figure S126). Compound **20** bears an additional hydroxy group at C-14 compared to **3**, indicated by the correlation of H-13 and H-15 in its NOESY spectrum and in those of related compounds **8**–**10** and **12**–**14**. The result clearly demonstrates that DriE is responsible for C-14 hydroxylation.

**Figure 4:**
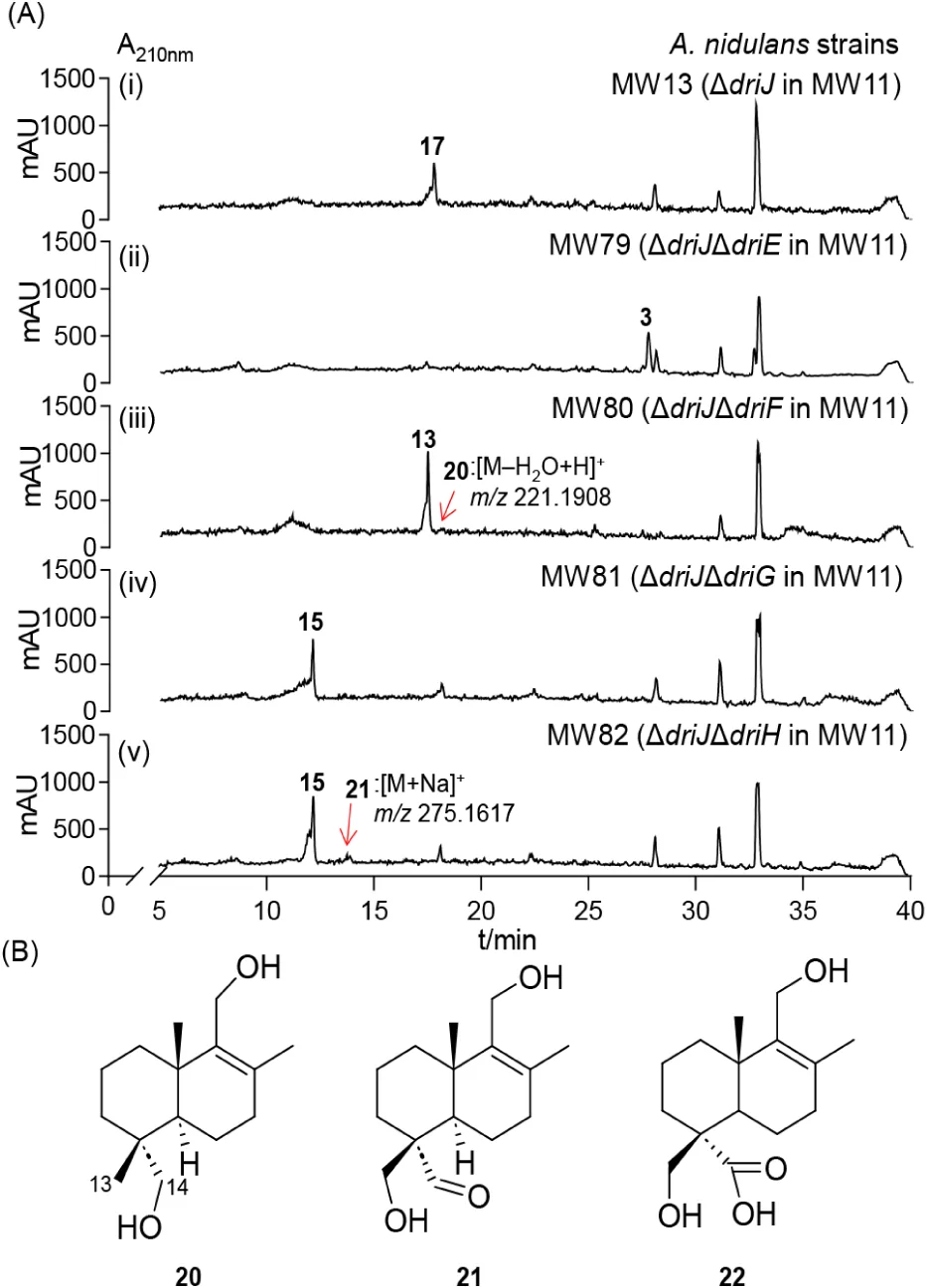
LC-MS chromatograms of the metabolites from *A. nidulans* transformants. Absorptions at UV 210 nm are displayed.

Compound **13** was detected as the predominant peak in the chromatogram after the cultivation and LC-MS analysis of the Δ*driJ*Δ*driF* mutant (Figure 4Aiii). Ion detection also indicated the presence of **20**, though with very low intensity. Both compounds lack a modification at C-13, indicating the function of the P450 DriF in the hydroxylation of this methyl group. Compound **20** can be considered as the substrate of DriF, which is oxidized to **15**. Heterologous coexpression of *driA*, *driI*, *driL*, *driK*, *driE*, and *driF* in *A. nidulans* LO8030 yielded **15** (Figure S126), which has an additional hydroxy group at C-13 compared to **20**. This proves that DriF is responsible for the conversion of **20** to **15** by C-13 hydroxylation.

In the Δ*driJ*Δ*driG* mutant, **15** was accumulated as the major product, whereas the Δ*driJ*Δ*driH* mutant produced mainly **15**, along with a small amount of **21** (Figure 4Aiv and Av). These results suggest that **15** is the substrate of DriG, which likely oxidizes the C-14 hydroxyl group to form the aldehyde intermediate **21**. However, **21** can be partially reduced by an endogenous enzyme back to **15**, as demonstrated by feeding it to the control strain BK06 (Figure 3Di), which accounted for the predominant accumulation of **15** in the Δ*driJ*Δ*driH* mutant (Figure 4Av).

### *In Vitro* Assays with Recombinant DriG Confirmed Its Role as NAD^+^-dependent Enzyme in the Aldehyde Formation

To investigate its function biochemically, recombinant His_6_-tagged DriG was overproduced in *E. coli* BL21, purified on Ni-NTA, and subjected to *in vitro* enzymatic assays (Figure S127). Incubation of compound **15** with the purified DriG in the presence of NAD^+^ led to the time-dependent formation of the aldehyde **21**, whereas no conversion was detected in the heat-inactivated enzyme control. Prolonged incubation led to a decrease in the amount of **21,** and an accumulation of the carboxylic acid product **22**. This suggests that **21** can be further oxidized by DriG under the reaction conditions (Figures 4B, 5Ai–Aiv). Substituting NAD^+^ with NADP^+^ did not result in the conversion of **15**, even after an incubation period of 1 h (Figure 5Av and vi). The DriG reaction followed Michaelis–Menten kinetics. An apparent *K*_M_ value of 0.16 ± 0.01 mM and turnover number of 3.04 ± 0.05 s^–1^ were determined for the conversion of **15** (Figure S128).

**Figure 5:**
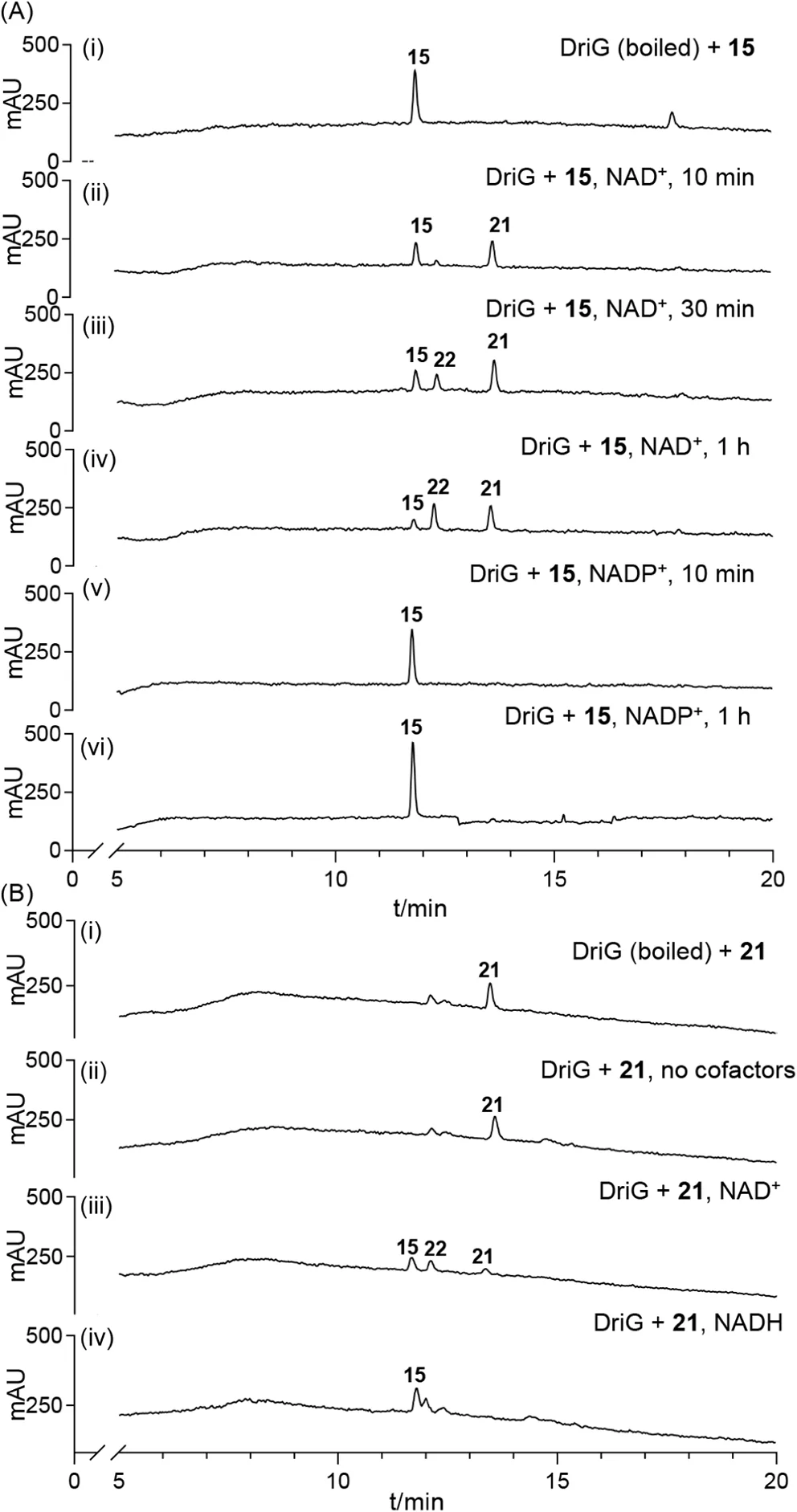
LC-MS chromatograms of DriG *in vitro* assays with **15** (A) and **21** (B). Absorptions at UV 210 nm are displayed.

Notably, the formation of the β-lactone product **17** was not observed in the DriG *in vitro* reaction, suggesting that DriG catalyzes alcohol-to-aldehyde oxidation, but is insufficient for the β-lactone formation. Additional enzyme assays using **21** as the substrate showed that **22** was generated in the presence of NAD^+^. Therefore, **22** was identified as an overoxidation shunt product derived from the aldehyde **21** (Figure 5Biii). Replacing the cofactor NAD^+^ by NADH resulted in reduction of **21** to **15** (Figure 5Biv). These results demonstrate that DriG function as a cofactor-dependent redox enzyme.

### DriG and DriH Sequentially Convert Ustidrimane I (15) to the β-Lactone Derivative Ustidrimane K (17)

To further investigate the roles of the two dehydrogenases DriG and DriH in the β-lactone formation, additional feeding experiments were conducted. As shown in Figure 3C, compared to the absence of detectable conversion in the control strain *A. nidulans* BK06, the feeding of **15** to *A. nidulans* MW88 harboring *driG* resulted in the modest formation of compound **21**. This can be attributed to its possible reduction back to compound **15** by endogenous enzymes in *A. nidulans* or by DriG itself (Figures 3Di and 5Biii). Moreover, feeding of **15** to *A. nidulans* MW86 harboring both *driG* and *driH*, resulted in the formation of the β-lactone product **17**, accompanied by a minor amount of **21**. These results support a sequential pathway in which DriG first oxidizes **15** to the aldehyde intermediate **21**, which is subsequently processed by DriH toward β-lactone formation. In addition, feeding of **21** to the *driH* expression strain led to complete conversion of **21** to the β-lactone product **17**, thereby confirming the β-lactone formation catalyzed by DriH originating from the aldehyde substrate **21** (Figure 3Dii).

### DriD and DriJ Are Together Responsible for the Formation of the γ-Lactone Ring

Further feeding experiments were conducted to verify the functions of DriJ and DriD. As shown in Figure 3Bii, **17** was completely transformed in the *driJ* expression strain to the hydroxylated product **23**. Intriguingly, **23** also underwent further oxidation to form **18**, which carries a furan ring, as well as the γ-lactone product **19**. These results indicate that DriJ can not only convert **17** to **23**, but also further oxidize it to generate **18** and **19**. To exclude the possibility of endogenous enzymes for the conversion, **23** was fed to the control strain BK06 and the *driJ* expression strain. As shown in Figure 3Ei and Eii, **23** was not converted by any host enzymes, rather, it was transformed by DriJ into **18** and **19**. In addition, strains expressing *driD* alone or coexpressing *driJdriD* converted **23** exclusively to **19**, with the higher level of **19** observed in the *driJdriD* coexpressing strain (Figure 3Eiii and Eiv). It is therefore proposed that DriD can also oxidize the C-12 hydroxy group in **23** to an aldehyde group and further convert it to form the γ-lactone product **19**, with higher efficiency for the second step. High γ-lactone formation was observed in the presence of both DriD and DriJ.

### DriC Is Responsible for C-6 Hydroxylation

As shown in Figure 2Bx, deletion of the P450 gene *driC* abolished the production of **1** and **2** and led to the accumulation of **19**, containing both β- and γ-lactone rings but lacks the C-6 substituent. In addition, feeding of **19** to the *A. nidulans* MW48 harboring *driC* led to its complete conversion to **2**, confirming the function of DriC in hydroxylating C-6 (Figure 3F).

### Proposed Pathway of Ustidrimane A in *A. ustus* NRRL 280

Based on the genetic engineering, feeding, and *in vitro* enzymatic studies described above, an integrated biosynthetic pathway for the formation of both β- and γ-lactone rings in **1** is proposed (Scheme 1). The core scaffold **3** is assembled through the sequential reactions catalyzed by the terpene cyclase DriA and the two phosphatases DriI and DriL. Initiating from **3**, the β-lactone moiety is generated sequentially by the two P450 monooxygenases DriE and DriF and the two dehydrogenases DriG and DriH. Initially, DriE and DriF hydroxylate the C-14 and C-13 methyl groups of **3**, respectively, thereby generating the C-13/C-14 dihydroxylated intermediate **15**. DriG then oxidizes stereoselectively the C-14 hydroxyl group in **15** to an aldehyde **21**, which likely undergoes intramolecular hemiacetalization to form the cyclic hemiacetal intermediate. Subsequent DriH-catalyzed oxidation results in the conversion of **21** to the β-lactone product **17** (Scheme 1).

**Scheme 1:**
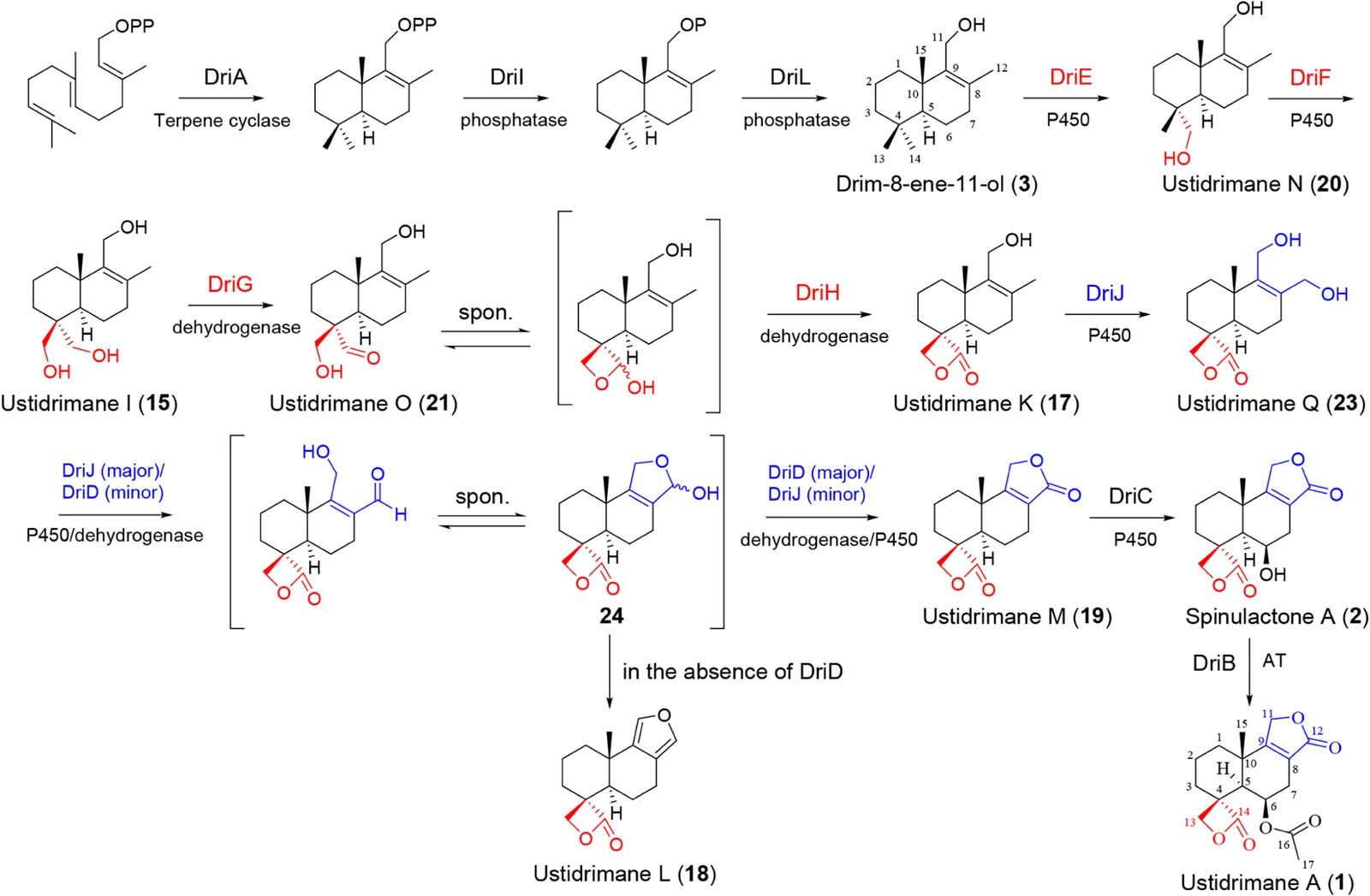
Proposed Biosynthetic Pathway of Ustidrimane A (**1**)

The formation of the γ-lactone **19** from **17** is a result of a combined action of the P450 DriJ and the dehydrogenase DriD. DriJ catalyzes the hydroxylation at the C-12 methyl group in **17**, yielding the C-11/C-12 dihydroxylated product **23**. This product is subsequently oxidized at C-12, predominantly by DriJ and to a lesser extent by the dehydrogenase DriD, resulting in the formation of the hemiacetal intermediate **24**. The hydroxyl group of this intermediate is then primarily oxidized by the dehydrogenase DriD, with minor contributions from P450 DriJ or host enzymes, to generate the γ-lactone product **19**. In the absence of DriD, **24** undergoes dehydration to afford **18.** Finally, the P450 monooxygenase DriC hydroxylates C-6 of **19** to form intermediate **2**, which is subsequently acetylated by the acetyltransferase DriB to yield the final product **1** (Scheme 1).

## Conclusions

In this study, we elucidated the *dri* biosynthetic gene cluster responsible for ustidrimane A formation in *Aspergillus ustus* NRRL 280 through heterologous expression, precursor feeding, and *in vitro* enzymatic characterization. We demonstrated the coordinated action of cytochrome P450 enzymes and dehydrogenases in the regiospecific (and stereoselective) and successive construction of β- and γ-lactone rings on the drimane-type sesquiterpene scaffold via oxidation of one of the two hydroxymethyl groups and the formed hemiacetals. The γ-lactone is assembled by one P450 (DriJ) and one dehydrogenase (DriD), via a hemiacetal as reported previously.^17,18^ Interestingly, a sequential oxidative cascade with two P450s (DriE and DriF) and two dehydrogenases (DriG and DriH) for the β-lactone formation differs completely from known mechanisms of β-lactone-forming enzymes. The reported mechanisms include ATP-dependent activation of β-hydroxy acids, NRPS- or PKS-associated lactonization, and *α/β*-hydrolase-catalyzed cyclization. In comparison, the Dri pathway uses two P450 enzymes for selective hydroxylation of two methyl groups. The two dehydrogenases catalyze successively stereoselective alcohol oxidation and conversion of the hemiacetal to the β-lactone ring. To the best of our knowledge, this is the first example for the β-lactone formation in a terpenoid biosynthetic pathway. Therefore, this work provides new biosynthetic insights into the β-lactone formation in terpenes and unveils a new strategy for constructing this strained ring system.

## Supplementary Material



## Notes

Materials; experimental procedures; physicochemical properties; spectroscopic data (PDF)

## References

[1] Sartori S. K., Diaz M. A. N., Diaz-Muñoz G. (2021). Lactones: Classification, synthesis,
biological activities, and industrial applications. Tetrahedron.

[2] Hur J., Jang J., Sim J. (2021). A review of the pharmacological activities
and recent synthetic advances of γ-butyrolactones. Int. J. Mol. Sci..

[3] Nozaki Y., Katayama N., Harada S., Ono H., Okazaki H. (1989). Lactivicin,
a naturally occurring non-beta-lactam antibiotic having beta-lactam-like
action: Biological activities and mode of action. J. Antibiot..

[4] Scott T. A., Heine D., Qin Z., Wilkinson B. (2017). An L-threonine
transaldolase is required for L-threo-β-hydroxy-α-amino
acid assembly during obafluorin biosynthesis. Nat. Commun..

[5] Robinson S. L., Christenson J. K., Wackett L. P. (2019). Biosynthesis and chemical diversity
of β-lactone natural products. Nat. Prod.
Rep..

[6] Weibel E. K., Hadvary P., Hochuli E., Kupfer E., Lengsfeld H. (1987). Lipstatin,
an inhibitor of pancreatic lipase, produced by *Streptomyces
toxytricini*. I. Producing organism, fermentation, isolation
and biological activity. J. Antibiot..

[7] Umezawa H., Aoyagi T., Uotani K., Hamada M., Takeuchi T., Takahashi S. (1980). Ebelactone,
an inhibitor of esterase, produced by actinomycetes. J. Antibiot..

[8] Greenspan M. D., Yudkovitz J. B., Lo C. Y., Chen J. S., Alberts A. W., Hunt V. M., Chang M. N., Yang S. S., Thompson K. L., Chiang Y. C. (1987). Inhibition of hydroxymethylglutaryl-coenzyme
A synthase
by L-659,699. Proc. Natl. Acad. Sci. U. S. A..

[9] Liu D.-Z., Wang F., Liao T.-G., Tang J.-G., Steglich W., Zhu H.-J., Liu J.-K. (2006). Vibralactone:
A lipase inhibitor
with an unusual fused beta-lactone produced by cultures of the basidiomycete *Boreostereum vibrans*. Org. Lett..

[10] Feling R. H., Buchanan G. O., Mincer T. J., Kauffman C. A., Jensen P. R., Fenical W. (2003). Salinosporamide A:
a highly cytotoxic proteasome inhibitor
from a novel microbial source, a marine bacterium of the new genus *Salinospora*. Angew. Chem., Int. Ed..

[11] Kudo Y., Oka J. I., Yamada K. (1981). Anisatin,
a potent GABA antagonist,
isolated from *Illicium anisatum*. Neurosci. Lett..

[12] Borgström B. (1988). Mode of action
of tetrahydrolipstatin: A derivative of the naturally occurring lipase
inhibitor lipstatin. Biochim. Biophys. Acta.

[13] Christenson J. K., Richman J. E., Jensen M. R., Neufeld J. Y., Wilmot C. M., Wackett L. P. (2017). β-Lactone
synthetase found in the olefin biosynthesis
pathway. Biochemistry.

[14] Kreitler D. F., Gemmell E. M., Schaffer J. E., Wencewicz T. A., Gulick A. M. (2019). The structural basis of N-acyl-α-amino-β-lactone
formation catalyzed by a nonribosomal peptide synthetase. Nat. Commun..

[15] Wyatt M. A., Ahilan Y., Argyropoulos P., Boddy C. N., Magarvey N. A., Harrison P. H. M. (2013). Biosynthesis
of ebelactone A: Isotopic tracer, advanced
precursor and genetic studies reveal a thioesterase-independent cyclization
to give a polyketide β-lactone. J. Antibiot..

[16] Feng K.-N., Yang Y.-L., Xu Y.-X., Zhang Y., Feng T., Huang S.-X., Liu J.-K., Zeng Y. (2020). A hydrolase-catalyzed
cyclization forms the fused bicyclic *β*-lactone
in vibralactone. Angew. Chem., Int. Ed..

[17] Kitaoka N., Zhang J., Oyagbenro R. K., Brown B., Wu Y., Yang B., Li Z., Peters R. J. (2021). Interdependent evolution
of biosynthetic gene clusters for momilactone production in rice. Plant Cell.

[18] Li P., Meng J., Zhang X., Zhang X., Ye Y., Zhao Y., Huang X., Zha Z., Guan Z., Lai S., Chen Z., Luo Z., Wang J., Chen C., Liu J., Gu L., Sun Y., Li S., Zhu H., Ye Y., Zhou Y., Zhang Y. (2025). Cooperative redox reactions encoded
by two gene clusters enable intermolecular cycloaddition cascade for
the formation of meroaspochalasins. Angew. Chem.,
Int. Ed..

[19] Zhang D., Du W., Pan X., Lin X., Li F.-R., Wang Q., Yang Q., Xu H.-M., Dong L.-B. (2024). Discovery and biosynthesis
of bacterial drimane-type sesquiterpenoids from *Streptomyces
clavuligerus*. Beilstein J. Org. Chem..

[20] Du W., Yang Q., Xu H., Dong L. (2022). Drimane-type sesquiterpenoids
from fungi. Chin. J. Nat. Med..

[21] Dai Q., Zhang F.-L., Feng T. (2021). Sesquiterpenoids
specially produced
by fungi: Structures, biological activities, chemical and biosynthesis
(2015–2020). J. Fungi..

[22] Chen T.-H., Lin H.-C. (2023). Terpene synthases
in the biosynthesis of drimane-type
sesquiterpenes across diverse organisms. ChemBiochem.

[23] Peng G.-K., Su F., Liu J.-K., Chen H.-P., Li X. (2026). Drimane sesquiterpenes
isolated from the fruiting body of the clavarioid fungus *Ramaria
botrytoides*. Molecules.

[24] Zhang X.-Q., Liu J., Xu W.-L., Men W.-H., He H.-B., Guo Z.-Y., Proksch P. (2025). Spinulactones A-F:
Bioactive drimane-type sesquiterpenes
from the endophytic *Penicillium spinulosum* 9–1. J. Nat. Prod..

[25] Li S.-Y., Yang X.-Q., Chen J.-X., Wu Y.-M., Yang Y.-B., Ding Z.-T. (2022). The induced cryptic metabolites and
antifungal activities
from culture of *Penicillium chrysogenum* by supplementing
with host *Ziziphus jujuba* extract. Phytochemistry.

[26] Henquet M. G. L., Prota N., van der Hooft J. J. J., Varbanova-Herde M., Hulzink R. J. M., de Vos M., Prins M., de Both M. T. J., Franssen M. C. R., Bouwmeester H. (2017). Identification of a drimenol synthase and drimenol oxidase from *Persicaria hydropiper*, involved in the biosynthesis of insect
deterrent drimanes. Plant J..

[27] Kwon M., Cochrane S. A., Vederas J. C., Ro D.-K. (2014). Molecular cloning
and characterization of drimenol synthase from valerian plant (*Valeriana officinalis*). FEBS Lett..

[28] Shinohara Y., Takahashi S., Osada H., Koyama Y. (2016). Identification of a
novel sesquiterpene biosynthetic machinery involved in astellolide
biosynthesis. Sci. Rep..

[29] Huang Y., Hoefgen S., Valiante V. (2021). Biosynthesis of fungal drimane-type
sesquiterpene esters. Angew. Chem., Int. Ed..

[30] Chen T.-H., Chen C.-T., Lee C.-F., Huang R.-J., Chen K.-L., Lu Y.-C., Liang S.-Y., Pham M.-T., Rao Y. K., Wu S.-H., Chein R.-J., Lin H.-C. (2023). The biosynthetic
gene cluster of mushroom-derived antrocin encodes two dual-functional
haloacid dehalogenase-like terpene cyclases. Angew. Chem., Int. Ed..

[31] Gui P., Fan J., Zhu T., Fu P., Hong K., Zhu W. (2022). Sesquiterpenoids
from the mangrove-derived *Aspergillus ustus* 094102. Mar. Drugs.

[32] Neuhaus G. F., Loesgen S. (2021). Antibacterial drimane
sesquiterpenes from *Aspergillus
ustus*. J. Nat. Prod..

[33] Zhou H., Zhu T., Cai S., Gu Q., Li D. (2011). Drimane sesquiterpenoids
from the mangrove-derived fungus *Aspergillus ustus*. Chem. Pharm. Bull..

[34] Liu H., Edrada-Ebel R., Ebel R., Wang Y., Schulz B., Draeger S., Müller W. E. G., Wray V., Lin W., Proksch P. (2009). Drimane sesquiterpenoids
from the fungus *Aspergillus
ustus* isolated from the marine sponge *Suberites domuncula*. J. Nat. Prod..

