# Supplementary material for: Cytochrome P450- and Dehydrogenase-Mediated Regiospecific and Stereoselective Formation of β- and γ‑Lactones in Drimane-Type Sesquiterpenoid Biosynthesis
Source: J Am Chem Soc. 2026 Aug 28;148(35):38483–90. doi: 10.1021/jacs.6c13983 (PMC13564434; doi:10.1021/jacs.6c13983)
Supplement: Supplementary file 1 [file ja6c13983_si_001.pdf]

Supporting Information

Cytochrome P450- and Dehydrogenase-Mediated Regiospecific and Stereoselective Formation of *β*- and γ-Lactones in Drimane-Type Sesquiterpenoid Biosynthesis

Meiting Wu,[a, ‡] Jingjing Wu,[a, ‡] Daniel J. Janzen,[a] Wen-Bing Yin,[b] and Shu-Ming Li[a, *]

[a]Philipps-Universität Marburg, Fachbereich Pharmazie, Institut für Pharmazeutische Biologie und Biotechnologie, Robert-Koch-Straße 4, Marburg 35037, Germany.

[b]State Key Laboratory of Microbial Diversity and Innovative Utilization, Institute of Microbiology, Chinese Academy of Sciences, Beijing 100101, China.

‡ These authors contributed equally
*Corresponding author. Email: shuming.li@staff.uni-marburg.de

Table of Contents

Experiment Procedures 9

1. Media, strains, and growth conditions 9
2. Genomic DNA extraction 9
3. RNA isolation and cDNA synthesis...9
4. PCR amplification, cloning, and plasmid construction 9
5. Genetic manipulation in *A. nidulans*..10
6. Overproduction and purification of DriG 11
7. *In vitro* assays of DriG 11
8. LC-MS analysis 11
9. NMR analysis...12
10. Large-scale fermentation, extraction, and isolation of secondary metabolites 12
11. Structural elucidation 15
12. Measurement of optical rotation 19
13. Experimental ECD 19
14. Feeding experiments 19
15. Physicochemical properties of the compounds described in this study 19

Supplementary Tables 22

Table S1. Sequence identities of proteins encoded by *dri* cluster (GenBank accession number: PZ445384) from *A. ustus* NRRL 280 with those of known clusters. 22

Table S2. Strains used in this study. 23

Table S3. Plasmids used and constructed in this study...24

Table S4. Primers designed in this study. 27

Table S5. NMR data of ustidrimane A (**1**) in $CDCl_3$. 35

Table S6. NMR data of drim-8-ene-11-ol (**3**) in $CDCl_3$. 36

Table S7. NMR data of confertifolin (**4**) in $CDCl_3$. 37

Table S8. NMR data of 6*β*-hydroxyconfertifolin (**5**) in $CDCl_3$..............................38

Table S9. NMR data of 6*β*-acetoxyconfertifolin (**6**) in $CDCl_3$...............................39

Table S10. NMR data of 3*β*-hydroxyconfertifolin (**7**) in $CDCl_3$............................40

Table S11. NMR data of ustidrimane B (**8**) in $CDCl_3$. 41

Table S12. NMR data of ustidrimane C (**9**) in DMSO-d_6. 42

Table S13. NMR data of ustidrimane D (**10**) in $CDCl_3$..43

Table S14. NMR data of ustidrimane E (**11**) in $CDCl_3$. 44

Table S15. NMR data of ustidrimane F (**12**) in $CDCl_3$. 45

Table S16. NMR data of ustidrimane G (**13**) in $CDCl_3$.......................................46
Table S17. NMR data of ustidrimane H (**14**) in $CDCl_3$.......................................47
Table S18. NMR data of ustidrimane I (**15**) in DMSO-d_6.....................................48
Table S19. NMR data of ustidrimane J (**16**) in DMSO-d_6. 49
Table S20. NMR data of ustidrimane K (**17**) in $CDCl_3$.......................................50
Table S21. NMR data of ustidrimane L (**18**) in $CDCl_3$. 51
Table S22. NMR data of ustidrimane M (**19**) in $CDCl_3$.......................................52
Table S23. NMR data of ustidrimane N (**20**) in $CDCl_3$.......................................53
Table S24. NMR data of ustidrimane O (**21**) in DMSO-d_6. 54
Table S25. NMR data of ustidrimane P (**22**) in $CDCl_3$. 55
Table S26. NMR data of ustidrimane Q (**23**) in DMSO-d_6. 56
Supplementary Figures 57
Figure S1. Comparative analysis of the *dri* and *ast* clusters. Amino acid identities of the respective homologous proteins are given in percent. 57
Figure S2. LC-MS chromatogram of the metabolites from *A. ustus* NRRL 280. Absorptions at UV 210 nm are displayed. 57
Figure S3. UV spectra of compounds **1–23**. 58
Figure S4. MS spectra of compounds **1–23**. 61
Figure S5. PCR verification of *A. nidulans* recombinant strains. 62
Figure S6. PCR verification of single deletion mutants. 63
Figure S7. PCR verification of double deletion mutants. 64
Figure S8. The ^{1}H NMR spectrum of ustidrimane A (**1**) in $CDCl_3$ (500 MHz). 65
Figure S9. The ^{13}C NMR spectrum of ustidrimane A (**1**) in $CDCl_3$ (125 MHz). 66
Figure S10. The HSQC spectrum of ustidrimane A (**1**) in $CDCl_3$..........................67
Figure S11. The HMBC spectrum of ustidrimane A (**1**) in $CDCl_3$.........................68
Figure S12. The ^{1}H-^{1}H COSY spectrum of ustidrimane A (**1**) in $CDCl_3$. 69
Figure S13. The NOESY spectrum of ustidrimane A (**1**) in $CDCl_3$. 70
Figure S14. The ^{1}H NMR spectrum of drim-8-ene-11-ol (**3**) in $CDCl_3$ (400 MHz). 71
Figure S15. The ^{13}C NMR spectrum of drim-8-ene-11-ol (**3**) in $CDCl_3$ (100 MHz). 72
Figure S16. The ^{1}H NMR spectrum of confertifolin (**4**) in $CDCl_3$ (400 MHz). 73
Figure S17. The ^{13}C NMR spectrum of confertifolin (**4**) in $CDCl_3$ (100 MHz). 74
Figure S18. The HSQC spectrum of confertifolin (**4**) in $CDCl_3$. 75

Figure S19. The ^{1}H NMR spectrum of 6*β*-hydroxyconfertifolin (**5**) in $CDCl_3$ (400 MHz). 76
Figure S20. The ^{13}C NMR spectrum of 6*β*-hydroxyconfertifolin (**5**) in $CDCl_3$ (100 MHz). 77
Figure S21. The HSQC spectrum of 6*β*-hydroxyconfertifolin (**5**) in $CDCl_3$. 78
Figure S22. The HMBC spectrum of 6*β*-hydroxyconfertifolin (**5**) in $CDCl_3$. 79
Figure S23. The ^{1}H-^{1}H COSY spectrum of 6*β*-hydroxyconfertifolin (**5**) in $CDCl_3$. 80
Figure S24. The NOESY spectrum of 6*β*-hydroxyconfertifolin (**5**) in $CDCl_3$........81
Figure S25. The ^{1}H NMR spectrum of 6*β*-acetoxyconfertifolin (**6**) in $CDCl_3$ (400 MHz). 82
Figure S26. The ^{13}C NMR spectrum of 6*β*-acetoxyconfertifolin (**6**) in $CDCl_3$ (100 MHz). 83
Figure S27. The HSQC spectrum of 6*β*-acetoxyconfertifolin (**6**) in $CDCl_3$. 84
Figure S28. The HMBC spectrum of 6*β*-acetoxyconfertifolin (**6**) in $CDCl_3$. 85
Figure S29. The ^{1}H-^{1}H COSY spectrum of 6*β*-acetoxyconfertifolin (**6**) in $CDCl_3$.86
Figure S30. The NOESY spectrum of 6*β*-acetoxyconfertifolin (**6**) in $CDCl_3$.........87
Figure S31. The ^{1}H NMR spectrum of 3*β*-hydroxyconfertifolin (**7**) in $CDCl_3$ (400 MHz). 88
Figure S32. The ^{13}C NMR spectrum of 3*β*-hydroxyconfertifolin (**7**) in $CDCl_3$ (100 MHz). 89
Figure S33. The HSQC spectrum of 3*β*-hydroxyconfertifolin (**7**) in $CDCl_3$. 90
Figure S34. The HMBC spectrum of 3*β*-hydroxyconfertifolin (**7**) in $CDCl_3$. 91
Figure S35. The ^{1}H-^{1}H COSY spectrum of 3*β*-hydroxyconfertifolin (**7**) in $CDCl_3$. 92
Figure S36. The ^{1}H NMR spectrum of ustidrimane B (**8**) in $CDCl_3$ (400 MHz)....93
Figure S37. The ^{13}C NMR spectrum of ustidrimane B (**8**) in $CDCl_3$ (100 MHz)...94
Figure S38. The HSQC spectrum of ustidrimane B (**8**) in $CDCl_3$. 95
Figure S39. The HMBC spectrum of ustidrimane B (**8**) in $CDCl_3$. 96
Figure S40. The ^{1}H-^{1}H COSY spectrum of ustidrimane B (**8**) in $CDCl_3$................97
Figure S41. The NOESY spectrum of ustidrimane B (**8**) in $CDCl_3$.........................98
Figure S42. The ^{1}H NMR spectrum of ustidrimane C (**9**) in DMSO-d_6 (400 MHz). 99
Figure S43. The ^{13}C NMR spectrum of ustidrimane C (**9**) in DMSO-d_6 (100 MHz). 100

Figure S44. The HSQC spectrum of ustidrimane C (**9**) in DMSO-d_6. 101
Figure S45. The HMBC spectrum of ustidrimane C (**9**) in DMSO-d_6. 102
Figure S46. The ^{1}H-^{1}H COSY spectrum of ustidrimane C (**9**) in DMSO-d_6. 103
Figure S47. The NOESY spectrum of ustidrimane C (**9**) in DMSO-d_6. 104
Figure S48. The ^{1}H NMR spectrum of ustidrimane D (**10**) in $CDCl_3$ (400 MHz). 105
Figure S49. The ^{13}C NMR spectrum of ustidrimane D (**10**) in $CDCl_3$ (100 MHz). 106
Figure S50. The HSQC spectrum of ustidrimane D (**10**) in $CDCl_3$. 107
Figure S51. The HMBC spectrum of ustidrimane D (**10**) in $CDCl_3$. 108
Figure S52. The ^{1}H-^{1}H COSY spectrum of ustidrimane D (**10**) in $CDCl_3$. 109
Figure S53. The NOESY spectrum of ustidrimane D (**10**) in $CDCl_3$. 110
Figure S54. The ^{1}H NMR spectrum of ustidrimane E (**11**) in $CDCl_3$ (400 MHz). 111
Figure S55. The HSQC spectrum of ustidrimane E (**11**) in $CDCl_3$. 112
Figure S56. The HMBC spectrum of ustidrimane E (**11**) in $CDCl_3$. 113
Figure S57. The ^{1}H NMR spectrum of ustidrimane F (**12**) in $CDCl_3$ (400 MHz). 114
Figure S58. The ^{13}C NMR spectrum of ustidrimane F (**12**) in $CDCl_3$ (100 MHz). 115
Figure S59. The HSQC spectrum of ustidrimane F (**12**) in $CDCl_3$. 116
Figure S60. The HMBC spectrum of ustidrimane F (**12**) in $CDCl_3$. 117
Figure S61. The ^{1}H-^{1}H COSY spectrum of ustidrimane F (**12**) in $CDCl_3$. 118
Figure S62. The NOESY spectrum of ustidrimane F (**12**) in $CDCl_3$. 119
Figure S63. The ^{1}H NMR spectrum of ustidrimane G (**13**) in $CDCl_3$ (400 MHz). 120
Figure S64. The ^{13}C NMR spectrum of ustidrimane G (**13**) in $CDCl_3$ (100 MHz). 121
Figure S65. The HSQC spectrum of ustidrimane G (**13**) in $CDCl_3$. 122
Figure S66. The HMBC spectrum of ustidrimane G (**13**) in $CDCl_3$. 123
Figure S67. The ^{1}H-^{1}H COSY spectrum of ustidrimane G (**13**) in $CDCl_3$. 124
Figure S68. The NOESY spectrum of ustidrimane G (**13**) in $CDCl_3$. 125
Figure S69. The ^{1}H NMR spectrum of ustidrimane H (**14**) in $CDCl_3$ (400 MHz). 126
Figure S70. The ^{13}C NMR spectrum of ustidrimane H (**14**) in $CDCl_3$ (100 MHz). 127
Figure S71. The HSQC spectrum of ustidrimane H (**14**) in $CDCl_3$. 128

Figure S72. The HMBC spectrum of ustidrimane H (**14**) in $CDCl_3$. 129
Figure S73. The ^1H-^1H COSY spectrum of ustidrimane H (**14**) in $CDCl_3$. 130
Figure S74. The NOESY spectrum of ustidrimane H (**14**) in $CDCl_3$....................131
Figure S75. The ^1H NMR spectrum of ustidrimane I (**15**) in DMSO-d_6 (400 MHz). 132
Figure S76. The ^{13}C NMR spectrum of ustidrimane I (**15**) in DMSO-d_6 (100 MHz). 133
Figure S77. The HSQC spectrum of ustidrimane I (**15**) in DMSO-d_6. 134
Figure S78. The HMBC spectrum of ustidrimane I (**15**) in DMSO-d_6. 135
Figure S79. The ^1H-^1H COSY spectrum of ustidrimane I (**15**) in DMSO-d_6. 136
Figure S80. The NOESY spectrum of ustidrimane I (**15**) in DMSO-d_6................137
Figure S81. The ^1H NMR spectrum of ustidrimane J (**16**) in DMSO-d_6 (400 MHz). 138
Figure S82. The ^{13}C NMR spectrum of ustidrimane J (**16**) in DMSO-d_6 (100 MHz). 139
Figure S83. The HSQC spectrum of ustidrimane J (**16**) in DMSO-d_6. 140
Figure S84. The HMBC spectrum of ustidrimane J (**16**) in DMSO-d_6. 141
Figure S85. The ^1H-^1H COSY spectrum of ustidrimane J (**16**) in DMSO-d_6........142
Figure S86. The ^1H NMR spectrum of ustidrimane K (**17**) in $CDCl_3$ (400 MHz). 143
Figure S87. The ^{13}C NMR spectrum of ustidrimane K (**17**) in $CDCl_3$ (100 MHz). 144
Figure S88. The HSQC spectrum of ustidrimane K (**17**) in $CDCl_3$. 145
Figure S89. The HMBC spectrum of ustidrimane K (**17**) in $CDCl_3$. 146
Figure S90. The ^1H-^1H COSY spectrum of ustidrimane K (**17**) in $CDCl_3$. 147
Figure S91. The NOESY spectrum of ustidrimane K (**17**) in $CDCl_3$....................148
Figure S92. The ^1H NMR spectrum of ustidrimane L (**18**) in $CDCl_3$ (400 MHz). 149
Figure S93. The ^{13}C NMR spectrum of ustidrimane L (**18**) in $CDCl_3$ (100 MHz). 150
Figure S94. The HSQC spectrum of ustidrimane L (**18**) in $CDCl_3$......................151
Figure S95. The HMBC spectrum of ustidrimane L (**18**) in $CDCl_3$......................152
Figure S96. The ^1H-^1H COSY spectrum of ustidrimane L (**18**) in $CDCl_3$. 153
Figure S97. The NOESY spectrum of ustidrimane L (**18**) in $CDCl_3$. 154

Figure S98. The ^{1}H NMR spectrum of ustidrimane M (**19**) in $CDCl_3$ (400 MHz). 155
Figure S99. The ^{13}C NMR spectrum of ustidrimane M (**19**) in $CDCl_3$ (100 MHz). 156
Figure S100. The HSQC spectrum of ustidrimane M (**19**) in $CDCl_3$. 157
Figure S101. The HMBC spectrum of ustidrimane M (**19**) in $CDCl_3$. 158
Figure S102. The ^{1}H-^{1}H COSY spectrum of ustidrimane M (**19**) in $CDCl_3$.........159
Figure S103. The NOESY spectrum of ustidrimane M (**19**) in $CDCl_3$. 160
Figure S104. The ^{1}H NMR spectrum of ustidrimane N (**20**) in $CDCl_3$ (400 MHz). 161
Figure S105. The ^{13}C NMR spectrum of ustidrimane N (**20**) in $CDCl_3$ (100 MHz). 162
Figure S106. The HSQC spectrum of ustidrimane N (**20**) in $CDCl_3$. 163
Figure S107. The HMBC spectrum of ustidrimane N (**20**) in $CDCl_3$. 164
Figure S108. The ^{1}H-^{1}H COSY spectrum of ustidrimane N (**20**) in $CDCl_3$. 165
Figure S109. The NOESY spectrum of ustidrimane N (**20**) in $CDCl_3$.................166
Figure S110. The ^{1}H NMR spectrum of ustidrimane O (**21**) in DMSO-d_6 (400 MHz). 167
Figure S111. The ^{13}C NMR spectrum of ustidrimane O (**21**) in DMSO-d_6 (100 MHz). 168
Figure S112. The HSQC spectrum of ustidrimane O (**21**) in DMSO-d_6. 169
Figure S113. The HMBC spectrum of ustidrimane O (**21**) in DMSO-d_6. 170
Figure S114. The ^{1}H-^{1}H COSY spectrum of ustidrimane O (**21**) in DMSO-d_6. ...171
Figure S115. The ^{1}H NMR spectrum of ustidrimane P (**22**) in $CDCl_3$ (400 MHz). 172
Figure S116. The ^{13}C NMR spectrum of ustidrimane P (**22**) in $CDCl_3$ (100 MHz). 173
Figure S117. The HSQC spectrum of ustidrimane P (**22**) in $CDCl_3$. 174
Figure S118. The HMBC spectrum of ustidrimane P (**22**) in $CDCl_3$. 175
Figure S119. The ^{1}H-^{1}H COSY spectrum of ustidrimane P (**22**) in $CDCl_3$. 176
Figure S120. The ^{1}H NMR spectrum of ustidrimane Q (**23**) in DMSO-d_6 (400 MHz). 177
Figure S121. The ^{13}C NMR spectrum of ustidrimane Q (**23**) in DMSO-d_6 (100 MHz). 178
Figure S122. The HSQC spectrum of ustidrimane Q (**23**) in DMSO-d_6. 179

Figure S123. The HMBC spectrum of ustidrimane Q (**23**) in DMSO-d_6. 180
Figure S124. The ^{1}H-^{1}H COSY spectrum of ustidrimane Q (**23**) in DMSO-d_6. ... 181
Figure S125. The ECD spectra of the isolated compounds **1** and **3–23** in MeOH. 182
Figure S126. LC-MS chromatograms of the metabolites from *A. nidulans* MW57 and MW58. Absorptions at UV 210 nm are displayed. 183
Figure S127. SDS-PAGE analysis of purified recombinant DriG. 184
Figure S128. Determination of kinetic parameters of DriG toward ustidrimane I (**15**). 185
References 186

Experiment Procedures

1. Media, strains, and growth conditions

All strains used in this study are listed in Table S2. *Escherichia coli* DH5α and XL10-Gold were cultivated in liquid lysogeny broth (LB) medium or on agar plates at 37 °C. Ampicillin (50 µg/mL) were used for selection of the recombinant *E. coli* strains. *Saccharomyces cerevisiae* BJ5464-npgA was cultivated at 30 °C in yeast extract peptone dextrose (YPD) medium. Synthetic complete medium without uracil (SC-Ura) was used for selection.[1]

Aspergillus ustus NRRL 280 was cultivated at 30 °C on potato dextrose agar (PDA) plates for spore production and on rice medium (20 g of rice with 30 mL of H_2O in a 250 mL flask).

Aspergillus nidulans LO8030[2] were grown for sporulation at 37 °C on minimal medium supplemented with riboflavin, pyridoxine, uridine and uracil as reported previously.[3] The recombinant *A. nidulans* strains were cultivated at room temperature on liquid minimal medium (LMM) supplemented with pyridoxine, uridine and uracil. The single-gene deletion *A. nidulans* mutants were cultivated at room temperature in LMM medium supplemented with uridine and uracil. The double-gene deletion *A. nidulans* mutants were cultivated at room temperature in LMM medium without supplements. For each recombinant strain, two or three independent transformants were separately cultivated and analyzed under the same conditions. Consistent metabolite profiles were obtained.

2. Genomic DNA extraction

For genomic DNA extraction, mycelia of *A. nidulans* and *A. ustus* strains were harvested after cultivation in LMM or PD for 2 days and blotted dry with paper towels. Genomic DNA was isolated as previously described.[4] The mycelia were collected and disrupted with glass beads in 400 µL of LETS buffer (10 mM Tris-HCl, pH 8.0, 20 mM EDTA, 0.5% SDS, and 0.1 M LiCl) using a MiniLys homogenizer (Bertin Technologies, Montigny-le-Bretonneux, France) at full speed for 4 min. Then 300 µL of LETS buffer was added and the resulting cell lysate was extracted with 700 µL of phenol/chloroform/isoamyl alcohol (25:24:1, v/v/v). Genomic DNA was precipitated from the aqueous phase by adding two volumes of absolute ethanol, washed with 1 mL of 70% ethanol, air-dried, and dissolved in distilled H_2O.

3. RNA isolation and cDNA synthesis

For RNA isolation from *Aspergillus ustus* NRRL 280, the fungus was cultivated in 5 mL LMM for 5 days at 30 °C. Total RNA was extracted using the Fungal RNA Mini Kit, E.Z.N.A.® Fungal RNA Kit (VWR/Omega Bio-tek), according to the manufacturer's instructions. First-strand cDNA was synthesized using the ProtoScript® II First Strand cDNA Synthesis Kit (New England BioLabs) with oligo-dT primers.

4. PCR amplification, cloning, and plasmid construction

Plasmids and primers used in this study are listed in Tables S3 and S4, respectively. The primers were synthesized by Seqlab GmbH. PCR amplification was carried out using the Phusion High-

Fidelity DNA polymerase from New England Biolabs.

Plasmids for heterologous expression and gene deletion were constructed by homologous recombination in *E. coli* XL10-Gold or *S. cerevisiae* BJ5464-npgA.

The plasmids for heterologous expression in *A. nidulans* LO8030[2] were constructed by PCR amplification from genomic DNA of *A. ustus* NRRL 280. The fragments were subsequently cloned into BamHI-linearized vector pYWB2[5] containing the flanking regions of the *wA* gene and the *afribo* selection marker, or into SfoI-linearized vector pJN017[6] containing the flanking regions of the *wA* gene, the *gpdA* promoter, and the *afribo* selection marker. The PCR-amplified genomic fragments containing 25–35 bp overlaps homologous to the ends of the linearized empty vector were assembled into the vector.

To generate plasmids pMW31–36 and pMW39 for single-gene deletion, approximately 1.5-kb 5′- and 3′-flanking regions were PCR-amplified from genomic DNA of *A. nidulans* MW11. The *afpyro* marker fragment was PCR-amplified from pYWB1,[1] and the vector backbone containing the *ampR* and *URA3* elements was PCR-amplified from pYWB2.[5] These fragments were assembled in yeast or in *E. coli* as described above. The resulting deletion constructs were linearized by SwaI digestion and used to transform *A. nidulans* LO8030,[2] yielding strains MW12, MW13, MW17–MW19, MW22, and MW23 (Table S2).

To generate plasmids pMW79–pMW82 for double-gene deletion, approximately 1.5-kb 5′- and 3′-flanking regions were PCR-amplified from genomic DNA of *A. nidulans* MW13. The *pyrG* marker fragment was PCR-amplified from pZL69,[7] and the vector backbone containing the *ampR* and *URA3* elements was PCR-amplified from pYWB2.[5] These fragments were assembled in yeast as described above. The resulting deletion constructs were linearized by SwaI digestion and used to transform *A. nidulans* LO8030,[2] yielding strains MW79–MW82 (Table S2).

5. Genetic manipulation in *A. nidulans*

A. nidulans LO8030[2] was used as the recipient host. Fungal protoplast preparation and transformation were performed as described previously.[8] The expression plasmids pMW30 (*driABCDEFGHIJKL*), pMW48 (*driC*), pMW49 (*driD*), pMW50 (*driJ*), pMW56 (*driAIKL*), pMW57 (*driAEIKL*), pMW58 (*driAEFIKL*), pMW60 (*driDJ*), pMW74 (*driEFGH*), pMW86 (*driGH*), pMW88 (*driG*), and pMW90 (*driH*) were introduced into *A. nidulans* LO8030,[2] yielding strains MW11, MW48, MW49, MW50, MW56, MW57, MW58, MW60, MW74, MW86, MW88, and MW90, respectively. Successful deletion of the target genes was confirmed by PCR amplification using gDNA as the template (Figure S5).

For single gene deletion in *A. nidulans* MW11, the deletion vectors, pMW31–pMW36 and pMW39, were linearized and then used to transform *A. nidulans* MW11, yielding the deletion strains *A. nidulans* MW12 (Δ*driG*), MW13 (Δ*driJ*), MW17 (Δ*driE*), MW18 (Δ*driF*), MW19 (Δ*driC*), MW22 (Δ*driD*), and MW23 (Δ*driH*). Successful deletion of the target genes was confirmed by PCR amplification using gDNA as the template (Figure S6).

To get double deletion mutants, the deletion vectors, pMW79–pMW82, were used to transform *A. nidulans* MW13, yielding the double-deletion strains *A. nidulans* MW79 (Δ*driJ*Δ*driE*),

MW80 (Δ*driJ*Δ*driF*), MW81 (Δ*driJ*Δ*driG*), and MW82 (Δ*driJ*Δ*driH*). Successful deletion of the target genes was confirmed by PCR amplification using gDNA as the template (Figure S7).

6. Overproduction and purification of DriG

The expression plasmid pMW51 was transformed into *E. coli* BL21(DE3). Terrific Broth (TB) medium containing 1.2 % (m/v) of tryptone, 2.4 % (m/v) of yeast extract, 0.4 % (v/v) of glycerol, 17 mM KH_2PO4 and 72 mM K_2HPO4 supplemented with kanamycin (50 µg mL^{-1}) was used for cultivation. DriG overproduction was induced with 0.5 mM isopropyl thiogalactoside (IPTG) at 25 °C for 16 h. The recombinant N-terminal His_6-tagged DriG protein was purified by Ni-NTA affinity chromatography (Qiagen) using the method described previously.[9] The concentration of the purified protein was determined using a NanoDrop 2000c spectrophotometer (Thermo Scientific), and the protein yield was approximately 3.5 mg per liter of culture. The purified protein was analyzed by SDS-PAGE using a 12% acrylamide gel, which showed a protein band with the expected size at 30.1 kDa (Figure S127).

7. *In vitro* assays of DriG

To investigate the conversion of **15** and **21** by DriG, the enzymatic reactions were performed in a 50 µL system containing DriG (2.5 µM), substrate (0.1 mM), NAD^+ (5 mM), phenazine methosulfate (PMS, 20 µM) and Tris-HCl buffer (50 mM, pH 8.0) at 25 °C. Incubations with heat-inactivated protein were used as controls. The reactions were terminated by addition of 50 µL of methanol and monitored using LC-MS.

For protein-dependent assays, the reaction mixtures containing **15** (0.02 mM) and DriG at final concentrations of 0, 0.01, 0.02, 0.05, 0.10, 0.20, 0.50, 1.0, or 2.0 µM were incubated for 5 min. For time-dependent assays, the reaction mixtures containing **15** (0.02 mM) and DriG (0.20 µM) were incubated for 0, 1, 2, 3, 4, 5 or 10 min. All reactions additionally contained NAD^+ (5 mM), PMS (20 µM) and were performed at 25 °C in a total volume of 50 µL in Tris-HCl buffer (50 mM, pH 8.0). All the assays were performed in duplicates.

For steady-state kinetic analysis, the reaction mixtures (50 µL, in 50 mM Tris-HCl buffer, pH 8.0) containing DriG (0.20 µM), NAD^+ (5 mM), PMS (20 µM), and varying concentrations of **15** (0, 0.02, 0.05, 0.1, 0.2, 0.5, 1.0, 2.0 mM) were incubated at 25 °C for 3 min. Apparent kinetic parameters were obtained by nonlinear regression fitting of the initial rate data to the Michaelis–Menten equation using GraphPad Prism 8.2. All measurements were performed in triplicate.

8. LC-MS analysis

LC–MS analysis of the fungal extracts and isolated compounds was performed on an Agilent 1260 HPLC system coupled to a Bruker microTOF-Q III mass spectrometer, using a VDSpher PUR 100 C18-M-SE column (3 µm, 150 × 2.0 mm; VDS Optilab Chromatographietechnik GmbH, Bremen, Germany). Compounds were separated at a flow rate of 0.3 mL/min with a 30 min linear gradient from 5% to 100% MeCN in H_2O, with both solvents containing 0.1% (v/v) HCOOH. Data acquisition and analysis were performed using Compass DataAnalysis 4.2 software (Bruker Daltonik). UV chromatograms were illustrated for absorption at 210 nm.

9. NMR analysis

NMR spectra of the isolated compounds were recorded on a JEOL ECA-500 or ECZ-400S spectrometer (JEOL, Akishima, Tokyo, Japan). Samples were dissolved in $CDCl_3$ or DMSO-*d*$_6$. All spectra were processed using MestReNova 15.0.0 software (Mestrelab Research, Santiago de Compostela, Spain).

10. Large-scale fermentation, extraction, and isolation of secondary metabolites

For the isolation of compound **1** from *A. ustus* NRRL 280, fresh spores were inoculated into 10 × 1 L flasks, each containing 100 g of rice and 150 mL of H_2O, and cultivated for 14 days. For the isolation of structurally interesting compounds from different *A. nidulans* transformants, fresh spores were inoculated into 16 × 1 L flasks, each containing 500 mL of LMM, and cultivated for 7 days.

The culture was extracted twice with EtOAc and the combined extracts were concentrated under reduced pressure to afford crude extracts.

The crude extract of *A. ustus* NRRL 280 were analyzed by LC-MS, revealing a peak corresponding to ustidrimane A **(1)** at t_R = 16.2 min. The dried extract was then subjected to silica gel column chromatography using a gradient of petroleum ether/ethyl acetate (10:1–1:1, v/v) to afford 32 fractions (Fr. 1–32). Fr. 26 was further purified by semipreparative HPLC using a CH_3CN/H_2O (0.1% HCOOH) gradient (25:75–34:66, v/v, 20 min) to yield pure **1**, at t_R = 35 min.

A peak corresponding to drim-8-ene-11-ol **(3)** at t_R = 27.8 min was detected in the crude extract of *A. nidulans* MW79 in LC-MS. The crude extract was subjected to preparative HPLC using a CH_3CN/H_2O (0.1% HCOOH) gradient (50:50–80:20, v/v, 40 min) to afford a fraction at t_R = 21–24 min. The fraction was further purified by semipreparative HPLC using a CH_3CN/H_2O (0.1% HCOOH) gradient (50:50–80:20, v/v, 10 min) to yield pure **3**, t_R = 27 min.

LC-MS analysis of crude extract of *A. nidulans* MW17 revealed peaks corresponding to confertifolin (**4**), 6*β*-hydroxyconfertifolin **(5)**, and 6*β*-acetoxyconfertifolin (**6**) at t_R = 25.5, 20.0, and 22.6 min, respectively. The crude extract was subjected to preparative HPLC using a CH_3CN/H_2O (0.1% HCOOH) gradient (30:70–60:40, v/v, 30 min) to afford fractions at t_R = 18–19, 25–26 and 30–31 min. The 30-31 min fraction was further purified by semipreparative HPLC using CH_3CN/H_2O (0.1% HCOOH, 61:39, v/v) to yield **4**, t_R = 32 min. The 18–19 min fraction was further purified by semipreparative HPLC using CH_3CN/H_2O (0.1% HCOOH, 60:40, v/v) to yield **5**, t_R = 19 min. The 25–26 min fraction was further purified by semipreparative HPLC using CH_3CN/H_2O (0.1% HCOOH, 63:39, v/v) to yield **6**, t_R = 27 min.

LC-MS analysis of crude extract of *A. nidulans* MW17 showed another peak corresponding to 3*β*-hydroxyconfertifolin (**7**) at t_R = 15.3 min. The crude extract was subjected to preparative HPLC using a CH_3CN/H_2O (0.1% HCOOH) gradient (10:90–60:40, v/v,40 min) to a afford fraction at t_R = 17–20 min. The fraction was further purified by semipreparative HPLC using

CH_3CN/H_2O (0.1% HCOOH, 32:68) to yield pure **7**, t_R = 19 min.

The crude extract of *A. nidulans* MW18 was analyzed by LC-MS, revealing a peak corresponding to ustidrimane B **(8)** at t_R = 13.8 min. The crude extract was subjected to preparative HPLC using a CH_3CN/H_2O (0.1% HCOOH) gradient (5:95–40:60, v/v, 40 min) to afford a fraction at t_R = 25–27 min. The fraction was further purified by semipreparative HPLC using CH_3CN/H_2O (0.1% HCOOH, 22:78, v/v) to yield **8**, t_R = 30 min.

LC-MS analysis of crude extract of *A. nidulans* MW18 revealed peaks corresponding to ustidrimane C (**9**), ustidrimane D **(10)**, and ustidrimane G **(13),** respectively at t_R = 13.8 ,16.9 and 17.5 min, respectively. The crude extract was subjected to preparative HPLC using a CH_3CN/H_2O (0.1% HCOOH) gradient (5:95–60:40, v/v, 30 min) to afford a fraction at t_R = 15–21 min. The fraction was further purified by semipreparative HPLC using a CH_3CN/H_2O (0.1% HCOOH) gradient (25:75–34:66, v/v, 20 min) to yield pure **9**, **10** and **13**, t_R = 20, 38, and 44 min, respectively.

The crude extract of *A. nidulans* MW18 was analyzed by LC-MS, revealing peaks corresponding to ustidrimane E **(11)**, ustidrimane F **(12)** and ustidrimane H **(14)** at t_R = 17.3, 17.4 and 18.8 min, respectively. The crude extract was subjected to preparative HPLC using a CH_3CN/H_2O (0.1% HCOOH) gradient (10:90–60:40, v/v, 30 min) to afford fractions at t_R = 19–21 and 21–24 min. The 19–21 min fraction was further purified by semipreparative HPLC using a CH_3CN/H_2O (0.1% HCOOH) gradient (10:90–15:85, v/v, 10 min, followed by 28:72) to yield **11** and **12**, t_R = 49 and 51 min, respectively. The 21–24 min fraction was further purified by semipreparative HPLC using CH_3CN/H_2O (0.1% HCOOH, 42:58, v/v) to yield **14**, t_R = 21 min.

A peak corresponding to ustidrimane I **(15)** at t_R = 12.2 min was detected in the crude extract of *A. nidulans* MW12. The crude extract was subjected to preparative HPLC using a CH_3CN/H_2O (0.1% HCOOH) gradient (5:95–40:60, v/v, 40 min) to afford pure **15**, t_R = 20 min.

The crude extract of *A. nidulans* MW23 was analyzed by LC-MS, showing a peak corresponding to ustidrimane J **(16)** at t_R = 11.5 min. The crude extract was subjected to preparative HPLC using a CH_3CN/H_2O (0.1% HCOOH) gradient (2:98–50:50, v/v, 40 min) to afford a fraction at t_R = 20–22 min. The fraction was further purified by semipreparative HPLC using CH_3CN/H_2O (0.1% HCOOH, 22:78, v/v) to yield **16**, t_R = 15 min.

LC-MS analysis of crude extract of *A. nidulans* MW13 revealed a peak corresponding to ustidrimane K **(17)** at t_R = 17.9 min. The crude extract was subjected to preparative HPLC using a CH_3CN/H_2O (0.1% HCOOH) gradient (20:80–50:50, v/v, 40 min) to afford a fraction at t_R = 22–24 min. The fraction was further purified by semipreparative HPLC using CH_3CN/H_2O (0.1% HCOOH, 37:63, v/v) to yield **17**, t_R = 33 min.

A peak corresponding to ustidrimane L **(18)** was detected in the crude extract of *A. nidulans* MW22 at t_R = 23.5 min. The crude extract was subjected to preparative HPLC using a CH_3CN/H_2O (0.1% HCOOH) gradient (5:95–100:0, v/v, 40 min) to afford a fraction at t_R = 17–23 min. The fraction was further purified by semipreparative HPLC using CH_3CN/H_2O (0.1% HCOOH, 56:44, v/v) to yield **18**, t_R = 29 min.

A peak corresponding to ustidrimane M **(19)** was detected in crude extract of *A. nidulans* MW19 at t_R = 16.7 min. The crude extract was subjected to preparative HPLC using a CH_3CN/H_2O (0.1% HCOOH) gradient (8:92–40:60, v/v, 30 min) to afford **19**, t_R =30–32 min.

The crude extract of *A. nidulans* MW57 was analyzed by LC-MS, revealing a peak corresponding to ustidrimane N **(20)** at t_R = 18.1 min. The crude extract was subjected to preparative HPLC using a CH_3CN/H_2O (0.1% HCOOH) gradient (20:80–60:40, v/v, 40 min) to a afford fraction at t_R = 19–21 min. The fraction was further purified by semipreparative HPLC using CH_3CN/H_2O (0.1% HCOOH, 40:60, v/v) to yield **20**, t_R = 21 min.

The crude extract of *A. nidulans* MW82 was analyzed by LC-MS, revealing a peak corresponding to ustidrimane O **(21)** at t_R = 13.9 min. The crude extract was subjected to preparative HPLC using a CH_3CN/H_2O (0.1% HCOOH) gradient (10:90–50:50, v/v, 40 min) to a afford fraction eluting at t_R = 17–20 min. The fraction was further purified by semipreparative HPLC using CH_3CN/H_2O (0.1% HCOOH, 25:75, v/v) to yield **21**, t_R = 25 min.

To isolate ustidrimane P **(22),** big-scale enzymatic reaction was carried on. The enzymatic reaction was performed in a 10 × 5 mL system containing DriG (3.7 µg/µL), compound **15** (0.1 mM), NAD^+ (5 mM), phenazine methosulfate (PMS, 20 µM) and Tris-HCl buffer (50 mM, pH 8.0) at 25 °C. After one night of cultivation with shaking at room temperature, the reactions were terminated by addition of 10 × 5 mL of methanol and monitored using LC-MS, revealing a peak corresponding to **22** at t_R = 13.8 min. The crude extract was subjected to preparative HPLC using a CH_3CN/H_2O gradient (0.1% HCOOH, 5:95–100:0, v/v, 40 min) to afford a fraction at t_R = 11–13 min. The fraction was further purified by semipreparative HPLC using CH_3CN/H_2O (0.1% HCOOH, 20:80, v/v) to yield **22**, t_R = 27 min.

For the isolation of ustidrimane Q **(23)**, big-scale feeding experiment was conducted. **17** was dissolved in DMSO to prepare a 20 mM stock solution. *A. nidulans* MW50 was first cultivated in 10 × 600 mL LMM cultures for 3 days at room temperature with shaking at 200 rpm. Subsequently, 120 µL of the stock solution was added to each culture to give a final concentration of 20 µM. After an additional 2 days of cultivation with shaking at room temperature, the culture was extracted twice with ethyl acetate. The crude extract was analyzed by LC-MS, revealing a peak corresponding to **23** at t_R = 13.1 min. The crude extract was subjected to preparative HPLC using a CH_3CN/H_2O gradient (0.1% HCOOH, 10:90–40:60, v/v, 40 min) to afford a fraction at t_R = 13–15 min. The fraction was further purified by semipreparative HPLC using a CH_3CN/H_2O (0.1% HCOOH) gradient (10:90–25:75, v/v, 10 min) to yield **23**, t_R = 25 min.

11. Structural elucidation

Ustidrimane A **(1)**. NMR data of **1** are similar to those of spinulactone A,[10] suggesting that both compounds share the same drimane-type sesquiterpene skeleton. The major difference was two additional carbon signals at δ_C 169.4 and 21.4, together with a methyl singlet at δ_H 2.08, indicating an additional acetyl group in **1**. In addition, the signal of proton H-6 was markedly shifted downfield from δ_H 4.38 in spinulactone A to δ_H 5.44 in **1**, whereas signal of carbon C-6 remained oxygenated at δ_C 68.46, indicating acetylation of the hydroxy group at C-6 in **1.** (Figures S8 and S9). The planar structure of **1** was further confirmed by HSQC, ^{1}H-^{1}H COSY, and HMBC spectroscopic analyses (Figures S10–S13) correlations were observed between H-13 (δ_H 4.70) and H-15 (δ_H 1.30), as well as between H-5 (δ_H 2.02) and H-6 (δ_H 5.44), suggesting their spatial proximity. Furthermore, the ECD spectrum (Figure S125) of **1** showed a similar pattern to that of spinulactone A,[10] supporting the assignment of the same absolute configuration. Accordingly, compound **1** was established as the 6-*O*-acetyl derivative of spinulactone A, and was named as ustidrimane A.

Compounds **3**, **4**, **5**, **6**, and **7**: Comparison of the spectroscopic data with those reported in the literatures identified them as drim-8-ene-11-ol (**3**),[11] confertifolin (**4**),[12,13] 6*β*-hydroxyconfertifolin (**5**),[14] 6*β*-acetoxyconfertifolin (**6**),[15] and 3*β*-hydroxyconfertifolin (**7**), respectively.[16]

Ustidrimane B **(8)**. Compared with **4**, compound **8** was assigned as the C-6/C-14 dihydroxylated derivative. The replacement of the C-14 methyl group by an oxygenated methylene group was evidenced by the disappearance of the C-14 methyl carbon (δ_C 33.4) and proton (δ_H 0.95) signals and the appearance of an oxymethylene carbon resonance at δ_C 71.9 with corresponding proton signals at δ_H 3.64 and 3.23, together with the conversion of the C-6 methyl carbon resonance (δ_C 18.2) into an oxygenated methylene carbon resonance (δ_C 65.0), proved hydroxylation at C-14 and C-6 (Figures S36 and S37). The assignment was further supported by the key HMBC correlations from H_3-13 to C-14, and H_2-14 to C-4 and C-5, as well as the ^{1}H-^{1}H COSY correlations of H-6 with H-5 and H_2-7 (Figure S39 and S40). The stereochemistry of **8** was confirmed by NOESY correlations between H-13 and H-15, and between H-6 and H-5 (Figure S41). It was a new compound and named as ustidrimane B.

Ustidrimane C (**9**). Relative to **3**, compound **9** was assigned as the C-12 hydroxylated and C-14 oxidized derivative. The replacement of the C-12 methyl group by a hydroxymethyl group was evidenced by the disappearance of the C-12 methyl carbon (δ_C 19.4) and proton (δ_H 1.72) signals and the appearance of an oxygenated methylene carbon resonance at δ_C 61.2 with corresponding proton signals at δ_H 4.01 and 3.82. In addition, oxidation of the C-14 methyl group to a carboxyl group was supported by the loss of the C-14 methyl carbon (δ_C 33.4) and proton (δ_H 0.89) signals and the emergence of a carboxyl carbon resonance at δ_C 181.5 (Figure S42 and S43). These assignments were further supported by the key HMBC correlations from H_3-13 to C-14 and from H-12 to C-7, C-8, and C-9 (Figure S45). The configuration of **9** at C-4 was confirmed by NOESY correlation between H-13 and H-15. (Figure S47). It was a new compound and named as ustidrimane C.

Ustidrimane D **(10)**. Compared with **9**, compound **10** was assigned as the γ-lactone cyclized derivative. The conversion of the C-12 hydroxymethyl group into a γ-lactone carbonyl was evidenced by the disappearance of the C-12 oxymethylene carbon resonance (δ_C 61.2) and its corresponding proton signals (δ_H 4.01 and 3.82), together with the appearance of a lactone carbonyl resonance at δ_C 174.5. Consistently, the C-11 and H-11 resonances were shifted downfield from δ_C 55.4 to 68.2 and from δ_H 4.01/3.82 to 4.76/4.68 (Figure S48 and S49). The structural assignment was further corroborated by the absence of the HMBC correlations from H_2-12 to C-7, C-8, and C-9, consistent with γ-lactone formation (Figure S51). The correlation between H-15 and H-13 in the NOESY spectrum confirmed the configuration of **10** at C-4 (Figure S53). It was a new compound and named as ustidrimane D.

Ustidrimane E **(11)**. Relative to **10**, compound **11** was assigned as the C-14 reduced derivative. Reduction of the C-14 carboxyl group to a hydroxymethyl group was evidenced by the disappearance of the C-14 carboxyl carbon resonance (δ_C 183.6) and the appearance of an oxymethylene carbon resonance at δ_C 71.5 with corresponding proton signals at δ_H 3.49 and 3.19. The structural assignment was further supported by the key HMBC correlations from H_3-13 to C-14 and from H_2-14 to C-4 and C-5 (Figures S54–S56). Compound **11** was obtained in a low amount and its stereochemistry was tentatively assigned, based on the common biosynthetic origin, to be identical to those of the structurally related compounds **8–10** isolated from the Δ*driF* mutant. It was a new compound and named as ustidrimane E.

Ustidrimane F **(12)**. Compared with **11**, compound **12** was assigned as the C-6 acetylated derivative. The presence of an acetoxy group was evidenced by the appearance of an acetyl carbonyl resonance at δ_C 170.5 and the corresponding methyl proton signal at δ_H 2.05. In addition, the C-6 carbon and proton resonances were markedly shifted downfield from δ_C 17.6 to 66.5 and from δ_H 1.85/1.49 to 5.68, respectively, indicating acetylation at C-6 (Figures S57 and S58). The structural assignment was further supported by the key HMBC correlations from H_3-2′ to C-1′ and C-6 and from H-6 to C-2′, as well as the ^{1}H-^{1}H COSY correlations of H-6 with H-5 and H_2-7 (Figures S60 and S61). The configuration of **12** was confirmed by the NOESY correlations between H-13 and H-15 as well as H-6 and H-5 (Figure S62). It was a new compound and named as ustidrimane F.

Ustidrimane G **(13)**. Differing from **9**, compound **13** was assigned as the C-12 dehydroxylated derivative. Replacement of the C-12 hydroxymethyl group by a methyl group was evidenced by the disappearance of the C-12 oxymethylene carbon resonance (δ_C 61.2) and its corresponding proton signals (δ_H 4.01 and 3.82), together with the appearance of a methyl carbon resonance at δ_C 19.4 and its corresponding proton signal at δ_H 1.72 (Figure S63 and S64). The structural assignment was further supported by the key HMBC correlations from H_3-12 to C-7, C-8, and C-9 (Figure S66). The configuration of **13** at C-4 was confirmed by the NOESY correlation between H-15 and H-13 (Figure S68). It was a new compound and named as ustidrimane G.

Ustidrimane H **(14)**. Relative to **12**, compound **14** was assigned as the C-14 aldehyde derivative. Conversion of the C-14 hydroxymethyl group into an aldehyde was evidenced by the disappearance of the C-14 oxymethylene carbon resonance (δ_C 71.0) and its corresponding

proton signals (δ_H 3.66 and 3.20), together with the appearance of an aldehyde carbon resonance at δ_C 204.7 and the corresponding aldehydic proton signal at δ_H 9.34 (Figures S69 and S70). The structural assignment was further supported by the key HMBC correlations from H_3-13 to C-14 and from H-14 to C-4 (Figure S72). The NOESY correlations between H-13 and H-15 as well as between H-6 and H-5 proved the stereochemistry of **14** (Figure S74). It was a new compound and named as ustidrimane H.

In summary, clear NOESY correlations between H-13 and H-15 were observed in the spectra of compounds **8–10** and **12–14**, proving their same configuration at C-4 (4*R*). Compounds **8**, **12**, and **14** showed NOESY correlations between H-5 and H-6, indicating that they share the same relative configuration at C-6 (6*S*).

Ustidrimane I **(15)**. Compared with **16**, compound **15** exhibited cleavage of the γ-lactone ring. The loss of the γ-lactone carbonyl resonance at C-12 (δ_C 173.9), accompanied by the appearance of a methyl carbon resonance at δ_C 19.1 and its corresponding proton signal at δ_H 1.75, indicated conversion of the lactone carbonyl into a methyl group. Consistently, the C-13 and H-13 resonances were shifted upfield from δ_C 68.31 to 61.5 and from δ_H 4.41/4.14 to 3.53/3.29, respectively (Figures S75 and S76). The structure was further supported by the key HMBC correlations from H_3-12 to C-7, C-8, and C-9, as well as from H_2-11 to C-8, C-9, and C-10 (Figure S78). It was a new compound and named as ustidrimane I.

Ustidrimane J **(16)**. Compared with **19**, compound **16** exhibited cleavage of the *β*-lactone ring. The loss of the β-lactone carbonyl signal at C-14 (δ_C 173.9) and the appearance of an oxygenated methylene carbon resonance at δ_C 66.4 were indicative of *β*-lactone opening to generate two hydroxyl groups. Correspondingly, the C-13 carbon and proton resonances were shifted upfield from δ_C 68.31 to 61.6 and from δ_H 4.41/4.14 to 3.51/3.41, respectively (Figure S81 and S82). This assignment was further corroborated by the HMBC correlations from H_2-13 to C-14 and from H_2-14 to C-4 and C-5 (Figure S84). It was a new compound and named as ustidrimane J.

Ustidrimane K **(17)**. Compared with **19**, compound **17** showed cleavage of the γ-lactone ring. The lactone carbonyl carbon C-12 resonance at δ_C 173.7 disappeared and was replaced by a new sp^3 carbon signal at δ_C 19.6, accompanied by the appearance of a methyl proton signal at δ_H 1.75. Meanwhile, C-11 and H-11 shifted upfield from δ_C 68.29 to 58.1 and from δ_H 4.73/4.70 to 4.21/4.04, respectively (Figures S86 and S87). The HMBC correlations from H_3-12 to C-7, C-8, and C-9, together with the correlation from H_2-11 to C-8, C-9, and C-10, further confirmed the opened γ-lactone structure (Figure S89). It was a new compound and named as ustidrimane K.

Ustidrimane L **(18)**. In comparison with **19**, showed replacement of the γ-lactone moiety with a furan ring. The exocyclic methylene proton signals of H-11 (δ_H 4.73 and 4.70) were replaced by two olefinic proton signals at δ_H 7.15 and 7.11. Likewise, the oxygenated methylene carbon C-11 (δ_C 68.29) and lactone carbonyl carbon C-12 (δ_C 173.7) were replaced by two sp^2 carbons at δ_C 136.1 and 137.6, respectively (Figures S92 and S93). The HMBC correlations from H-11 to C-8, C-9, and C-12 confirmed the presence of the furan ring (Figure S95). It was a new

compound and named as ustidrimane L.

Ustidrimane M **(19)**. Compared with **1**, compound **19** lacked the acetyl group at C-6. The acetyl carbon signals at δ_C 169.4 (C-1′) and 21.4 (C-2′) were absent from the ^{13}C NMR spectrum, and the characteristic acetyl methyl singlet at δ_H 2.08 together with its corresponding HSQC correlation was no longer observed. Instead, C-6 resonated at δ_C 19.0 rather than δ_C 68.4 in **1**, while the H-6 signals appeared at δ_H 1.82 and 1.78 instead of δ_H 5.44, consistent with the loss of the acetoxy substituent (Figures S98–S100). This assignment was further supported by the ^1H-^1H COSY spectrum, which showed correlations between H_2-7 and H_2-6, as well as between H_2-6 and H-5 (Figure S102). Accordingly, compound **19** was identified as the derivative of **1**. It was a new compound and named as ustidrimane M.

Ustidrimane N **(20)**. Relative to **3**, compound **20** was assigned as the C-14 hydroxylated derivative. Replacement of the C-14 methyl group by a hydroxymethyl group was evidenced by the disappearance of the C-14 methyl carbon resonance (δ_C 33.4) and its corresponding proton signal (δ_H 0.89), together with the appearance of an oxymethylene carbon resonance at δ_C 72.1 and the corresponding proton signals at δ_H 3.44 and 3.17 (Figures S104-S105). The structural assignment was further supported by the key HMBC correlations from H_3-13 to C-14 and from H_2-14 to C-4 and C-5 (Figure S107). The configuration at C-4 was confirmed by the the NOESY correlation between H-15 and H-13 (Figure S109). It was a new compound and named as ustidrimane N.

Ustidrimane O **(21)**. Compared with **15**, compound **21** was identified as the C-14 aldehyde derivative. Oxidation of the C-14 hydroxymethyl group to an aldehyde was evidenced by the disappearance of the C-14 oxymethylene carbon resonance (δ_C 66.8) and its corresponding proton signals (δ_H 3.32 and 3.26), together with the appearance of an aldehyde carbon resonance at δ_C 208.2 and the corresponding aldehydic proton signal at δ_H 9.60 (Figures S110 and S111). The assignment was further corroborated by the key HMBC correlations from H-14 to C-4 and C-13 (Figure S113). It was a new compound and named as ustidrimane O.

Ustidrimane P **(22).** Compared with **21**, compound **22** was identified as the C-14 carboxylic acid derivative. Oxidation of the C-14 aldehyde to a carboxylic acid was evidenced by the disappearance of the aldehyde carbon resonance (δ_C 208.2) and its proton signal δ_H 9.60), together with the emergence of a carboxyl carbon resonance at δ_C 180.0 (Figures S115–S116). The assignment was further supported by the key HMBC correlations from H-13 to C-14 and C-13 (Figure S118). Accordingly, compound **22** was a new compound and named ustidrimane P.

Ustidrimane Q **(23)**. Relative to **17**, compound **23** was assigned as the C-12 hydroxylated derivative. Replacement of the C-12 methyl group by a hydroxymethyl group was evidenced by the disappearance of the C-12 methyl carbon resonance (δ_C 19.6) and its corresponding proton signal (δ_H 1.75), together with the appearance of an oxymethylene carbon resonance at δ_C 61.0 and the corresponding proton signals at δ_H 4.07 and 3.81 (Figures S120-S121). The structural assignment was further supported by the key HMBC correlations from H-12 to C-7, C-8, and C-9 (Figure S123). It was a new compound and named as ustidrimane Q.

12. Measurement of optical rotation

Optical rotations were measured on a Jasco DIP-370 polarimeter at 20 °C using the sodium D line (λ = 589.3 nm). Prior to measurement, the polarimeter was calibrated using 10% aqueous glucose solution and methanol as controls.

13. Experimental ECD

Experimental ECD spectra were recorded on a J-1500 CD spectrometer (Jasco Deutschland GmbH, Pfungstadt, Germany). Samples were dissolved in MeOH and measured over the range of 200–400 nm using a quartz cuvette with a 1 mm path length (Hellma Analytics, Müllheim, Germany).

14. Feeding experiments

For feeding experiments, the precursors were dissolved in DMSO to prepare 20 mM stock solutions. The fungal strains were first cultivated in 5 mL of LMM for 2 days at room temperature with shaking at 200 rpm. Subsequently, 5 µL of each stock solution was added to the 5 mL cultures to give a final concentration of 20 µM. After cultivation for an additional 3 days at room temperature with shaking at 200 rpm, 2 mL sample was taken and extracted with 2 mL of EtOAc and then dissolved in 50 µL of methanol for LC-MS analysis.

15. Physicochemical properties of the compounds described in this study

Ustidrimane A **(1)**. White powder. $[\alpha]^{20}_{D}$ –60 (*c* 0.50, MeOH). $C_{17}H_{20}O_6$, ESI-MS *m/z* 321.1336 $[M+H]^+$ (calcd for $C_{17}H_{21}O_6^+$: 321.1333). Spectral data are listed in Table S5 and NMR spectra in Figures S8–S13, respectively. ECD spectra are given in Figure S125.

Drim-8-ene-11-ol B **(3)**. White powder. $[\alpha]^{20}_{D}$ +15 (*c* 0.40, MeOH). $C_{15}H_{26}O$, ESI-MS *m/z* 205.1976 $[M–H_2O+H]^+$ (calcd for $C_{15}H_{25}^+$: 205.1951). Spectral data are listed in Table S6 and NMR spectra in Figures S14–S15, respectively. ECD spectra are given in Figure S125.

Confertifolin **(4)**. White powder. $[\alpha]^{20}$ +33 (*c* 0.60, MeOH). $C_{15}H_{22}O_2$, ESI-MS *m/z* 235.1703 $[M+H]^+$ (calcd for $C_{15}H_{23}O_2^+$: 235.1693. Spectral data are listed in Table S7 and NMR spectra in Figures S16–S18, respectively. ECD spectra are given in Figure S125.

6*β*-Hydroxyconfertifolin **(5)**. White powder. $[\alpha]^{20}_{D}$ +39 (*c* 0.70, MeOH). $C_{15}H_{22}O_3$, ESI-MS *m/z* 251.1655 $[M+H]^+$ (calcd for $C_{15}H_{23}O_3^+$: 251.1642). Spectral data are listed in Table S8 and NMR spectra in Figures S19–S24, respectively. ECD spectra are given in Figure S125.

6*β*-Acetoxyconfertifolin **(6)**. White powder. $[\alpha]^{20}_{D}$ +34 (*c* 0.70, MeOH). $C_{17}H_{24}O_4$, ESI-MS *m/z* 293.1770 $[M+Na]^+$ (calcd for $C_{17}H_{24}NaO_4^+$: 293.1747). Spectral data are listed in Table S9 and NMR spectra in Figures S25–S30, respectively. ECD spectra are given in Figure S125

3*β*-Hydroxyconfertifolin **(7)**. White powder. $[\alpha]^{20}_{D}$ +10 (*c* 0.50, MeOH). $C_{15}H_{22}O_3$, ESI-MS *m/z* 251.1656 $[M+H]^+$ (calcd for $C_{15}H_{23}O_3^+$: 251.1642). Spectral data are listed in Table S10 and NMR spectra in Figures S31–S35, respectively. ECD spectra are given in Figure S125.

Ustidrimane B **(8)**. White powder. $[\alpha]^{20}_{D}$ +73 (*c* 0.15, MeOH). $C_{15}H_{22}O_4$, ESI-MS *m/z* 267.1592 $[M+H]^+$ (calcd for $C_{15}H_{23}O_4^+$: 267.1591). Spectral data are listed in Table S11 and NMR spectra

in Figures S36–S41, respectively. ECD spectra are given in Figure S125.

Ustidrimane C (**9**). White powder. $[\alpha]^{20}_{D}$ +100 (*c* 0.01, MeOH). $C_{15}H_{24}O_4$, ESI-MS *m/z* 291.1564 $[M+Na]^+$ (calcd for $C_{15}H_{24}Na^+O_4$: 291.1567). Spectral data are listed in Table S12 and NMR spectra in Figures S42–S47, respectively. ECD spectra are given in Figure S125

Ustidrimane D (**10**). White powder. $[\alpha]^{20}_{D}$ +186 (*c* 0.07, MeOH). $C_{15}H_{20}O_4$, ESI-MS *m/z* 265.1449 $[M+H]^+$ (calcd for $C_{15}H_{21}O_4^+$: 265.1434). Spectral data are listed in Table S13 and NMR spectra in Figures S48–S53, respectively. ECD spectra are given in Figure S125.

Ustidrimane E (**11**). White powder. $[\alpha]^{20}_{D}$ +150 (*c* 0.02, MeOH). $C_{15}H_{22}O_3$, ESI-MS *m/z* 251.1646 $[M+H]^+$ (calcd for $C_{15}H_{23}O_3^+$: 251.1642). Spectral data are listed in Table S14 and NMR spectra in Figure S54–S56, respectively. ECD spectra are given in Figure S125.

Ustidrimane F (**12**). White powder. $[\alpha]^{20}_{D}$ –8 (*c* 0.50, MeOH). $C_{17}H_{24}O_5$, ESI-MS *m/z* 309.1712 $[M+H]^+$ (calcd for $C_{17}H_{25}O_5^+$: 309.1697). Spectral data are listed in Table S15 and NMR spectra in Figures S57–S62, respectively. ECD spectra are given in Figure S125.

Ustidrimane G (**13**). White powder. $[\alpha]^{20}_{D}$ +55 (*c* 0.04, MeOH). $C_{15}H_{24}O_3$, ESI-MS *m/z* 235.1709 $[M–H_2O+H]^+$ (calcd for $C_{15}H_{23}O_2^+$: 235.1704). Spectral data are listed in Table S16 and NMR spectra in Figures S63–S68, respectively. ECD spectra are given in Figure S125.

Ustidrimane H (**14**). White powder. $[\alpha]^{20}_{D}$ +12 (*c* 0.20, MeOH). $C_{17}H_{24}O_3$, ESI-MS *m/z* 329.1363 $[M+Na]^+$ (calcd for $C_{17}H_{24}NaO_3^+$: 329.1359). Spectral data are listed in Table S17 and NMR spectra in Figure S69–S74, respectively. ECD spectra are given in Figure S125.

Ustidrimane I (**15**). White powder. $[\alpha]^{20}_{D}$ +30 (*c* 0.10, MeOH). $C_{15}H_{26}O_3$, ESI-MS *m/z* 277.1785 $[M+Na]^+$ (calcd for $C_{15}H_{26}NaO_3^+$: 277.1774). Spectral data are listed in Table S18 and NMR spectra in Figures S75–S80, respectively. ECD spectra are given in Figure S125.

Ustidrimane J (**16**). White powder. $[\alpha]^{20}_{D}$ +76 (*c* 0.50, MeOH). $C_{15}H_{22}O_4$, ESI-MS *m/z* 267.1623 $[M+H]^+$ (calcd for $C_{15}H_{23}O_4^+$: 267.1591). Spectral data are listed in Table S19 and NMR spectra in Figures S81–S85, respectively. ECD spectra are given in Figure S125.

Ustidrimane K (**17**). White powder. $[\alpha]^{20}_{D}$ +122 (*c* 0.50, MeOH). $C_{15}H_{22}O_3$, ESI-MS *m/z* 273.1463 $[M+Na]^+$ (calcd for $C_{15}H_{22}NaO_3^+$: 273.1461). Spectral data are listed in Tables S20 and NMR spectra in Figure S86–S91, respectively. ECD spectra are given in Figure S125

Ustidrimane L (**18**). White powder. $[\alpha]^{20}_{D}$ +17 (*c* 0.60, MeOH). $C_{15}H_{18}O_3$, ESI-MS *m/z* 247.1335 $[M+H]^+$ (calcd for $C_{15}H_{19}O_3^+$: 247.1329). Spectral data are listed in Table S21 and NMR spectra in Figures S92–S97, respectively. ECD spectra are given in Figure S125.

Ustidrimane M (**19**). White powder. $[\alpha]^{20}_{D}$ -135 (*c* 0.20, MeOH). $C_{15}H_{18}O_4$, ESI-MS *m/z* 263.1291 $[M+H]^+$ (calcd for $C_{15}H_{19}O_4^+$: 263.1278). Spectral data are listed in Table S22 and NMR spectra in Figures S98–S103, respectively. ECD spectra are given in Figure S125.

Ustidrimane N (**20**). White powder. $[\alpha]^{20}_{D}$ +15 (*c* 0.40, MeOH). $C_{15}H_{26}O_2$, ESI-MS *m/z* 221.1908 $[M–H_2O+H]^+$ (calcd for $C_{15}H_{25}O^+$: 221.1900). Spectral data are listed in Table S23 and NMR spectra in Figures S104–S109, respectively. ECD spectra are given in Figure S125.

Ustidrimane O (**21**). White powder. $[\alpha]^{20}_{D}$ +2 (*c* 0.40, MeOH). $C_{15}H_{24}O_3$, ESI-MS *m/z* 275.1617 $[M+Na]^+$ (calcd for $C_{15}H_{24}NaO_3^+$: 275.1618). Spectral data are listed in Table S24 and NMR

spectra in Figures S110–S114, respectively. ECD spectra are given in Figure S125.

Ustidrimane P **(22)**. White powder. $[\alpha]^{20}_{D}$ +23 (*c* 0.30, MeOH). $C_{15}H_{24}O_4$, ESI-MS *m/z* 291.1568 $[M+Na]^+$ (calcd for $C_{15}H_{24}NaO_4^+$: 291.1567). Spectral data are listed in Table S25 and NMR spectra in Figures S115–S119, respectively. ECD spectra are given in Figure S125.

Ustidrimane Q **(23)**. White powder. $[\alpha]^{20}_{D}$ +5 (*c* 0.20, MeOH). $C_{15}H_{22}O_4$, ESI-MS *m/z* 289.1426 $[M+Na]^+$ (calcd for $C_{15}H_{22}NaO_4^+$: 289.1410). Spectral data are listed in Tables S26 and NMR spectra in Figures S120–S124, respectively. ECD spectra are given in Figure S125.

Supplementary Tables

Table S1. Sequence identities of proteins encoded by *dri* cluster (GenBank accession number: PZ445384) from *A.ustus* NRRL 280 with those of known clusters.

Protein	Size (aa)	homologous protein, organism	Coverage /identity (%)	Accession No.	Size (aa)	Putative function
DriA	485	Sesquiterpene cyclase AstC, *Aspergillus oryzae* RIB40	97/58	Q2UEK4.1	480	Sesquiterpene cyclase
DriB	487	*O*-acetyltransferase AndG, *Emericella variecolor* NBRC 32302	97/41	A0A097ZPE2.1	481	Acetylation
DriC	543	P450 monooxygenase AstF, *Aspergillus oryzae* RIB40	95/49	Q2UEK7.1	521	Hydroxylation
DriD	261	Short chain reductase AstE, *Aspergillus oryzae* RIB40	97/55	Q2UEK6.1	261	Aldehyde formation (lactone formation)
DriE	487	P450 monooxygenase AstJ, *Aspergillus oryzae* RIB40	95/63	Q2UEL1.1	500	Hydroxylation
DriF	497	P450 monooxygenase AstJ, *Aspergillus oryzae* RIB40	94/61	Q2UEL1.1	500	Hydroxylation
DriG	269	Chanoclavine-I dehydrogenase EasD, *Arthroderma benhamiae*	100/56	C5FTN0.1	264	Aldehyde formation
DriH	431	Retinaldehyde dehydrogenase 2, *Gallus gallus*	84/43	O93344.2	431	Aldehyde formation (lactone formation)
DriI	206	Sesquiterpene phosphatase AstI, *Aspergillus oryzae* RIB40	81/42	Q2UEL0.1	201	Dephosphorylation
DriJ	514	P450 monooxygenase AstD, *Aspergillus oryzae* RIB40	89/60	Q2UEK5.1	512	Hydroxylation
DriK	597	MFS-type transporter CdmB, *Talaromyces verruculosus*	100/57	A0A3G9H2R5.1	578	Transportation
DriL	209	Sesquiterpene phosphatase AstK, *Aspergillus oryzae* RIB40	97/55	P9WEM0.1	196	Dephosphorylation

Table S2. Strains used in this study.

Strain	Genotype	Source
E. coli DH5α	F⁻*endA1 glnV44 thi-1 recA1 gyrA96 deoR nupG* φ80dlacZΔM15 Δ(*lacZYA-argF*) U169, *hsdR17*(rK^{-} mK^{+}), λ^{-}	Thermo Scientific
E. coli XL10-Gold	Tet^{r} Δ(*mcrA*)*183* Δ(*mcrCB-hsdSMR-mrr*)173 *endA1 supE44 thi-1 recA1 gyrA96 relA1* lac Hte [F′ *proAB* lacIqZΔM15 Tn10 (Tet^{r}) Tn5 (Kan^{r}) Amy]	Agilent
Saccharomyces cerevisiae BJ5464-npgA	*MATα ura3-52 leu2-Δ1 trp1 his3-Δ200 pep4::HIS3 prb1-Δ1.6R can1 GAL npgA*	[17]
Aspergillus ustus NRRL 280	wild type	NRRL
Aspergillus nidulans LO8030	*pyroA4, riboB2, AfpyrG89, nkuA::argB*, deletions of nine major endogenous secondary metabolite clusters	[2]
A. nidulans BK06	*wA*-PKS::*gpdA*(p)-*Afribo* in LO8030	[18]
A. nidulans MW11	Δ*wA*::*driABCDEFGHIJKL-afribo* in LO8030	This study
A. nidulans MW12	Δ*driG*::*afpyro* in MW11	This study
A. nidulans MW13	Δ*driJ*::*afpyro* in MW11	This study
A. nidulans MW17	Δ*driE*::*afpyro* in MW11	This study
A. nidulans MW18	Δ*driF*::*afpyro* in MW11	This study
A. nidulans MW19	Δ*driC*::*afpyro* in MW11	This study
A. nidulans MW22	Δ*driD*::*afpyro* in MW11	This study
A. nidulans MW23	Δ*driH*::*afpyro* in MW11	This study
A. nidulans MW48	Δ*wA*::*gpdA*(p)*driC-afribo* in LO8030	This study
A. nidulans MW49	Δ*wA*::*gpdA*(p)*driD-afribo* in LO8030	This study
A. nidulans MW50	Δ*wA*::*gpdA*(p)*driJ-afribo* in LO8030	This study
A. nidulans MW56	Δ*wA*::*driAIKL-afribo* in LO8030	This study
A. nidulans MW57	Δ*wA*::*driAEIKL-afribo* in LO8030	This study
A. nidulans MW58	Δ*wA*::*driAEFIKL-afribo* in LO8030	This study
A. nidulans MW60	Δ*wA*::*gpdA*(p)*driDJ-afribo* in LO8030	This study
A. nidulans MW74	Δ*wA*::*driEFGH-afribo* in LO8030	This study
A. nidulans MW79	Δ*driE*::*pyrG* in MW11	This study
A. nidulans MW80	Δ*driF*::*pyrG* in MW11	This study
A. nidulans MW81	Δ*driG*::*pyrG* in MW11	This study
A. nidulans MW82	Δ*driH*::*pyrG* in MW11	This study
A. nidulans MW86	Δ*wA*::*driGH-afribo* in LO8030	This study
A. nidulans MW88	Δ*wA*::*driG-afribo* in LO8030	This study
A. nidulans MW90	Δ*wA*::*driH-afribo* in LO8030	This study

NRRL: ARS Culture Collection

Table S3. Plasmids used and constructed in this study.

Plasmid	Description	Source/Ref.
pJN017	*URA3*, *wA* flanking, *A. fumigatus riboB* (*afribo*), Amp^R, *gpdA*(p)	[6]
pYWB1	*URA3*, *wA* flanking, *A. fumigatus pyroA* (*afpyro*), Amp^R	[1]
pYWB2	*URA3*, *wA* flanking, *AfriboB*, Amp^R	[5]
pET28a(+)	Kan^R, T7(P), 6×His, MCS, T7 Terminator, *lacI*	Merck, Germany
pZL69	AMA1, URA3, *afpyrG*, Amp^R, *gpdA*(p)	[7]
pMW30	a 23,245 bp genomic fragment corresponding to nt 361–23,605 of PZ445384, including *driA–driL* and flanking regions, inserted into pYWB2	This study
pMW31	*driC* deletion plasmid containing amp^R, *URA3*, *afpyroA*, and 1507 bp 5′-flanking region of *driC* (nt 9950–8444 of PZ445384), and 1593 bp 3′-flanking region of *driC* (nt 6810–5218 of PZ445384)	This study
pMW32	*driD* deletion plasmid containing amp^R, *URA3*, *afpyroA*, and 1463 bp 5′-flanking region of *driD* (corresponding to nt 7486–8948 of PZ445384), and 1552 bp 3′-flanking region of *driD* (nt 9767–11,318 of PZ445384)	This study
pMW33	*driE* deletion plasmid containing amp^R, *URA3*, *afpyroA*, and 1541 bp 5′-flanking region of *driE* (corresponding to nt 13,209–11,669 of PZ445384), and 1542 bp 3′-flanking region of *driE* (nt 10,054–8513 of PZ445384)	This study
pMW34	*driF* deletion plasmid containing amp^R, *URA3*, *afpyroA*, and 1409 bp 5′-flanking region of *driF* (corresponding to nt 10,874–12,282 of PZ445384), and 1554 bp 3′-flanking region of *driF* (nt 13,935–15,488 of PZ445384)	This study
pMW35	*driG* deletion plasmid containing amp^R, *URA3*, *afpyroA*, and 1450 bp 5′-flanking region of *driG* (corresponding to nt 16,317–14,868 of PZ445384), and 1497 bp 3′-flanking region of *driG* (nt 13,996–12,500 of PZ445384)	This study
pMW36	*driJ* deletion plasmid containing amp^R, *URA3*, *afpyroA*, and 1469 bp 5′-flanking region of *driJ* (corresponding to nt 16,598–18,066 of PZ445384), and 1462 bp 3′-flanking region of *driJ* (nt 19,726–21,187 of PZ445384)	This study
pMW39	*driH* deletion plasmid containing amp^R, *URA3*, *afpyroA*, and 1462 bp 5′-flanking region of *driH* (corresponding to nt 14,067–15,528 of PZ445384), and 1534 bp 3′-flanking region of *driH* (nt 16,898–18,432 of PZ445384)	This study
pMW48	a 2018 bp genomic fragment corresponding to nt 8435–66418 of PZ445384, including *driC* and 3’-flanking region, inserted into pJN017	This study
pMW49	a 1081 bp genomic fragment corresponding to nt 8963–10,043 of PZ445384, including *driD* and 3’-flanking region, inserted into pJN017	This study

Table S3. Plasmids used and constructed in this study (Continued).

Plasmid	Description	Source/Ref.
pMW50	a 1987 bp genomic fragment corresponding to nt 18,063–20,049 of PZ445384, including *driJ* and 3'-flanking region, inserted into pJN017	This study
pMW51	an 810 bp fragment of *driG* from cDNA of *A. ustus* NRRL 280 inserted in pET28a(+)	This study
pMW56	a 2548 bp fragment containing *driA* and flanking regions corresponding to nt 361–2908 of PZ445384, a 1049 bp fragment containing *driI* and flanking regions corresponding to nt 16,908–17,956, and a 3138 bp fragment containing *driKL* and flanking regions corresponding to nt 20,468–23,605, inserted into pYWB2	This study
pMW57	a 2548 bp fragment containing *driA* and flanking regions corresponding to nt 361–2908 of PZ445384, a 2225 bp fragment containing *driE* and flanking regions corresponding to nt 9824–12,048, a 1049 bp fragment containing *driI* and flanking regions corresponding to nt 16,908–17,956, and a 3138 bp fragment containing *driKL* and flanking regions corresponding to nt 20,468–23,605, inserted into pYWB2	This study
pMW58	a 2548 bp fragment containing *driA* and flanking regions corresponding to nt 361–2908 of PZ445384, a 4112 bp fragment containing *driEF* and flanking regions corresponding to nt 9824–13,935, a 1049 bp fragment containing *driI* and flanking regions corresponding to nt 16,908–17,956, and a 3138 bp fragment containing *driKL* and flanking regions corresponding to nt 20,468–23,605, inserted into pYWB2	This study
pMW60	a 1044 bp fragment containing *driD* and 3'-flanking region corresponding to nt 8963–10,006 of PZ445384, and a 2293 bp fragment containing *driJ* and flanking regions corresponding to nt 17,746–20,038, inserted into pJN017	This study
pMW74	a 7095 bp genomic fragment containing *driEFGH* and flanking regions corresponding to nt 9824–16,918 of PZ445384 inserted into pYWB2	This study
pMW79	*driE* deletion plasmid containing amp^R, *URA3*, *pyrG*, and 1541 bp 5′-flanking region of *driE* (corresponding to nt 13,209–11,669 of PZ445384), and 1542 bp 3′-flanking region of *driE* (corresponding to nt 10,054–8513)	This study
pMW80	*driF* deletion plasmid containing amp^R, *URA3*, *pyrG*, and 1409 bp 5′-flanking region of *driF* (corresponding to nt 10,874–12,282 of PZ445384), and 1554 bp 3′-flanking region of *driF* (corresponding to nt 13,935–15,488)	This study
pMW81	*driG* deletion plasmid containing amp^R, *URA3*, *pyrG*, and 1450 bp 5′-flanking region of *driG* (corresponding to nt 16,317–14,868 of PZ445384), and 1497 bp 3′-flanking region of *driG* (corresponding to nt 13,996–12,500)	This study

Table S3. Plasmids used and constructed in this study (Continued).

Plasmid	Description	Source/Ref.
pMW82	*driH* deletion plasmid containing amp^R, *URA3*, *pyrG*, and 1462 bp 5′-flanking region of *driH* (corresponding to nt 14,067–15,528 of PZ445384), and 1534 bp 3′-flanking region of *driH* (corresponding to nt 16,898–18,432)	This study
pMW86	a 2993 bp fragment containing *driGH* and flanking regions corresponding to nt 13,926–16,918 of PZ445384 inserted into pYWB2	This study
pMW88	a 1604 bp fragment containing *driG* and flanking regions corresponding to nt 13,925–15,528 of PZ445384 inserted into pYWB2	This study
pMW90	a 2066 bp fragment containing *driH* and flanking regions corresponding to nt 14,868–16,933 of PZ445384 inserted into pYWB2	This study

Table S4. Primers designed in this study.

Primer	Sequence 5'→3'	Use
pMW30_1 F	ATTGCCTGATCAGCGGACTTGACTCTCCTTCTCCTGATCTGCCAAGAGTAGCCTATGCC	Amplification of the DNA region of *driA*-*driL* and its upstream and downstream from *A. ustus* NRRL 280 to construct pMW30
pMW30_1 R	TGACCAGATGCTTGAGCGTATC	
pMW30_2 F	TGACATTGCTCTGGCCTGAG	
pMW30_2 R	CCGTTGCAGTTGTCTCACTCC	
pMW30_3 F	CAGGAACCGCTCATTCAGTCC	
pMW30_3 R	GCCAGCAATCTGTTCAGTGG	
pMW30_4 F	CCACGAGACCGCATCTTCTTC	
pMW30_4 R	AGCCTCAACACCATATTTAATCCCATGTGGATCCCTTCTGGTCAAGCGATTCTGGTC	
MW11_ver_1 F	GTCTTCACCTGTCGCTCGATTC	Verification of transformants of strain MW11
MW11_ver_1 R	GGCTTACACAGAGGCCATGG	
MW11_ver_2 F	CAGGAACCGCTCATTCAGTCC	
MW11_ver_2 R	CGCAGATCTGGCCAGTATTG	
MW11_ver_3 F	GCCAGCAATCTGTTCAGTGG	
MW11_ver_3 R	GTCAAGCCGAGTGAGAAGAGC	
pMW31_driC_up_f	GAATTCGTAATCATGGTCATAGCTGTTTCCTGTGTTCAAGTCTTTCATCTCTCCGTCC	Amplification of the upstream region of *driC* from *A. ustus* NRRL 280 to construct pMW31
pMW31_driC_up_r	GGTAATATCAGATGGTCTCGAACTGACCTTACATTACACGGTGTTCTTGATAGTCCAGG	
pMW31_driC_down_f	ATAGACGTCAAGGTACACATCCTACGGAGTTACGATCGTGCTGATTCTTCTGGCTGTTC	Amplification of the downstream region of *driC* from *A. ustus* NRRL 280 to construct pMW31
pMW31_driC_down_r	CCTTTTGCTCACATGTTCTTTCCTGCGTTATCCCCTGCTGGTAGTGATGCCATCTCCTG	
pMW32_driD_up_f	TGATGAATTCGTAATCATGGTCATAGCTGTTTCCTGTGTTCGAACAACACGCCCACCAG	Amplification of the upstream region of *driD* from *A. ustus* NRRL 280 to construct pMW32
pMW32_driD_up_r	GTGGTAATATCAGATGGTCTCGAACTGACCTTACATTACTTGACGCGTGGGGGATGATG	

Table S4. Primers designed in this study (continued).

Primer	Sequence 5’→3’	Use
pMW32_driD_down_f	AGACGTCAAGGTACACATCCTACGGAGTTACGATCGGACGGATGAATTGATCGCAATAC	Amplification of the downstream region of *driD* from *A. ustus* NRRL 280 to construct pMW32
pMW32_driD_down_r	GGCCTTTTGCTCACATGTTCTTTCCTGCGTTATCCCCTGGAAGCGGCTTCTATCGCATG	
pMW33_driE_up_f	GATGAATTCGTAATCATGGTCATAGCTGTTTCCTGTGTCTGCTTGGATGATCAGGGTGG	Amplification of the upstream region of *driE* from *A. ustus* NRRL 280 to construct pMW33
pMW33_driE_up_r	AATATCAGATGGTCTCGAACTGACCTTACATTACGTGCTCACATCAGTTCCAGATCAAC	
pMW33_driE_down_f	AGACGTCAAGGTACACATCCTACGGAGTTACGATCCAACTTCAAGTCTATACAAGTGGG	Amplification of the downstream region of *driE* from *A. ustus* NRRL 280 to construct pMW33
pMW33_driE_down_r	CTTTTGCTCACATGTTCTTTCCTGCGTTATCCCCTGTGGTGATGACAATGACAATGAAG	
pMW34_driF_up_f	GTGATGAATTCGTAATCATGGTCATAGCTGTTTCCTGTGTCTCGTCGTTCTTGCTCAAG	Amplification of the upstream region of *driF* from *A. ustus* NRRL 280 to construct pMW34
pMW34_driF_up_r	GGTAATATCAGATGGTCTCGAACTGACCTTACATTACCATCTCTTCTGGTCTATCCTCG	
pMW34_driF_down_f	TAGACGTCAAGGTACACATCCTACGGAGTTACGATCGGTAGCTCGGAGTAATTTCAGAC	Amplification of the downstream region of *driF* from *A. ustus* NRRL 280 to construct pMW34
pMW34_driF_down_r	GCCTTTTGCTCACATGTTCTTTCCTGCGTTATCCCCTGGCCACTAGCTGATCATCAACG	
pMW35_driG_up_f	GATGAATTCGTAATCATGGTCATAGCTGTTTCCTGTGTCAGATCTGGCCAGTATTGAAC	Amplification of the upstream region of *driG* from *A. ustus* NRRL 280 to construct pMW35
pMW35_driG_up_r	TAATATCAGATGGTCTCGAACTGACCTTACATTACGTCATTGCTATGTAGTTTGAAGCG	
pMW35_driG_down_f	ATAGACGTCAAGGTACACATCCTACGGAGTTACGATCGTGCAGGAGTTCCATGAATACC	Amplification of the downstream region of *driG* from *A. ustus* NRRL 280 to construct pMW35
pMW35_driG_down_r	TGGCCTTTTGCTCACATGTTCTTTCCTGCGTTATCCCCTGCCTGCGTGAACTACACGAC	

Table S4. Primers designed in this study (continued).

Primer	Sequence 5'→3'	Use
pMW36_driJ_up_f	ATGAATTCGTAATCATGGTCATAGCTGTTTCCTGTGTTTCTACCGTGAGGAGATCTTCG	Amplification of the upstream region of *driJ* from *A. ustus* NRRL 280 to construct pMW36
pMW36_driJ_up_r	GTAATATCAGATGGTCTCGAACTGACCTTACATTACCCATTGTGAACGAGAGGATATGG	
pMW36_driJ_down_f	TAGACGTCAAGGTACACATCCTACGGAGTTACGATCGAATGACTCTGAGGCAGTAAAGG	Amplification of the downstream region of *driJ* from *A. ustus* NRRL 280 to construct pMW36
pMW36_driJ_down_r	CCTTTTGCTCACATGTTCTTTCCTGCGTTATCCCCTGGCATAGAGAGAAGCGACAATCC	
pMW39_driH_up_f	GATGAATTCGTAATCATGGTCATAGCTGTTTCCTGTGTGTCTTCAGCCTCGATGGCATC	Amplification of the upstream region of *driH* from *A. ustus* NRRL 280 to construct pMW39
pMW39_driH_up_r	GTAATATCAGATGGTCTCGAACTGACCTTACATTACCATGTCAACGTCTACCGTATCGG	
pMW39_driH_down_f	AGATAGACGTCAAGGTACACATCCTACGGAGTTACGATCTGCTGATCCTGAGTCTAGCC	Amplification of the downstream region of *driH* from *A. ustus* NRRL 280 to construct pMW39
pMW39_driH_down_r	CTTTTGCTCACATGTTCTTTCCTGCGTTATCCCCTGGAGATATACGAGAGTTCCGTCGG	
verif_up_r	CTACAACCGTCAGGAACAGCTG	Verification of 5F of Δ*driC*-Δ*driH* and Δ*driJ* mutant
verif_down_f	CTGAGACCACTACCCAGTTGAC	Verification of 3F of Δ*driC*-Δ*driH* and Δ*driJ* mutant
AmpURA_f	CAGGGGATAACGCAGGAAAG	Amp^R and *URA3* fragment to construct pMW31 - pMW36 and pMW39
AmpURA_r	ACACAGGAAACAGCTATGACCATG	
afpyro_f	GTAATGTAAGGTCAGTTCGAGACCATC	*afpyro* fragment to construct pMW31 - pMW36 and pMW39
afpyro_r	GATCGTAACTCCGTAGGATGTGTACC	
verif_deledriC_f	GCTCAAGCATCTGGTCAAGATCG	Verification of Δ*driC* mutant
verif_deledriC_r	GCATTCAGCTGTCCCTCATTG	

Table S4. Primers designed in this study (continued).

Primer	Sequence 5'→3'	Use
verif_deledriD_f	GACTGCCTCTACCACGAATGAG	Verification of Δ*driD* mutant
verif_deledriD_r	CGTCATGTAGGCTGAGTTGC	
verif_deledriE_f	GCCATACGGTATAGCTTCTCGAC	Verification of Δ*driE* and Δ*driJ*/Δ*driE* mutant
verif_deledriE_r	GAGCCATTGGTGATGAACTACG	
verif_deledriF_f	CATTACCTGGTGGTTATCAGG	Verification of Δ*driF* and Δ*driJ*/Δ*driF* mutant
verif_deledriF_r	CTACTCCTTCGCTGTAACC	
verif_deledriG_f	TCATGGAACTCCTGCACCAAC	Verification of Δ*driG* and Δ*driJ*/Δ*driG* mutant
verif_deledriG_r	ATGACCCGAGTATCAGGAGGC	
verif_deledriH_f	CGTCAAGCCGAGTGAGAAGAG	Verification of Δ*driH* and Δ*driJ*/Δ*driH* mutant
verif_deledriH_r	GTTAATGCCGACTGTCCCAGC	
verif_deledriJ_f	CCGACGGAACTCTCGTATATCTC	Verification of Δ*driJ* mutant
verif_deledriJ_r	CCATGCTGAACAGACTCCTCG	
driG_CDS_f	ATCATCACAGCAGCGGCCTGGTGCCGCGCGGCAGCCATATGACCCGAGTATCAGGAGGC	*driG* coding sequence amplified from cDNA to construct pMW51
driG_CDS_r	TCCGCGACCCATTTGCTGTCCACCAGTCATGCTAGCCATCATGGAACTCCTGCACCAAC	
pMW48 f	TGACTAACAGCTACCCCGCTTGAGCAGACATCACCATGGACGAGTCCAGTATCGTTTTC	Amplification of the gene *driC* from *A. ustus* NRRL 280 to construct pMW48
pMW48 r	AAATATCCCAGCCTCAACACCATATTTAATCCCATGTGTGACATTGCTCTGGCCTGAG	
pMW49 f	GCAGCTTGACTAACAGCTACCCCGCTTGAGCAGACATCACCATGACTGCCTCTACCACG	Amplification of the gene *driD* from *A. ustus* NRRL 280 to construct pMW49
pMW49 r	ATATCCCAGCCTCAACACCATATTTAATCCCATGTGCTATACAAGTGGGACTCGATTG	
pMW50 f	CGCAGCTTGACTAACAGCTACCCCGCTTGAGCAGACATCACCATGGGCGCACCTGCGAC	Amplification of the gene *driJ* from *A. ustus* NRRL 280 to construct pMW50
pMW50 r	TATCCCAGCCTCAACACCATATTTAATCCCATGTGGTCGACGGTAGGGATAATCTGCG	

Table S4. Primers designed in this study (continued).

Primer	Sequence 5'→3'	Use
pMW56_A_f	ATTGCCTGATCAGCGGACTTGACTCTCCTTCTCCTGATCTGCCAAGAGTAGCCTATGCC	Amplification of fragments *driA*, *driI* and *driKL* along with its upstream and downstream from *A. ustus* NRRL 280 to construct pMW56
pMW56_A_r	CATGTCAATAATTCTCTTACATACTCGGGCTAGACTCCTGTCTGGAAATCCTCCCTACC	
pMW56_I_f	CAGAAGTTTCGCGGGTAGGGAGGATTTCCAGACAGGAGTCTAGCCCGAGTATGTAAGAG	
pMW56_I_r	GATCTGGGCCGGGTCATTGTGAATCGCAGCTCACGATCGGTTGCTATGTGGTGCTACAG	
pMW56_KL_f	TGCTCTGAAAGGTGGGTACTGTAGCACCACATAGCAACCGATCGTGAGCTGCGATTCAC	
pMW56_KL_r	AGCCTCAACACCATATTTTAATCCCATGTGGGATCCCTTCTGGTCAAGCGATTCTGGTC	
pMW57_A_f	ATTGCCTGATCAGCGGACTTGACTCTCCTTCTCCTGATCTGCCAAGAGTAGCCTATGCC	Amplification of the genes *driA, driE,* and *driI*, *driKL* from *A. ustus* NRRL 280 to construct pMW57
pMW57_A_r	GTTGTTATCTTCGAAGACCAGCTGAAGCTTGTCCATCCTGTCTGGAAATCCTCCCTACC	
pMW57_E_f	TCGCCAGAAGTTTCGCGGGTAGGGAGGATTTCCAGACAGGATGGACAAGCTTCAGCTGG	
pMW57_E_r	GCCATGTCAATAATTCTCTTACATACTCGGGCTAGACTCCGCGCTCATTCCACAATACC	
pMW57_I_f	CTTTATGCATAAATAGGTATTGTGGAATGAGCGCGGAGTCTAGCCCGAGTATGTAAGAG	
pMW57_I_r	GATCTGGGCCGGGTCATTGTGAATCGCAGCTCACGATCGGTTGCTATGTGGTGCTACAG	
pMW57_KL_f	TGCTCTGAAAGGTGGGTACTGTAGCACCACATAGCAACCGATCGTGAGCTGCGATTCAC	
pMW57_KL_r	AGCCTCAACACCATATTTTAATCCCATGTGGGATCCCTTCTGGTCAAGCGATTCTGGTC	
pMW58_A_f	ATTGCCTGATCAGCGGACTTGACTCTCCTTCTCCTGATCTGCCAAGAGTAGCCTATGCC	Amplification of the genes *driA, driEF, driI* and *driKL* from *A. ustus* NRRL 280 to construct pMW58
pMW58_A_r	GTTGTTATCTTCGAAGACCAGCTGAAGCTTGTCCATCCTGTCTGGAAATCCTCCCTACC	
pMW58_EF_f	TCGCCAGAAGTTTCGCGGGTAGGGAGGATTTCCAGACAGGATGGACAAGCTTCAGCTGG	
pMW58_EF_r	TGCCATGTCAATAATTCTCTTACATACTCGGGCTAGACTCCTACTCCTTCGCTGTAACC	
pMW58_I_f	AACGGCCTCTTGCCCCGGTTACAGCGAAGGAGTAGGAGTCTAGCCCGAGTATGTAAGAG	
pMW58_I_r	GATCTGGGCCGGGTCATTGTGAATCGCAGCTCACGATCGGTTGCTATGTGGTGCTACAG	
pMW58_KL_f	TGCTCTGAAAGGTGGGTACTGTAGCACCACATAGCAACCGATCGTGAGCTGCGATTCAC	
pMW58_KL_r	AGCCTCAACACCATATTTTAATCCCATGTGGGATCCCTTCTGGTCAAGCGATTCTGGTC	

Table S4. Primers designed in this study (continued).

Primer	Sequence 5’→3’	Use
pMW60_1_f	CTTATTCCGTGGATACACATGTGCTTACTGGGAAGAGGCCGTACCTCTCTATTGTCAAG	Amplification of the genes *driD* and *driJ* from *A. ustus* NRRL 280 to construct pMW60
pMW60_1_r	TTCAGATTGTTTTGTATCTTGACAATAGAGAGGTACGGCCTCTTCCCAGTAAGCACATG	
pMW60_2_f	CCAGCCTCAACACCATATTTTAATCCCATGTGGGCGCCGGATAATCTGCGTTGTGATGG	
pMW60_2_r	GCAGCTTGACTAACAGCTACCCCGCTTGAGCAGACATCACCATGACTGCCTCTACCACG	
pMW74_f	ATTGCCTGATCAGCGGACTTGACTCTCCTTCTCCTGATCGATGGACAAGCTTCAGCTGG	Amplification of the fragment *driEFGH* from *A. ustus* NRRL 280 to construct pMW74
pMW74_r	AAATATCCCAGCCTCAACACCATATTTTAATCCCATGTGGGGCTAGACTCAGGATCAGC	
pyrG_f	GAGAGTTATTCTGTGTCTGACG	*pyrG* fragment to construct pMW79 - pMW82
pyrG_r	ATTCTGTCTGAGAGGAG	
pMW79_up_r	CACAACATATTTCGTCAGACACAGAATAACTCTCGTGCTCACATCAGTTCCAGATCAAC	Amplification of the upstream and downstream of *driE* from *A. ustus* NRRL 280 to construct pMW79
pMW79_down_f	GGCATCACGCATCAGTGCCTCCTCTCAGACAGAATCAACTTCAAGTCTATACAAGTGG G	
pMW80_up_r	ATACACAACATATTTCGTCAGACACAGAATAACTCTCCATCTCTTCTGGTCTATCCTCG	Amplification of the upstream and downstream of *driF* from *A. ustus* NRRL 280 to construct pMW80
pMW80_down_f	TGGCATCACGCATCAGTGCCTCCTCTCAGACAGAATGGTAGCTCGGAGTAATTTCAGAC	
pMW81_up_r	ACACAACATATTTCGTCAGACACAGAATAACTCTCGTCATTGCTATGTAGTTTGAAGCG	Amplification of the upstream and downstream of *driG* from *A. ustus* NRRL 280 to construct pMW81
pMW81_down_f	TTGGCATCACGCATCAGTGCCTCCTCTCAGACAGAATGTGCAGGAGTTCCATGAATACC	
pMW82_up_r	TACACAACATATTTCGTCAGACACAGAATAACTCTCCATGTCAACGTCTACCGTATCGG	Amplification of the upstream and downstream of *driH* from *A. ustus* NRRL 280 to construct pMW 82
pMW82_down_f	GCTTGGCATCACGCATCAGTGCCTCCTCTCAGACAGAATTGCTGATCCTGAGTCTAGCC	
verif _up_r	CGTGCCAATCTGGATGGAG	Verification of upstream of MW79 - MW82 double mutant

Table S4. Primers designed in this study (continued).

Primer	Sequence 5’→3’	Use
verif_down_f	CGATGAGCTCGTCCGTATTG	Verification of downstream of MW79 - MW82 double mutant
pMW86_f	TTGCCTGATCAGCGGACTTGACTCTCCTTCTCCTGATCGAAGGAGTAGGTAGCTCGGAG	Amplification of the fragment *driGH* from *A. ustus* NRRL 280 to construct pMW86
pMW86_r	AAATATCCCAGCCTCAACACCATATTTAATCCCATGTGGGGCTAGACTCAGGATCAGC	
pMW88_f	AATTGCCTGATCAGCGGACTTGACTCTCCTTCTCCTGATCCGAAGGAGTAGGTAGCTCG	Amplification of the fragment *driG* from *A. ustus* NRRL 280 to construct pMW88
pMW88_r	AATATCCCAGCCTCAACACCATATTTAATCCCATGTGCATGTCAACGTCTACCGTATC	
pMW90_f	CCTGATCAGCGGACTTGACTCTCCTTCTCCTGATCGTCATTGCTATGTAGTTTGAAGCG	Amplification of the fragment *driH* from *A. ustus* NRRL 280 to construct pMW 90
pMW90_r	ATATCCCAGCCTCAACACCATATTTAATCCCATGTGTTCTCTTACATACTCGGGCTAG	
MW48_verif_f	GCATTCAGCTGTCCCTCATTG	Verification of transformants of strain MW48
MW48_verif_r	TGACATTGCTCTGGCCTGAG	
MW49_verif_f1	GACTGCCTCTACCACGAATGAG	Verification of transformants of strain MW49
MW49_verif_r1	CGTCATGTAGGCTGAGTTGC	
MW49_verif_f2	CCATCCCTTATTCCTTTGAACC	
MW49_verif_r2	GGAGTAGATCGAGCTGCATTC	
MW50_verif_f	CCGACGGAACTCTCGTATATCTC	Verification of transformants of strain MW50
MW50_verif_r	CCATGCTGAACAGACTCCTCG	
MW56_verif_f1	TGCCAAGAGTAGCCTATGCC	Verification of transformants of strain MW56
MW56_verif_r1	ATGACAGTCATAGAACAGCGGC	
MW56_verif_f2	CCTCTTCCCAGTAAGCACATG	
MW56_verif_r2	GGAGTAGATCGAGCTGCATTC	
MW57_verif_f1	TGCCAAGAGTAGCCTATGCC	Verification of transformants of strain MW57
MW57_verif_r1	ATGACAGTCATAGAACAGCGGC	

Table S4. Primers designed in this study (continued).

Primer	Sequence 5'→3'	Use
MW57_verif_f2	GCCATACGGTATAGCTTCTCGAC	Verification of transformants of strain MW57
MW57_verif_r2	GAGCCATTGGTGATGAACTACG	
MW58_verif_f1	CATGCAGATTGGATTCGCAGC	Verification of transformants of strain MW58
MW58_verif_r1	CTACCCCTGGGTGGCAGTC	
MW58_verif_f2	ATGATATTCGATCCGGTTG	
MW58_verif_r2	CTACTCCTTCGCTGTAACC	
MW60_verif_f1	CCATCCCTTATTCCTTTGAACC	Verification of transformants of strain MW60
MW60_verif_r1	GGAGTAGATCGAGCTGCATTC	
MW60_verif_f2	GACTGCCTCTACCACGAATGAG	
MW60_verif_r2	GCCAGCAATCTGTTCAGTGG	
MW74_verif_f1	CCAACGTAACGACATCCGAG	Verification of transformants of strain MW74
MW74_verif_r1	CATGCAGATTGGATTCGCAGC	
MW74_verif_f2	CAGGAACCGCTCATTCAGTCC	
MW74_verif_r2	ATGACCCGAGTATCAGGAGGC	
MW86_verif_f	CAGGATCACTAGTGCCGTGC	Verification of transformants of strain MW86
MW86_verif_r	GTTAATGCCGACTGTCCCAGC	
MW88_verif_f	TCATGGAACTCCTGCACCAAC	Verification of transformants of strain MW88
MW88_verif_r	ATGACCCGAGTATCAGGAGGC	
MW90_verif_f	GACGTTGACATGGCTGTGAG	Verification of transformants of strain MW90
MW90_verif_r	GTTAATGCCGACTGTCCCAGC	

Table S5. NMR data of ustidrimane A (**1**) in $CDCl_3$.

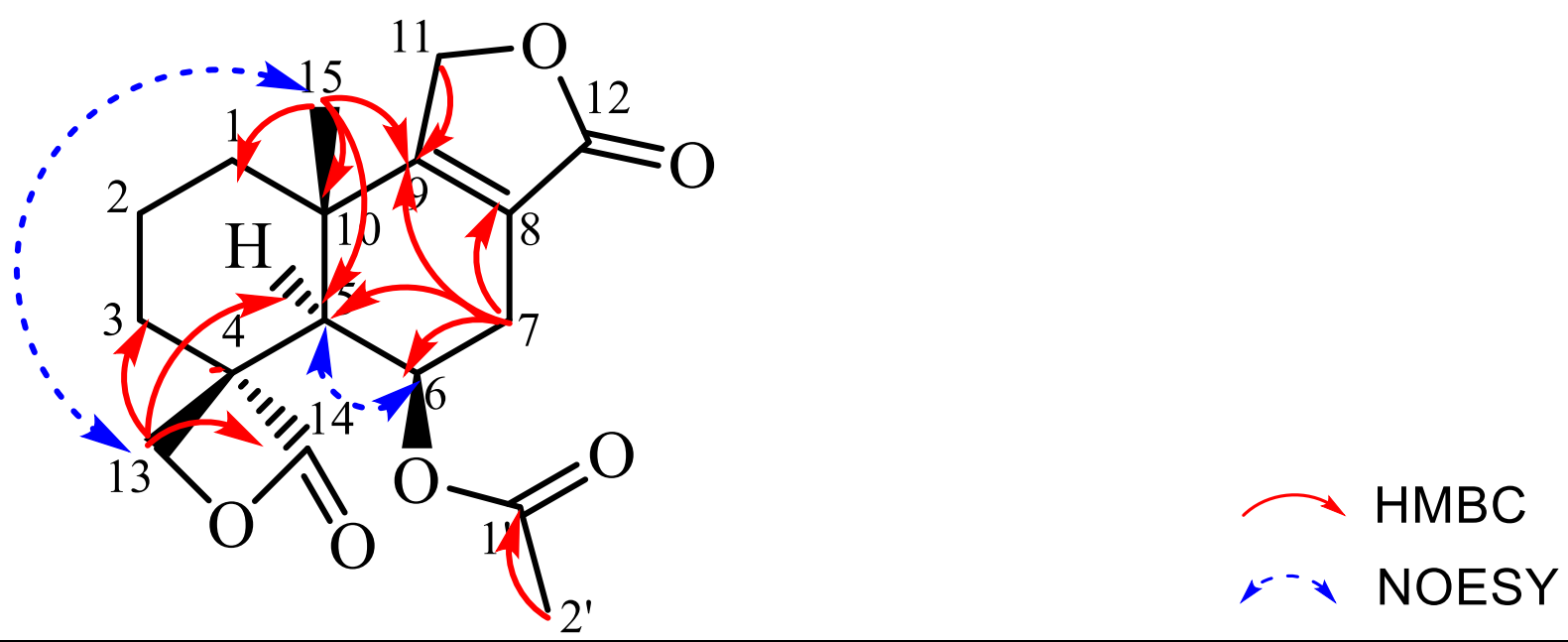

Position	δ_H (mult. *J* in Hz)	δ_C	Key HMBC	Key NOESY
1	1.73 (br d, 12.7)[a]	37.6		
	1.58 (td, 12.7, 4.1)			
2	1.87 (m)[b]	18.6		
	1.79 (qt, 13.8, 3.7)[a]			
3	2.15 (br d, 13.9)	34.1		
	1.93 (td, 13.5, 4.1)[b]			
4		60.6		
5	2.02 (d, 1.5)	45.3		H-6
6	5.44 (br s)	68.46		
7	2.62 (m)[c]	28.1	C-5, C-6, C-8, C-9	
	2.58 (m)[c]			
8		121.7		
9		166.1		
10		36.2		
11	4.82 (dt, 16.5, 2.7)	68.53		
	4.78 (dt, 16.5, 2.7)			
12		173.5		
13	4.70 (dd, 5.0, 1.5)	70.1	C-3, C-4, C-5, C-14	H-15
	4.22 (d, 5.0)			
14		173		
15	1.30 (s)	21.2	C-1, C-5, C-9, C-10	
1’		169.4		
2’	2.08 (s)	21.4	C-1’	

[a, b, c, d]: signals with the same letters overlapping with each other.

Table S6. NMR data of drim-8-ene-11-ol (**3**) in $CDCl_3$.

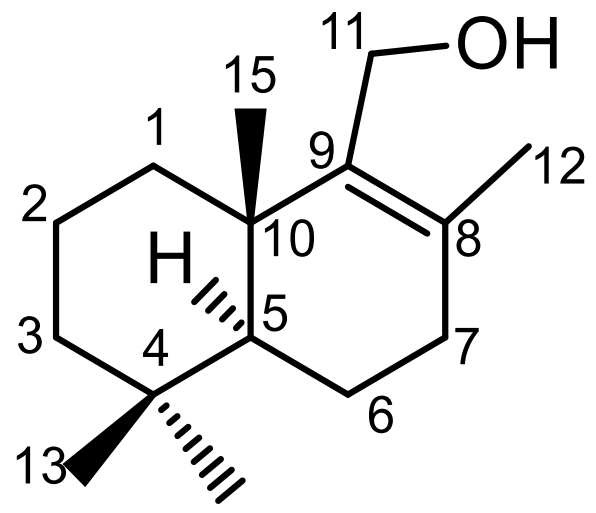

**Position**	***δ*H (mult. *J* in Hz)**	***δ*C**
1	1.60 (dt, 13.6, 3.2)	36.9
	1.20 (td, 13.2, 3.4)	
2	1.67 (m)	19.1
	1.50 (m)	
3	1.43 (m)	41.8
	1.12 (dt, 12.8, 3.4)[a]	
4		33.4
5	1.16 (dd, 12.6, 4.1)[a]	51.8
6	1.88 (br d, 12.7)	19.0
	1.40 (m)	
7	2.08 (m)[b]	33.8
	2.05 (m)[b]	
8		132.6
9		141.1
10		38.2
11	4.19 (d, 11.5)	58.4
	4.04 (d, 11.5)	
12	1.72 (br s)	19.4
13	0.84 (s)	21.7
14	0.89 (s)	33.4
15	0.96 (s)	20.8

[a, b]: signals with the same letters overlapping with each other.

The NMR data of **3** correspond well to those of drim-8-ene-11-ol.[11]

Table S7. NMR data of confertifolin (**4**) in $CDCl_3$.

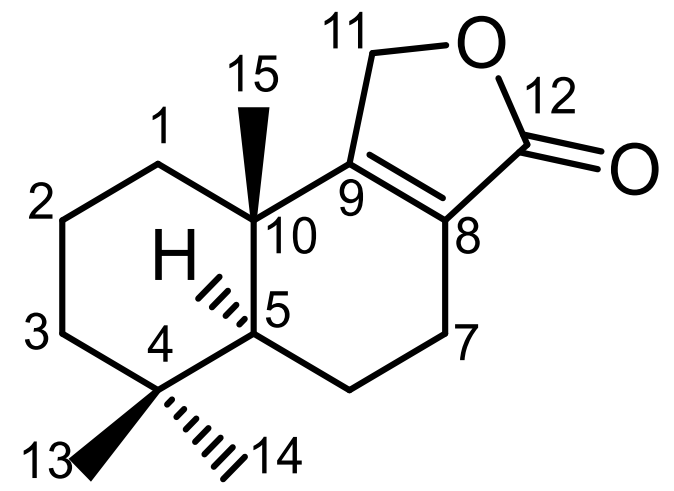

**Position**	**δ_H (mult. *J* in Hz)**	**δ_C**
1	1.64 (br d, 12.9)	36.3
	1.35 (td, 12.9, 3.8)	
2	1.72 (qt, 13.5, 3.2)	18.5
	1.57 (m)	
3	1.52 (m)	41.8
	1.23 (m)[a]	
4		33.4
5	1.26 (m)[a]	51.4
6	1.90 (br dd, 13.7, 7.2)	18.2
	1.49 (m)	
7	2.39 (m)	21.6
	2.14 (m)	
8		123.6
9		171.0
10		36.7
11	4.73 (dt, 16.9, 2.9)	68.4
	4.65 (ddd, 16.9, 3.1, 1.5)	
12		174.8
13	0.91 (s)	21.6
14	0.95 (s)	33.4
15	1.16 (s)	21.1

[a]: signals overlapping with each other.

The NMR data of **4** correspond well to those of confertifolin.[12,13]

Table S8. NMR data of 6*β*-hydroxyconfertifolin (**5**) in $CDCl_3$.

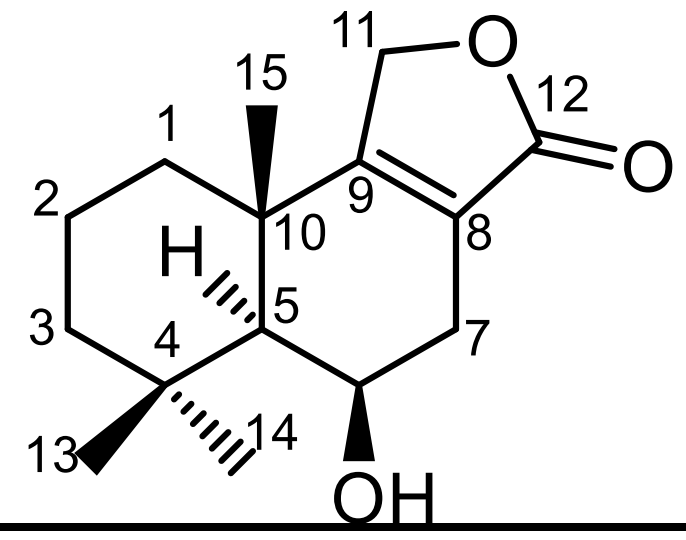

**Position**	***δ*$_H$ (mult. *J* in Hz)**	***δ*$_C$**
1	1.60 (m)[a]	39.5
	1.38 (td, 13.1, 4.0)	
2	1.81 (qt, 13.9, 3.7)	18.8
	1.57 (m)[a]	
3	1.48 (br d, 13.2)	43.2
	1.23 (m)[b]	
4		34.1
5	1.24 (m)[b]	54.3
6	4.70 (br s)[c]	65.1
7	2.46 (br d, 17.9)	32.9
	2.39 (br d, 17.9)	
8		120.7
9		170.2
10		36.8
11	4.82 (dt, 16.9, 2.6)	68.7
	4.71 (ddd, 16.9, 3.5, 1.5)[c]	
12		175
13	1.25 (s)	23.7
14	1.03 (s)	33.7
15	1.54 (s)	22.2

[a, b, c]: signals with the same letters overlapping with each other.

The NMR data of **5** correspond well to those of 6*β*-hydroxyconfertifolin.[14]

Table S9. NMR data of 6β-acetoxyconfertifolin (**6**) in $CDCl_3$.

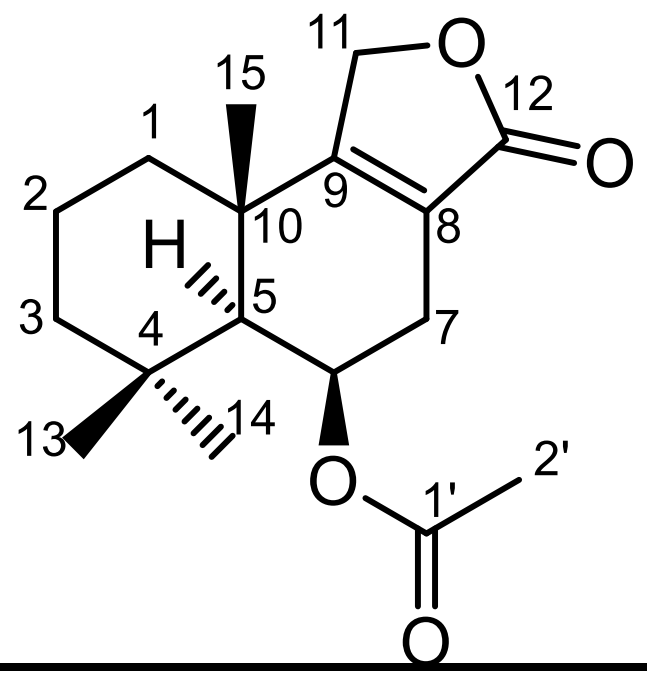

Position	δ_H (mult. *J* in Hz)	δ_C
1	1.67 (br s)	39.3
	1.42 (td, 13.0, 3.6)[a]	
2	1.82 (qt, 13.7, 3.3)	18.6
	1.62 (m)	
3	1.49 (m)	43.2
	1.26 (td, 13.5, 3.7)	
4		34.0
5	1.38 (br s)[a]	53.2
6	5.72 (d, 4.8)	66.5
7	2.50 (m)[b]	29.2
	2.45 (m)[b]	
8		120.6
9		169.7
10		37.0
11	4.83 (dt, 16.8, 2.6)	68.6
	4.74 (ddd, 16.8, 3.3, 1.7)	
12		174.3
13	1.04 (s)	33.5
14	1.05 (s)	23.0
15	1.52 (br s)	22.2
1’		170.3
2’	2.04 (s)	21.6

[a]: signals overlapping with each other.

The NMR data of **6** correspond well to those of 6β-acetoxyconfertifolin.[15]

Table S10. NMR data of 3β-hydroxyconfertifolin (**7**) in $CDCl_3$.

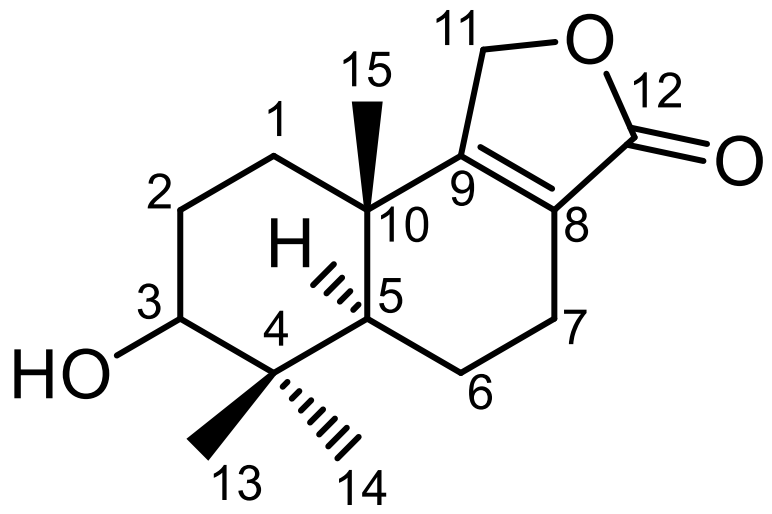

**Position**	δ_H **(mult.** ***J*** **in Hz)**	δ_C
1	1.70 (dt, 13.7, 3.4)[a]	34.2
	1.49 (td, 13.0, 4.1)	
2	1.80 (m)	27.2
	1.73 (m)[a]	
3	3.31 (dd, 11.1, 4.8)	78.5
4		39.1
5	1.21 (m)[a]	50.5
6	1.94 (br dd, 13.5, 7.1)	18.1
	1.59 (m)	
7	2.43 (m)	21.6
	2.15 (m)	
8		124
9		170
10		36.5
11	4.74 (dt, 16.9, 3.0)	68.3
	4.64 (ddd, 16.9, 3.6, 1.3)	
12		174.5
13	0.88 (s)	15.7
14	1.07 (s)	28.2
15	1.17 (s)	21.1

[a]: signals overlapping with each other.

The NMR data of **7** correspond well to those of 3β-hydroxyconfertifolin.[16]

Table S11. NMR data of ustidrimane B (**8**) in $CDCl_3$.

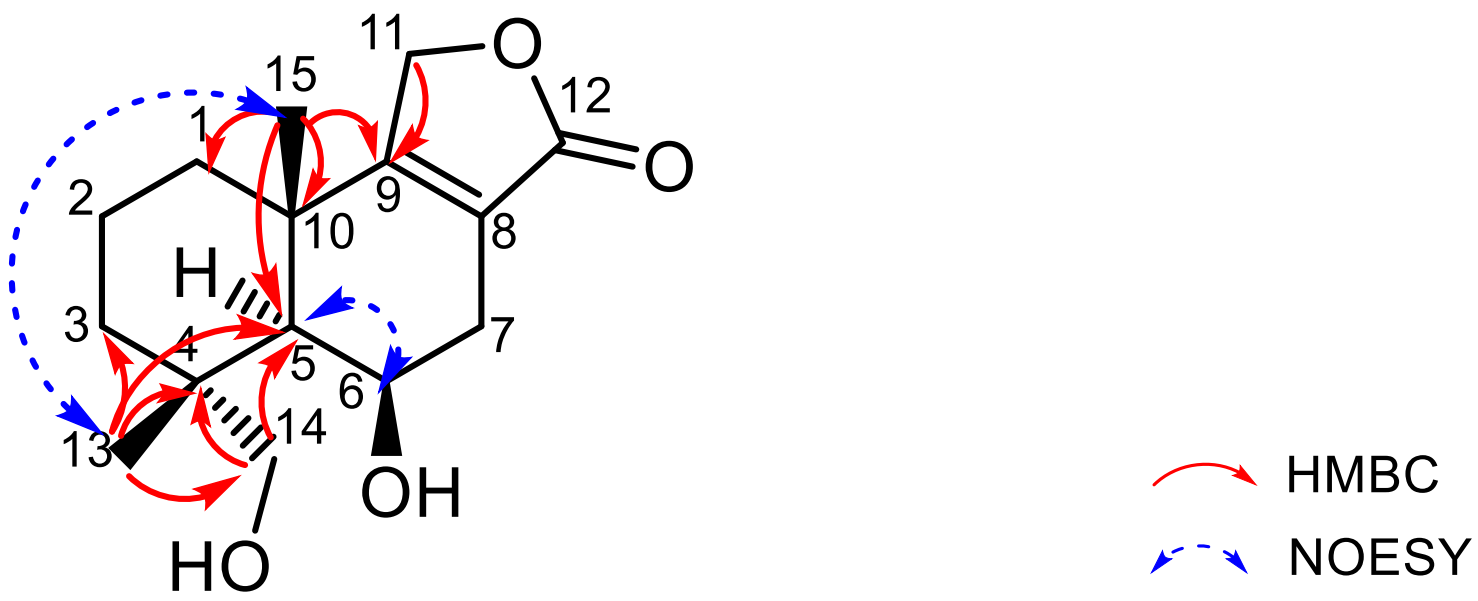

**Position**	δ_H **(mult.** ***J*** **in Hz)**	δ_C	**Key HMBC**	**Key NOESY**
1	1.58 (m)	38.9		
	1.39 (td, 12.8, 3.7)			
2	1.86 (qt, 13.5, 3.8)	18.2		
	1.67 (m)[a]			
3	1.53 (m)	36.6		
	1.28 (br d, 13.1)			
4		38.4		
5	1.66 (m)[a]	47.7		
6	4.69 (br s)	65.0		H-5
7	2.52 (br d,17.8)	32.6		
	2.35 (br d,17.8)			
8		120.7		
9		170.1		
10		36.61		
11	4.82 (dt, 16.8, 2.5)	68.8	C-9	
	4.73 (dd, 16.8, 3.2)			
12		175.0		
13	1.19 (s)	19.6	C-3, C-4, C-5, C-14	H-15
14	3.64 (d, 10.9)	71.9	C-4, C-5	
	3.23 (d, 10.9)			
15	1.55 (br s)	22.5	C-1, C-5, C-9, C-10	

[a]: signals overlapping with each other.

Table S12. NMR data of ustidrimane C (**9**) in DMSO-d_6.

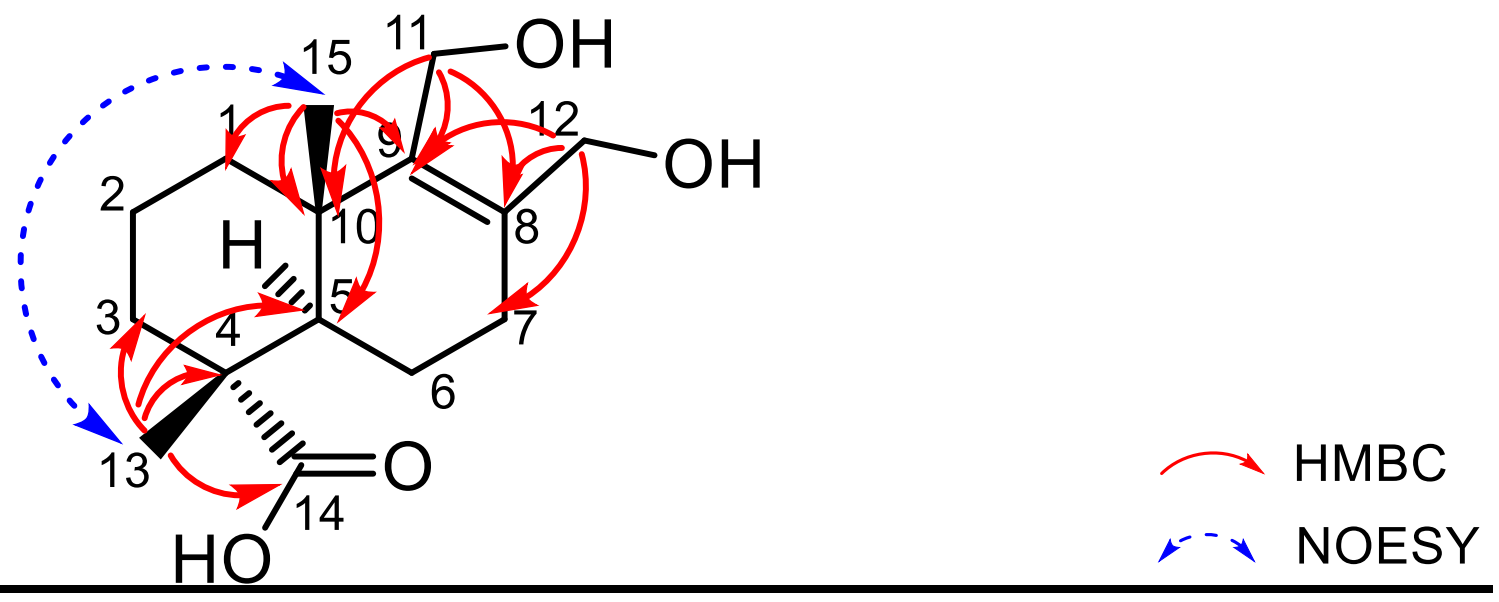

Position	δ_H (mult. *J* in Hz)	δ_C, type	Key HMBC	Key NOESY
1	1.83 (m)[a]	35.1		
	1.31 (td, 12.5, 4.1)[b]			
2	1.59 (m)[c]	17.8		
	1.52 (m)[d]			
3	1.64 (td, 13.1, 4.1)[c]	36.3		
	1.48 (m)[d,e]			
4		46.4		
5	1.86 (m)[a]	45.4		
6	1.44 (m)[e]	20.8		
	1.24 (m)[b]			
7	2.19 (dd, 18.1, 6.3)	28.4		
	2.03 (ddd,18.1, 10.4, 7.2)			
8		134.2		
9		142.4		
10		36.8		
11	4.01 (d, 12.5)[f]	55.4	C-8, C-9, C-10	
	3.86 (d, 12.5)			
12	4.01 (d, 12.5)[f]	61.2	C-7, C-8, C-9	
	3.82 (d, 12.5)			
13	1.07 (s)	16.4	C-3, C-4, C-5, C-14	H-15
14		181.5		
15	0.94 (s)	20.5	C-1, C-5, C-9, C-10	

[a, b, c, d, e, f]: signals with the same letters overlapping with each other.

Table S13. NMR data of ustidrimane D (**10**) in $CDCl_3$.

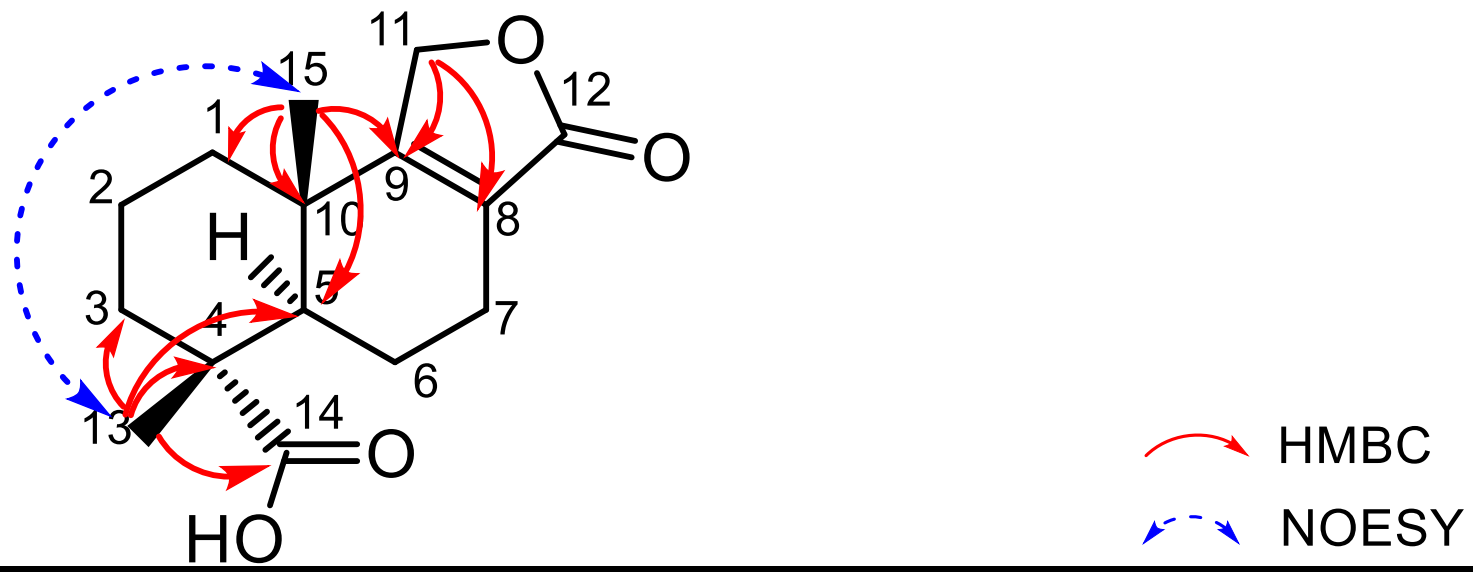

Position	δ_H (mult. *J* in Hz)	δ_C	Key HMBC	Key NOESY
1	1.72 (m)[a, b]	35.3		
	1.49 (td, 13.5, 3.5)			
2	1.78 (m)[c]	17.7		
	1.70 (m)[a, d]			
3	1.84 (td, 13.1, 4.3)	36.8		
	1.75 (m)[c, d]			
4		46.8		
5	2.13 (dd, 12.1, 2.2)	45.5		
6	1.68 (m)[b]	20.9		
	1.61 (m)			
7	2.38 (br d, 17.7)	21.3		
	2.24 (m)			
8		123.9		
9		169.8		
10		36.1		
11	4.76 (dt, 17.0, 2.8)	68.2	C-8, C-9	
	4.68 (ddd, 17.0, 3.6, 1.7)			
12		174.5		
13	1.27 (s)	16.2	C-3, C-4, C-5, C-14	
14		183.6		
15	1.21 (s)	21.3	C-1, C-5, C-9, C-10	H-13

[a, b, c, d]: signals with the same letters overlapping with each other.

Table S14. NMR data of ustidrimane E (**11**) in $CDCl_3$.

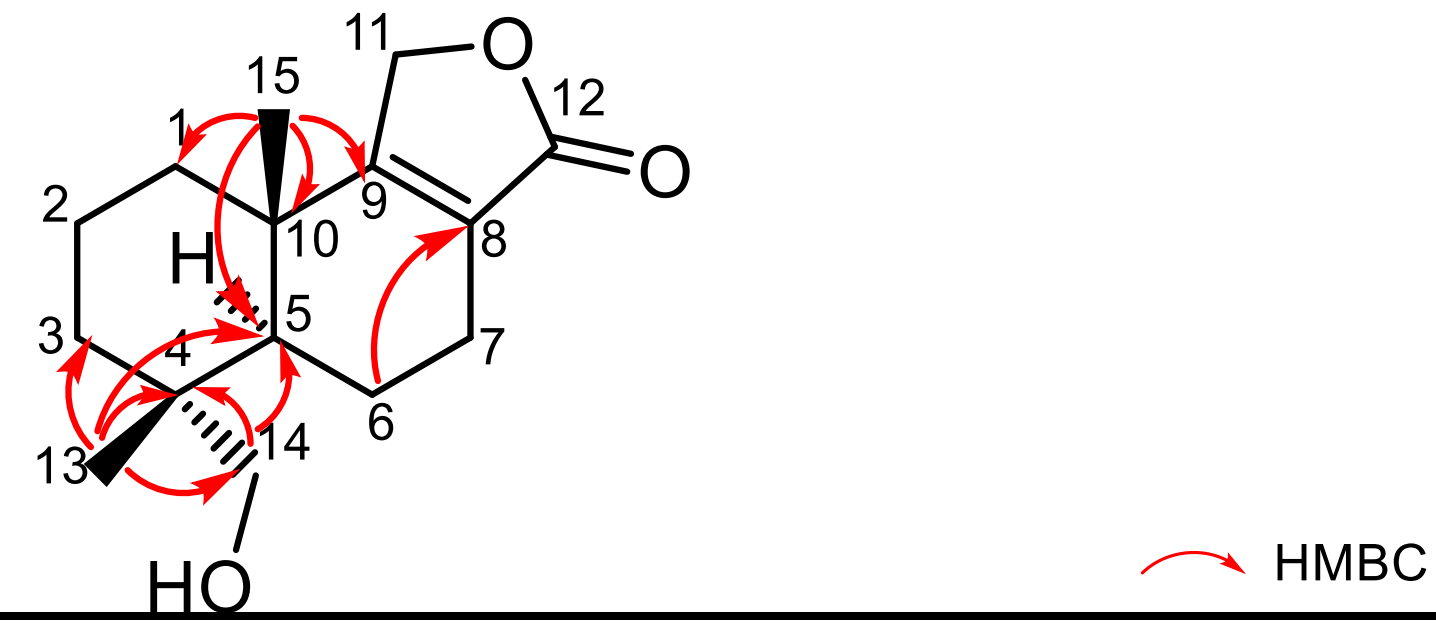

Position	δ_H (mult. *J* in Hz)	δ_C[a]	Key HMBC
1	1.67 (m)[b]	35.1	
	1.35 (m)[c]		
2	1.74 (m)	17.7	
	1.66 (m)[b,d]		
3	1.52 (m)[e]	37.6	
	1.35 (m)[c]		
4		34.9	
5	1.63 (m)[d]	44.3	
6	1.85 (br dd, 13.4, 7.1)	17.6	C-7
	1.49 (m)[e]		
7	2.38 (m)	21.1	
	2.19 (m)		
8		123.7	
9		170.7	
10		37.1	
11	4.75 (dt, 16.9, 2.8)	68.3	
	4.65 (ddd, 16.9, 3.6, 1.5)		
12			
13	0.86 (s)	17.1	C-3, C-4, C-5, C-14
14	3.49 (d, 10.8)	71.5	C-4, C-5
	3.19 (d, 10.8)		
15	1.20 (s)	21.3	C-1, C-5, C-9, C-10

a: The ^{13}C NMR data were deduced from the HSQC and HMBC spectra.

b, c, d, e: signals with the same letters overlapping with each other.

Table S15. NMR data of ustidrimane F (**12**) in $CDCl_3$.

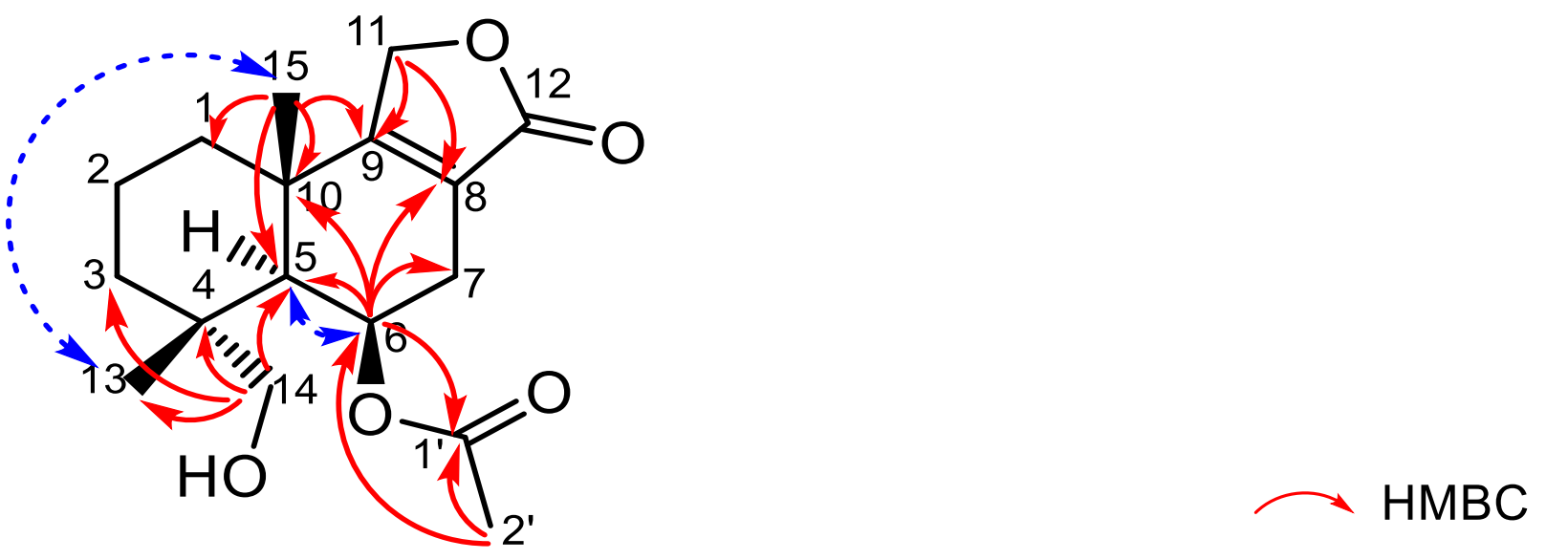

Position	δ_H (mult. *J* in Hz)	δ_C	Key HMBC	Key NOESY
1	1.64 (m)[a]	38.8		
	1.45 (td, 13.0, 3.9)			
2	1.84 (dt, 14.2, 4.4)	18.1		
	1.67 (m)[a, b]			
3	1.71 (m)[b]	36.5		
	1.30 (br d, 13.6)			
4		38.1		
5	1.91 (br s)	46.0		H-6
6	5.68 (br d, 5.2)	66.5	C-5, C-7, C-8, C-10, C-1’	
7	2.55 (m)	29.0		
	2.44 (br d, 18.1)			
8		120.7		
9		169.7		
10		36.7		
11	4.85 (dt, 16.9, 2.6)	68.7	C-8, C-9	
	4.76 (ddd, 16.8, 3.4, 1.5)			
12		174.4		
13	0.97 (s)	19.1		H-15
14	3.66 (d, 10.8)	71.0	C-3, C-4, C-5, C-13	
	3.20 (d, 10.8)			
15	1.55 (br s)	22.5	C-1, C-5, C-9, C-10	
1’		170.5		
2’	2.05 (s)	21.7	C-1’, C-6	

[a, b]: signals with the same letters overlapping with each other.

Table S16. NMR data of ustidrimane G (**13**) in $CDCl_3$.

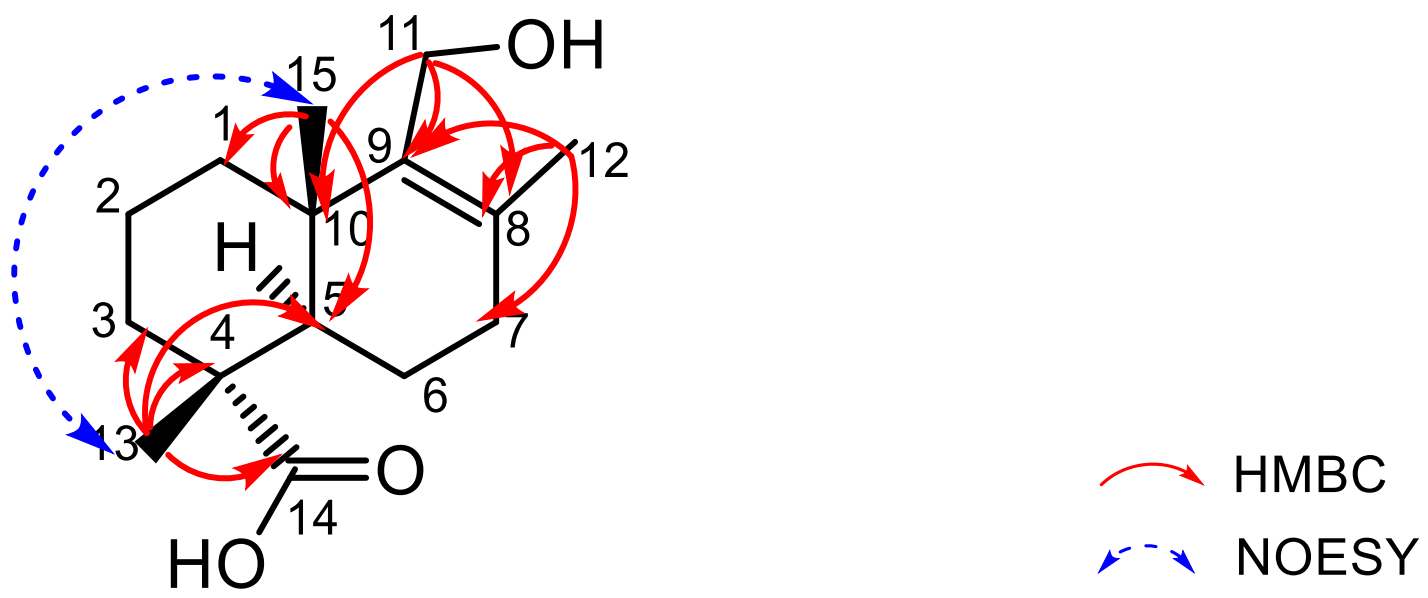

Position	δ_H (mult. *J* in Hz)	δ_C	Key HMBC	Key NOESY
1	1.92 (br d, 12.9)[a]	35.9		
	1.39 (td, 12.9, 3.6)			
2	1.66 – 1.62 (m)[b, c]	18.2		
3	1.78 (m)	36.7		
	1.68 (m)[b]			
4		47.4		
5	1.99 (m)[d]	45.9		
6	1.59 (m)[c]	21.6		
	1.32 (m)[a]			
7	2.17 (ddd, 18.5, 11.3, 7.3)	33.1		
	2.03 (m)[d]			
8		132.9		
9		140.3		
10		37.4		
11	4.21 (d, 11.6)	58.2	C-8, C-9, C-10	
	4.05 (d, 11.6)			
12	1.72 (s)	19.4	C-7, C-8, C-9	
13	1.19 (s)	16.2	C-3, C-4, C-5, C-14	
14		184.6		
15	0.99 (s)	20.9	C-1, C-5, C-9, C-10	H-13

[a, b, c, d]: signals with the same letters overlapping with each other.

Table S17. NMR data of ustidrimane H (**14**) in $CDCl_3$.

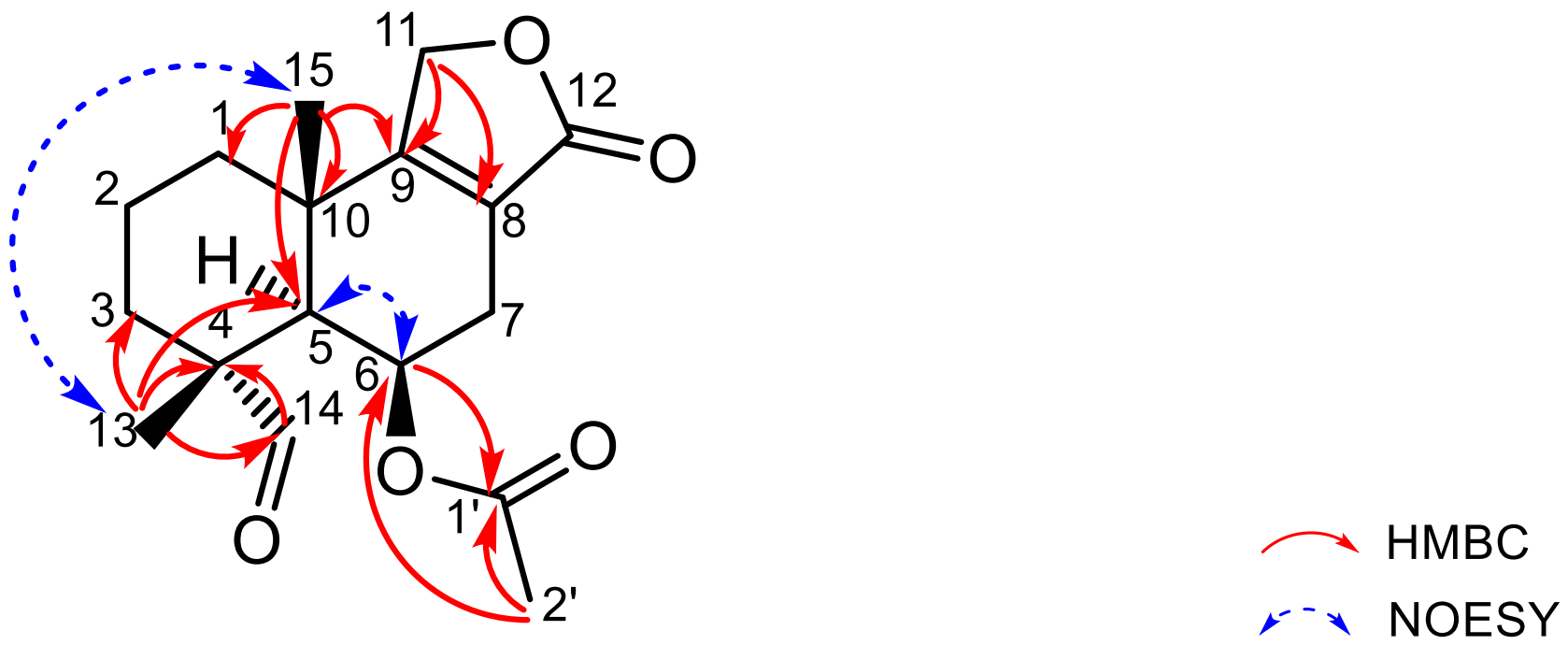

Position	δ_H (mult. *J* in Hz)	δ_C	Key HMBC	Key NOESY
1	1.72 (m)[a]	38.3		
	1.50 (m)[b]			
2	1.88 (qt, 13.5, 4.2)	17.2		
	1.78 (m)[a]			
3	1.46 (m)[b, c]	34.2		
	1.41 (td, 13.3, 4.4)[c]			
4		49.0		
5	2.05 (br s)	45.6		
6	5.23 (dt, 5.0, 1.3)	68.5	C-1'	H-5
7	2.57 (m)	28.9		
	2.47 (br d, 18.8)			
8		121.1		
9		168.4		
10		36.1		
11	4.85 (dt, 16.9, 2.7)	68.4	C-8, C-9	
	4.77 (ddd, 16.9, 3.5, 1.6)			
12		174.1		
13	1.34 (s)	16.3	C-3, C-4, C-5, C-14	H-15
14	9.34 (s)	204.7	C-4	
15	1.57 (br s)	22.6	C-1, C-5, C-9, C-10	
1'		169.9		
2'	2.03 (s)	21.5	C-1', C-6	

[a, b, c]: signals with the same letters overlapping with each other.

Table S18. NMR data of ustidrimane I (**15**) in DMSO-d_6.

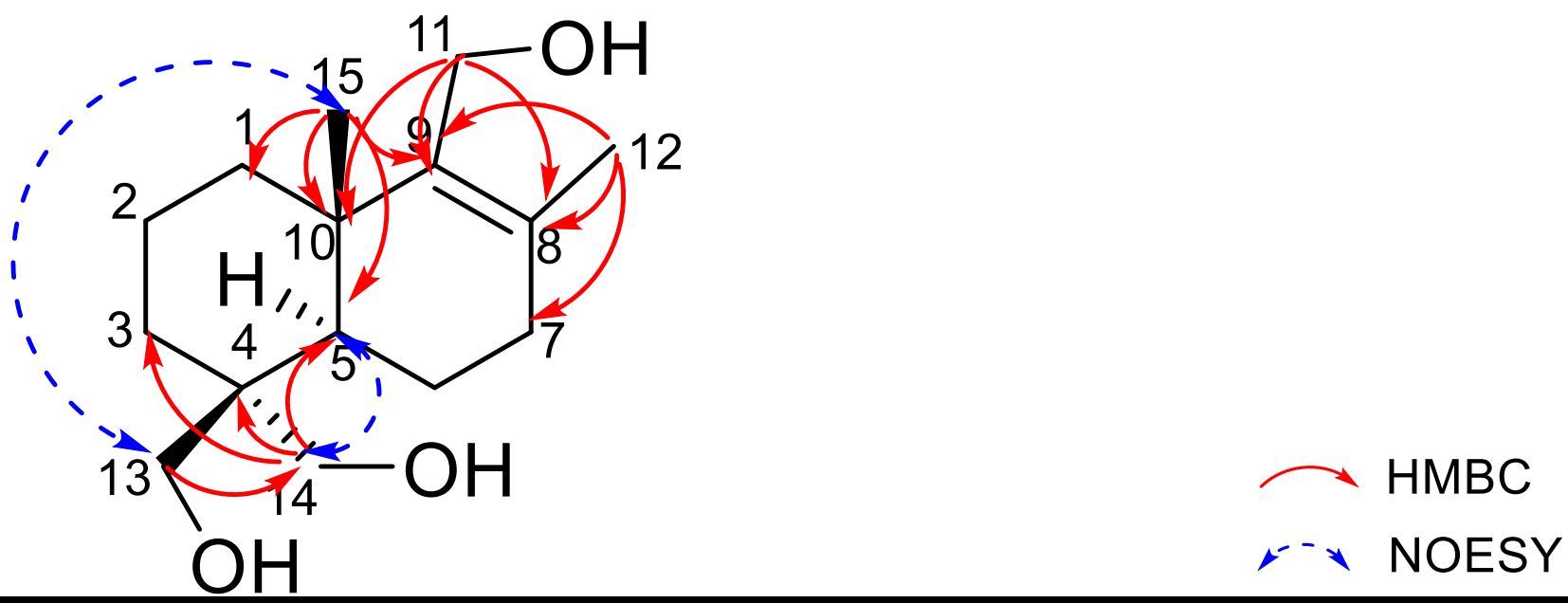

Position	δ_H (mult. *J* in Hz)	δ_C	Key HMBC	Key NOESY
1	1.76 (br d, 13.7)	35.8		
	1.18 (td, 13.0, 4.0)			
2	1.51 (m)[a]	17.9		
	1.39 (m)[b]			
3	1.50 (m)[a]	28.8		
	1.10 (td,13.9, 4.3)			
4		41.9		
5	1.46 (d, 1.6)	44.4		
6	1.67 (br dd, 12.7, 7.0)	18.5		
	1.31 (ddd, 12.2, 5.8, 1.1)[b]			
7	1.93 (ddd, 18.1, 11.3, 7.0)	33.3		
	1.82 (dd, 18.1, 6.2)			
8		129.3		
9		140.8		
10		37.1		
11	3.91 (d, 11.4)	56.2	C-8, C-9, C-10	
	3.80 (d, 11.4)			
12	1.56 (br s)	19.1	C-7, C-8, C-9	
13	3.53 (d, 10.9)	61.5	C-14	H-15
	3.29 (d, 10.9)[c]			
14	3.32 (d, 10.8)	66.8	C-3, C-4, C-5	H-5
	3.26 (d, 10.8)[c]			
15	0.87 (s)	21.0	C-1, C-5, C-9, C-10	

[a, b, c]: signals with the same letters overlapping with each other.

Table S19. NMR data of ustidrimane J (**16**) in DMSO-d_6.

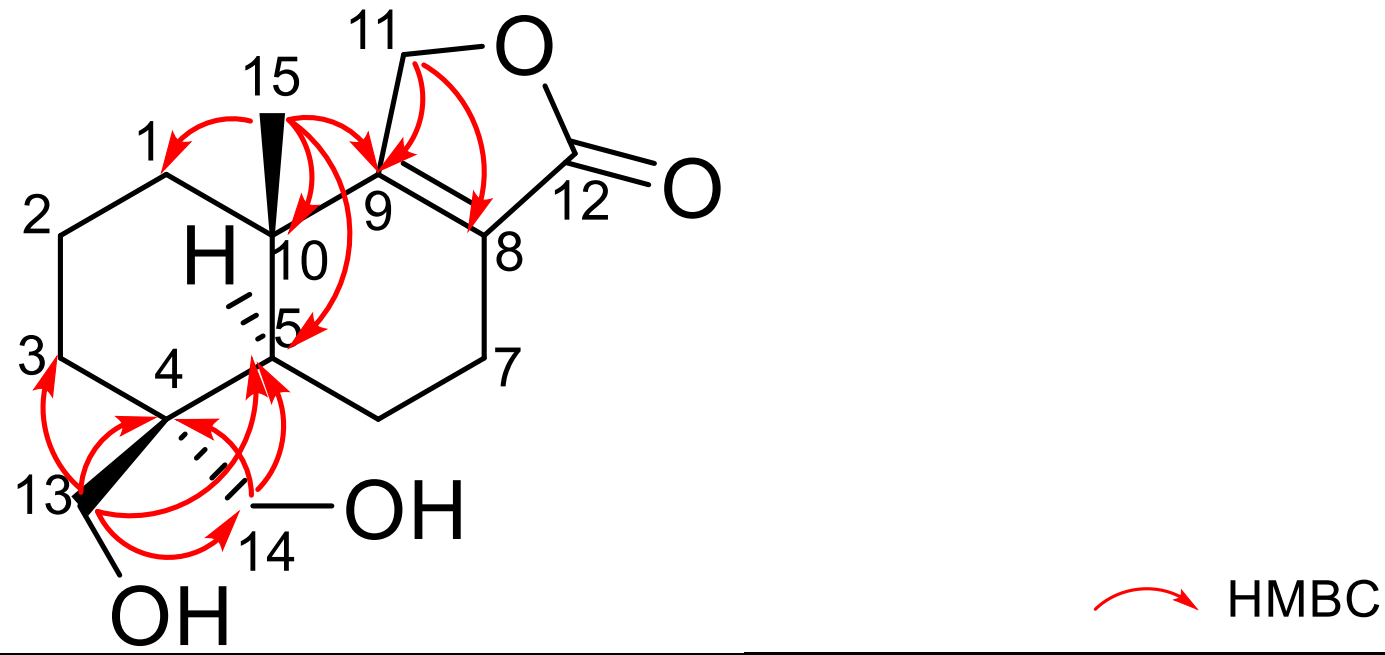

Position	δ_H (mult. *J* in Hz)	δ_C	Key HMBC
1	1.59 (m)[a, b]	35.2	
	1.31 (td, 13.1, 3.9)[c]		
2	1.68 (m)[d]	17.4	
	1.47 (m)[e]		
3	1.55 (m)[a, f]	29.1	
	1.23 (td, 13.3, 4.3)[c]		
4		42.0	
5	1.62 (m)[b, d]	43.9	
6	1.92 (m)[g]	17.7	
	1.51 (m)[e, f]		
7	2.14 (m)	21.1	
	1.98 (m)[g]		
8		121.7	
9		171.9	
10		35.8	
11	4.89 (dt, 17.5, 2.7)	68.3	C-8, C-9
	4.74 (br d, 17.5)		
12		173.9	
13	3.54 (d, 11.1)	61.6	C-3, C-4, C-5, C-14
	3.41 (d, 11.1)[h]		
14	3.44 (d, 11.0)[h]	66.4	C-4, C-5
	3.24 (d, 11.0)		
15	1.13 (s)	21.0	C-1, C-5, C-9, C-10

[a, b, c, d, e, f, g, h]: signals with the same letters overlapping with each other.

Table S20. NMR data of ustidrimane K (**17**) in $CDCl_3$.

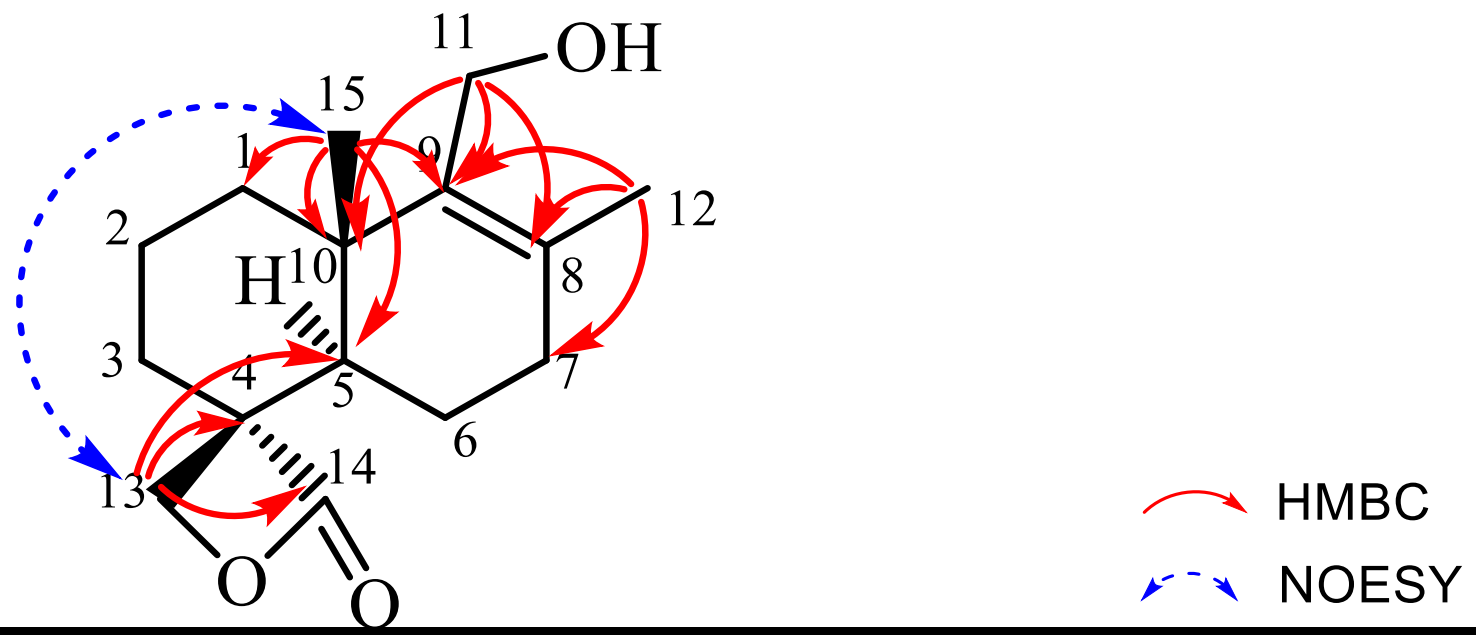

Position	δ_H (mult. *J* in Hz)	δ_C	Key HMBC	Key NOESY
1	1.96 (dt, 13.3, 3.8)	35.5		
	1.34 (td, 13.3, 3.9)			
2	1.57 (m)[a, b]	18.9		
	1.55 (m)[a]			
3	2.05 (m)[c]	32.6		
	1.82 (br td, 13.7, 4.5)			
4		62.1		
5	1.75 (br s)[d]	43.3		
6	1.69 (ddd, 12.2, 5.8, 1.5)	20.7		
	1.60 (m)[b]			
7	2.24 (ddd, 18.0, 10.8, 7.1)	32.4		
	2.09 (m)[c]			
8		133.7		
9		138.3		
10		37.2		
11	4.21 (d, 11.6)	58.1	C-8, C-9, C-10	
	4.04 (d, 11.6)			
12	1.75 (br s)[d]	19.6	C-7, C-8, C-9	
13	4.38 (dd, 5.2, 1.3)	68.9	C-3, C-4, C-5, C-14	H-15
	4.09 (d, 5.2)			
14		175.3		
15	0.83 (s)	19.5	C-1, C-5, C-9, C-10	

[a, b, c, d]: signals with the same letters overlapping with each other.

Table S21. NMR data of ustidrimane L (**18**) in $CDCl_3$.

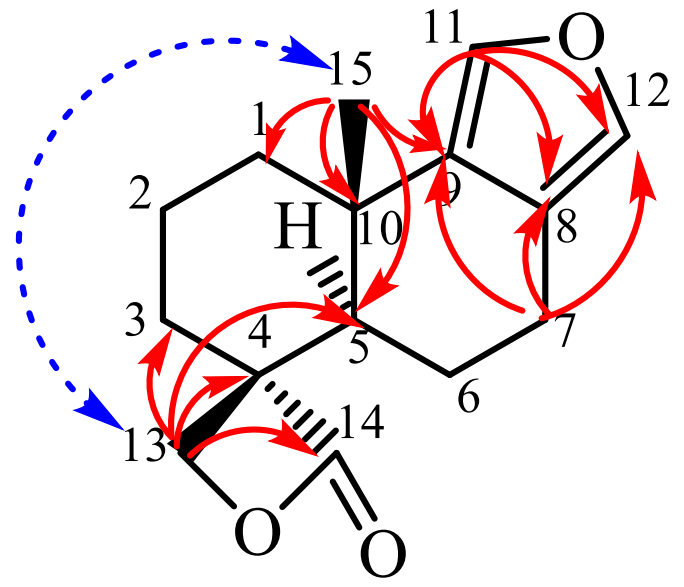

Position	δ_H (mult. *J* in Hz)	δ_C, type	Key HMBC	Key NOESY
1	1.97 (m)[a]	38.1		
	1.60 (m)[b]			
2	1.75 (m)[c]	19.0		
	1.65 (m)[b]			
3	2.11 (m)	32.7		
	1.92 (m)[a, d]			
4		61.6		
5	1.88 (m)[d, e]	42.8		
6	1.85 (dd, 11.2, 5.0)[e]	21.2		
	1.70 (m)[c]			
7	2.81 (dd, 16.0, 5.1)	19.8	C-8, C-9, C-12	
	2.61 (m)			
8		119.2		
9		133.5		
10		33.1		
11	7.15 (d, 1.3)	136.1	C-8, C-9, C-12	
12	7.11 (q, 1.4)	137.6		
13	4.41 (dd, 5.3, 1.4)	68.8	C-3, C-4, C-5, C-14	H-15
	4.14 (d, 5.3)			
14		175.0		
15	1.03 (s)	23.7	C-1, C-5, C-9, C-10	

[a, b, c, d, e]: signals with the same letters overlapping with each other.

Table S22. NMR data of ustidrimane M (**19**) in $CDCl_3$.

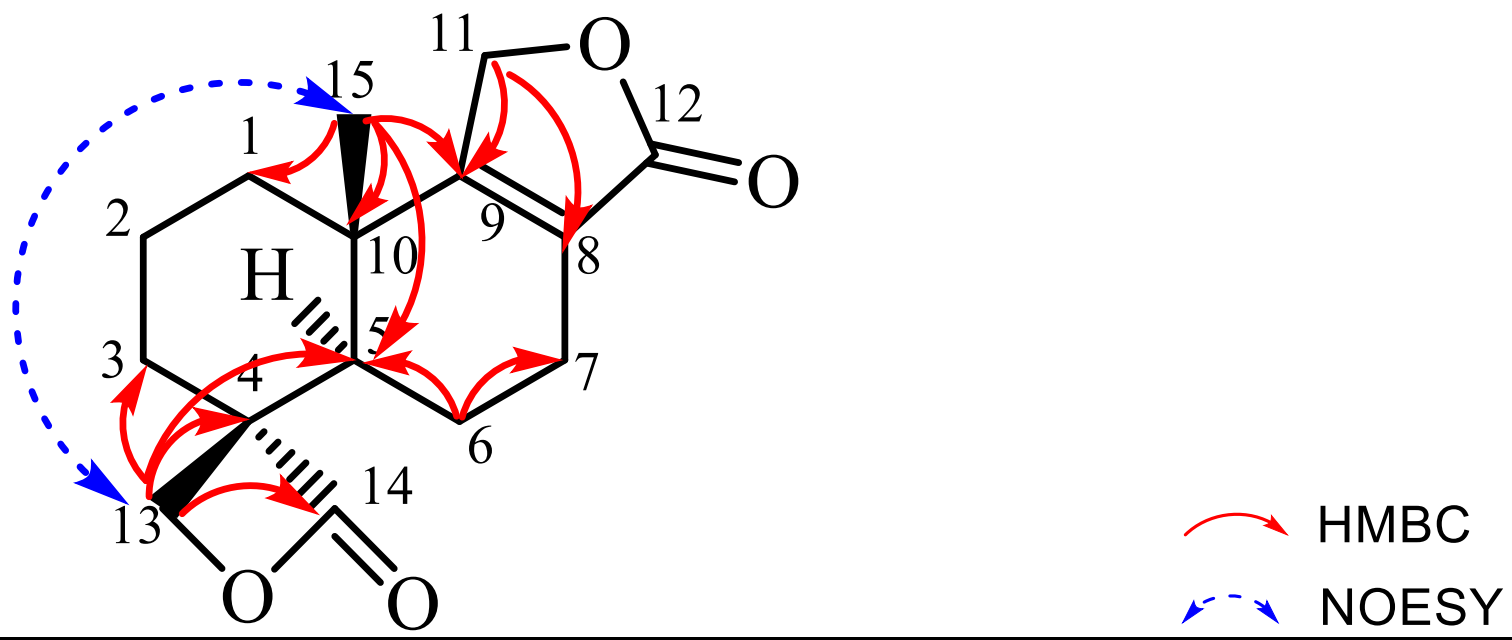

Position	δ_H (mult. *J* in Hz)	δ_C	Key HMBC	Key NOESY
1	1.71 (m)	34.9		
	1.51 (td, 13.7, 4.1)			
2	1.84 (m)[a]	18.3		
	1.66 (m)			
3	2.15 (m)	32.5		
	1.90 (m)[b]			
4		61.1		
5	1.88 (m)[b]	42.9		
6	1.82 (m)[a]	20.1	C-5, C-7	
	1.78 (ddd, 12.3, 5.8, 1.4)			
7	2.47 (m)	20.6		
	2.31 (m)			
8		124.7		
9		167.3		
10		35.8		
11	4.73 (dt, 17.1, 2.7)	68.29	C-8, C-9	
	4.70 (dt,17.1, 1.8)			
12		173.7		
13	4.39 (dd, 5.4, 1.5)	68.31	C-3, C-4, C-5, C-14	H-15
	4.16 (d, 5.4)			
14		173.9		
15	1.03 (s)	19.8	C-1, C-5, C-9, C-10	

[a, b]: signals with the same letters overlapping with each other.

Table S23. NMR data of ustidrimane N (**20**) in $CDCl_3$.

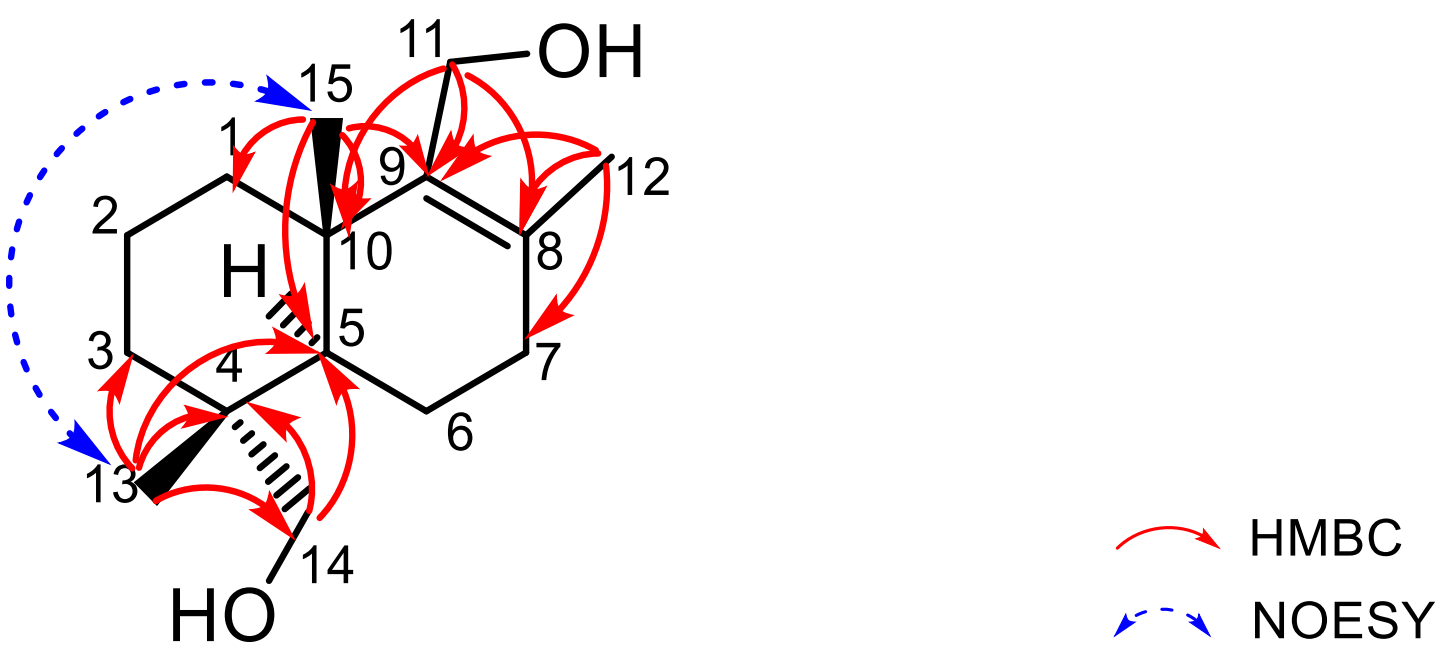

**Position**	δ_H **(mult.** ***J*** **in Hz)**	δ_C	**Key HMBC**	**Key NOESY**
1	1.91 (dt, 12.8, 3.3)	36.4		
	1.27 (td, 13.5, 4.0)[a]			
2	1.62 (m)[b]	18.4		
	1.61 (m)[b, c]			
3	1.42 (m)[d]	34.9		
	1.27 (m)[a]			
4		37.7		
5	1.46 (m)[e]	45.2		
6	1.58 (m)[c]	18.7		
	1.44 (m)[d, e]			
7	2.10 (m)[f]	33.3		
	2.09 (m)[f]			
8		132.4		
9		141.0		
10		37.8		
11	4.20 (d, 11.5)	58.4	C-8, C-9, C-10	
	4.05 (d, 11.5)			
12	1.72 (br s)	19.4	C-7, C-8, C-9	
13	0.80 (s)	17.5	C-3, C-4, C-5, C-14	H-15
14	3.44 (d, 10.9)	72.1	C-4, C-5	
	3.17 (d, 10.9)			
15	1.00 (s)	21.1	C-1, C-5, C-9, C-10	

[a, b, c, d, e, f]: signals with the same letters overlapping with each other.

Table S24. NMR data of ustidrimane O (**21**) in DMSO-d_6.

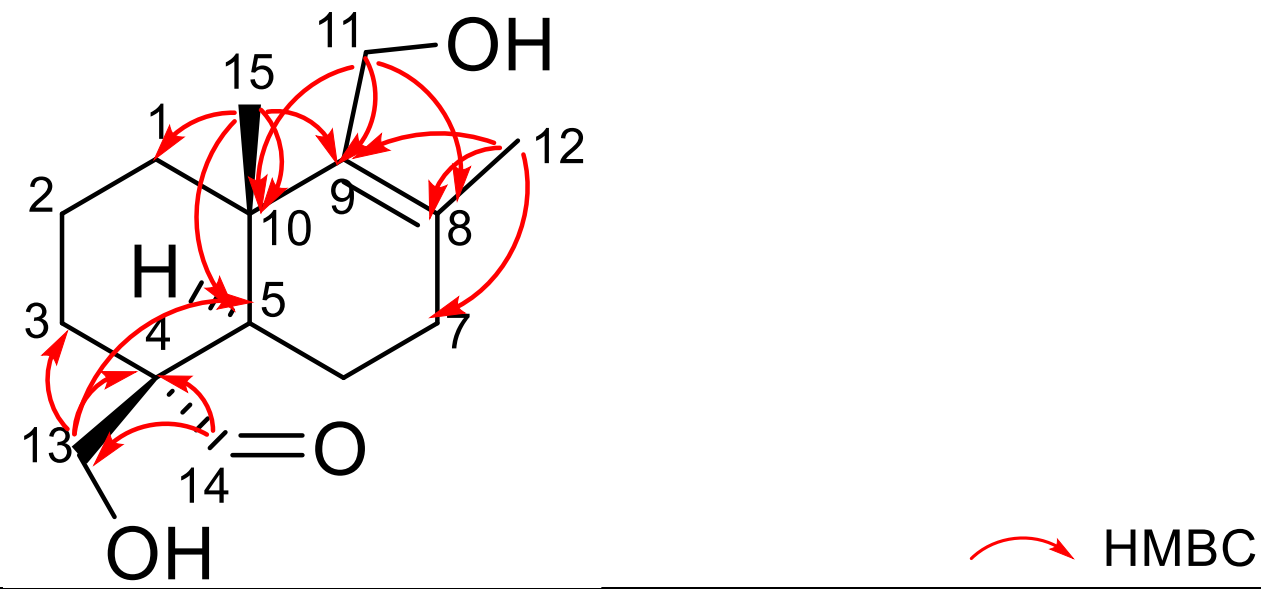

Position	δ_H (mult. *J* in Hz)	δ_C	Key HMBC
1	1.85 (m)[a]	35.3	
	1.29 (td, 12.8, 4.2)[b]		
2	1.54 – 1.50 (m)[c]	17.1	
3	1.72 (br d, 13.2)	26.7	
	1.22 (td, 12.9, 4.3)[b]		
4		53.7	
5	1.54 (m)[c]	44.9	
6	1.57 (m)[d]	20.1	
	1.43 (m)		
7	1.95 – 1.90 (m)[a]	32.7	
8		129.3	
9		140.1	
10		36.8	
11	3.95 (d, 11.2)	56.0	C-8, C-9, C-10
	3.84 (d, 11.2)[e]		
12	1.59 (br s)[d]	19.0	C-7, C-8, C-9
13	3.84 (d, 11.2)[e]	58.8	C-3, C-4, C-5
	3.78 (br d, 11.2)		
14	9.60 (br s)	208.2	C-4, C-13
15	0.94 (br s)	21.1	C-1, C-5, C-9, C-10

[a, b, c, d, e]: signals with the same letters overlapping with each other.

Table S25. NMR data of ustidrimane P (**22**) in $CDCl_3$.

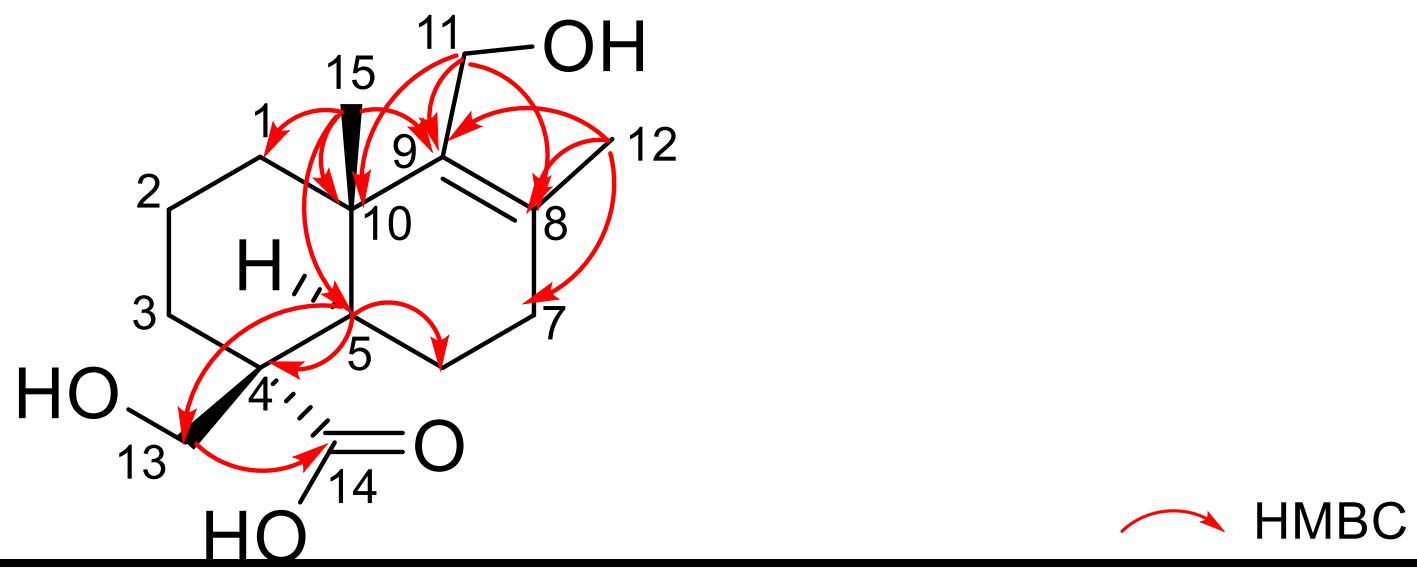

Position	δ_H (mult. *J* in Hz)	δ_C	Key HMBC
1	1.94 (m)[a]	36.0	
	1.37 (m)[b]		
2	1.63 – 1.60 (m)[c]	18.1	
3	2.12 (m)[d]	30.1	
	1.64 (m)[c]		
4		52.0	
5	2.05 (m)[d]	46.3	C4, C-13
6	1.77 (br s)	20.6	
	1.45 (m)[b]		
7	2.13 (m)[d]	33.6	
	1.96 (m)[a]		
8		132.8	
9		140.2	
10		37.4	
11	4.20 (d, 11.2)	58.2	C-8, C-9, C-10
	4.03 (d, 11.2)		
12	1.70 (s)	19.4	C-7, C-8, C-9
13	3.89 (br d, 12.5)[e]	61.3	C-14
	3.83 (br d, 12.5)[e]		
14		180.1	
15	0.97 (s)	21.3	C-1, C-5, C-9, C-10

[a, b, c, d, e]: signals with the same letters overlapping with each other.

Table S26. NMR data of ustidrimane Q (**23**) in DMSO-d_6.

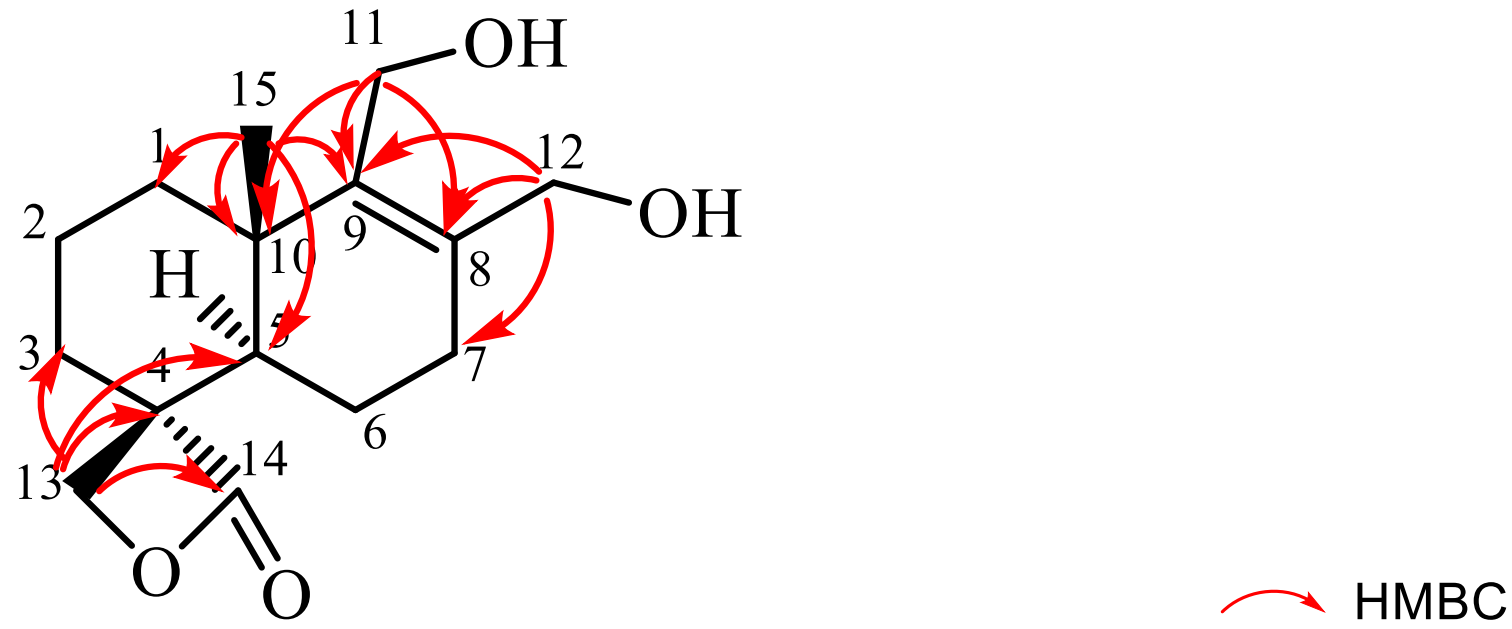

Position	δ_H (mult. *J* in Hz)	δ_C	Key HMBC
1	1.86 (br d, 13.0)	34.4	
	1.26 (td, 13.0, 4.4)		
2	1.65 – 1.60 (m)[a]	18.2	
3	2.02 (br d, 13.1)	31.8	
	1.58 (m)[a]		
4		60.9	
5	1.52 (dd, 12.8, 2.2)	42.9	
6	1.73 (m)	20.0	
	1.37 (dd, 12.0, 7.3)		
7	2.30 (dd, 18.1, 6.2)	27.5	
	2.11 (ddd, 18.1, 10.3, 7.0)		
8		134.9	
9		140.9	
10		36.7	
11	4.00 (d, 11.8)	55.3	C-8, C-9, C-10
	3.86 (d, 11.8)		
12	4.07 (d, 12.2)	61.0	C-7, C-8, C-9
	3.81 (d, 12.2)		
13	4.43 (d, 5.4)	68.4	C-3, C-4, C-5, C-14
	4.27 (d, 5.4)		
14		175.2	
15	0.78 (s)	18.9	C-1, C-5, C-9, C-10

[a]: signals with the same letters overlapping with each other.

Supplementary Figures

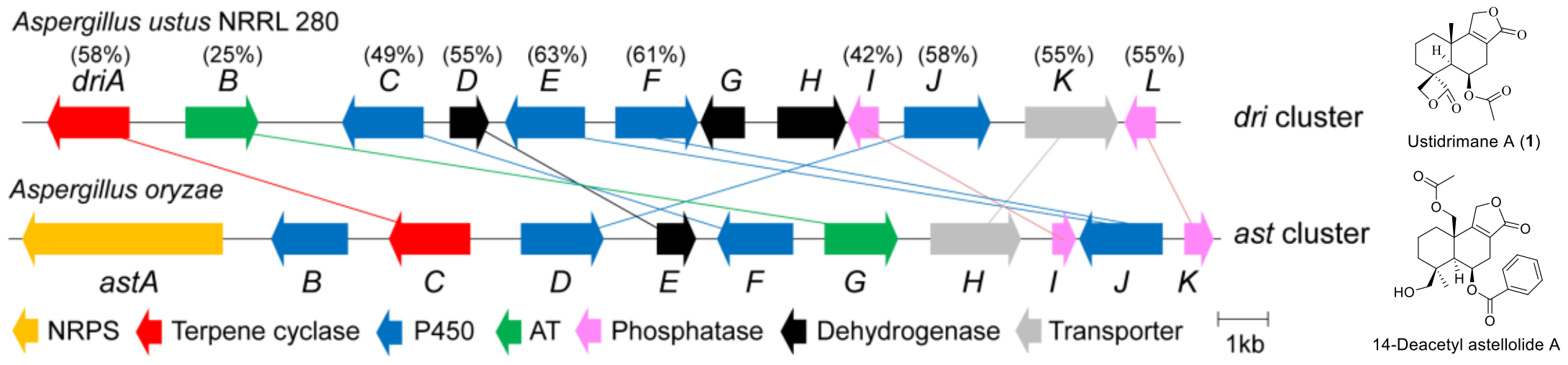

Figure S1. Comparative analysis of the *dri* and *ast* clusters. Amino acid identities of the respective homologous proteins are given in percent.

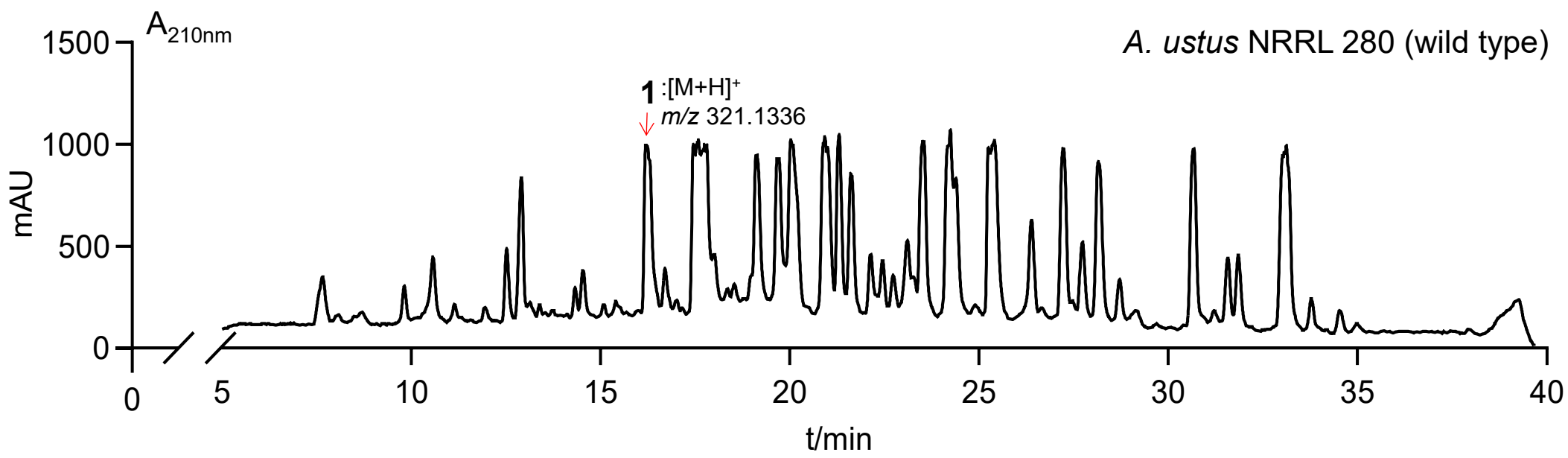

Figure S2. LC-MS chromatogram of the metabolites from *A. ustus* NRRL 280. Absorptions at UV 210 nm are displayed.

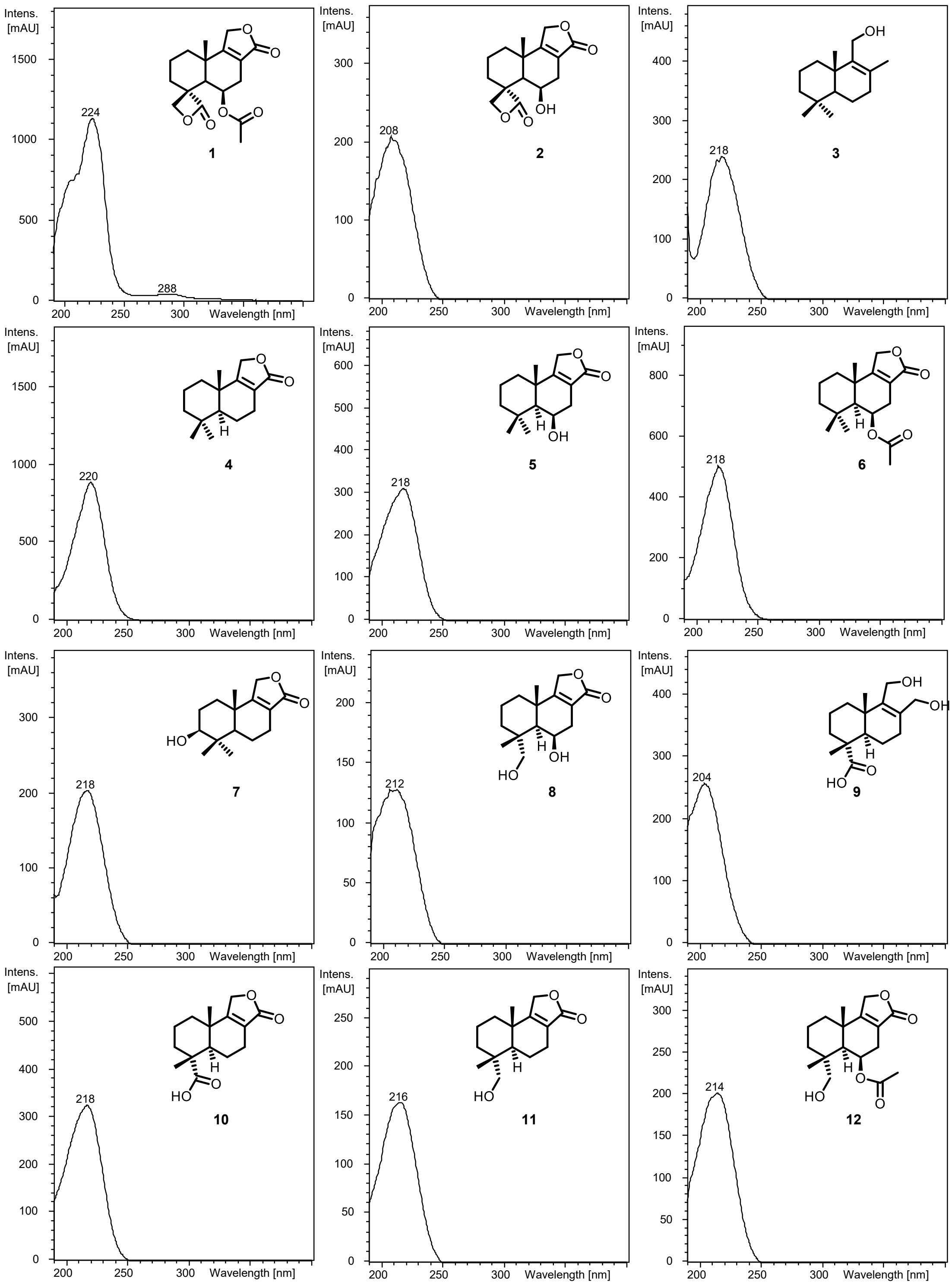

Figure S3. UV spectra of compounds **1–23**.

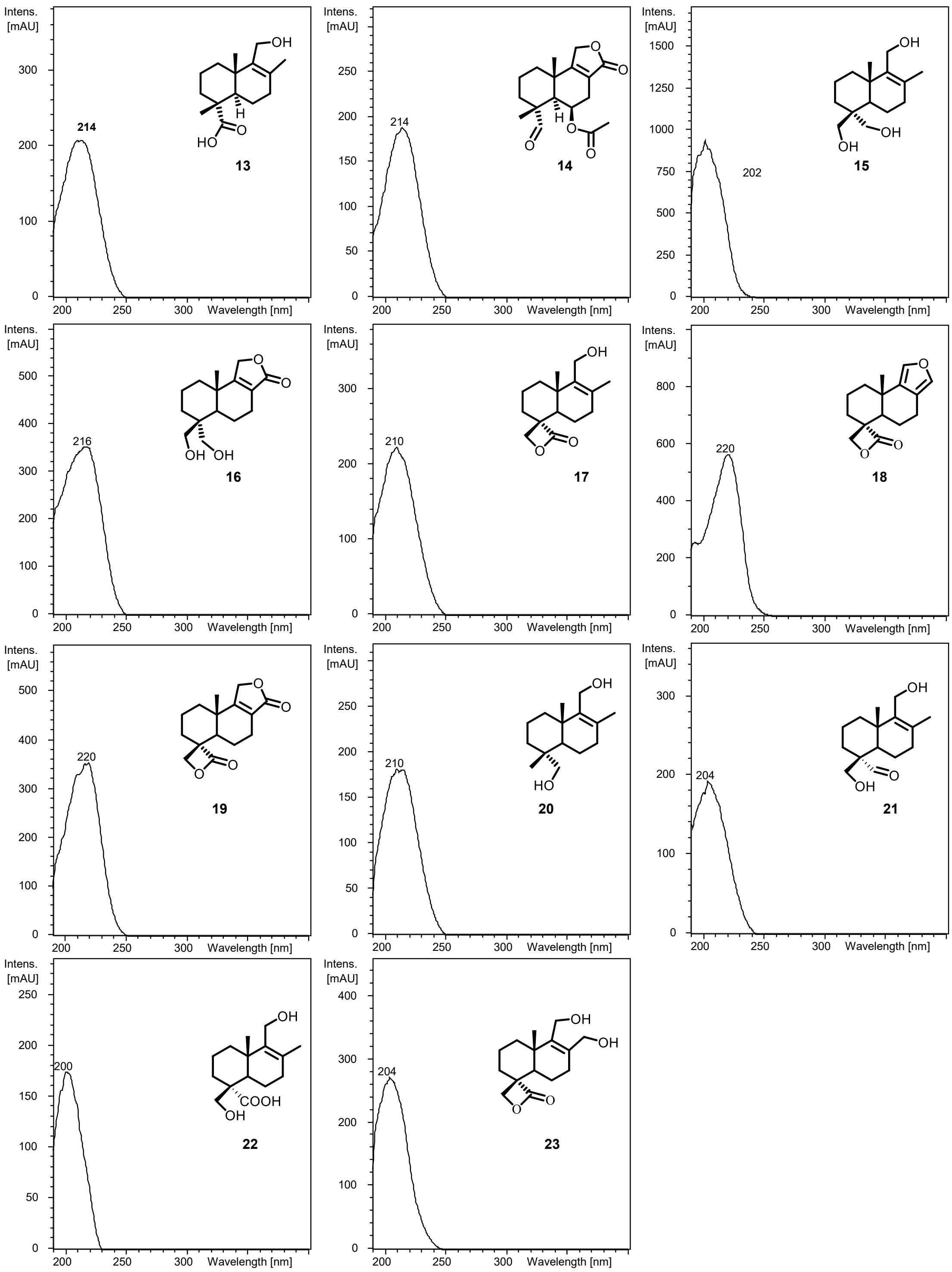

Figure S3. UV spectra of compounds **1–23** (continued).

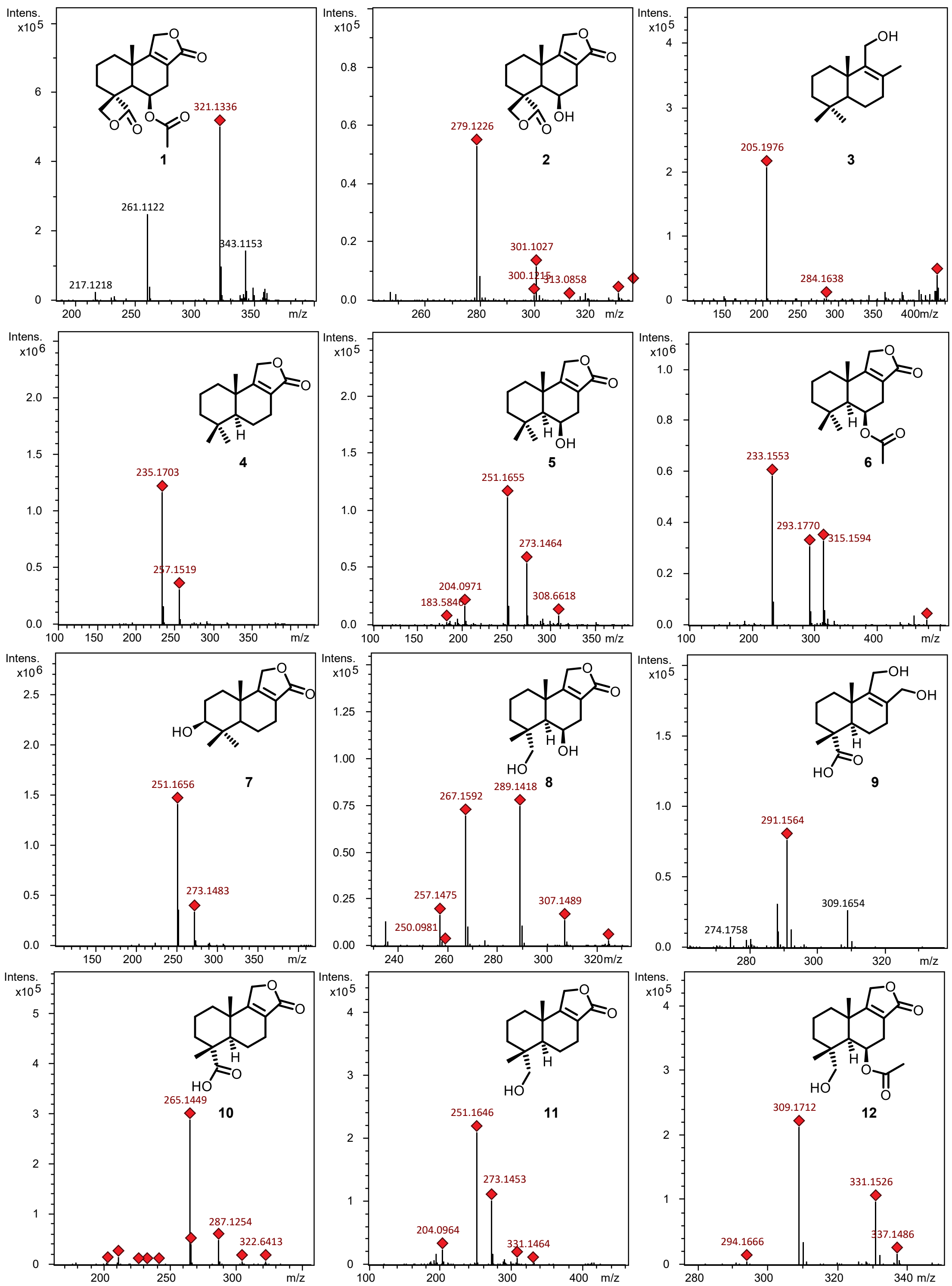

Figure S4. MS spectra of compounds **1–23**.

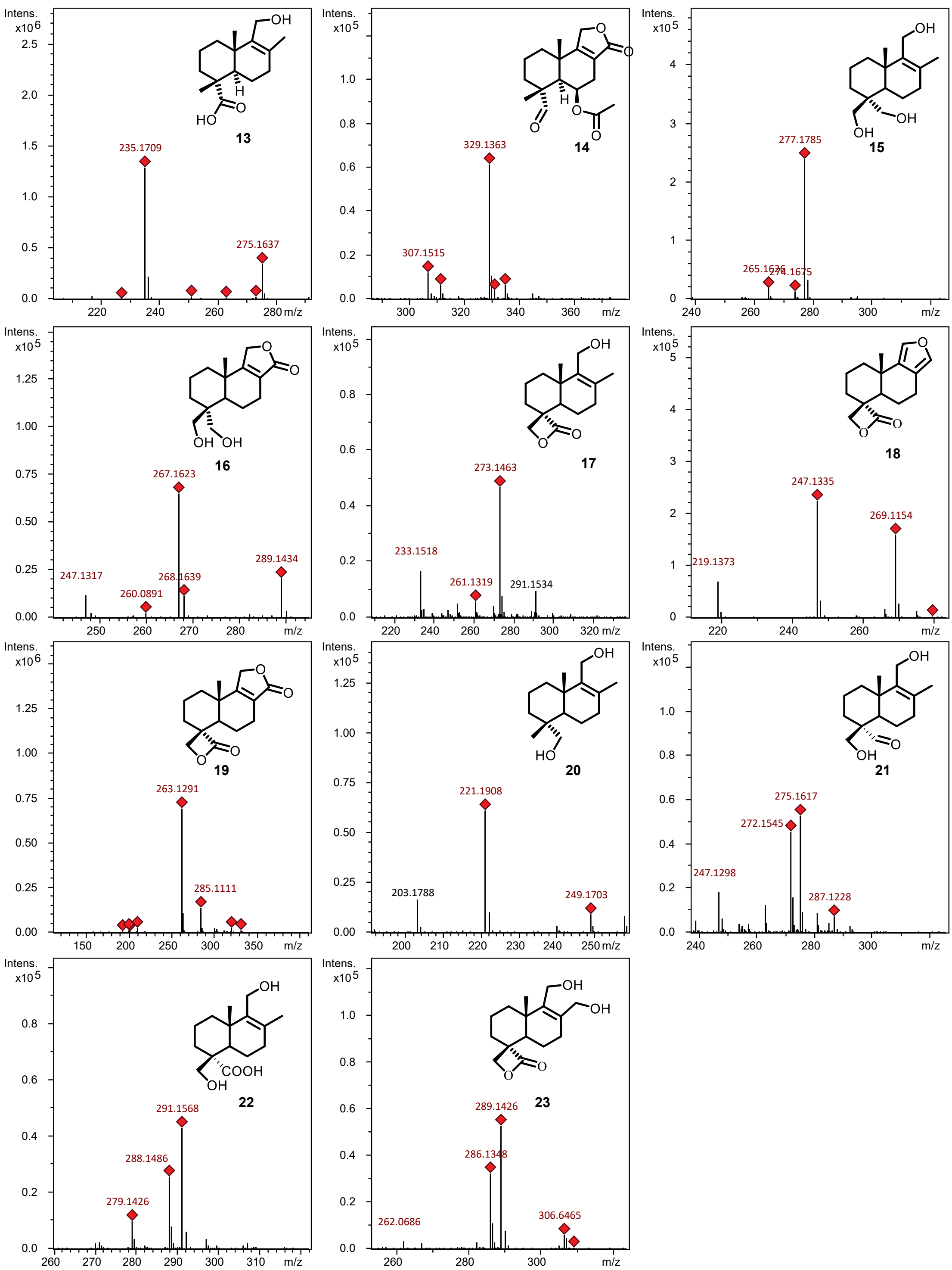

Figure S4. MS spectra of compounds **1-23** (continued).

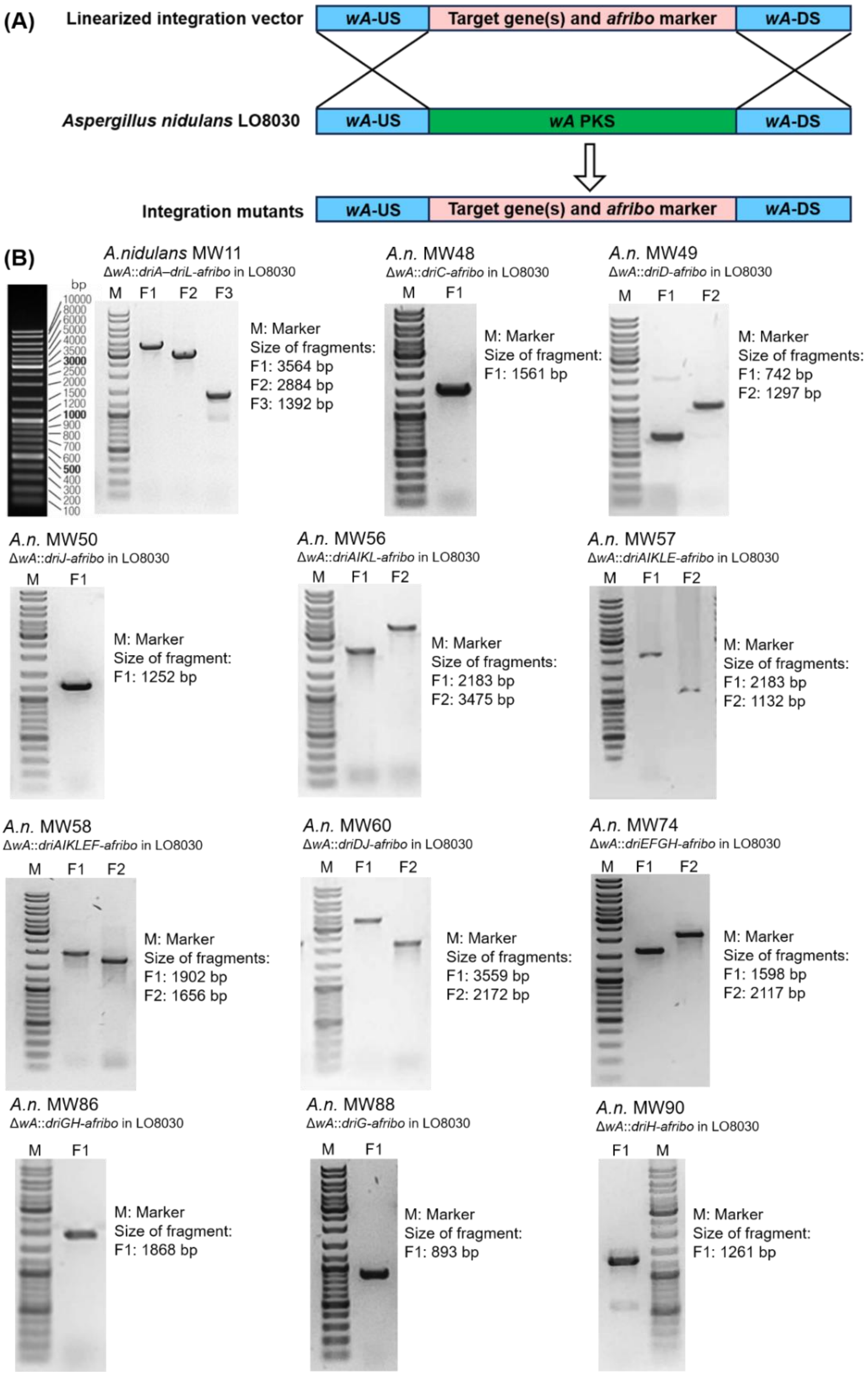

Figure S5. PCR verification of *A. nidulans* recombinant strains. (A) Schematic representation of the targeted integration into the wA-PKS locus of *A. nidulans* LO8030, yielding recombinant strains. *wA*-US and *wA*-DS represent the upstream and downstream flanking regions of *wA*-PKS locus. (B) PCR verification of genomic integration in *A. nidulans* transformant MW11, MW48, MW49, MW50, MW56, MW57, MW58, MW60, MW74, MW86, MW88, and MW90.

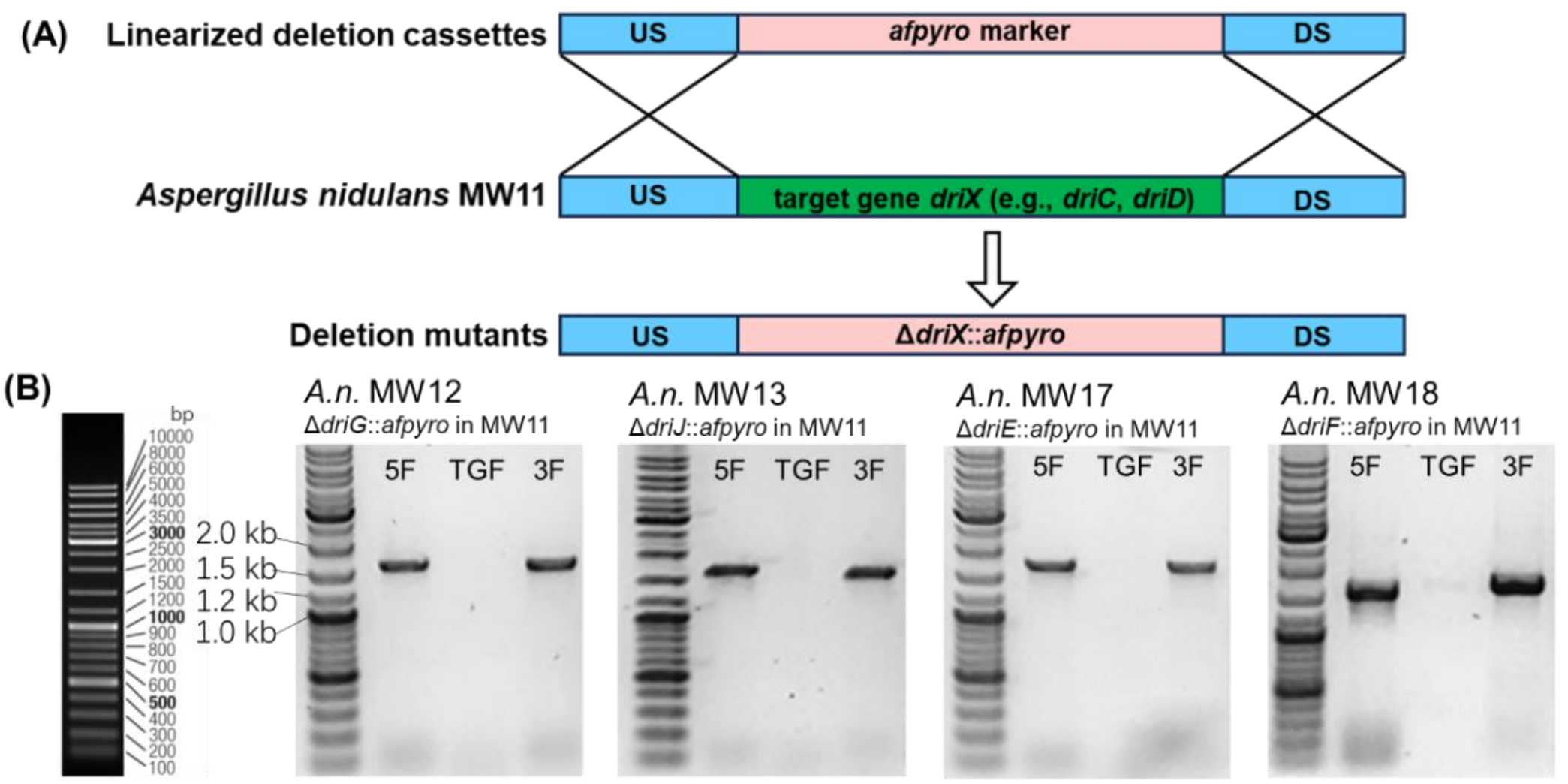

**Size of fragments**	**5F**: 1598 bp<br>**TGF**: 893 bp<br>**3F**: 1619 bp	**5F**: 1616 bp<br>**TGF**: 1252 bp<br>**3F**: 1581 bp	**5F**: 1689 bp<br>**TGF**: 1132 bp<br>**3F**: 1660 bp	**5F**: 1559 bp<br>**TGF**: 1472 bp<br>**3F**: 1674 bp

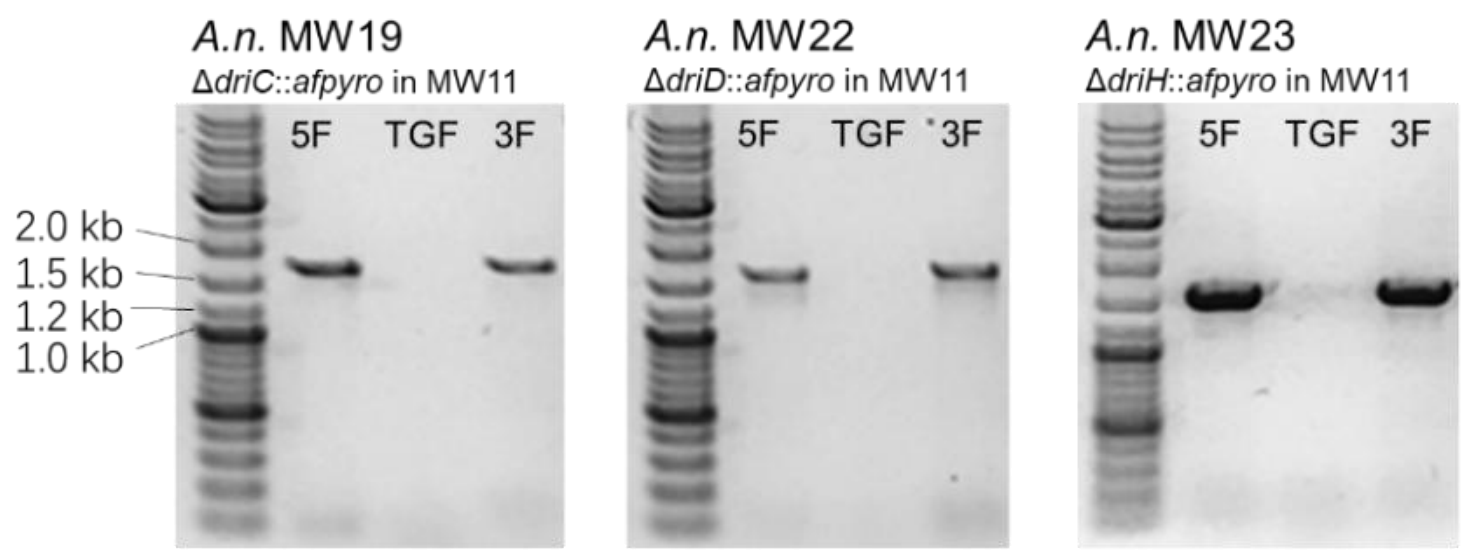

**Size of fragments**	**5F**: 1652 bp<br>**TGF**: 1146 bp<br>**3F**: 1712 bp	**5F**: 1612 bp<br>**TGF**: 742 bp<br>**3F**: 1673 bp	**5F**: 1572 bp<br>**TGF**: 1402 bp<br>**3F**: 1616 bp

Figure S6. PCR verification of single deletion mutants. (A) Schematic strategy for deleting selected genes in *A. nidulans* MW11. The target gene (*driG*, *driJ, driE, driF, driC, driD,* or *driH*) was replaced by the *afpyro* selectable marker through homologous recombination, yielding MW12, MW13, MW17, MW18, MW19, MW22 and MW23, respectively. US and DS represent the upstream and downstream flanking regions of the target gene, which were used as homologous arms for gene replacement. (B) PCR verification of the *dri* gene deletion mutants in *A. nidulans* MW11, including MW12 Δ*driG*, MW13 Δ*driJ*, MW17 Δ*driE*, MW18 Δ*driF*, MW19 Δ*driC*, MW22 Δ*driD*, and MW23 Δ*driH*. For each mutant, the 5′ junction fragment (5F), target gene fragment (TGF), and 3′ junction fragment (3F) were verified. Amplification of 5F and 3F fragments, together with the absence of the TGF fragment, confirmed successful replacement of the target gene by the *afpyro* marker.

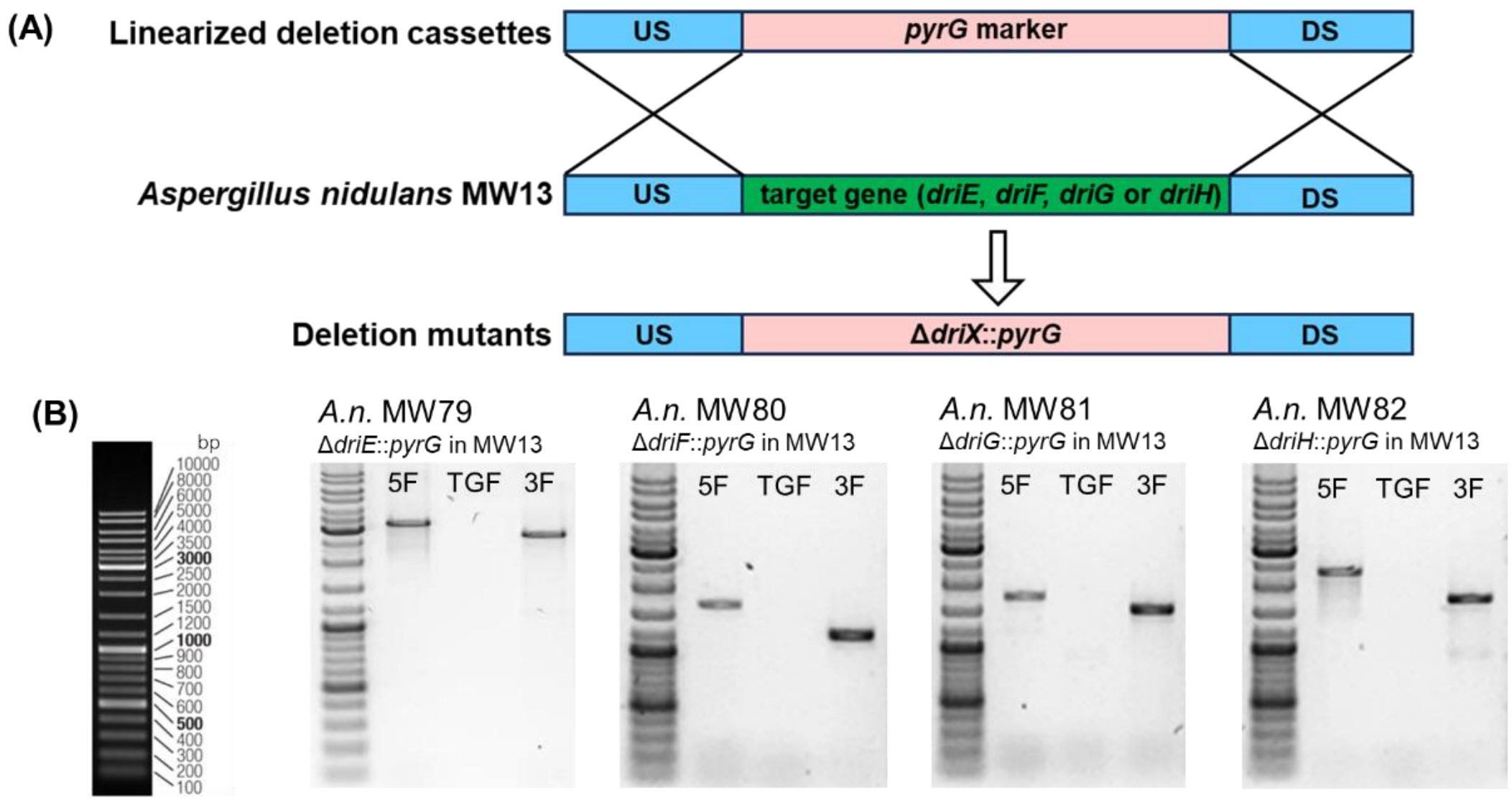

Size of fragments				
	**5F**: 3209 bp	**5F**: 1676 bp	**5F**: 1715 bp	**5F**: 2287 bp
	**TGF**: 1532 bp	**TGF**: 1472 bp	**TGF**: 893 bp	**TGF**: 1402 bp
	**3F**: 2760 bp	**3F**: 1194 bp	**3F**: 1497 bp	**3F**: 1689 bp

Figure S7. PCR verification of double deletion mutants. (A) Schematic strategy for deletion of selected genes in *A. nidulans* MW13. The target gene (*driE, driF, driG,* or *driH*) was replaced by the *pyrG* selectable marker through homologous recombination, yielding MW79, MW80, MW81, and MW82, respectively. US and DS represent the upstream and downstream flanking regions of the target gene, which were used as homologous arms for gene replacement. (B) PCR verification of *driE–driH* deletion mutants from *A. nidulans* MW13, which include MW79 (Δ*driJ*Δ*driE*), MW80 (Δ*driJ*Δ*driF*), MW81 (Δ*driJ*Δ*driG*), and MW82 (Δ*driJ*Δ*driH*). For each mutant, the 5′ junction fragment (5F), target gene fragment (TGF), and 3′ junction fragment (3F) were verified. Amplification of 5F and 3F fragments, together with the absence of the TGF fragment, confirmed successful replacement of the target gene by the *pyrG* marker.

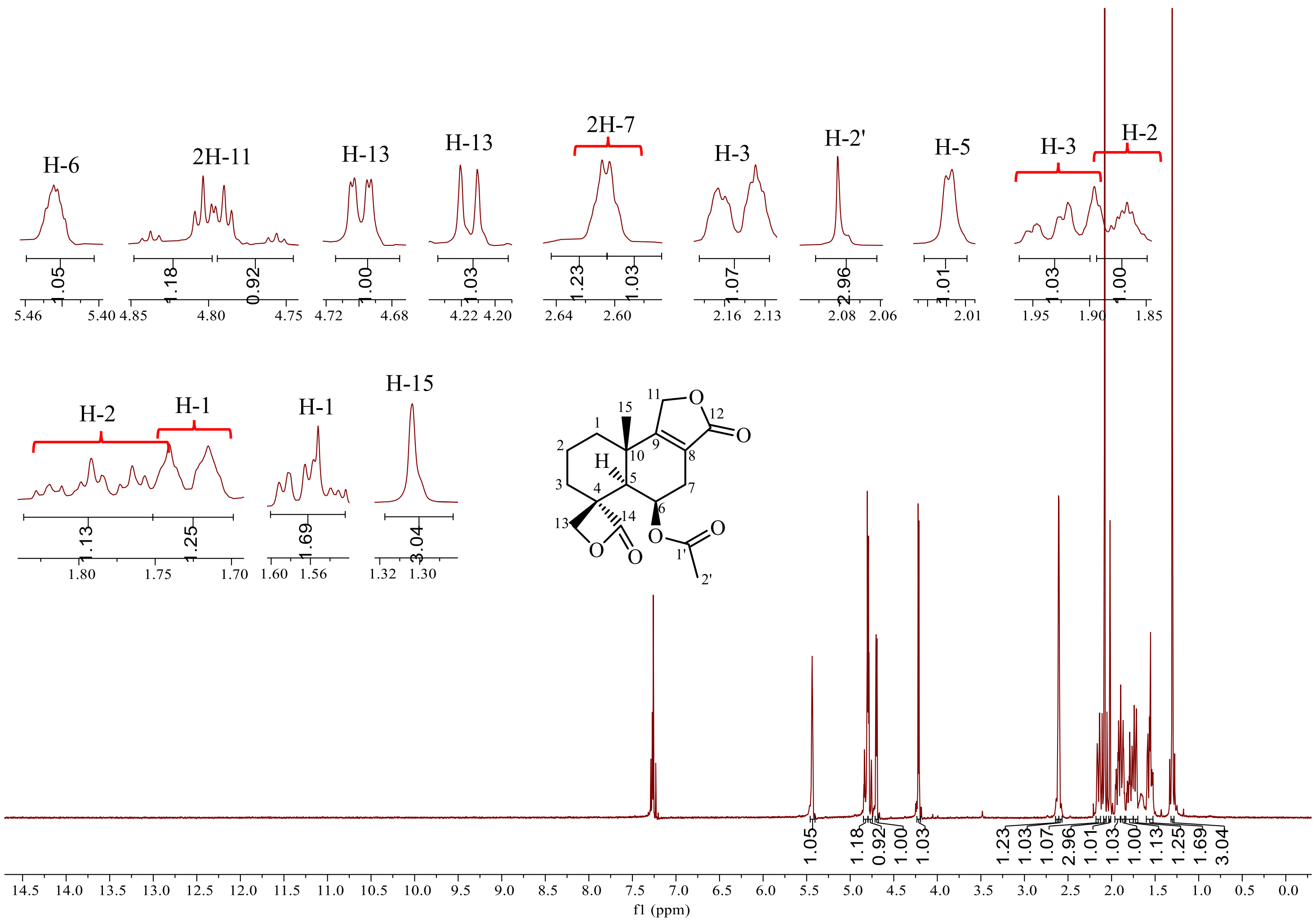

Figure S8. The ^{1}H NMR spectrum of ustidrimane A (**1**) in $CDCl_3$ (500 MHz).

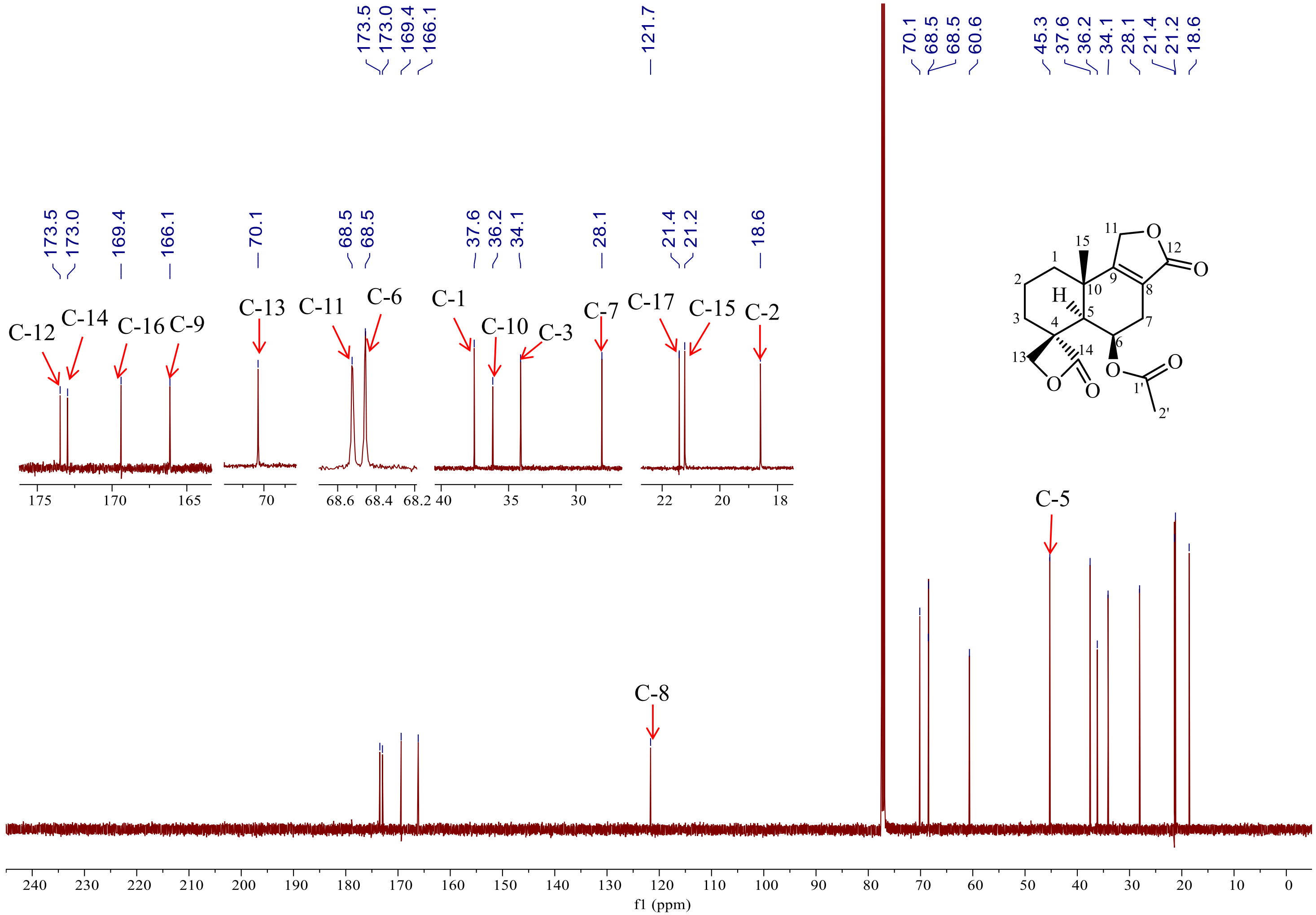

Figure S9. The ^{13}C NMR spectrum of ustidrimane A (**1**) in $CDCl_3$ (125 MHz).

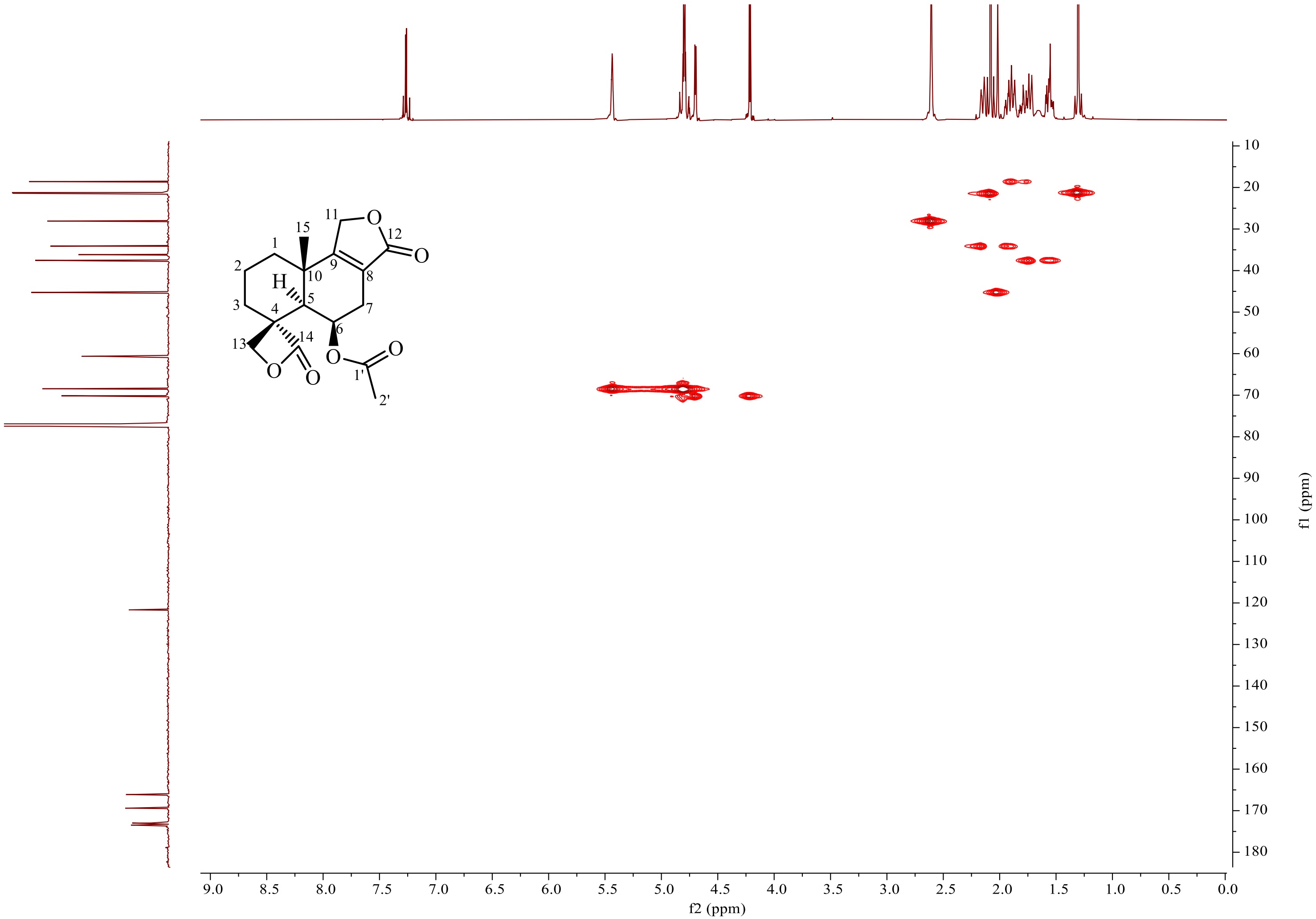

Figure S10. The HSQC spectrum of ustidrimane A (**1**) in $CDCl_3$.

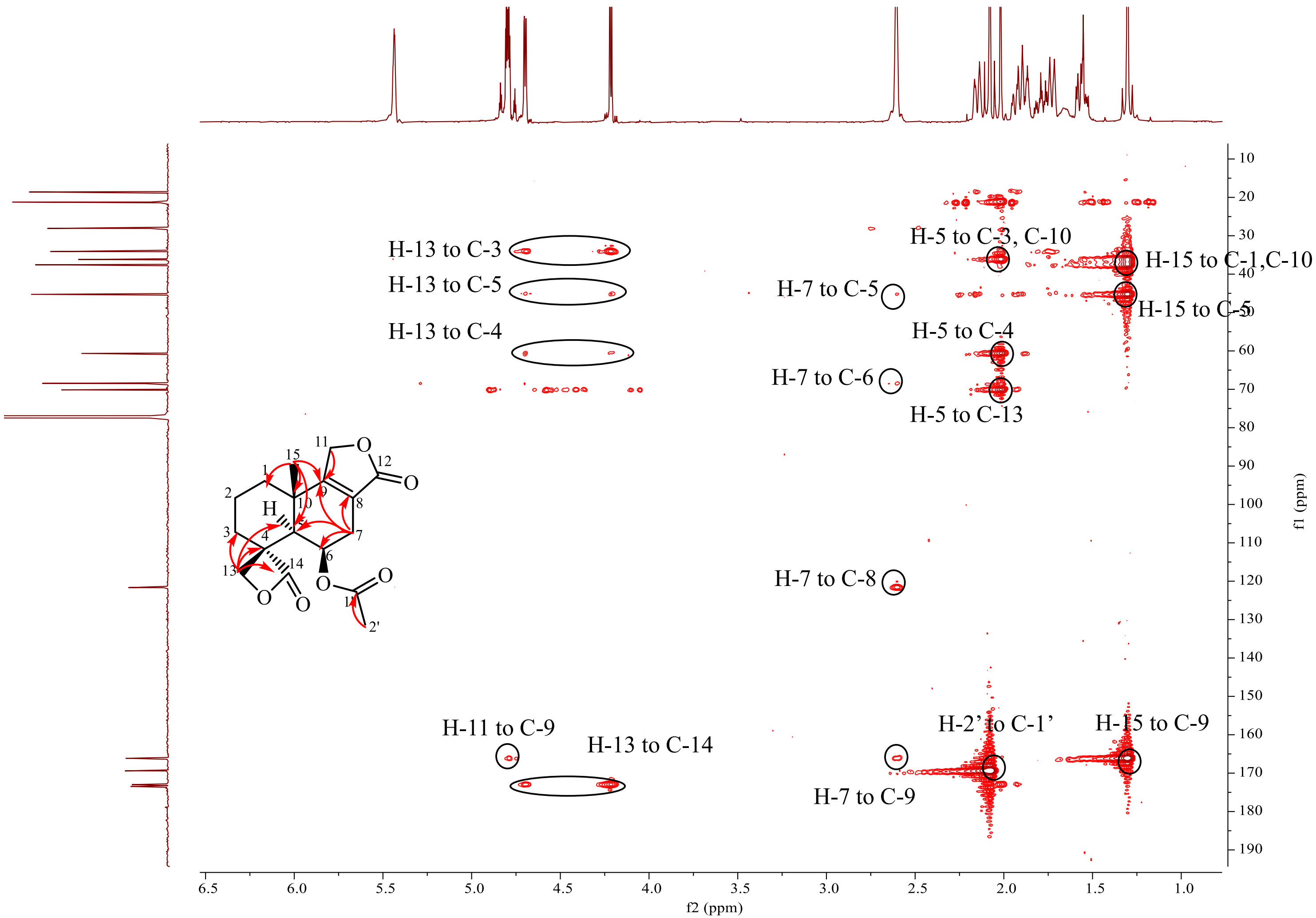

Figure S11. The HMBC spectrum of ustidrimane A (**1**) in $CDCl_3$.

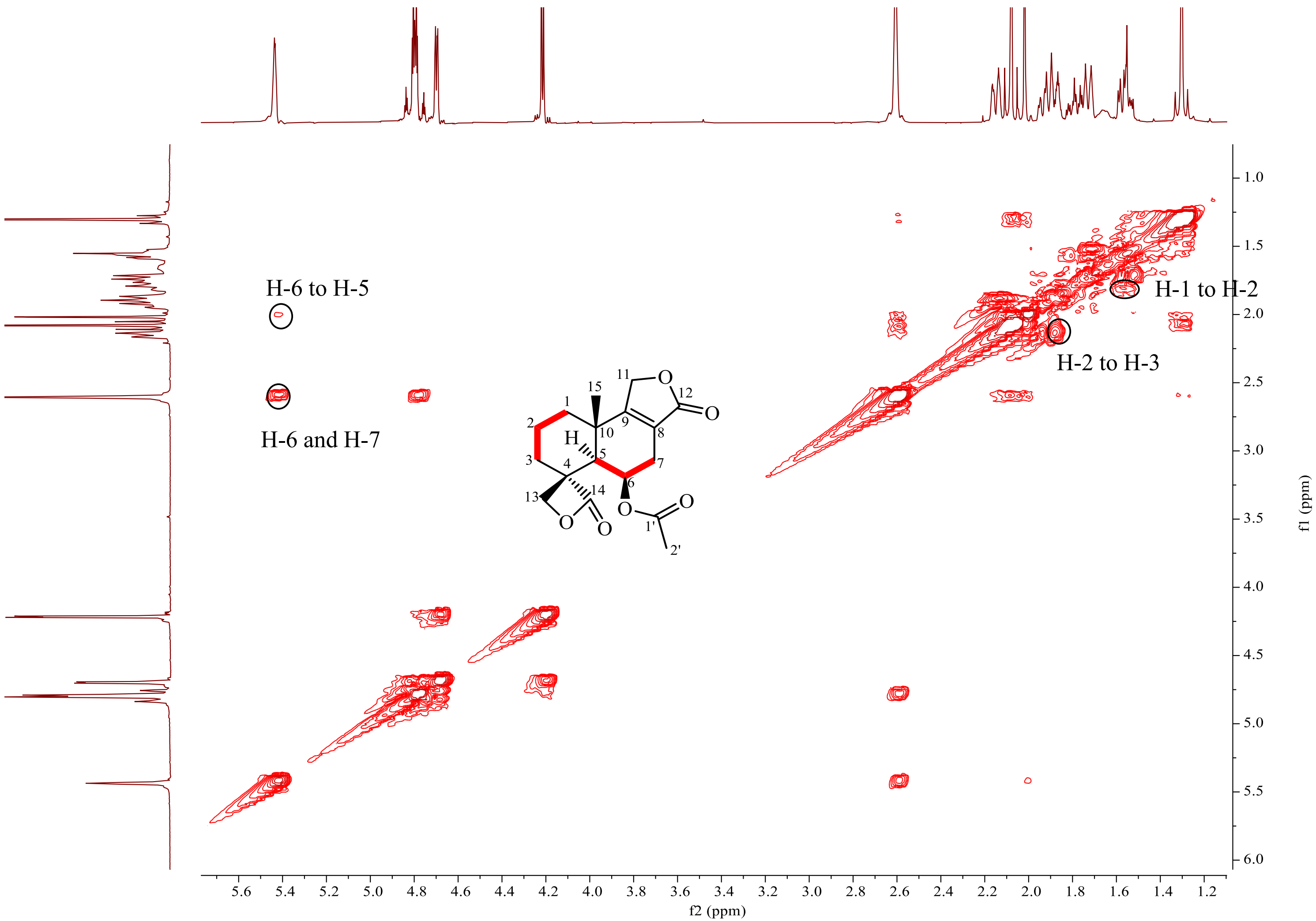

Figure S12. The ^{1}H-^{1}H COSY spectrum of ustidrimane A (**1**) in $CDCl_3$.

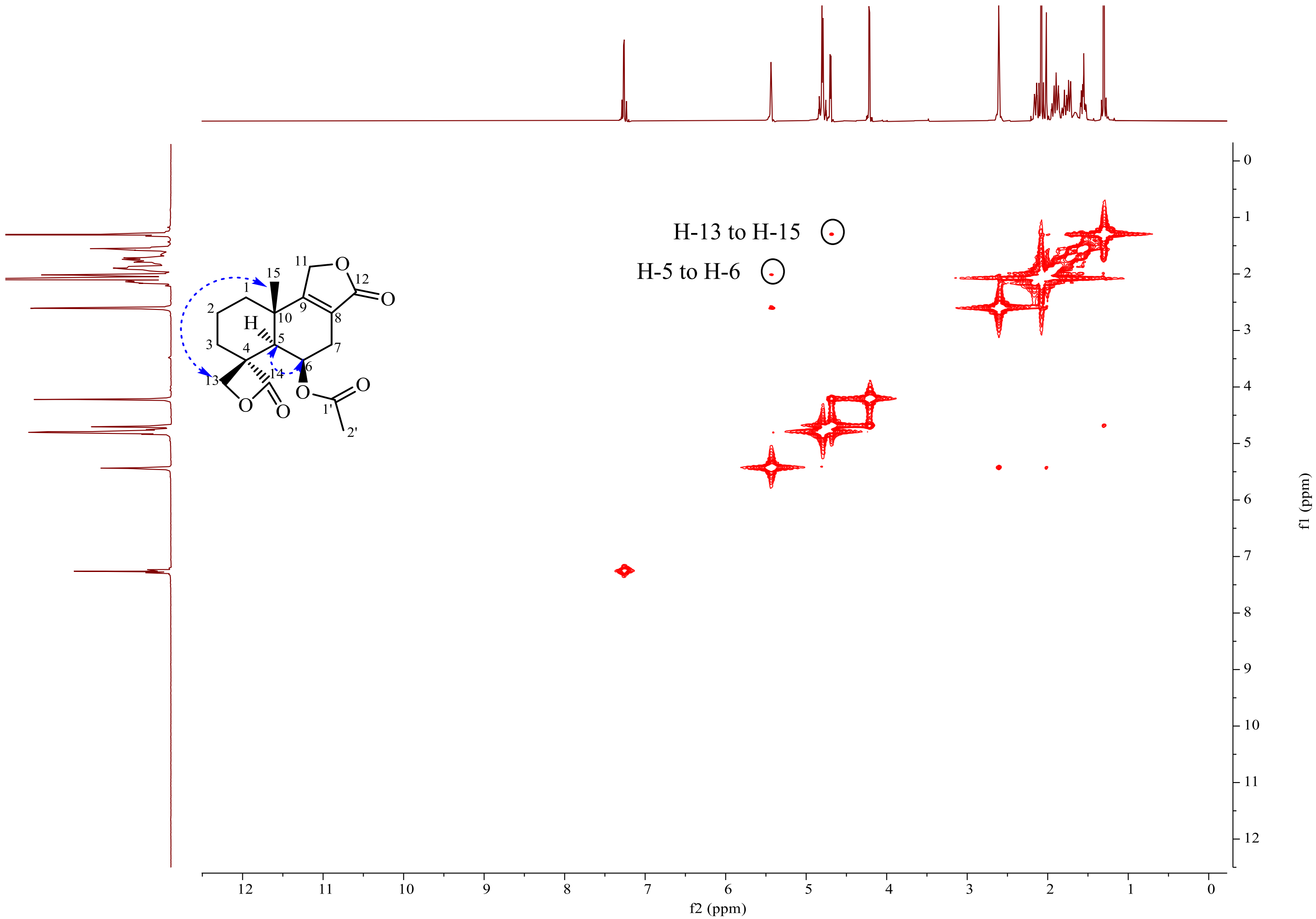

Figure S13. The NOESY spectrum of ustidrimane A (**1**) in $CDCl_3$.

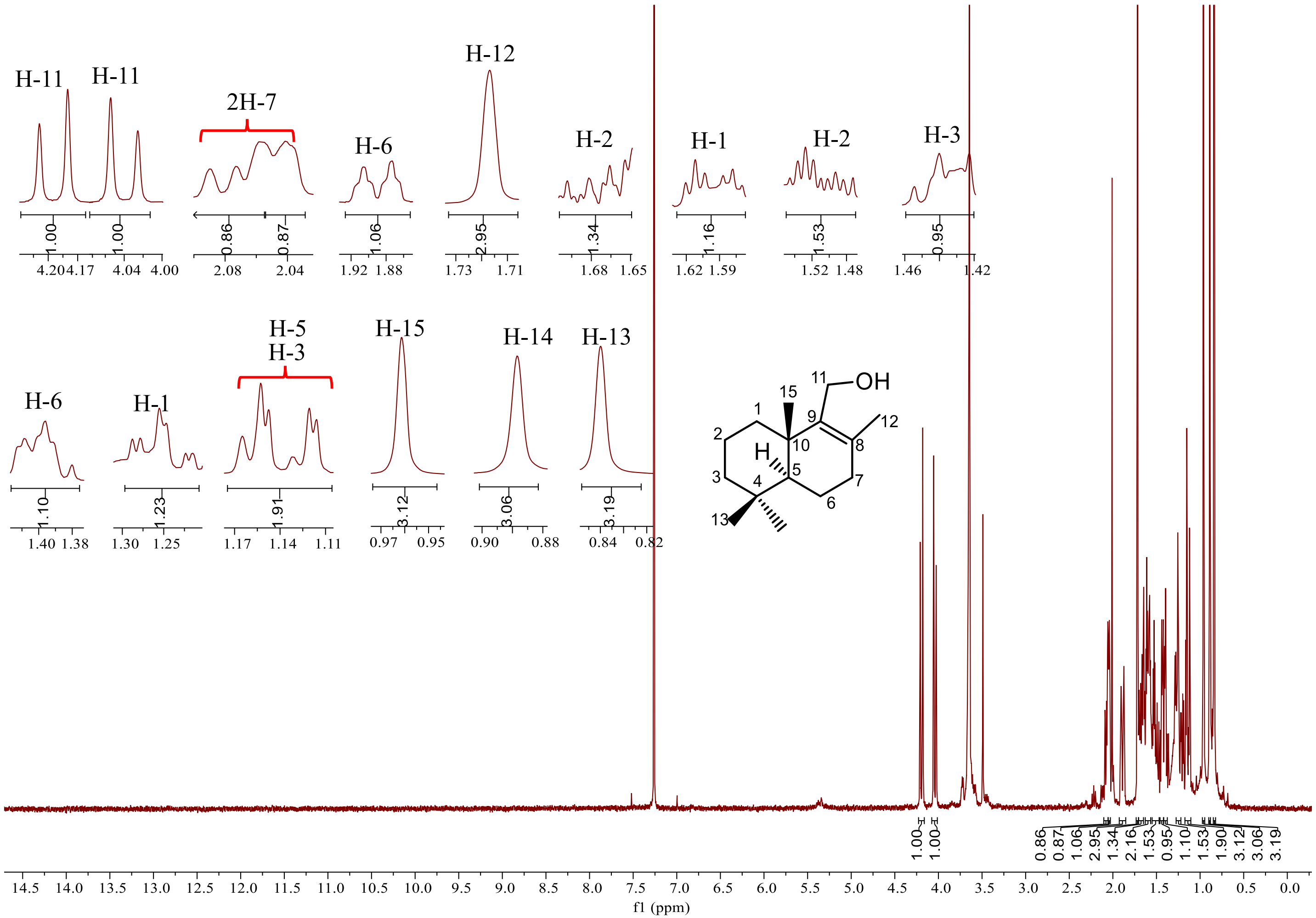

Figure S14. The ¹H NMR spectrum of drim-8-ene-11-ol (**3**) in $CDCl_3$ (400 MHz).

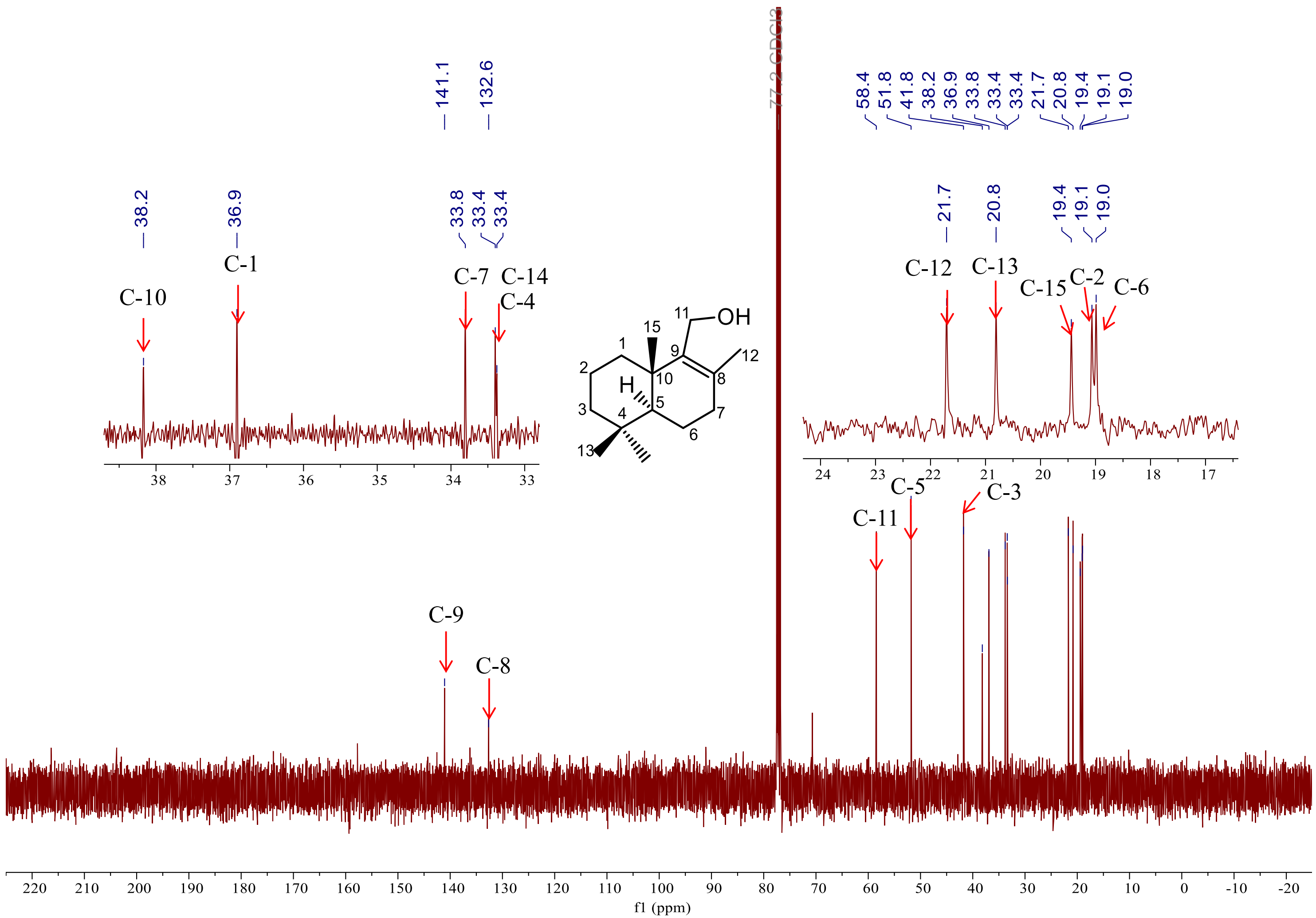

Figure S15. The ^{13}C NMR spectrum of drim-8-ene-11-ol (**3**) in $CDCl_3$ (100 MHz).

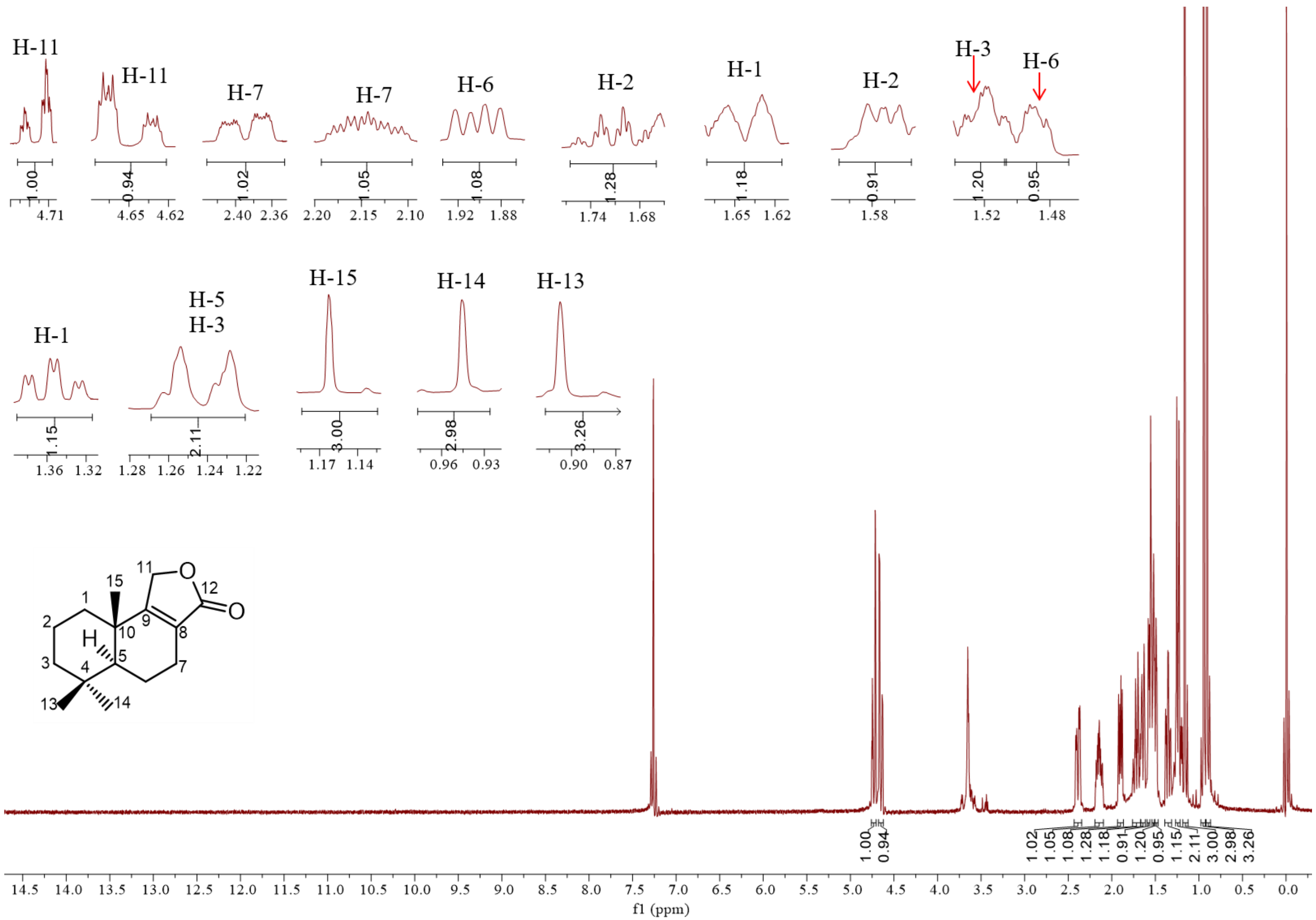

Figure S16. The ^{1}H NMR spectrum of confertifolin (**4**) in $CDCl_3$ (400 MHz).

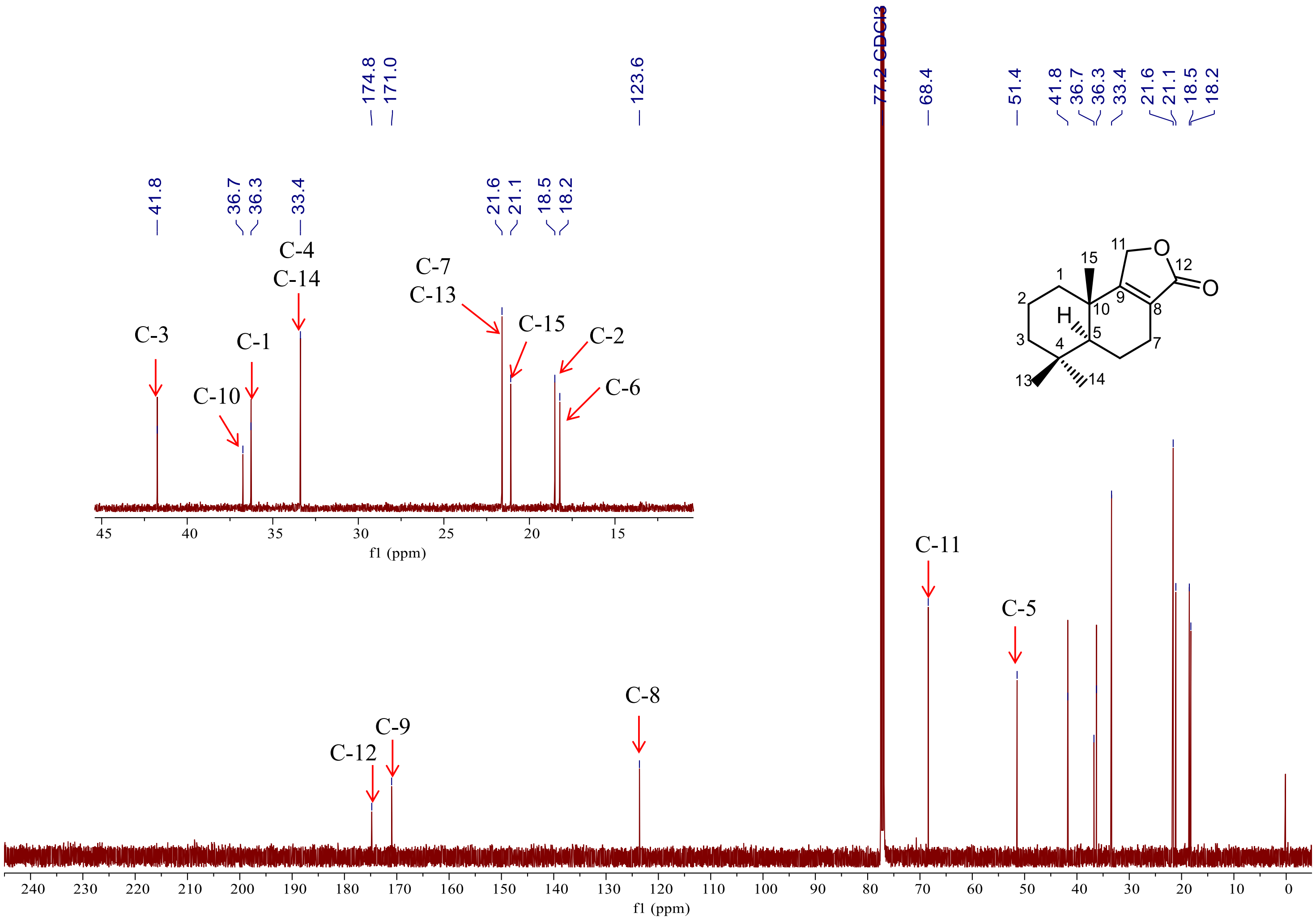

Figure S17. The ^{13}C NMR spectrum of confertifolin (**4**) in $CDCl_3$ (100 MHz).

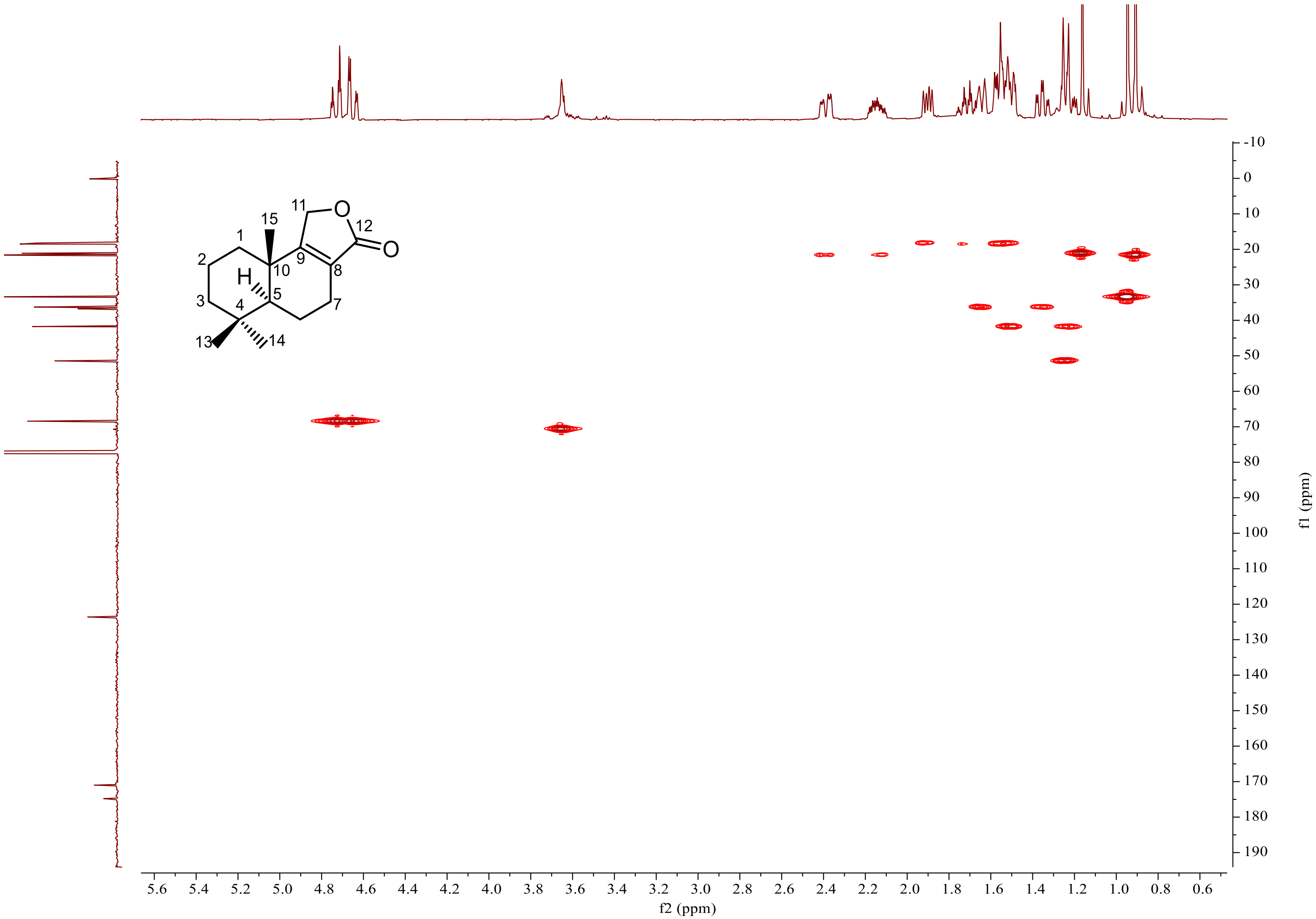

Figure S18. The HSQC spectrum of confertifolin (**4**) in $CDCl_3$.

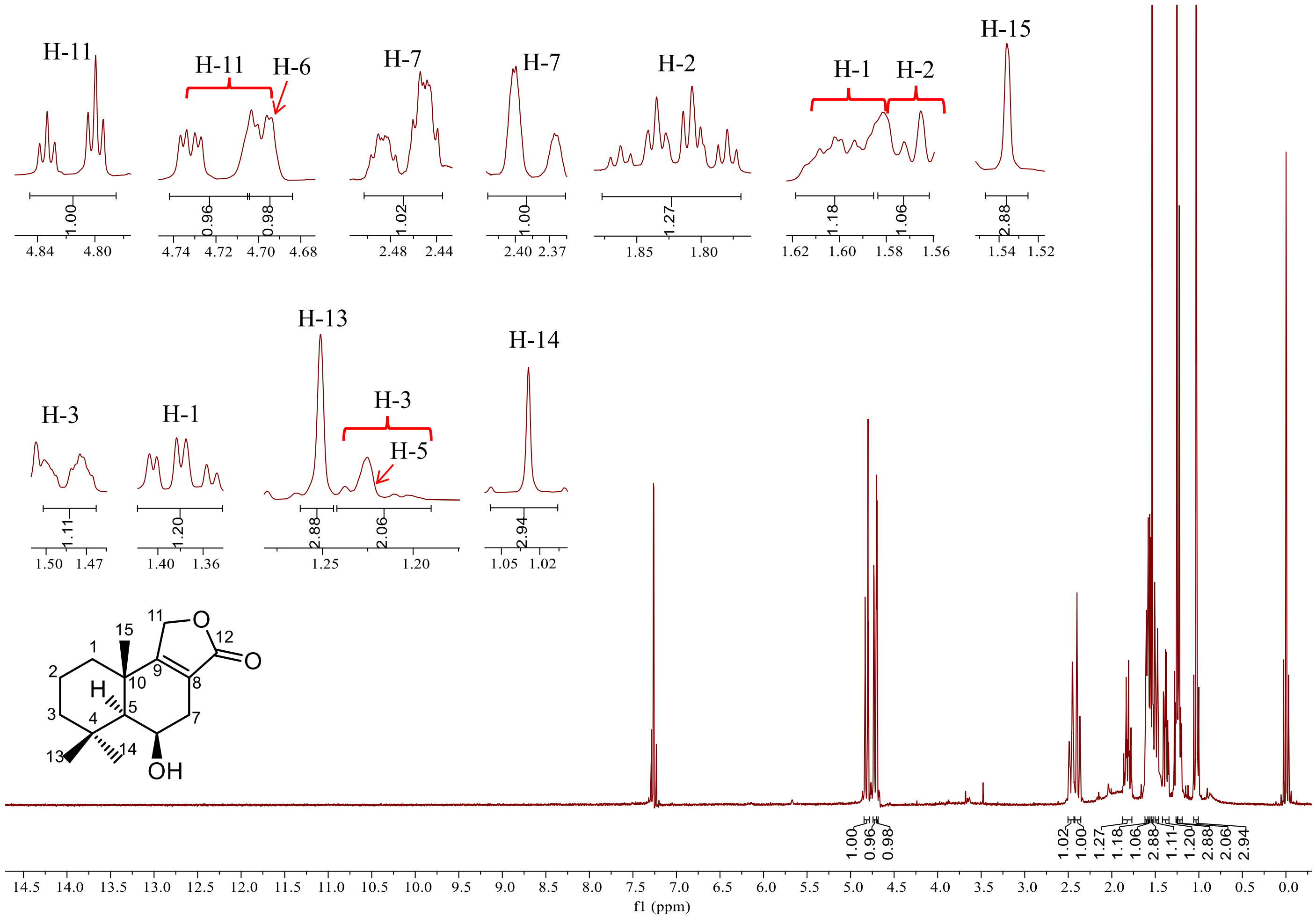

Figure S19. The ^{1}H NMR spectrum of 6*β*-hydroxyconfertifolin (**5**) in $CDCl_3$ (400 MHz).

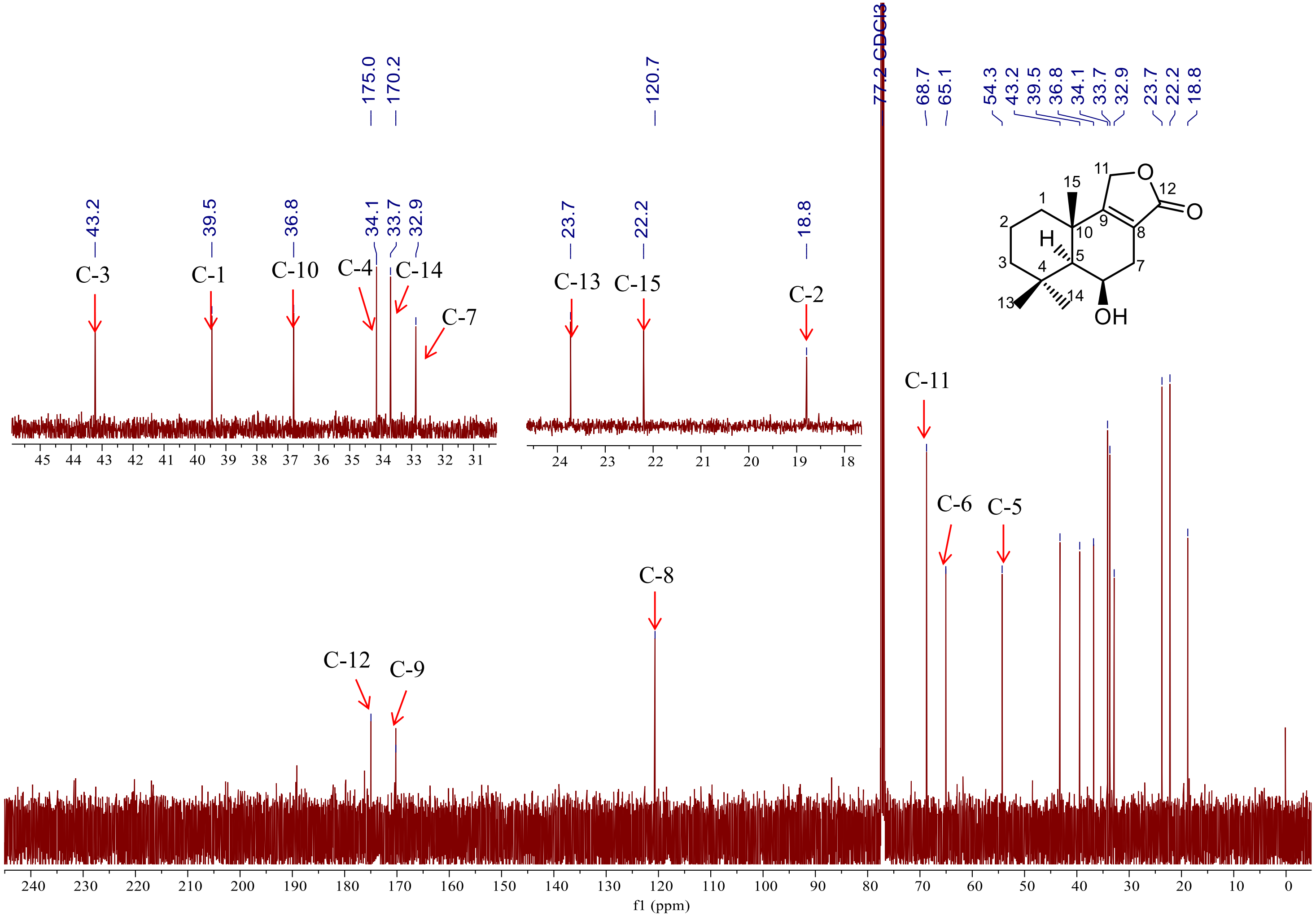

Figure S20. The ^{13}C NMR spectrum of 6*β*-hydroxyconfertifolin (**5**) in $CDCl_3$ (100 MHz).

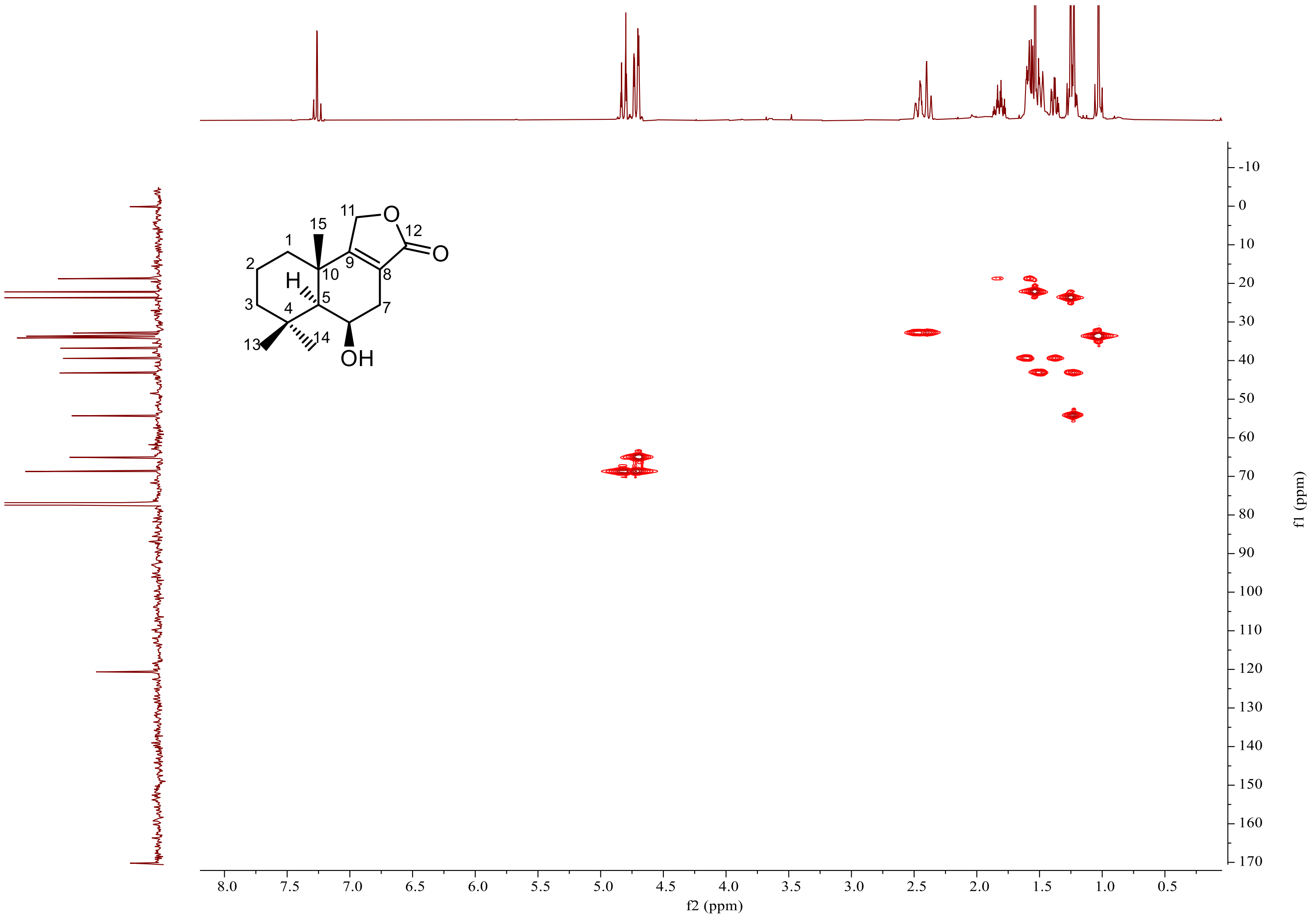

Figure S21. The HSQC spectrum of 6β-hydroxyconfertifolin (**5**) in $CDCl_3$.

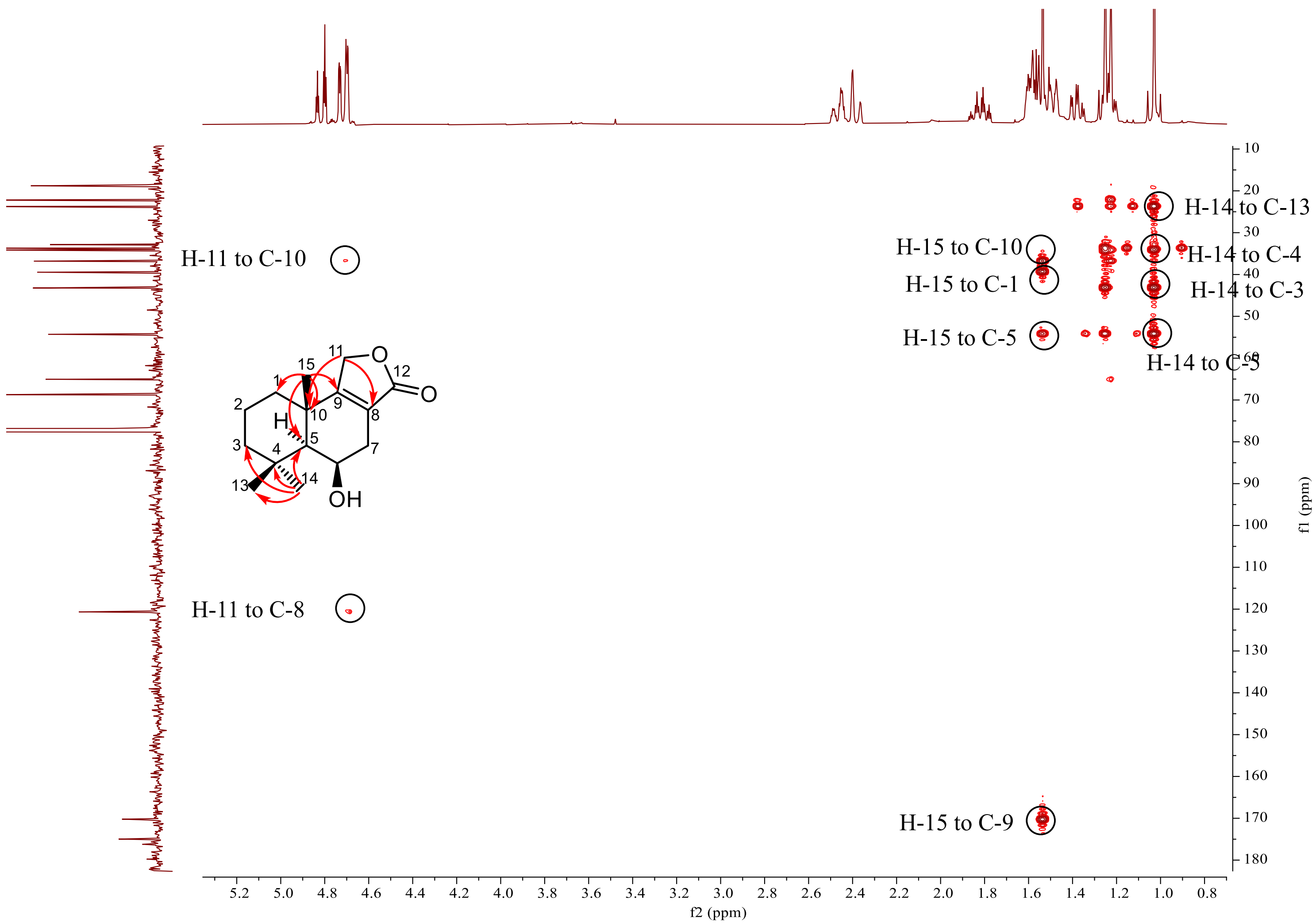

Figure S22. The HMBC spectrum of 6*β*-hydroxyconfertifolin (**5**) in $CDCl_3$.

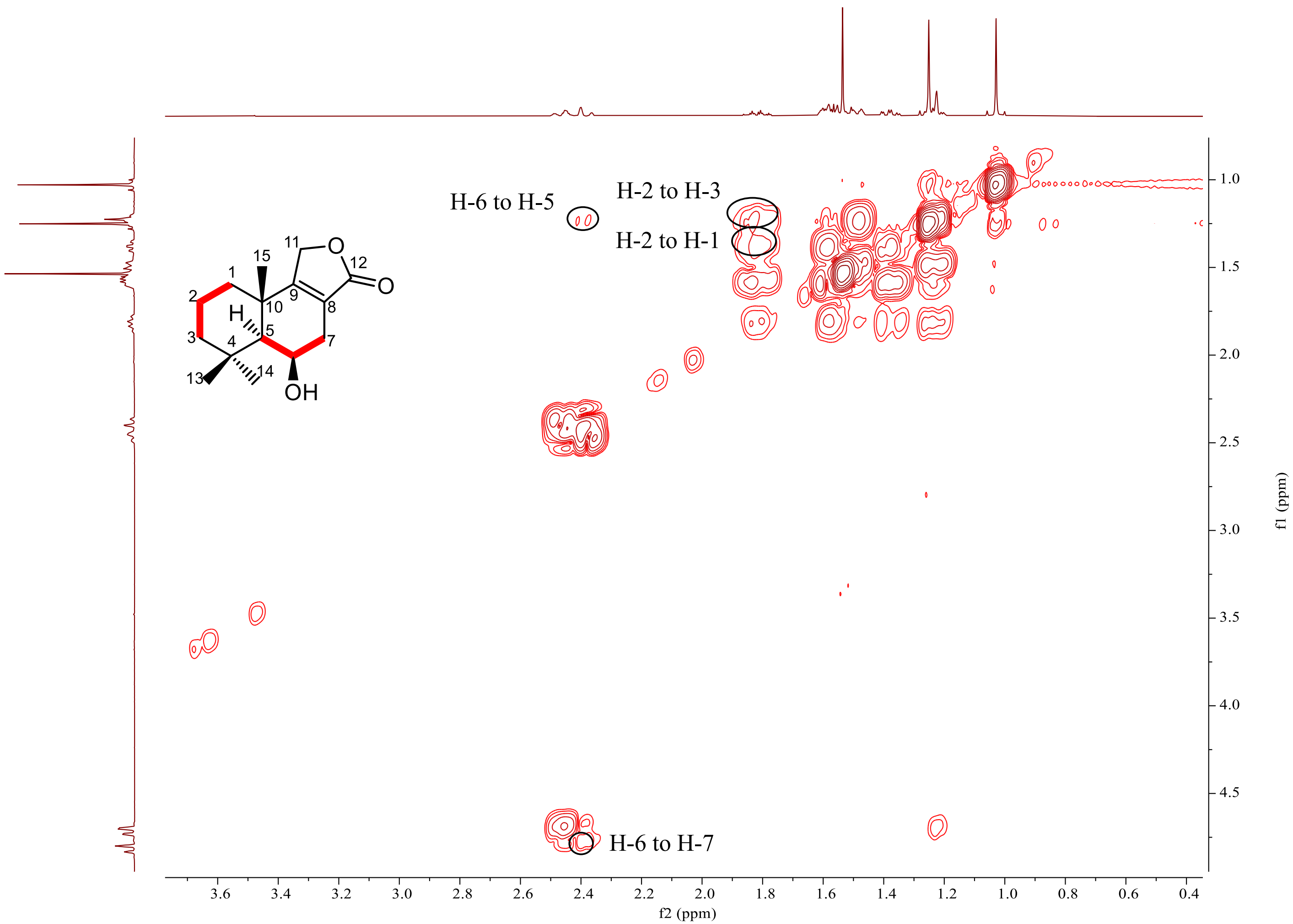

Figure S23. The ^{1}H-^{1}H COSY spectrum of 6*β*-hydroxyconfertifolin (**5**) in $CDCl_3$.

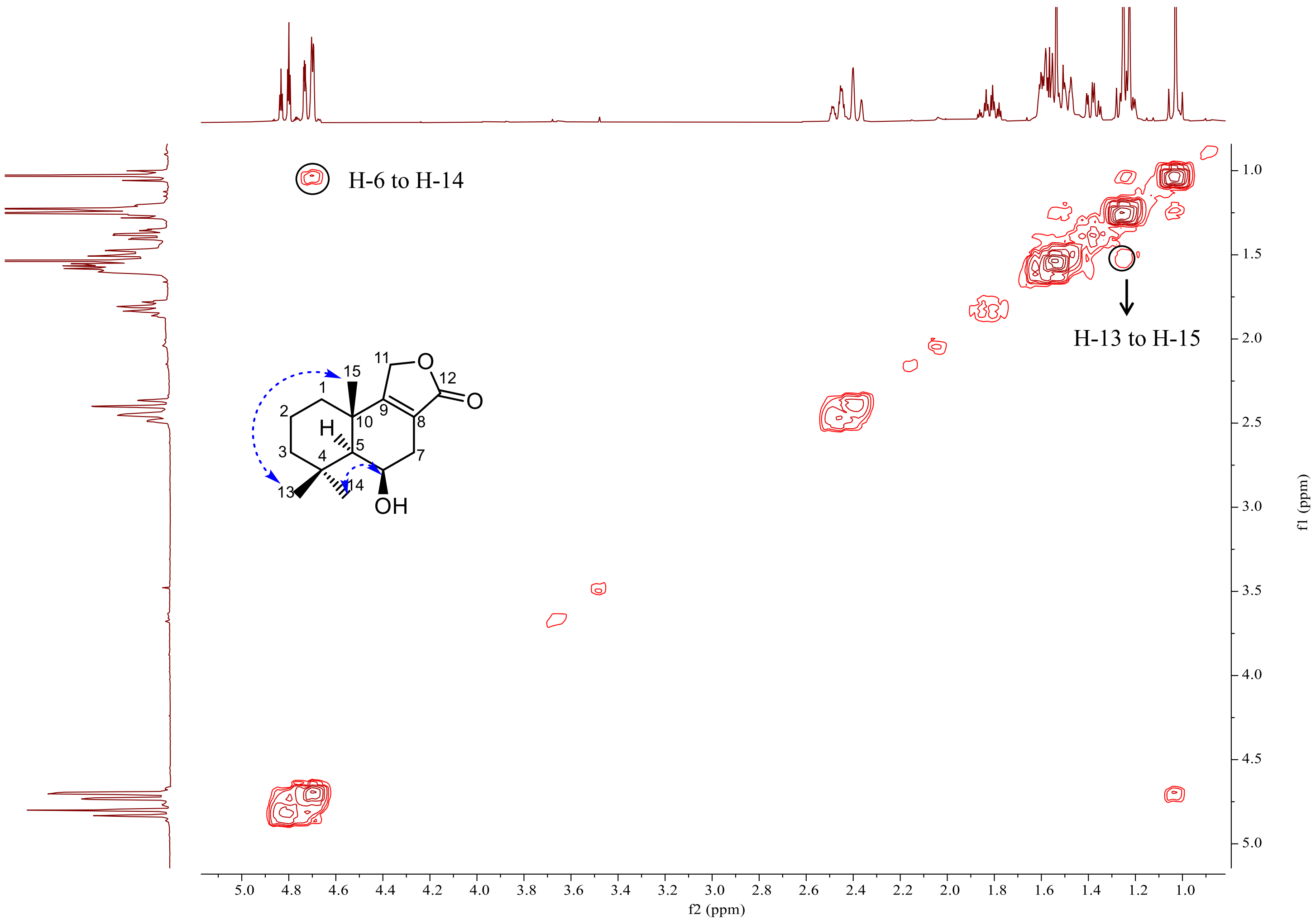

Figure S24. The NOESY spectrum of 6β-hydroxyconfertifolin (**5**) in $CDCl_3$.

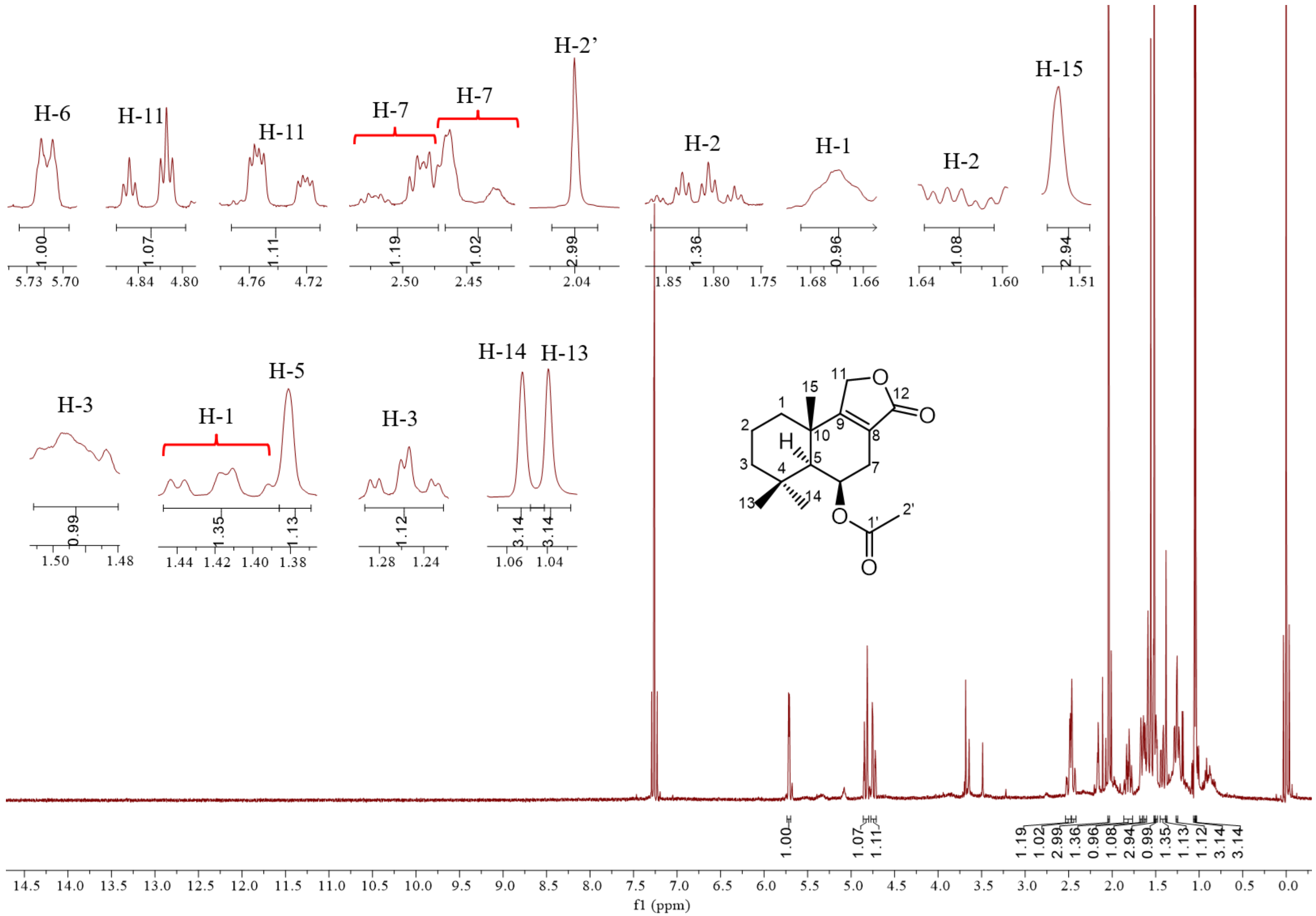

Figure S25. The ^{1}H NMR spectrum of 6*β*-acetoxyconfertifolin (**6**) in $CDCl_3$ (400 MHz).

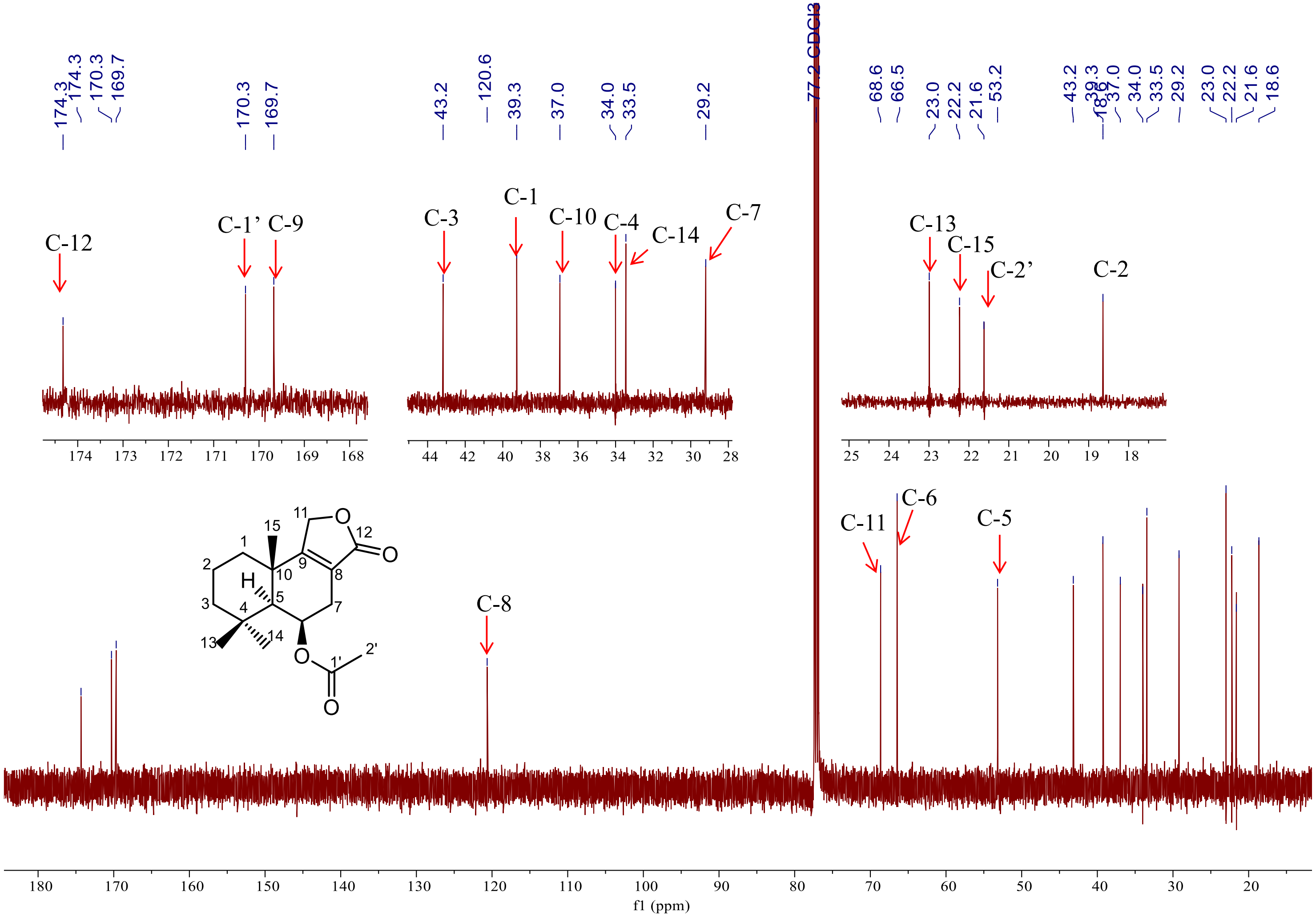

Figure S26. The ^{13}C NMR spectrum of 6*β*-acetoxyconfertifolin (**6**) in $CDCl_3$ (100 MHz).

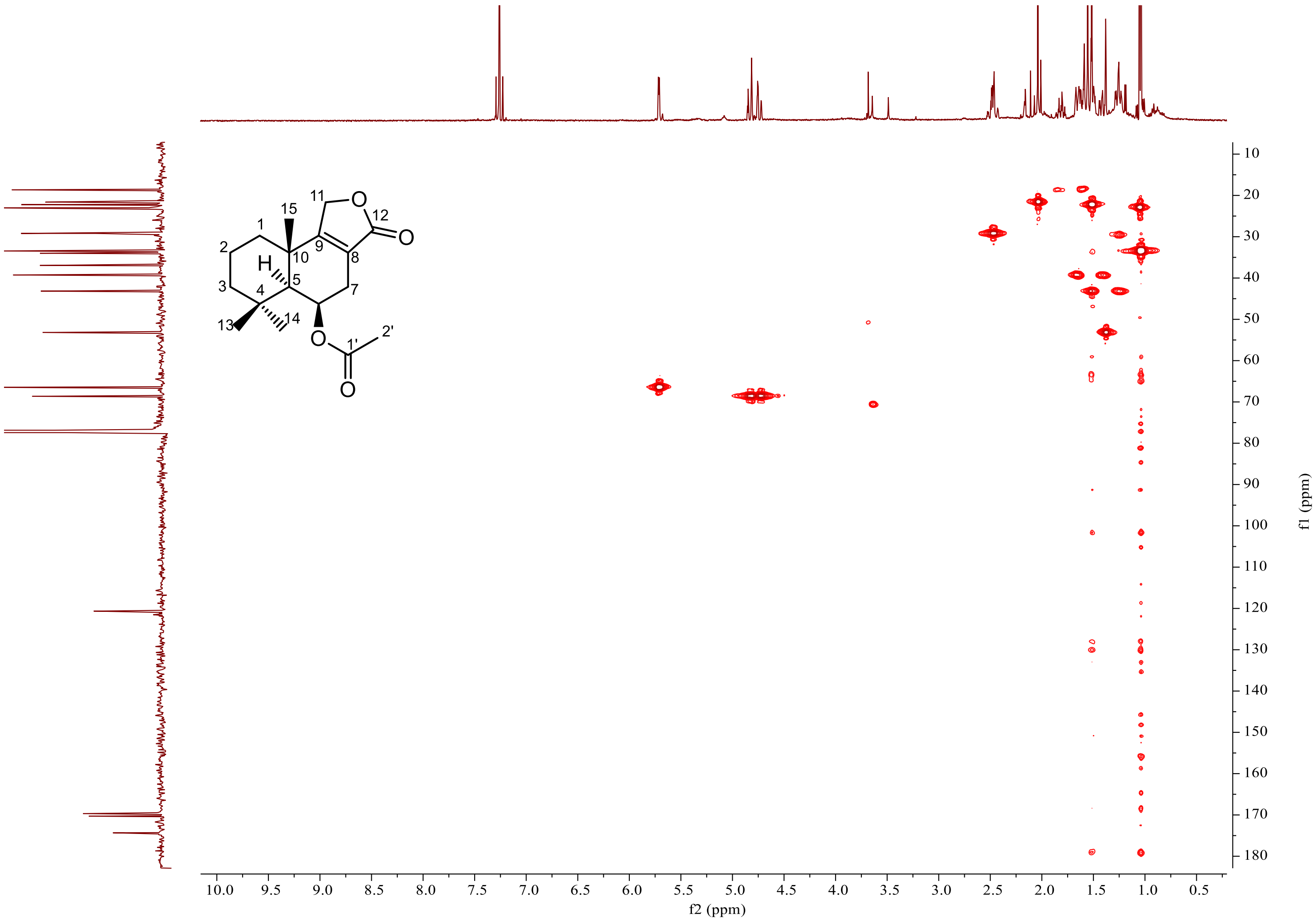

Figure S27. The HSQC spectrum of 6β-acetoxyconfertifolin (**6**) in $CDCl_3$.

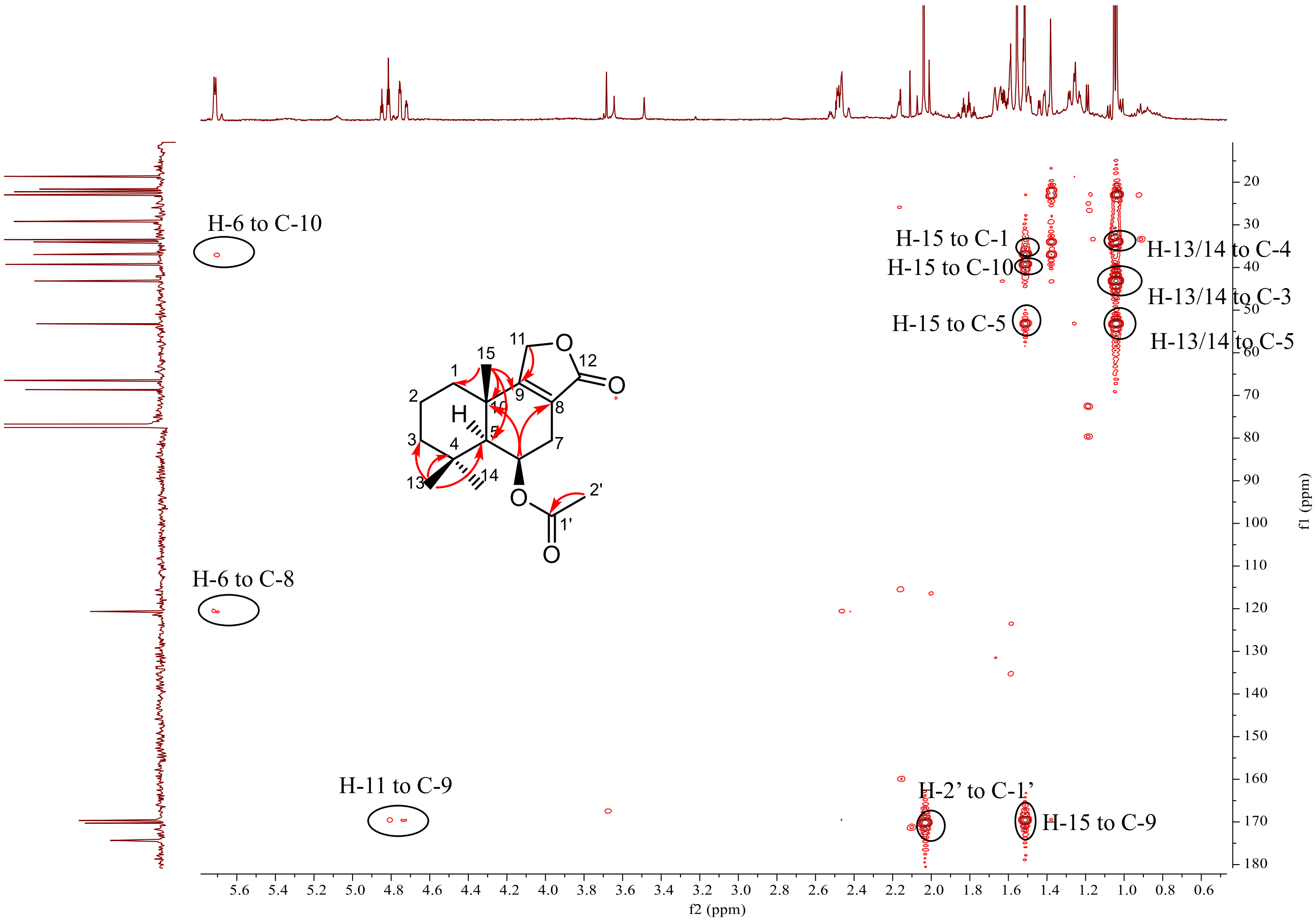

Figure S28. The HMBC spectrum of 6*β*-acetoxyconfertifolin (**6**) in $CDCl_3$.

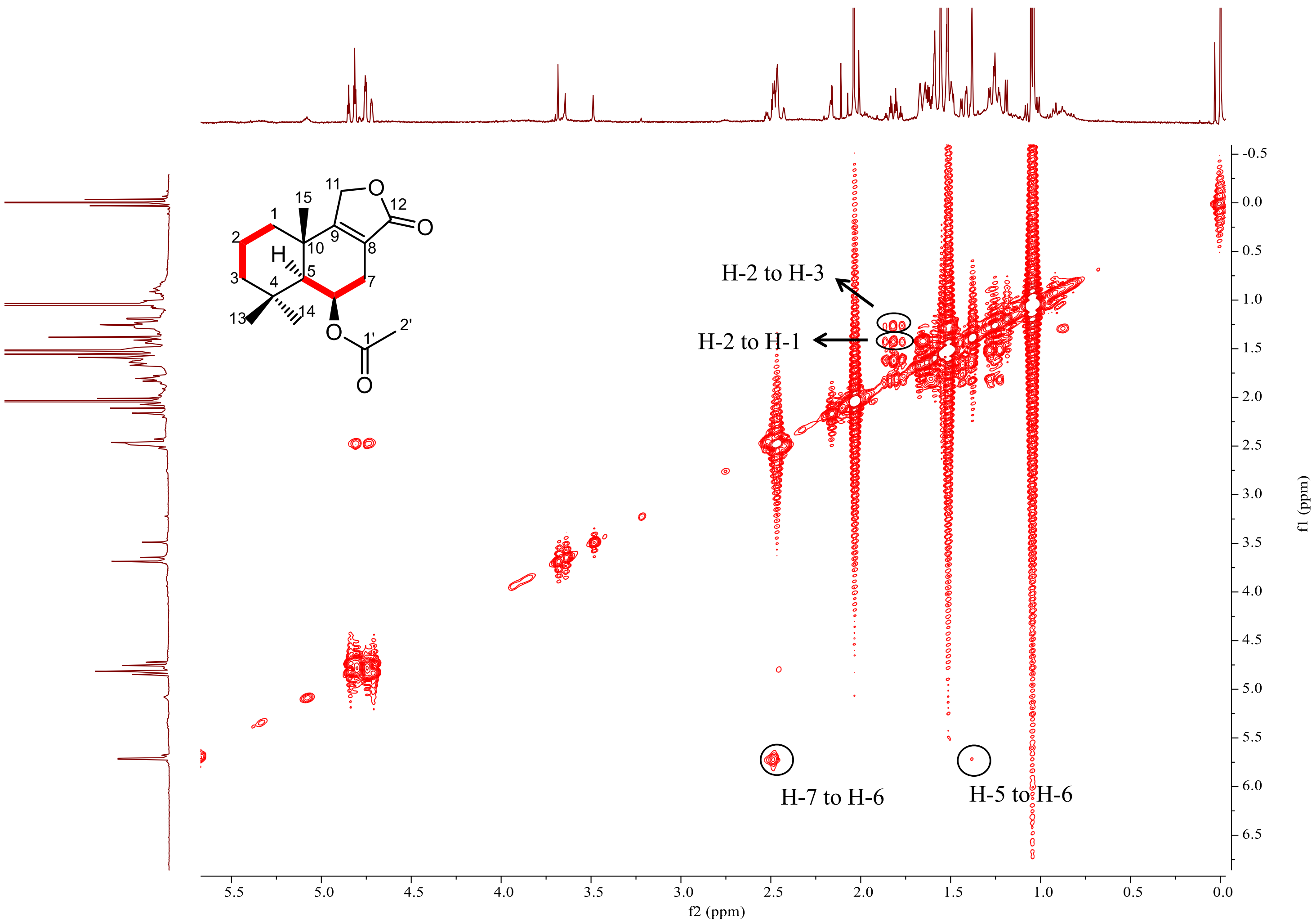

Figure S29. The ^{1}H-^{1}H COSY spectrum of 6β-acetoxyconfertifolin (**6**) in $CDCl_3$.

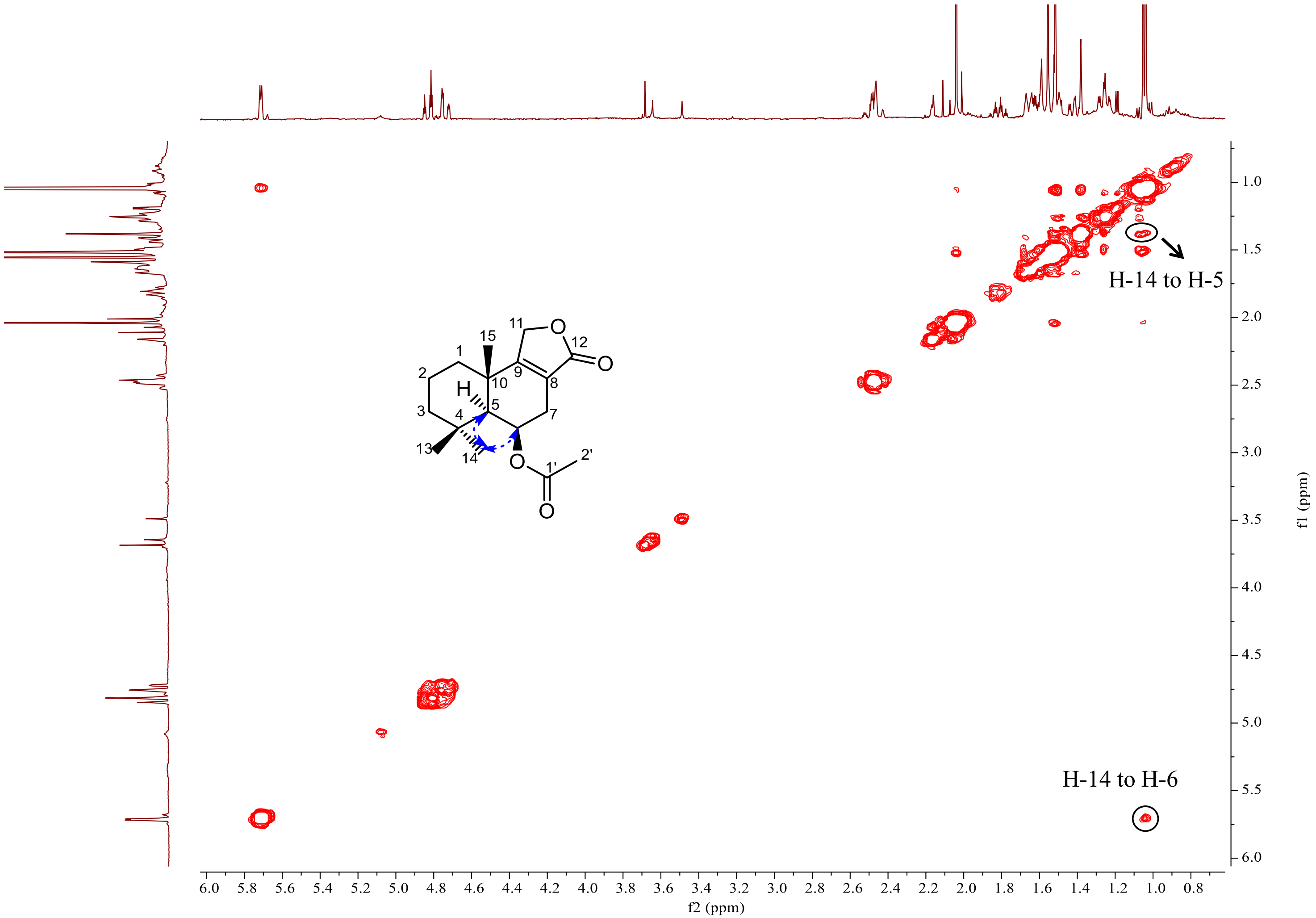

Figure S30. The NOESY spectrum of 6*β*-acetoxyconfertifolin (**6**) in $CDCl_3$.

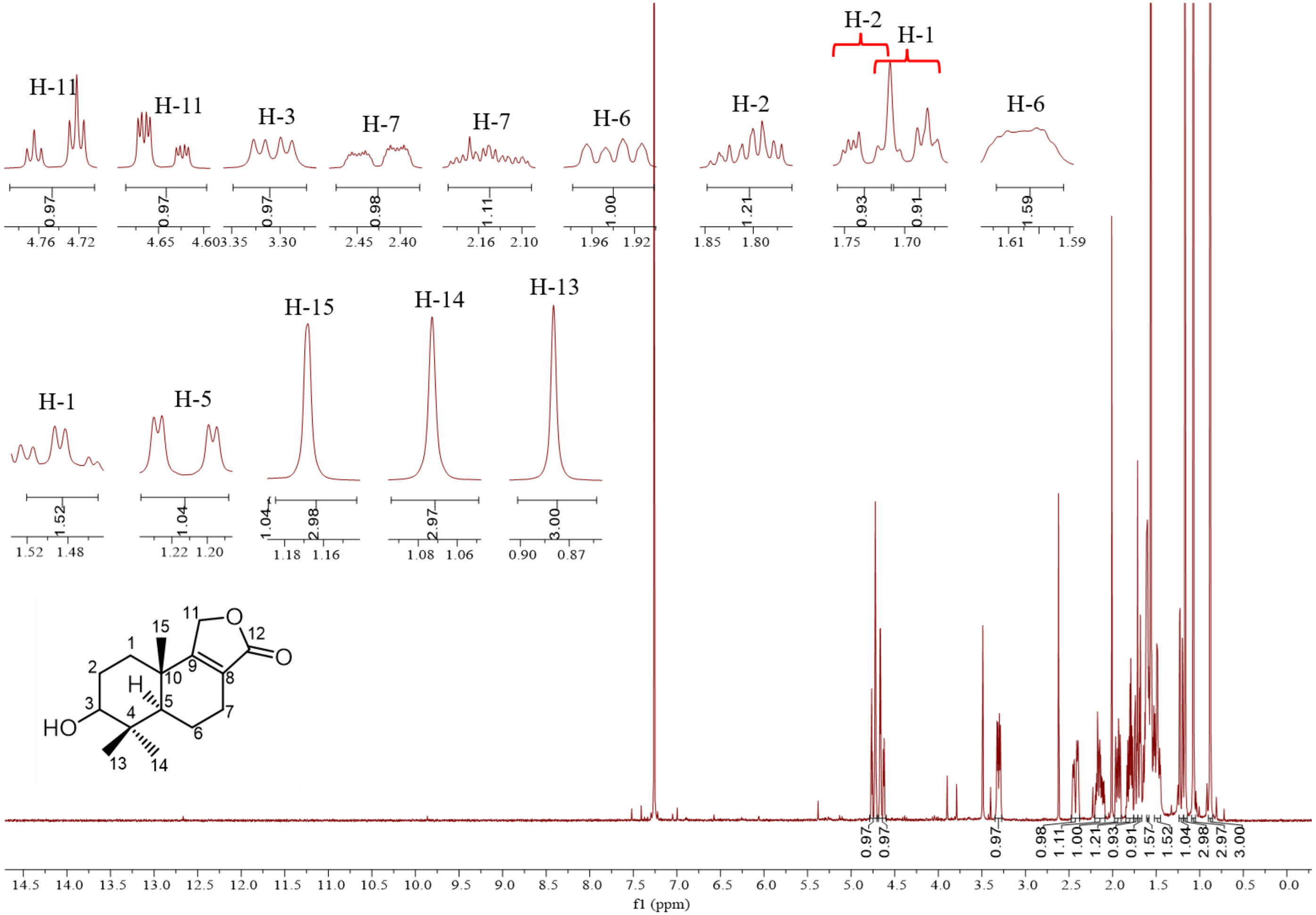

Figure S31. The ^{1}H NMR spectrum of 3*β*-hydroxyconfertifolin (**7**) in $CDCl_3$ (400 MHz).

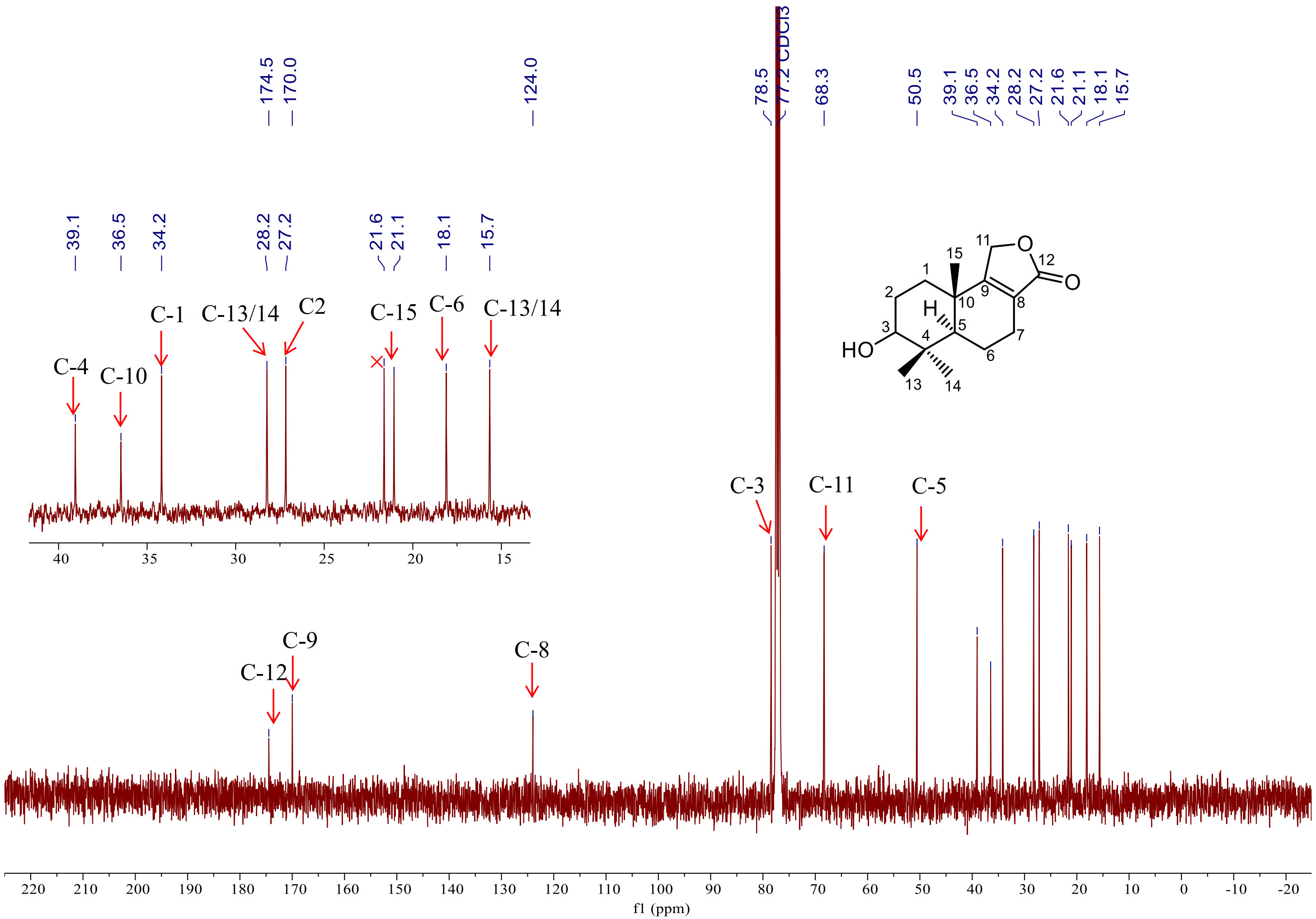

Figure S32. The ^{13}C NMR spectrum of 3*β*-hydroxyconfertifolin (**7**) in $CDCl_3$ (100 MHz).

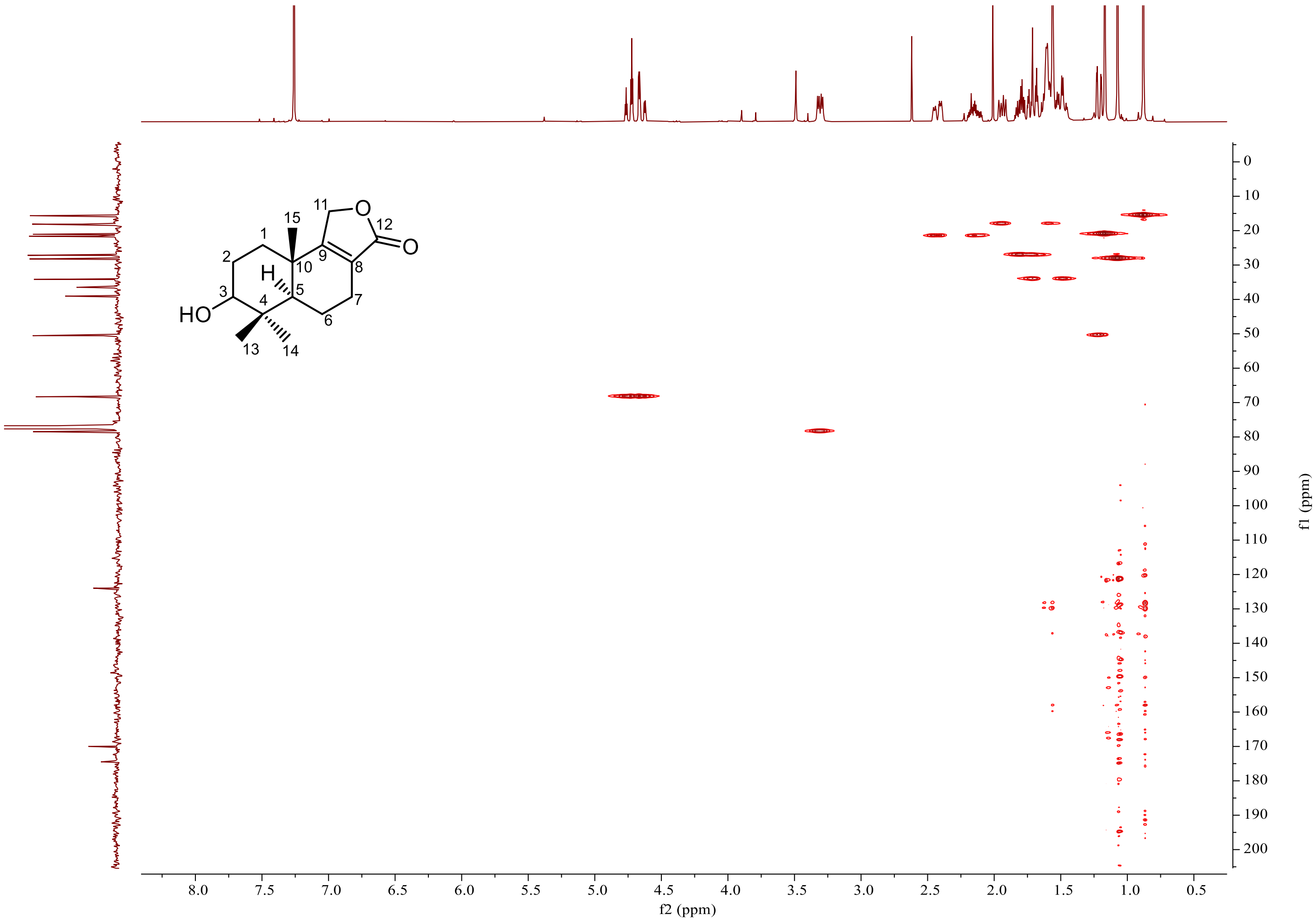

Figure S33. The HSQC spectrum of 3*β*-hydroxyconfertifolin (**7**) in $CDCl_3$.

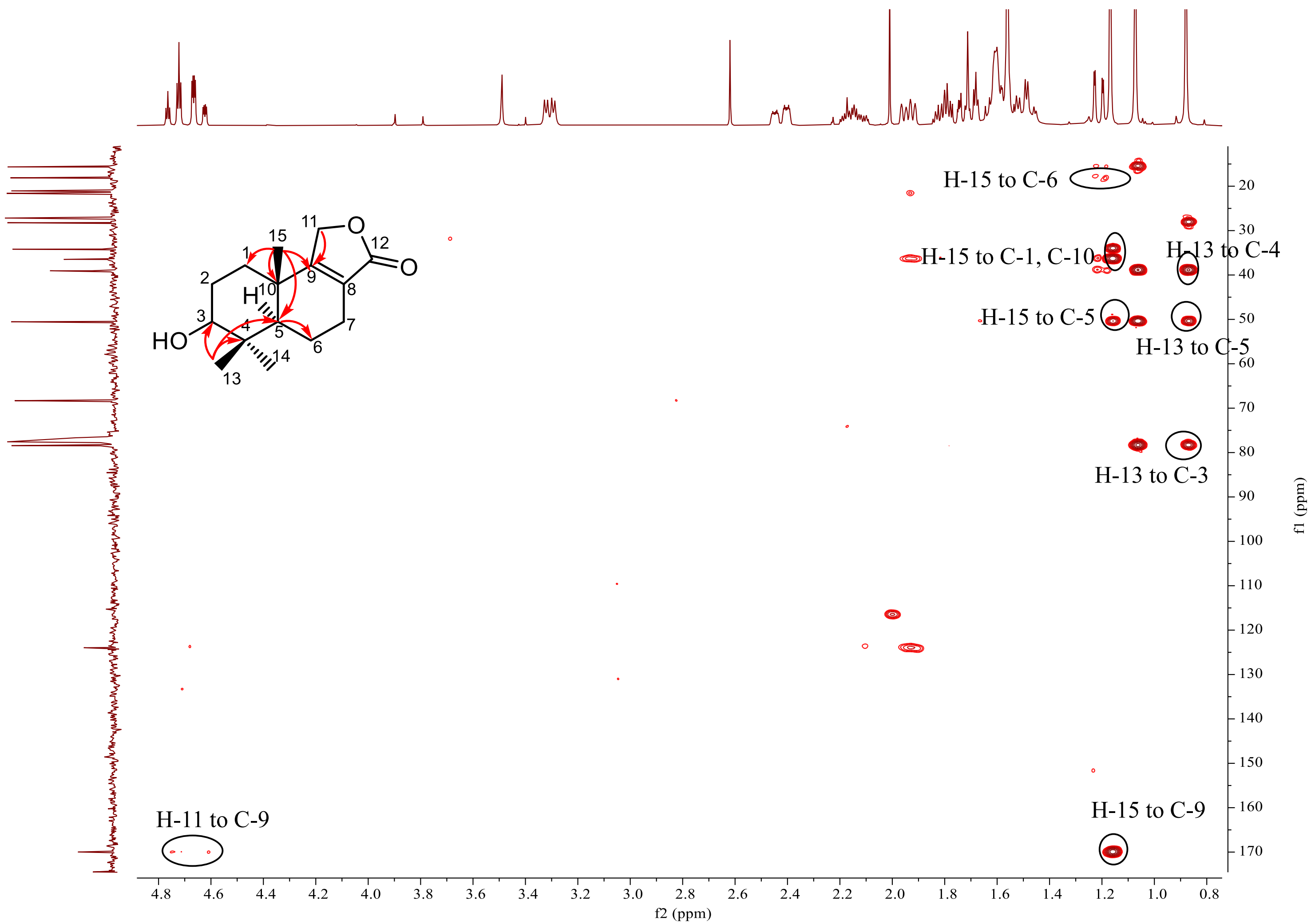

Figure S34. The HMBC spectrum of 3*β*-hydroxyconfertifolin (**7**) in $CDCl_3$.

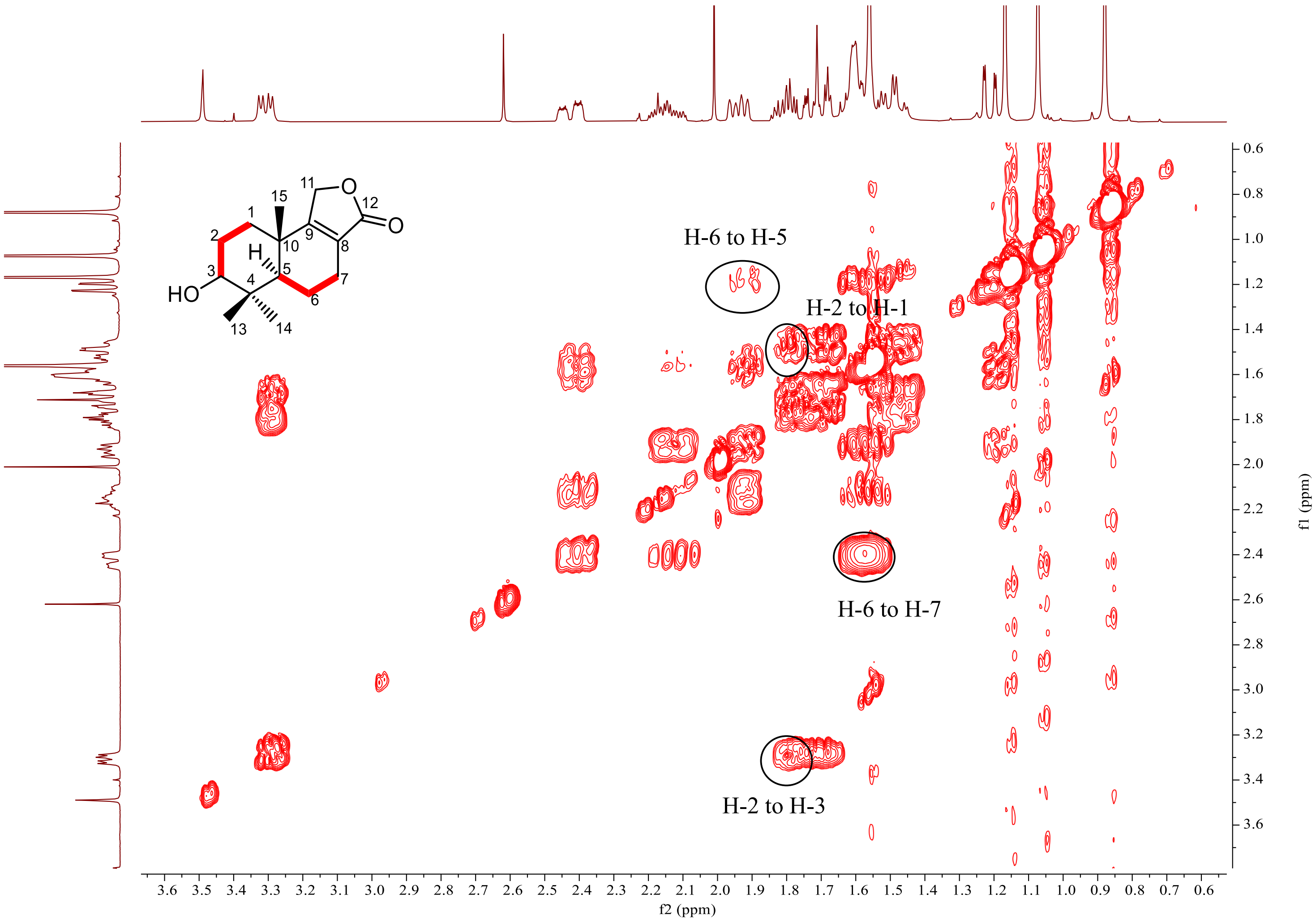

Figure S35. The ^{1}H-^{1}H COSY spectrum of 3*β*-hydroxyconfertifolin (**7**) in $CDCl_3$.

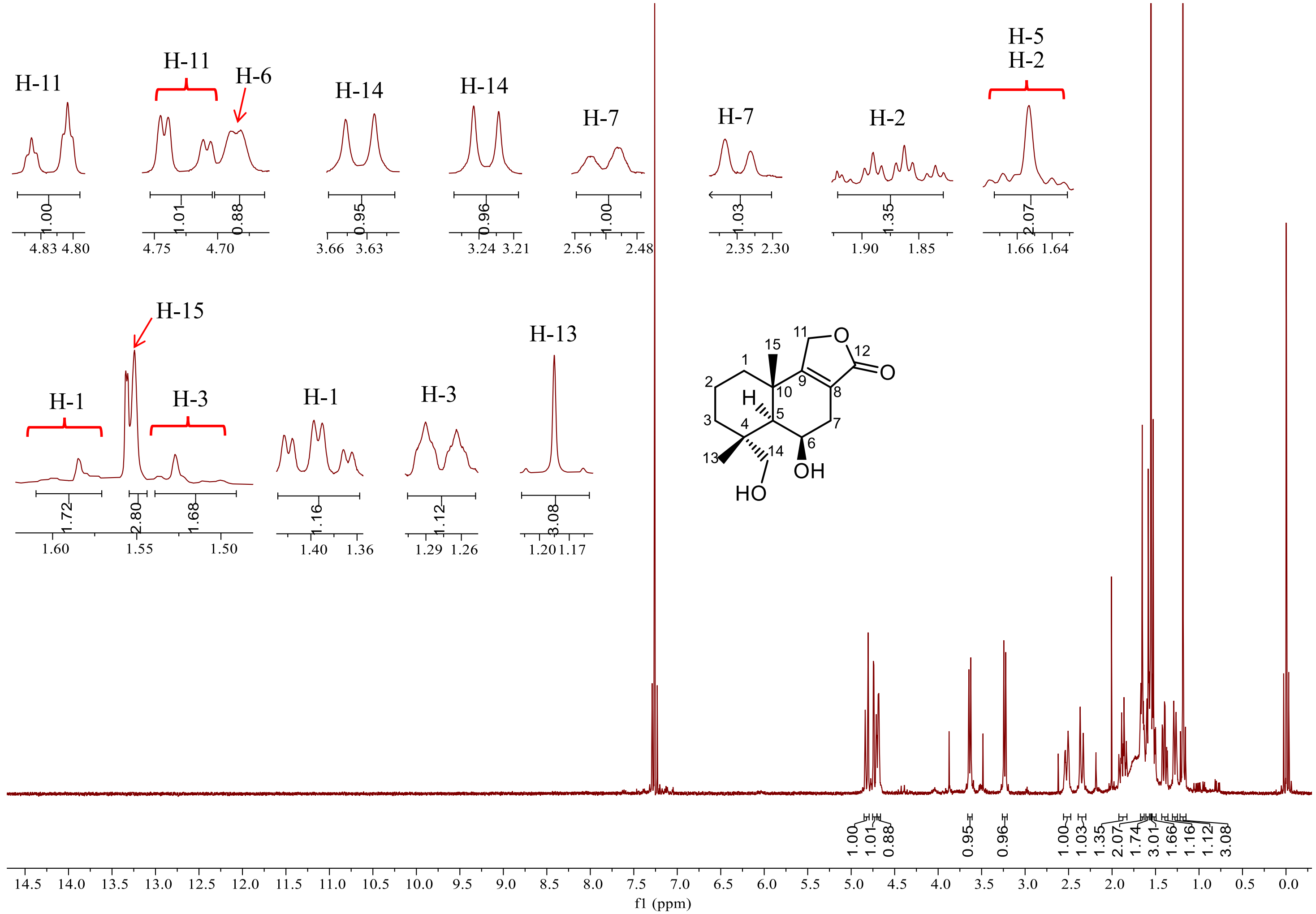

Figure S36. The ^{1}H NMR spectrum of ustidrimane B (**8**) in $CDCl_3$ (400 MHz).

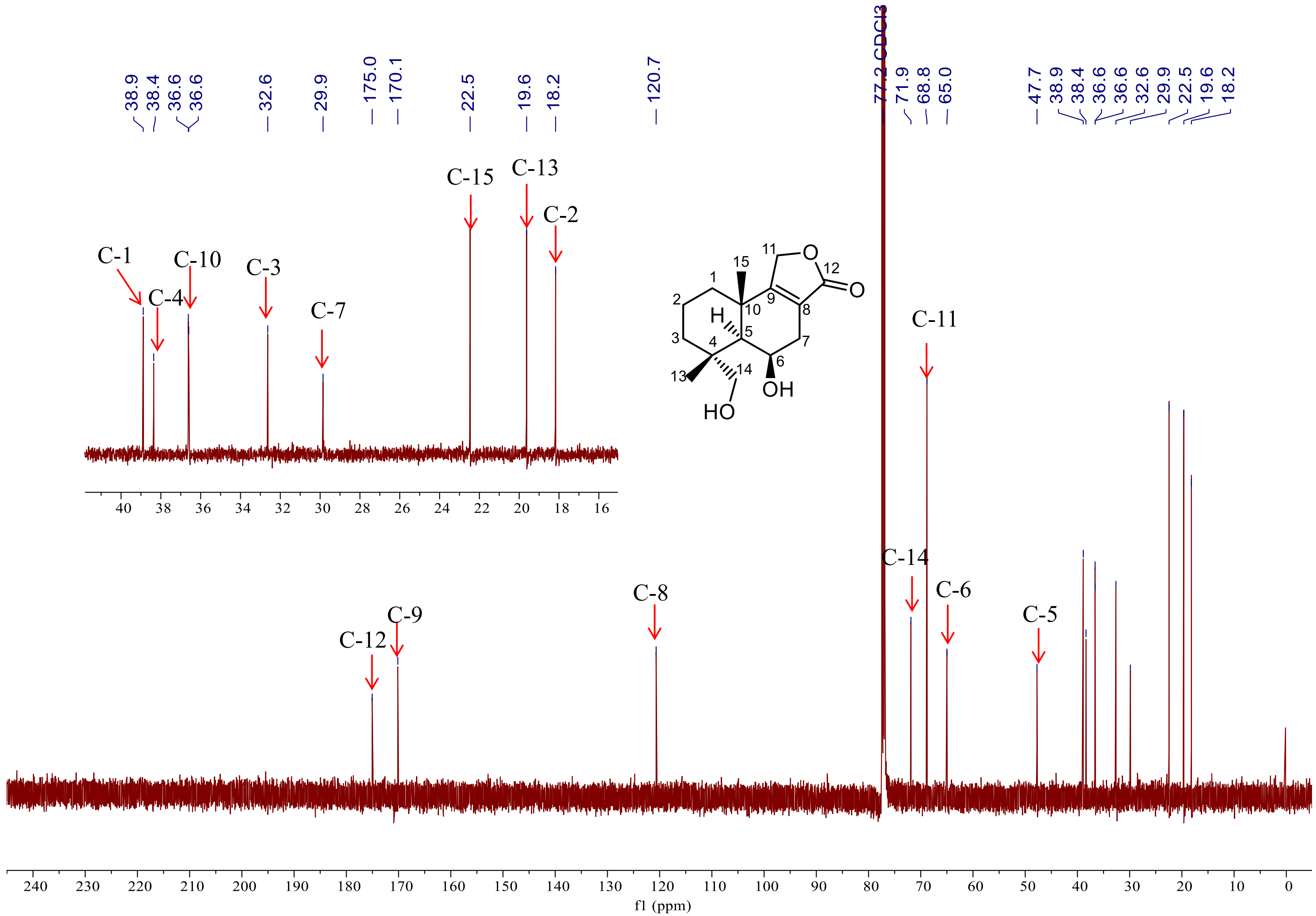

Figure S37. The ^{13}C NMR spectrum of ustidrimane B (**8**) in $CDCl_3$ (100 MHz).

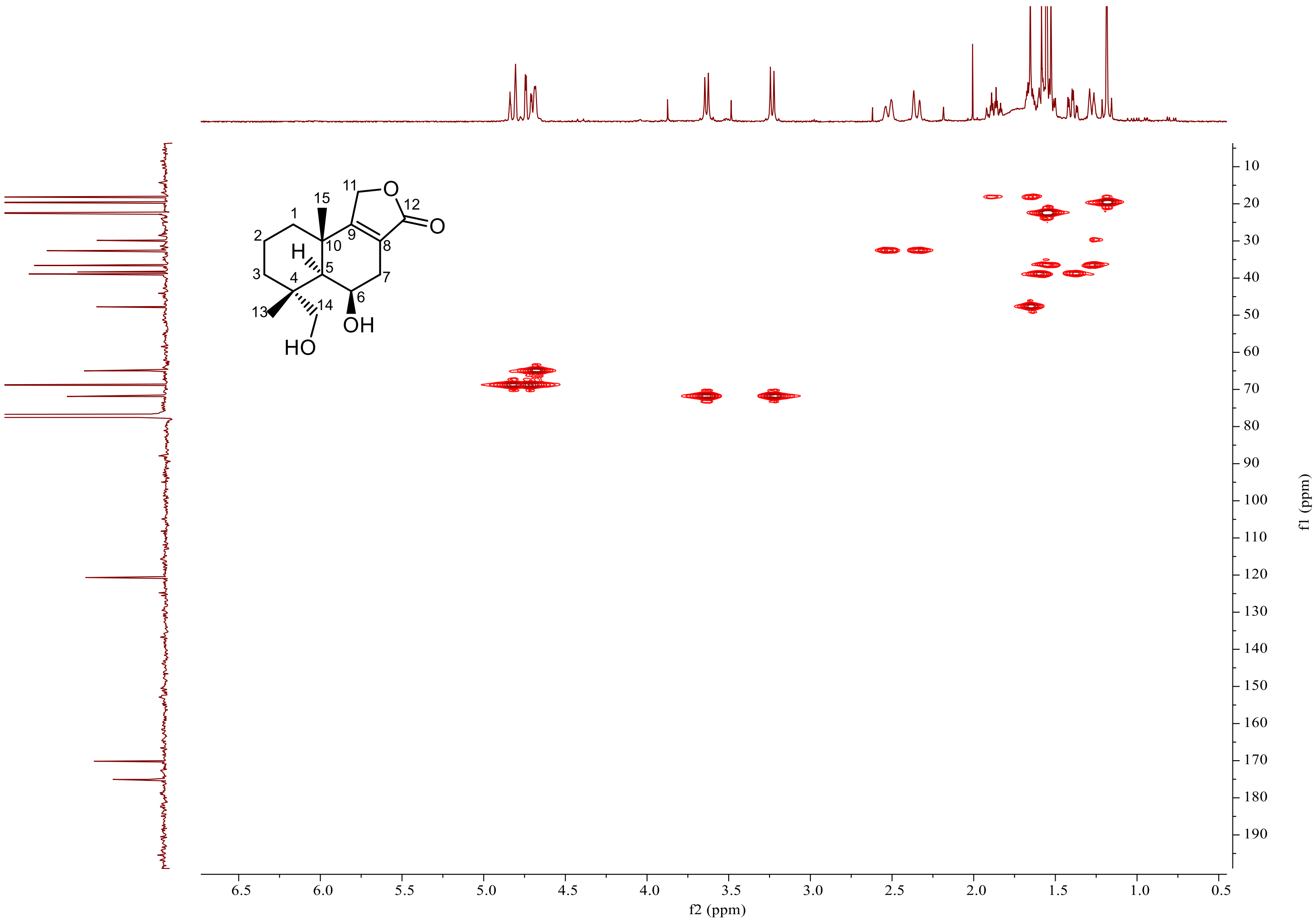

Figure S38. The HSQC spectrum of ustidrimane B (**8**) in $CDCl_3$.

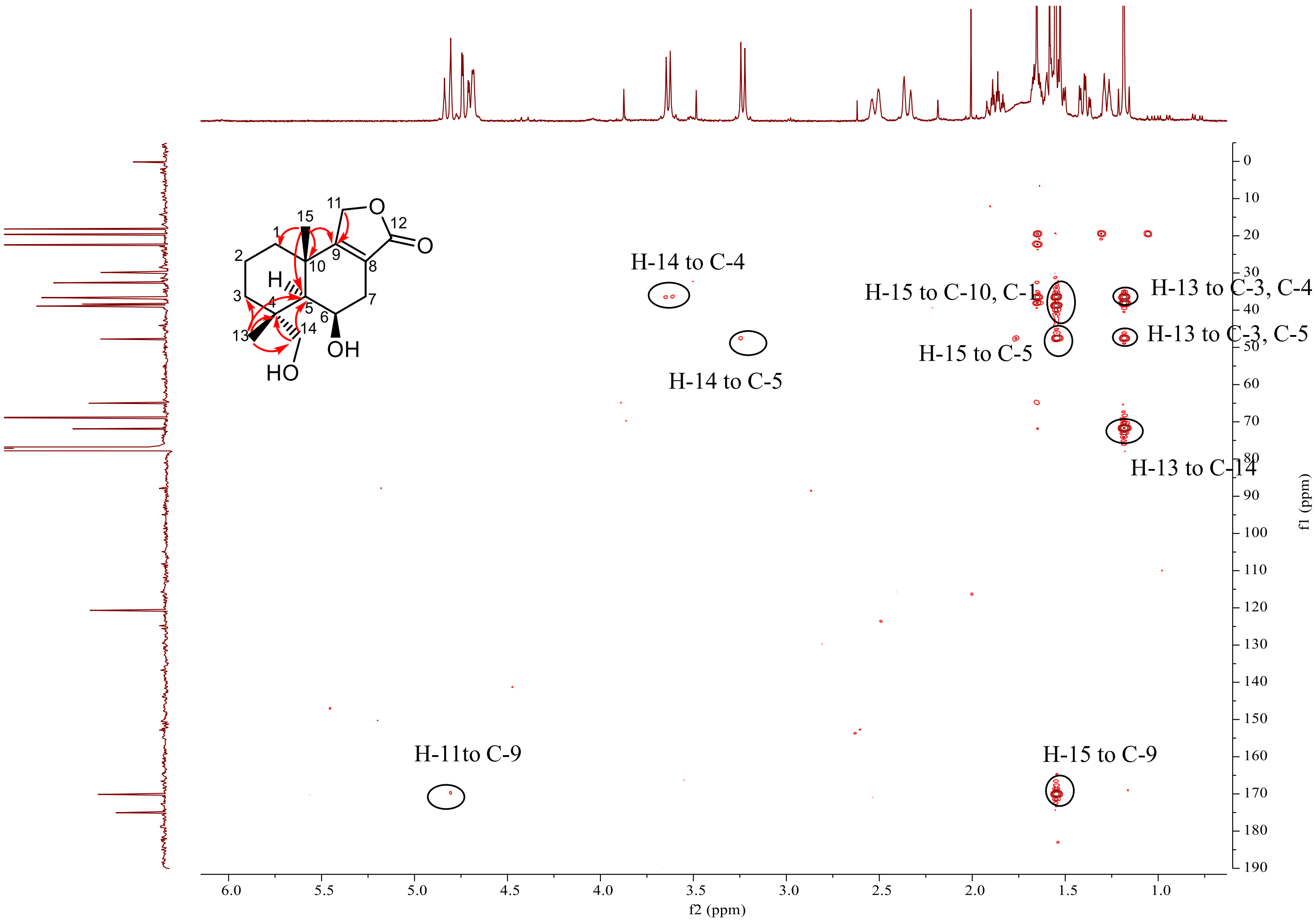

Figure S39. The HMBC spectrum of ustidrimane B (**8**) in $CDCl_3$.

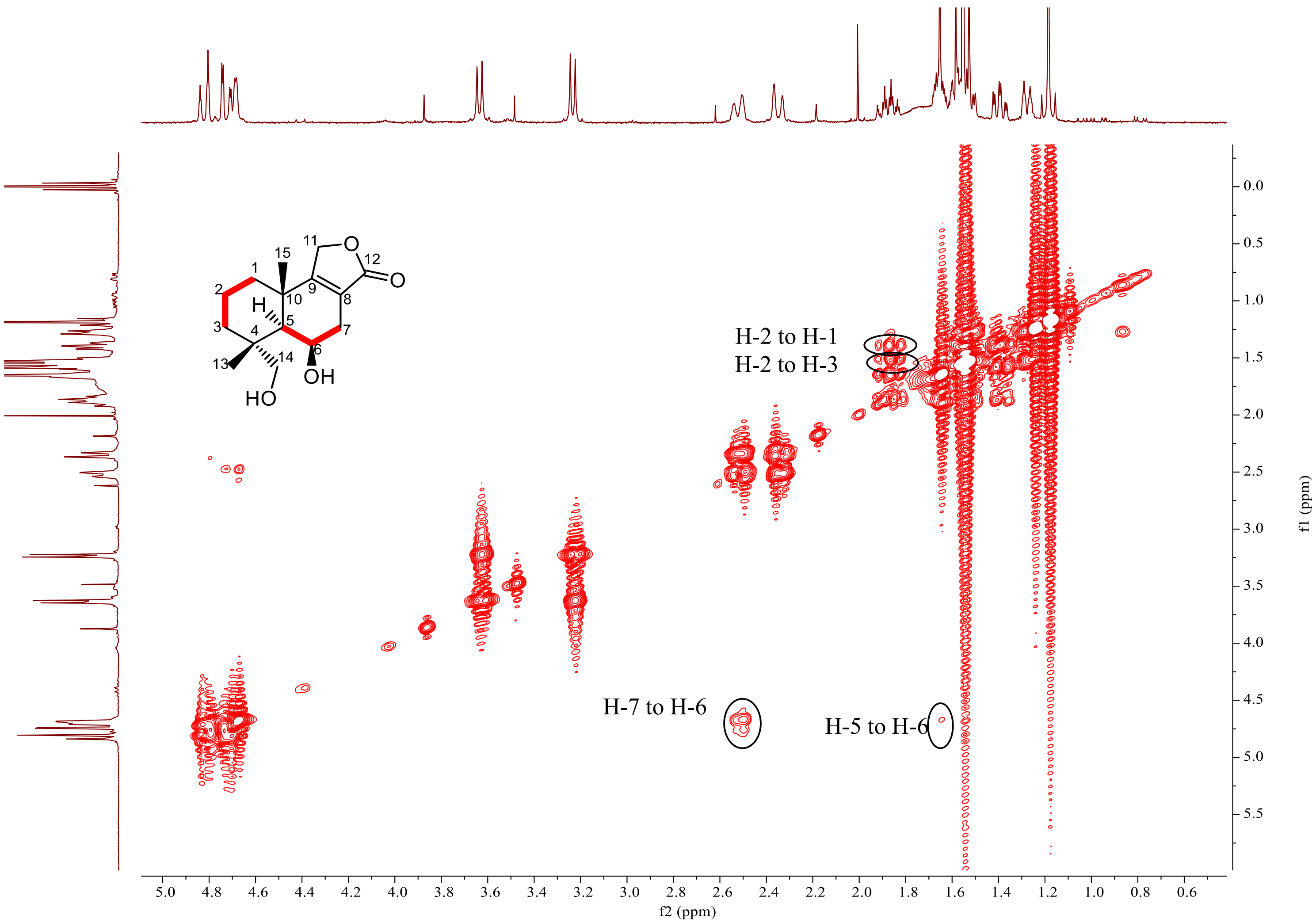

Figure S40. The ^{1}H-^{1}H COSY spectrum of ustidrimane B (**8**) in $CDCl_3$.

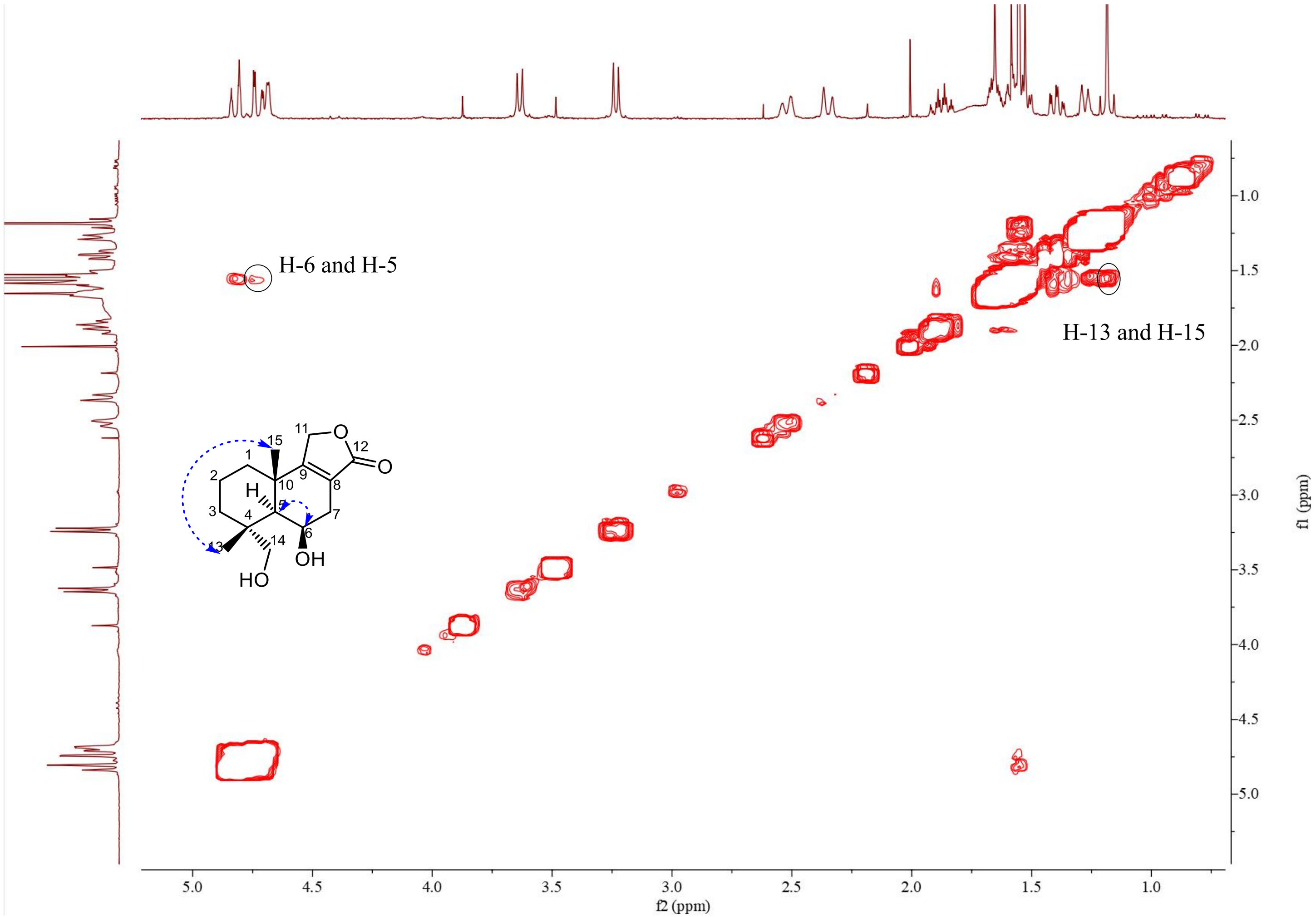

Figure S41. The NOESY spectrum of ustidrimane B (**8**) in $CDCl_3$.

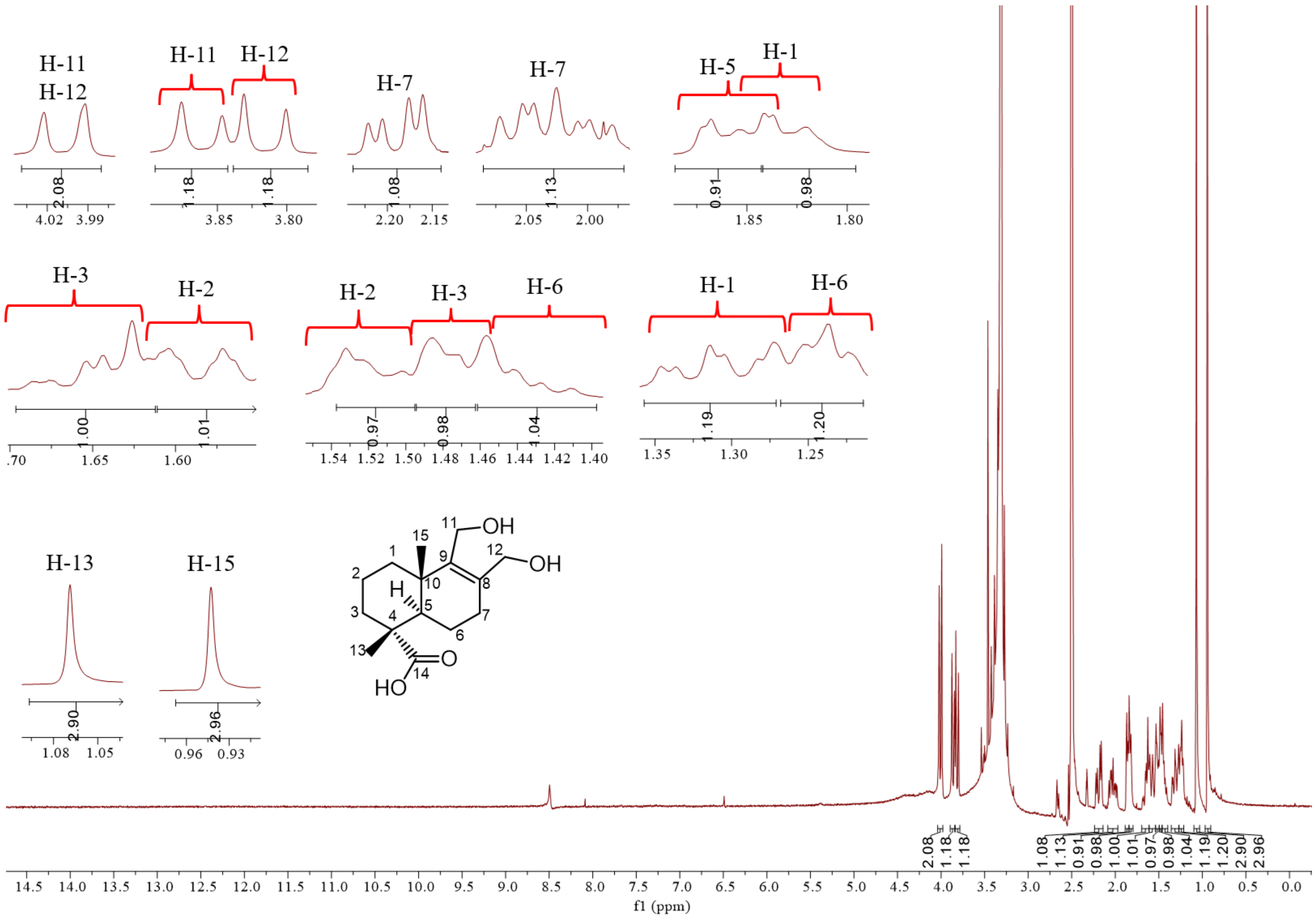

Figure S42. The ^{1}H NMR spectrum of ustidrimane C (**9**) in DMSO-d_6 (400 MHz).

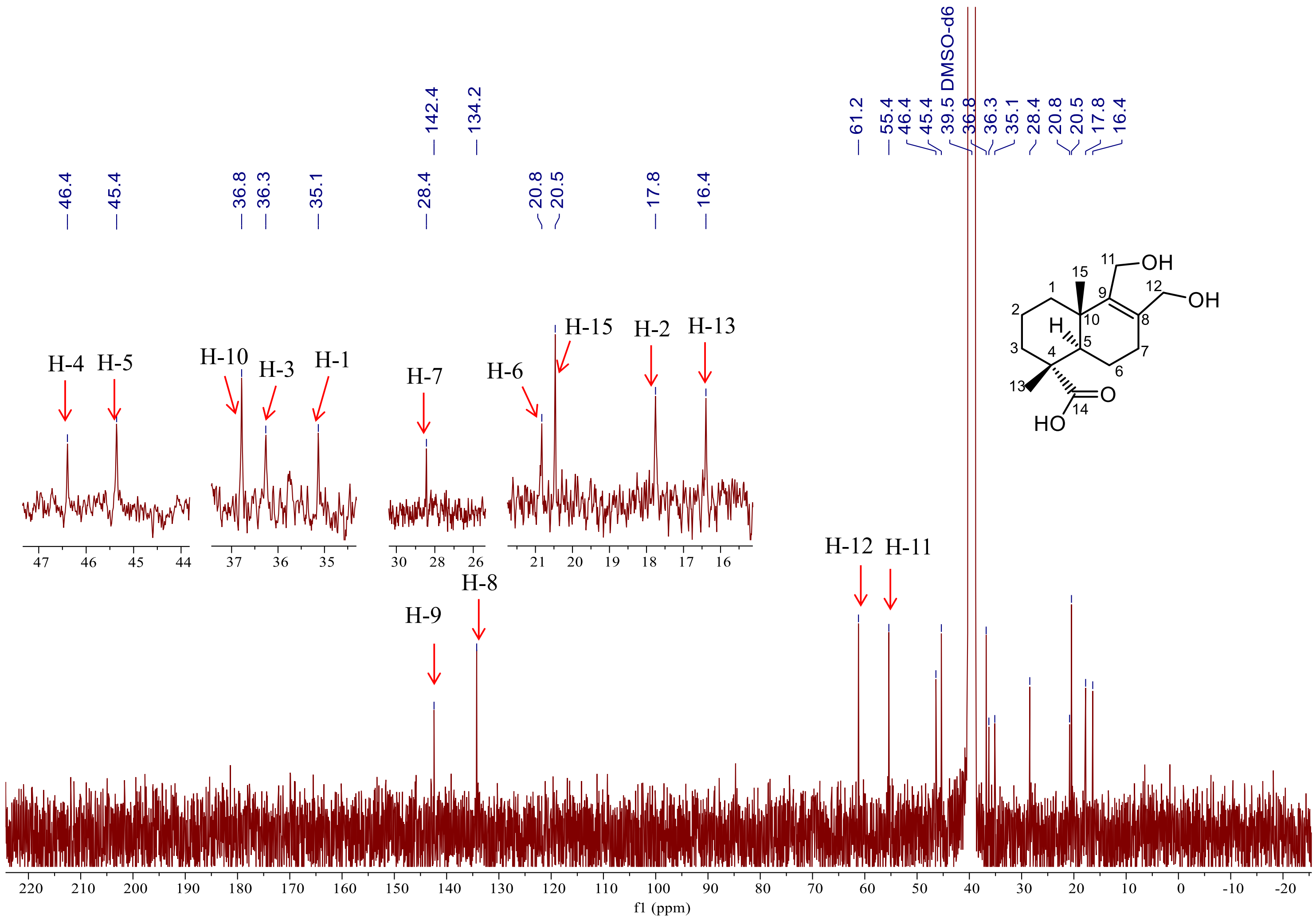

Figure S43. The ^{13}C NMR spectrum of ustidrimane C (**9**) in DMSO-d_6 (100 MHz).

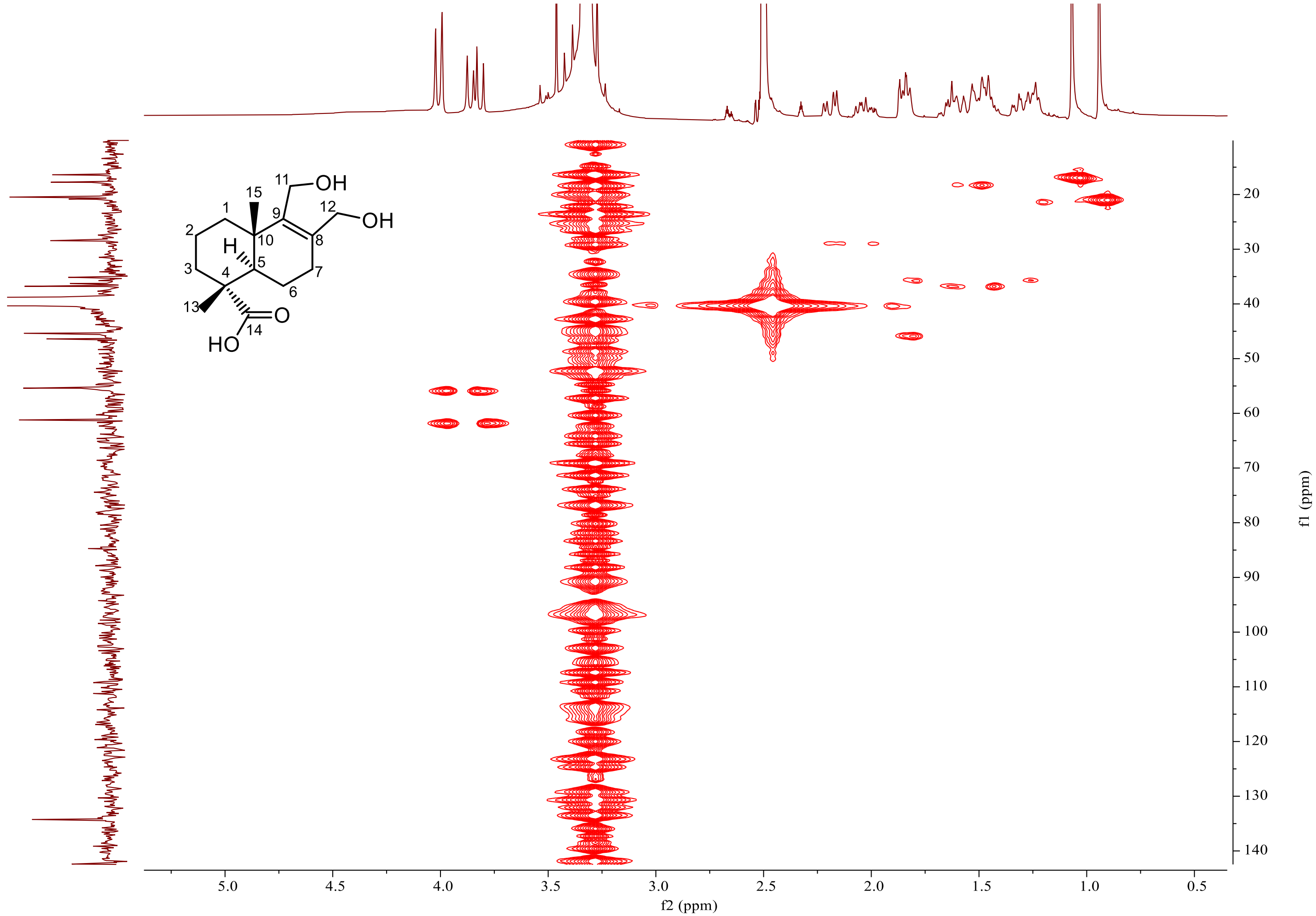

Figure S44. The HSQC spectrum of ustidrimane C (**9**) in DMSO-d_6.

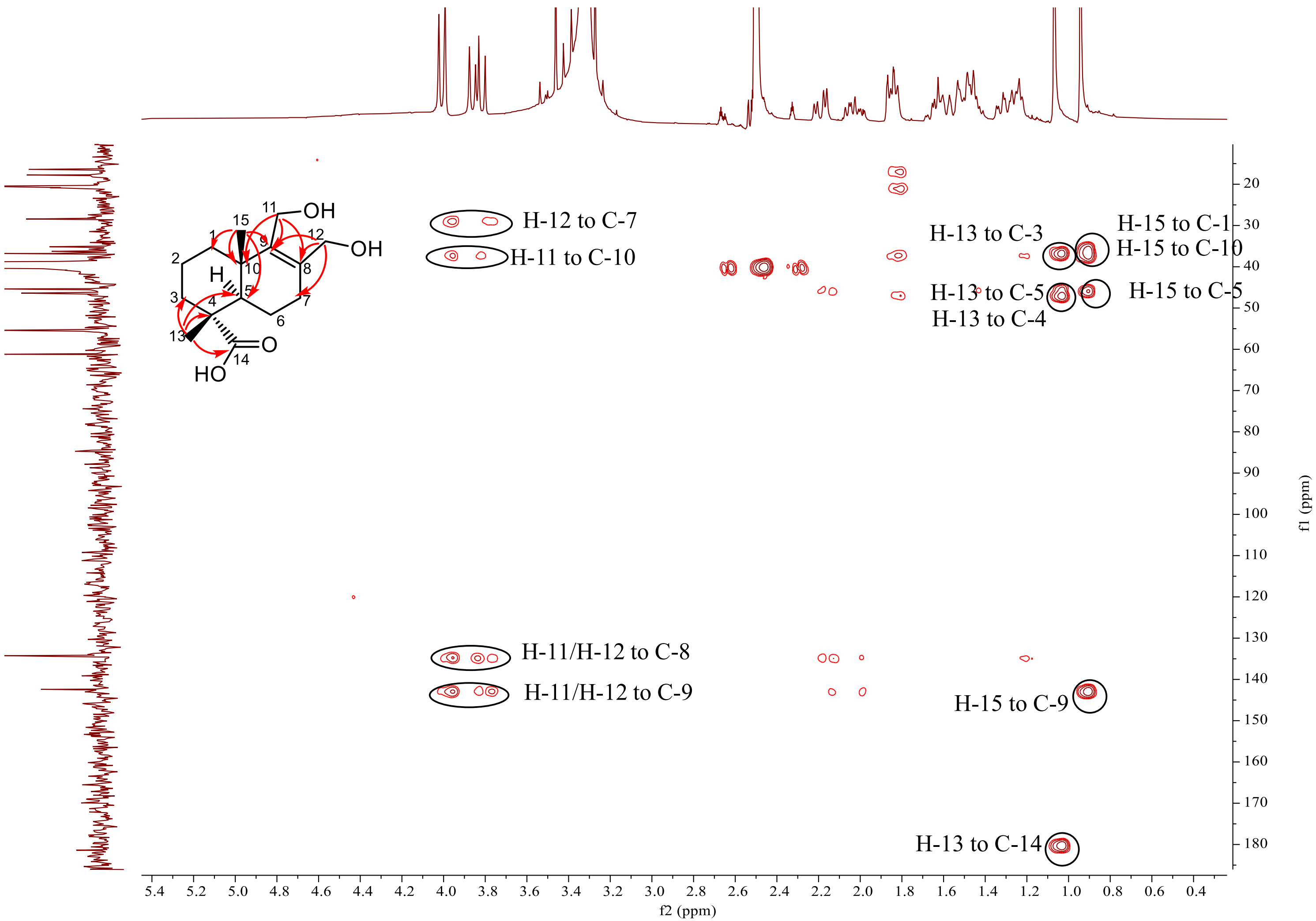

Figure S45. The HMBC spectrum of ustidrimane C (**9**) in DMSO-d_6.

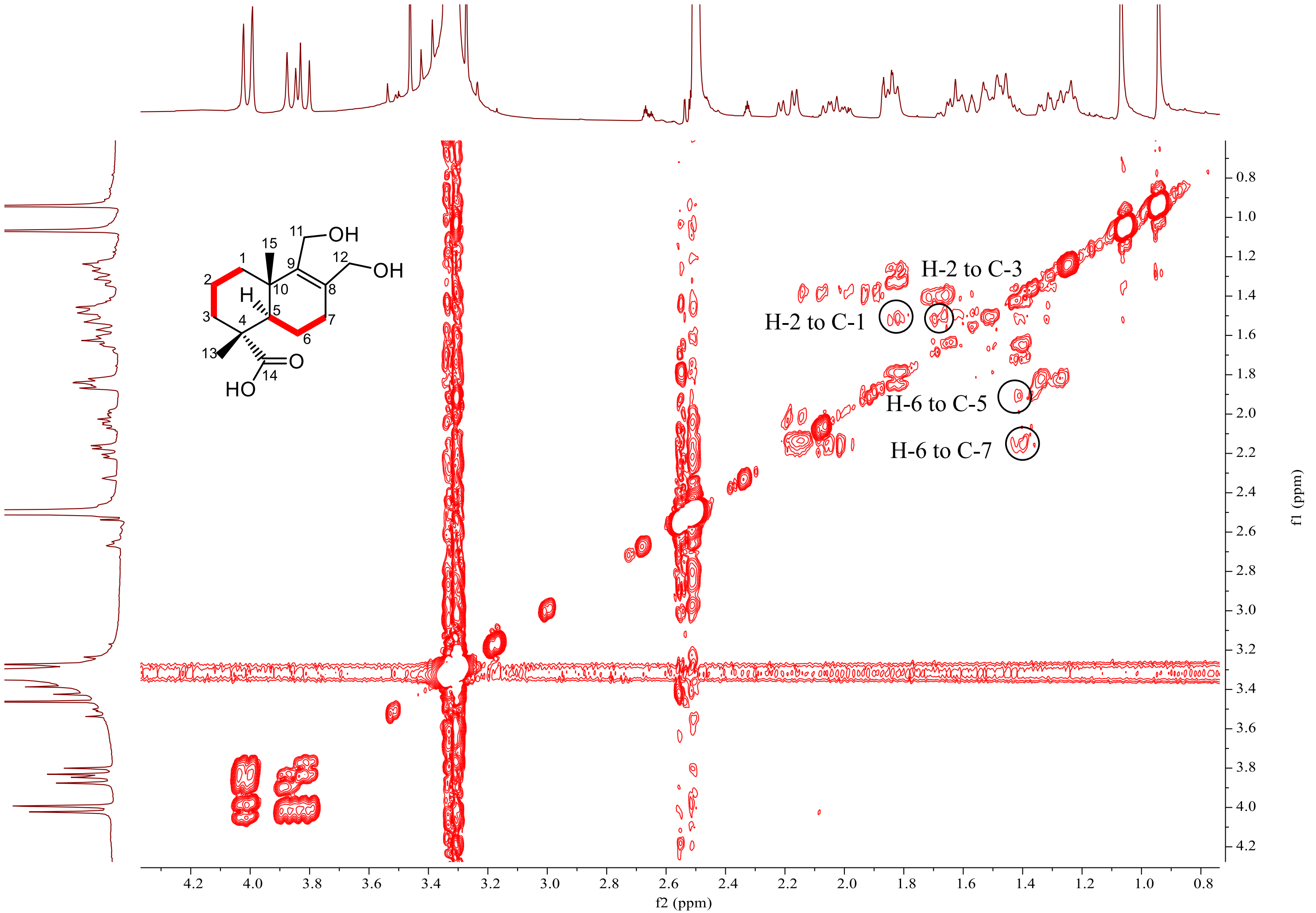

Figure S46. The ^{1}H-^{1}H COSY spectrum of ustidrimane C (**9**) in DMSO-d_6.

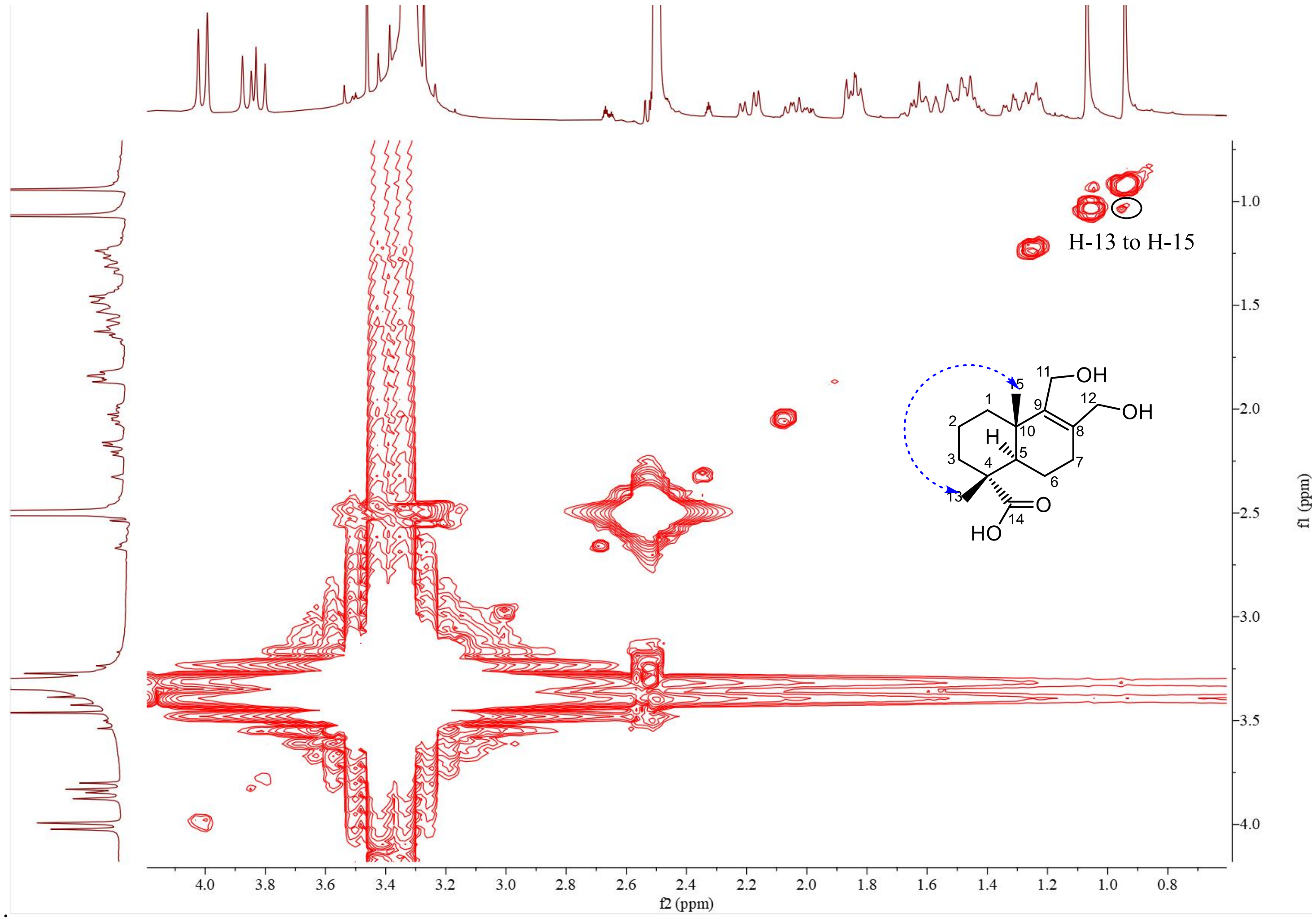

Figure S47. The NOESY spectrum of ustidrimane C (**9**) in DMSO-d_6.

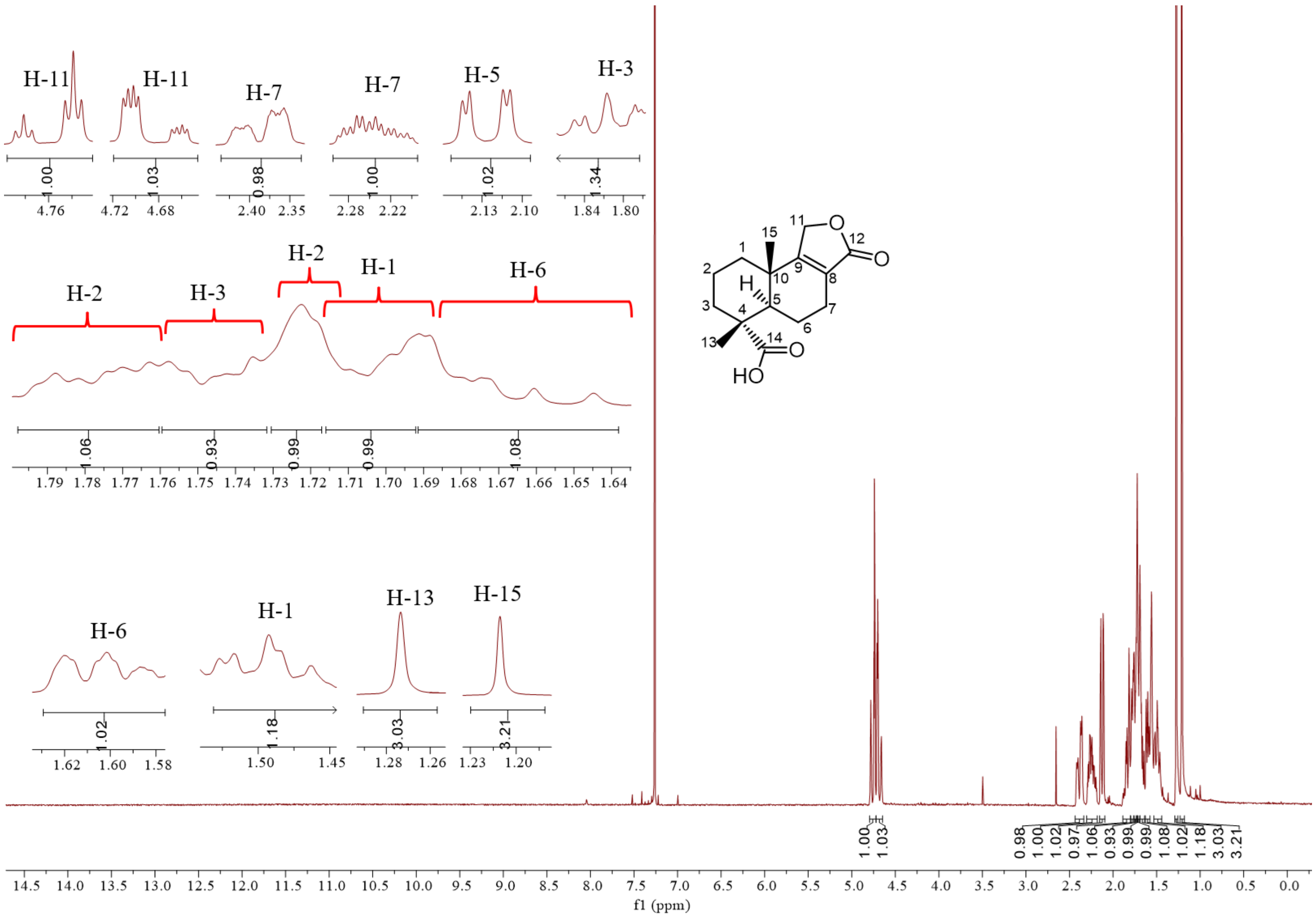

Figure S48. The ^{1}H NMR spectrum of ustidrimane D (**10**) in $CDCl_3$ (400 MHz).

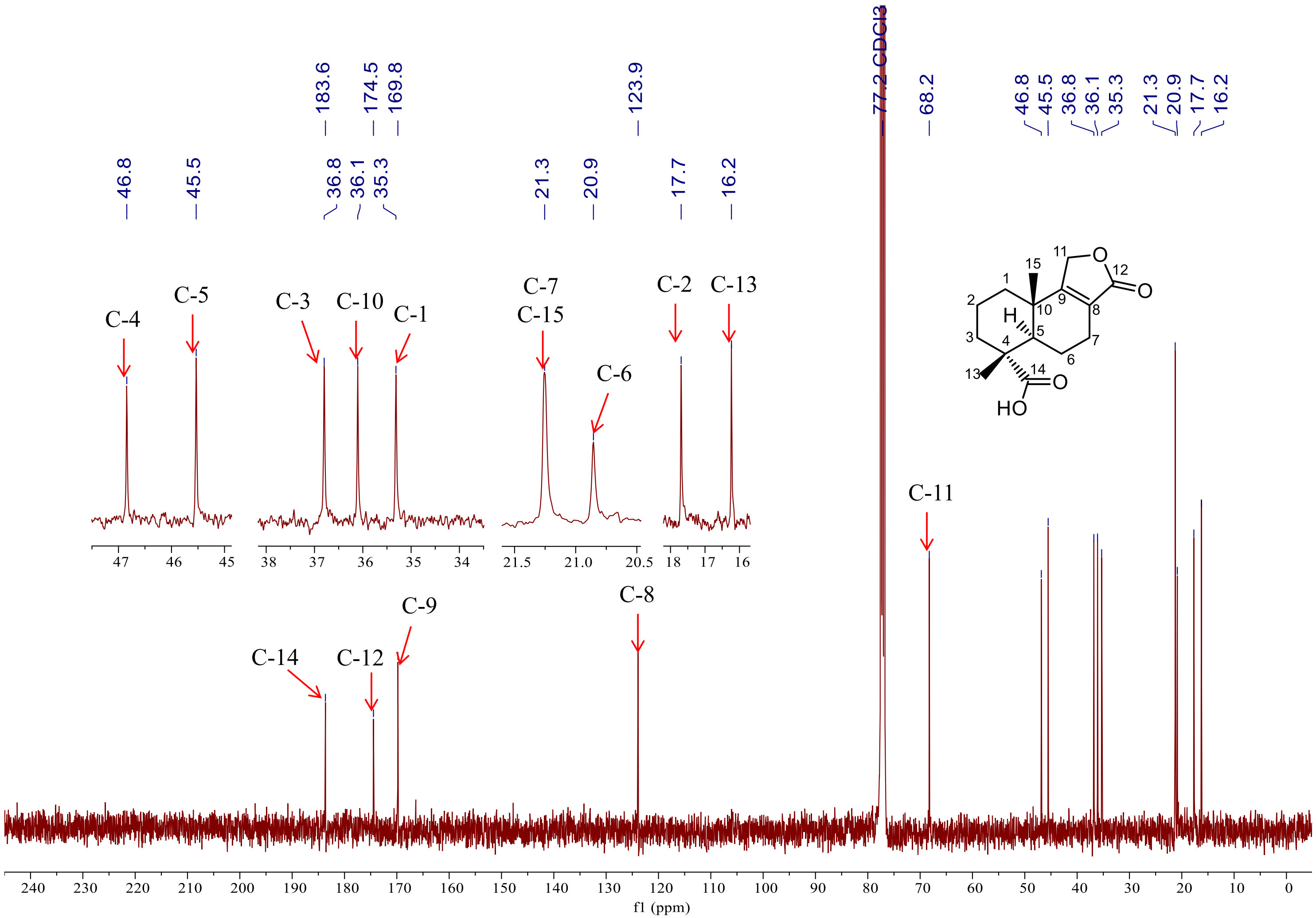

Figure S49. The ^{13}C NMR spectrum of ustidrimane D (**10**) in $CDCl_3$ (100 MHz).

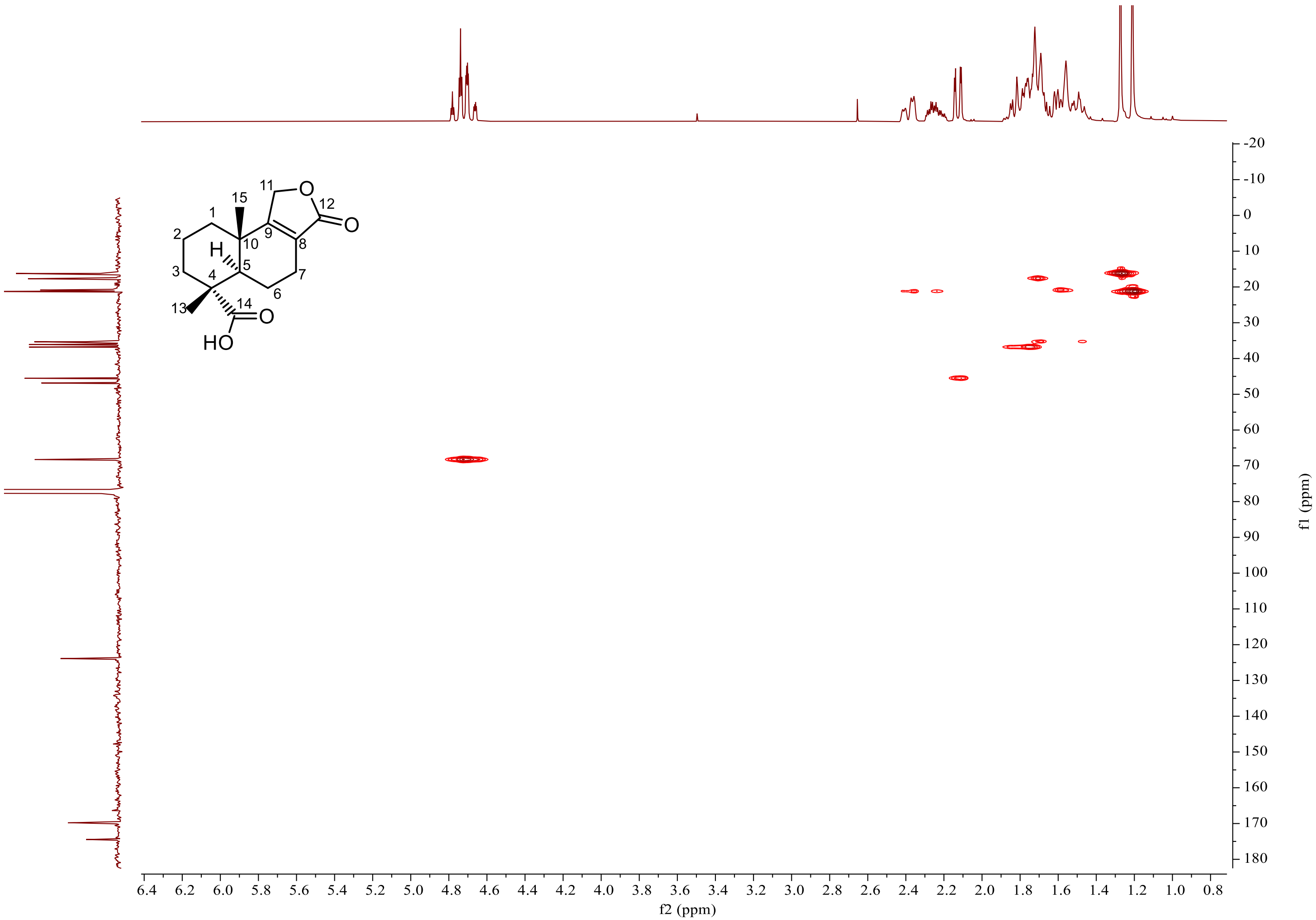

Figure S50. The HSQC spectrum of ustidrimane D (**10**) in $CDCl_3$.

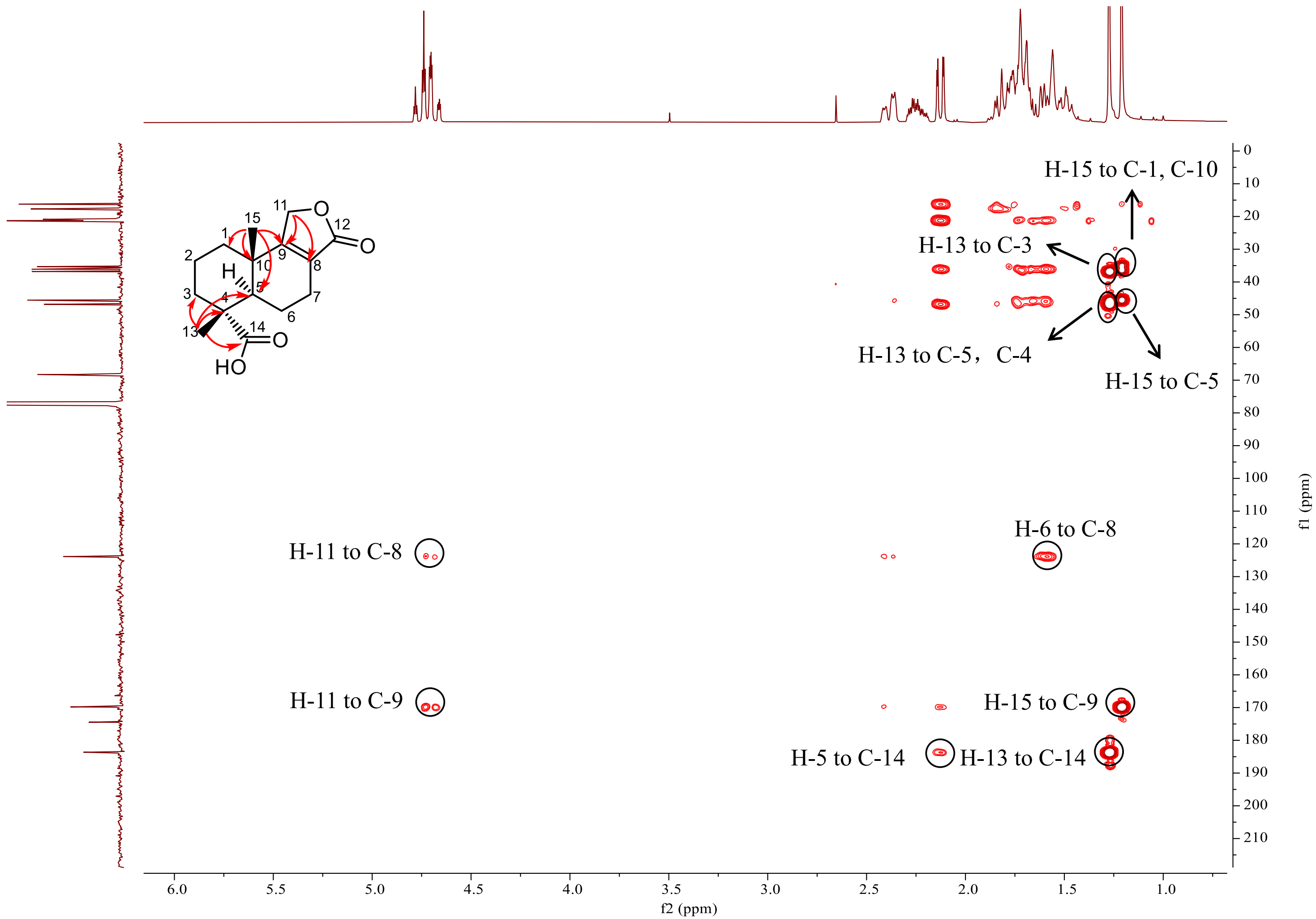

Figure S51. The HMBC spectrum of ustidrimane D (**10**) in $CDCl_3$.

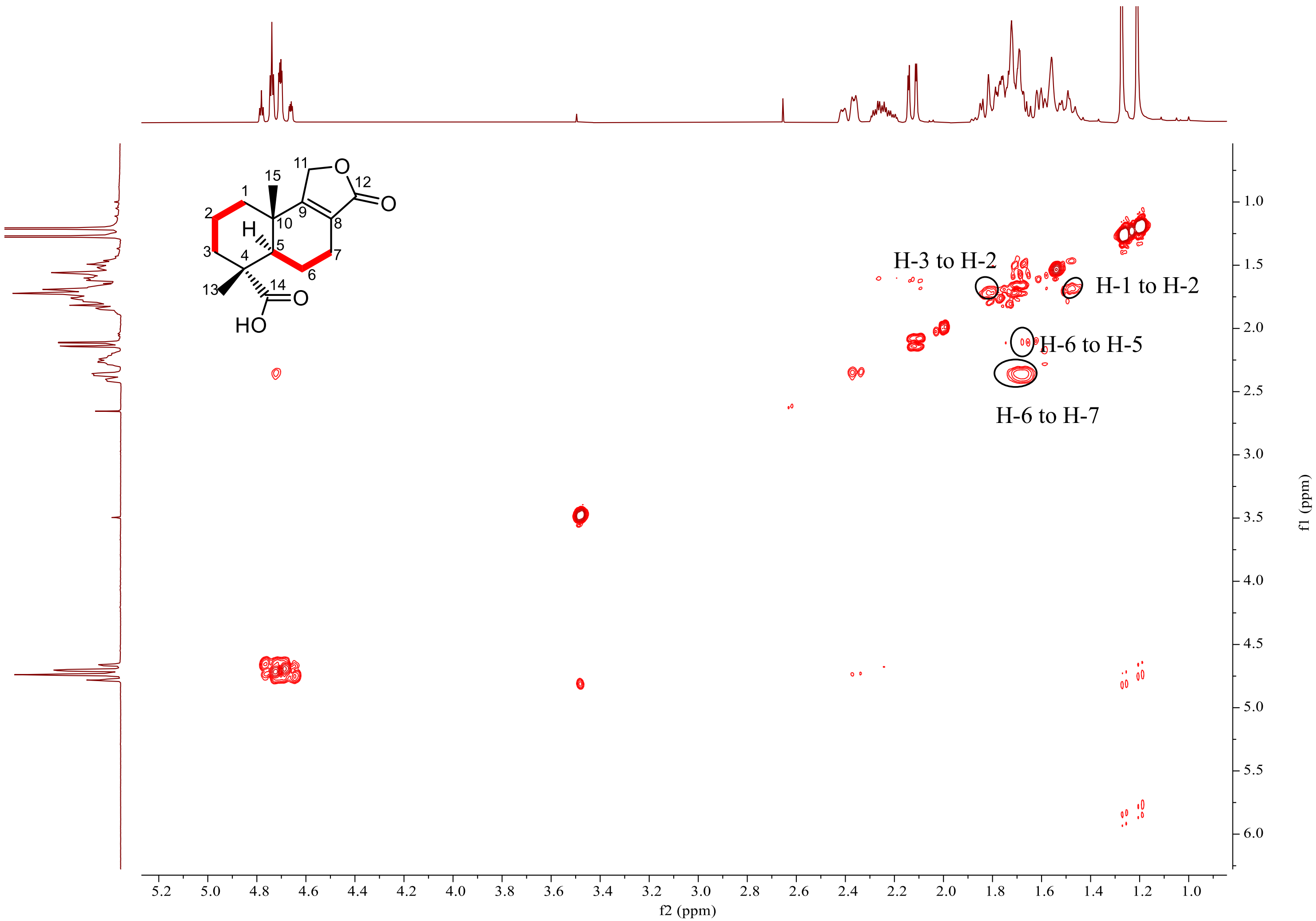

Figure S52. The ^{1}H-^{1}H COSY spectrum of ustidrimane D (**10**) in $CDCl_3$.

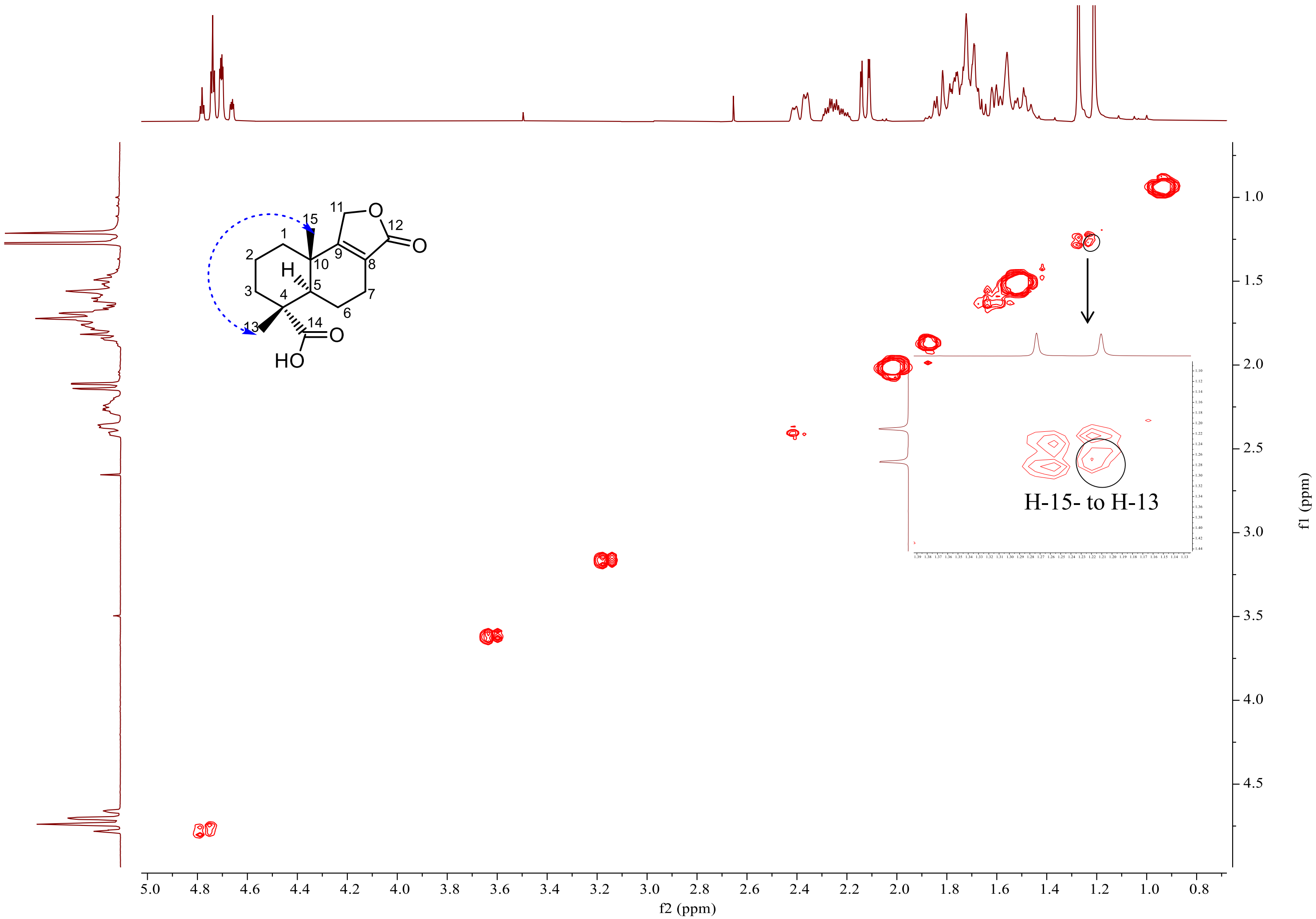

Figure S53. The NOESY spectrum of ustidrimane D (**10**) in $CDCl_3$.

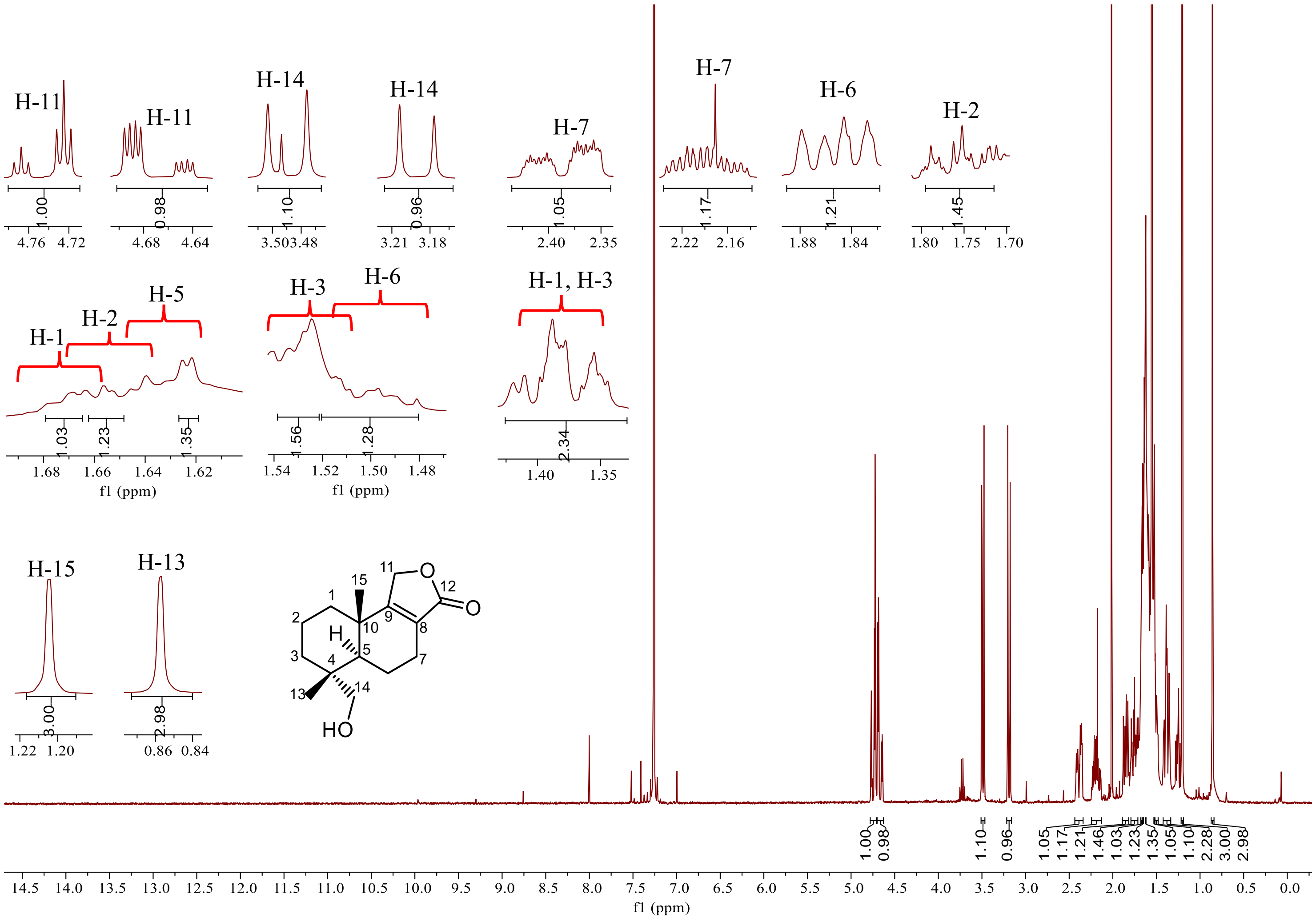

Figure S54. The ^{1}H NMR spectrum of ustidrimane E (**11**) in $CDCl_3$ (400 MHz).

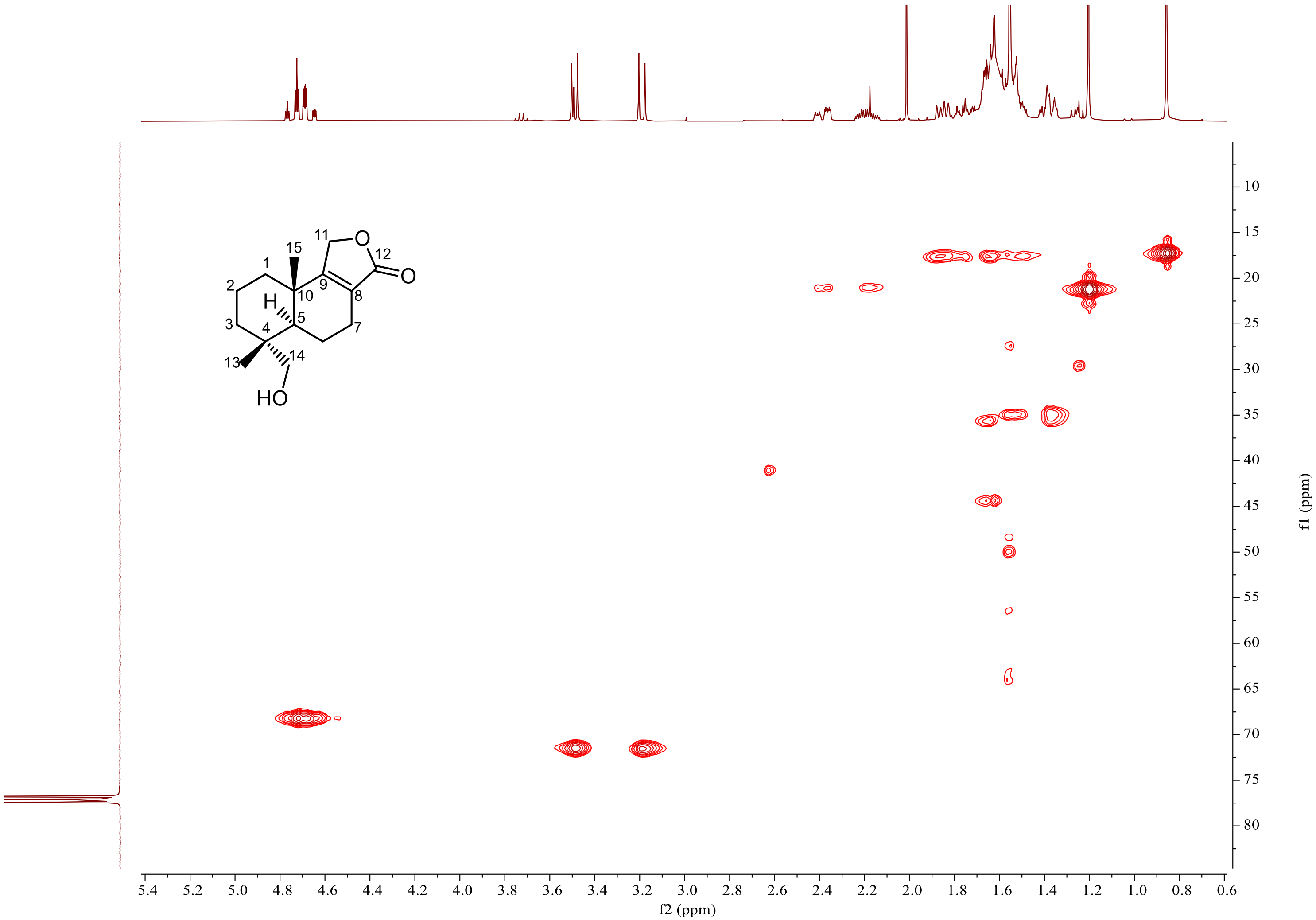

Figure S55. The HSQC spectrum of ustidrimane E (**11**) in $CDCl_3$.

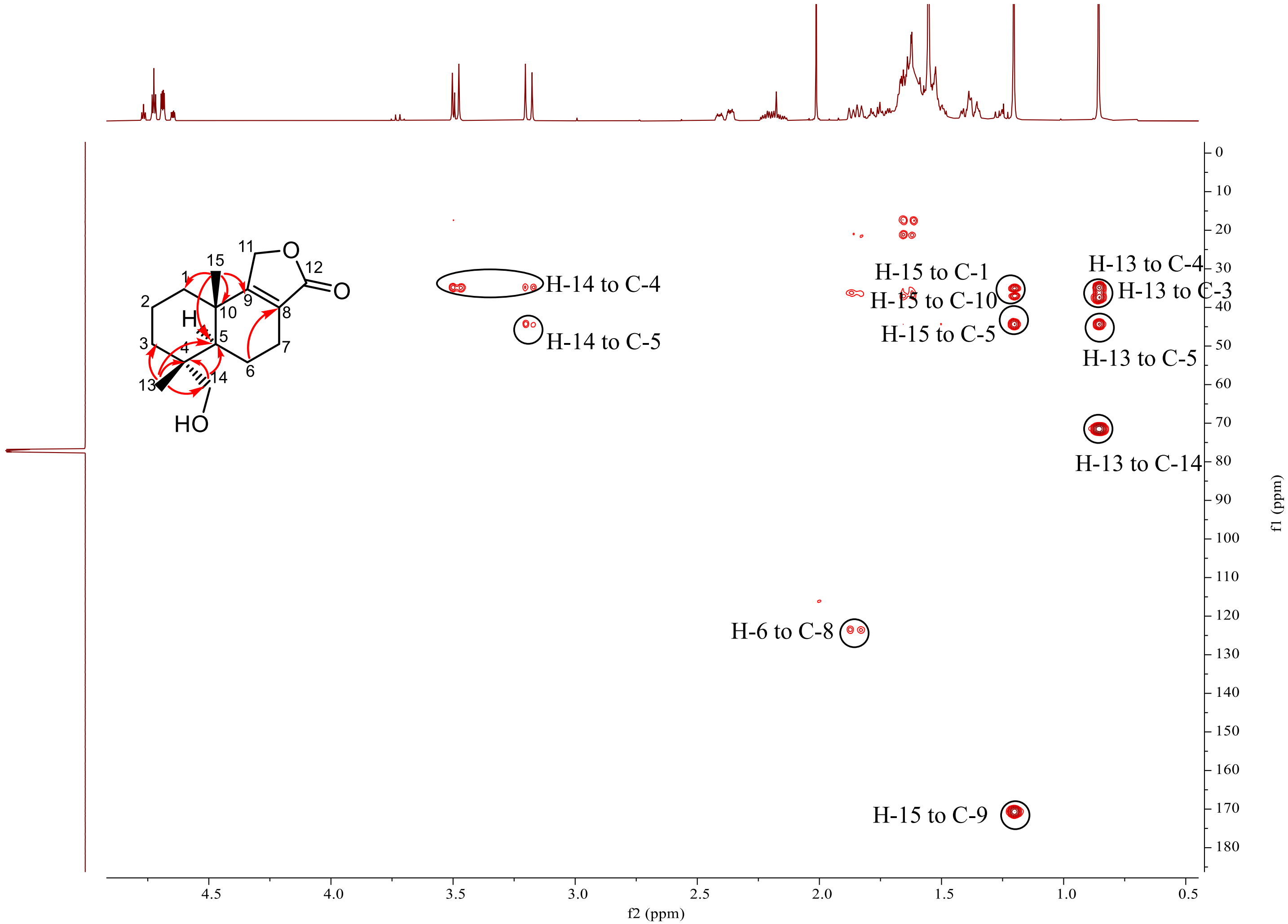

Figure S56. The HMBC spectrum of ustidrimane E (**11**) in $CDCl_3$.

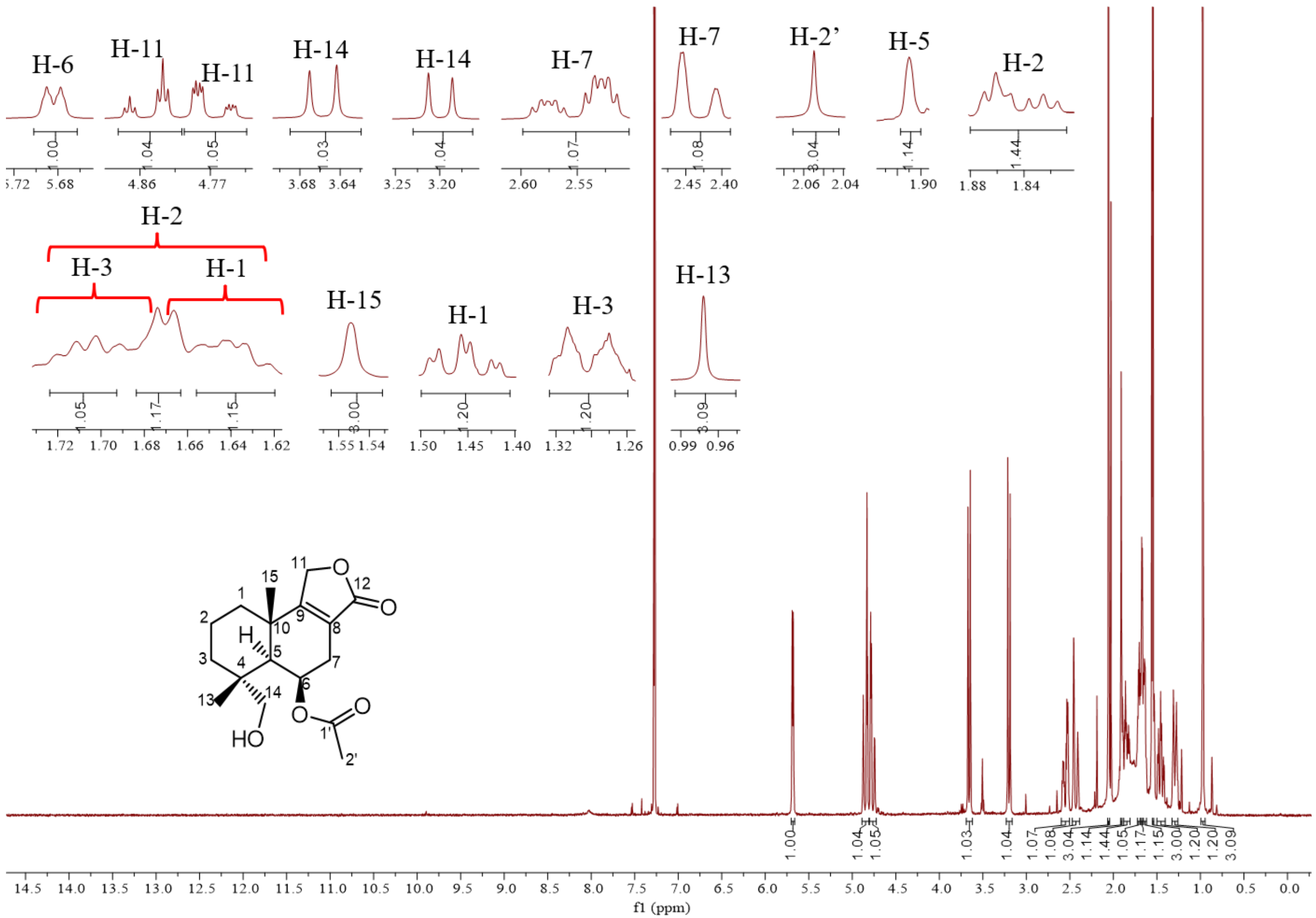

Figure S57. The ^{1}H NMR spectrum of ustidrimane F (**12**) in $CDCl_3$ (400 MHz).

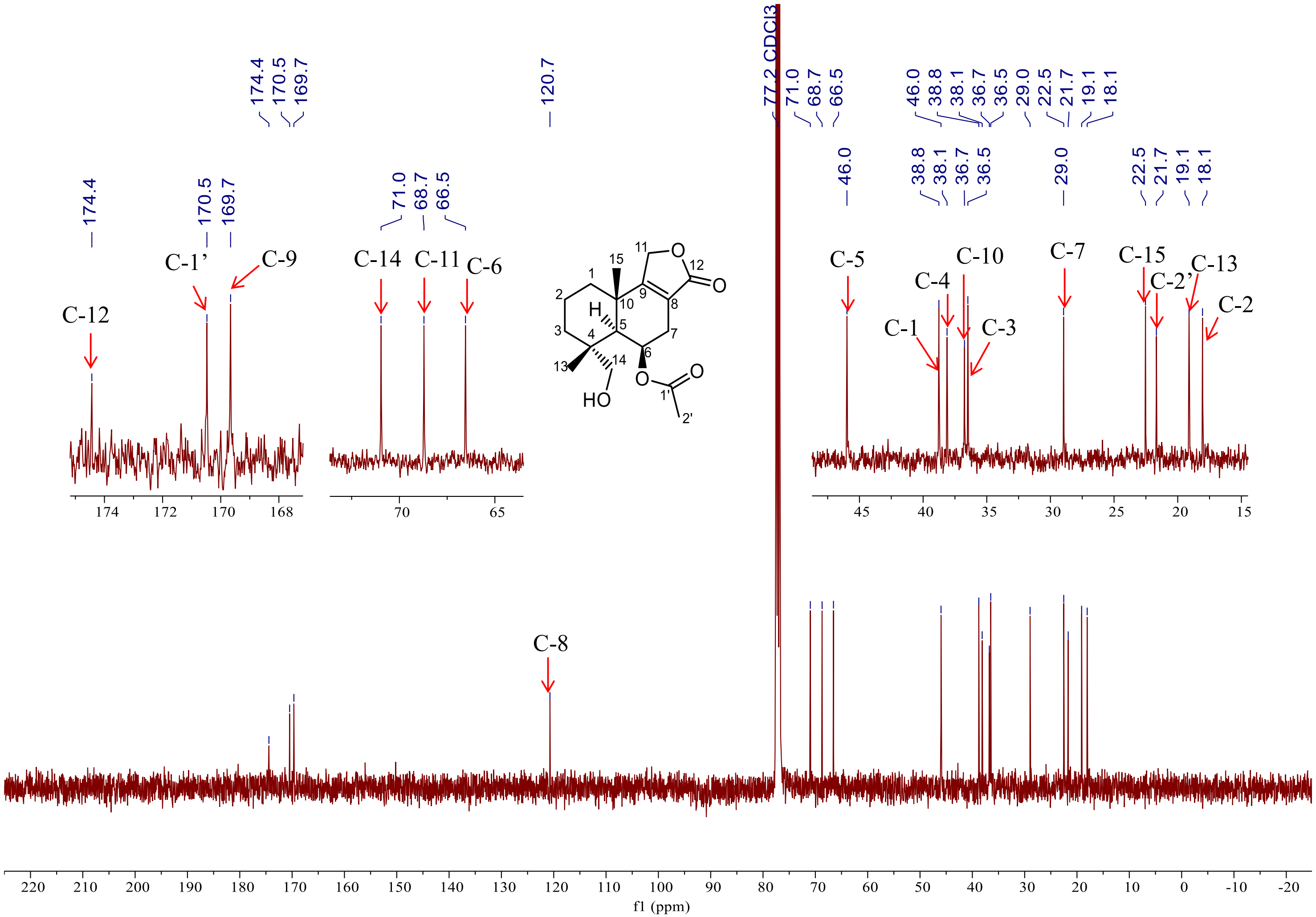

Figure S58. The ^{13}C NMR spectrum of ustidrimane F (**12**) in $CDCl_3$ (100 MHz).

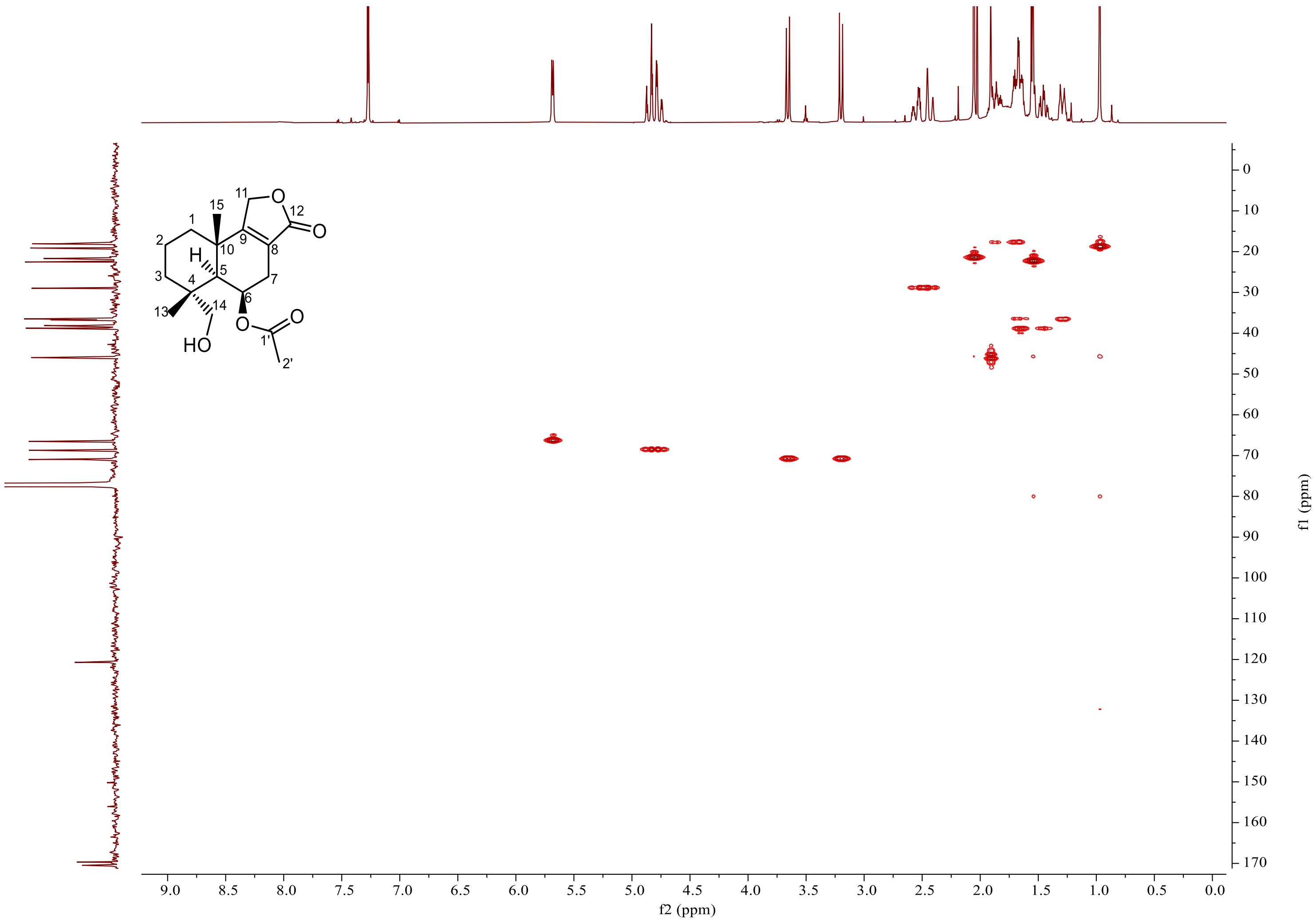

Figure S59. The HSQC spectrum of ustidrimane F (**12**) in $CDCl_3$.

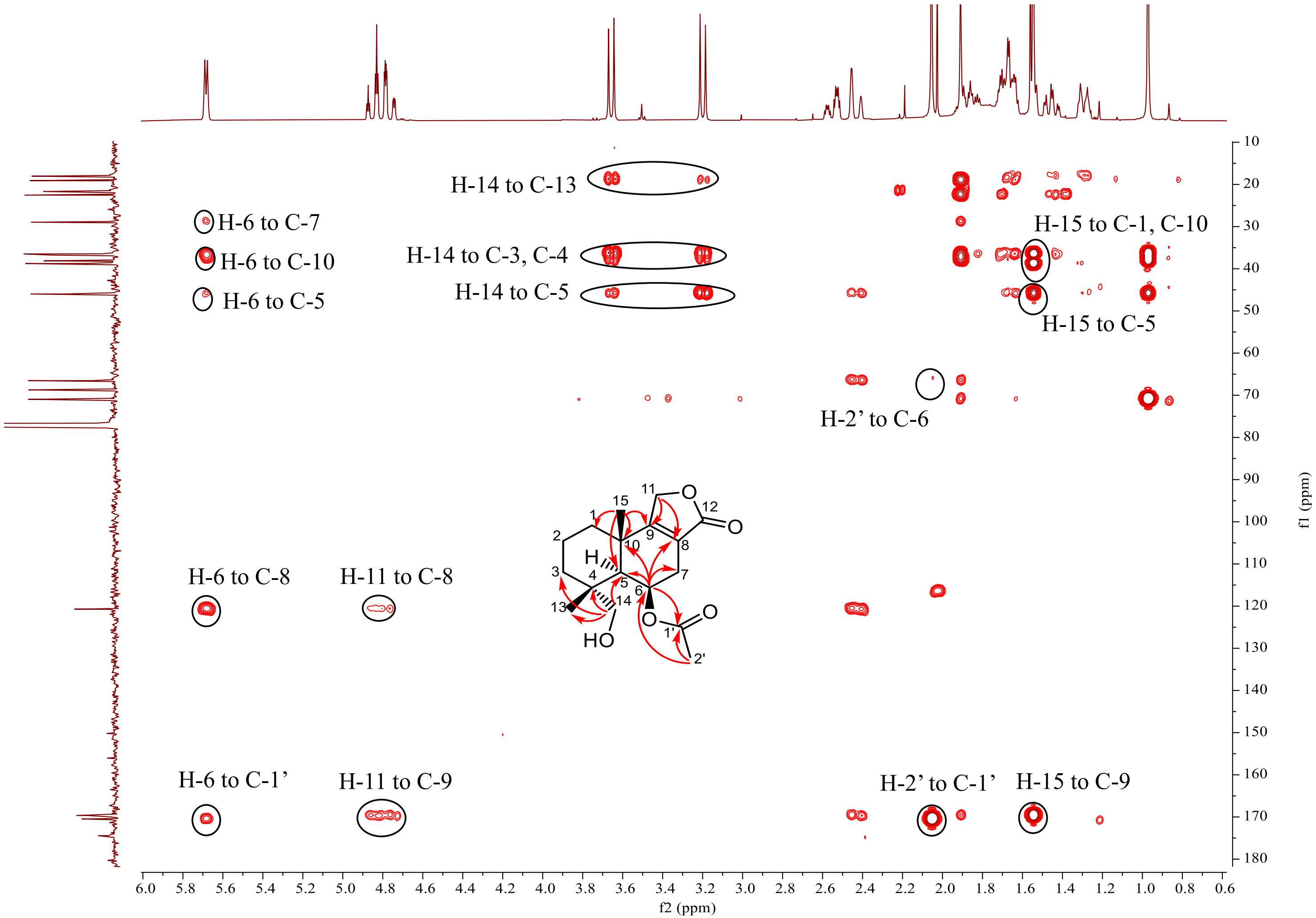

Figure S60. The HMBC spectrum of ustidrimane F (**12**) in $CDCl_3$.

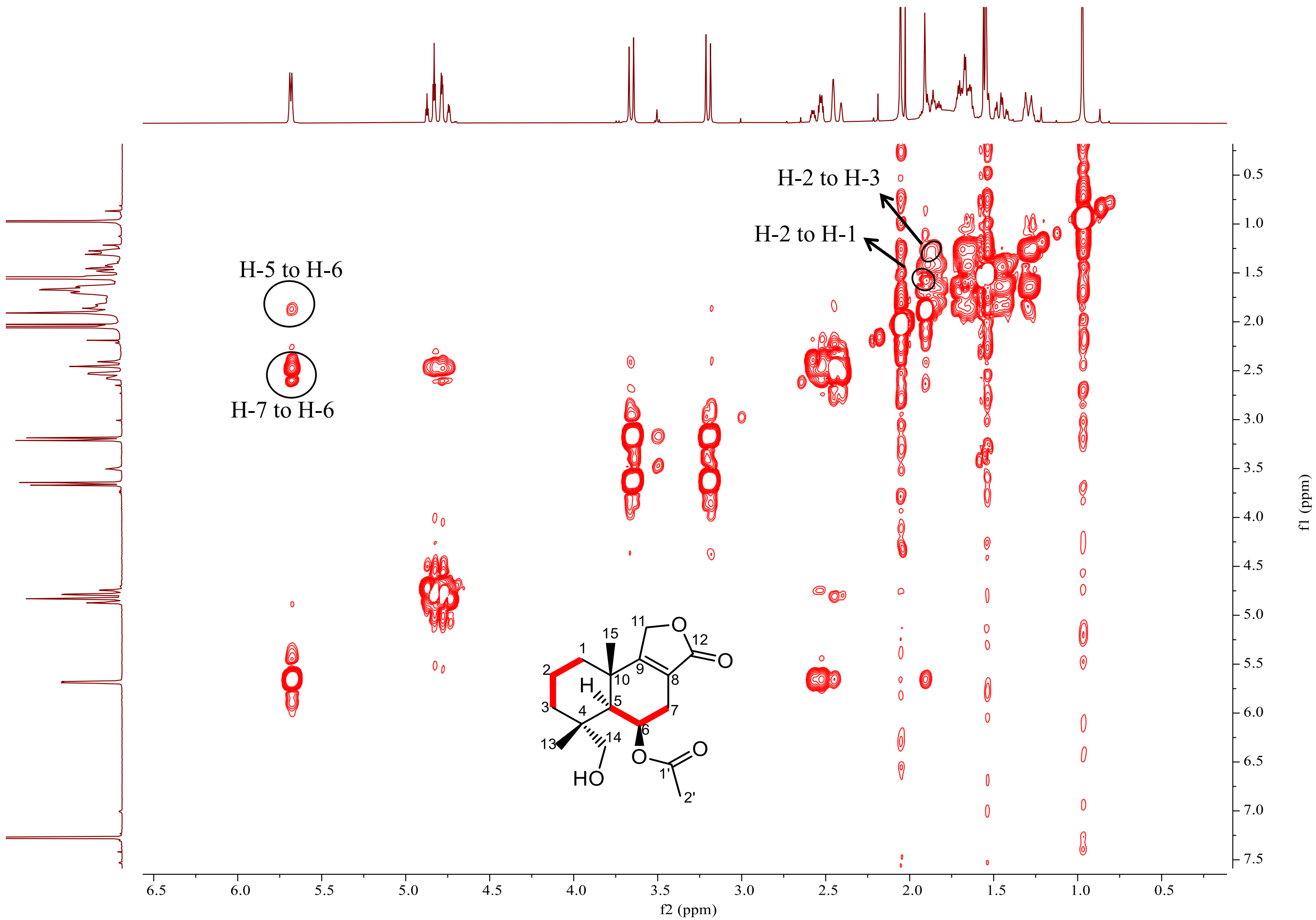

Figure S61. The ^{1}H-^{1}H COSY spectrum of ustidrimane F (**12**) in $CDCl_3$.

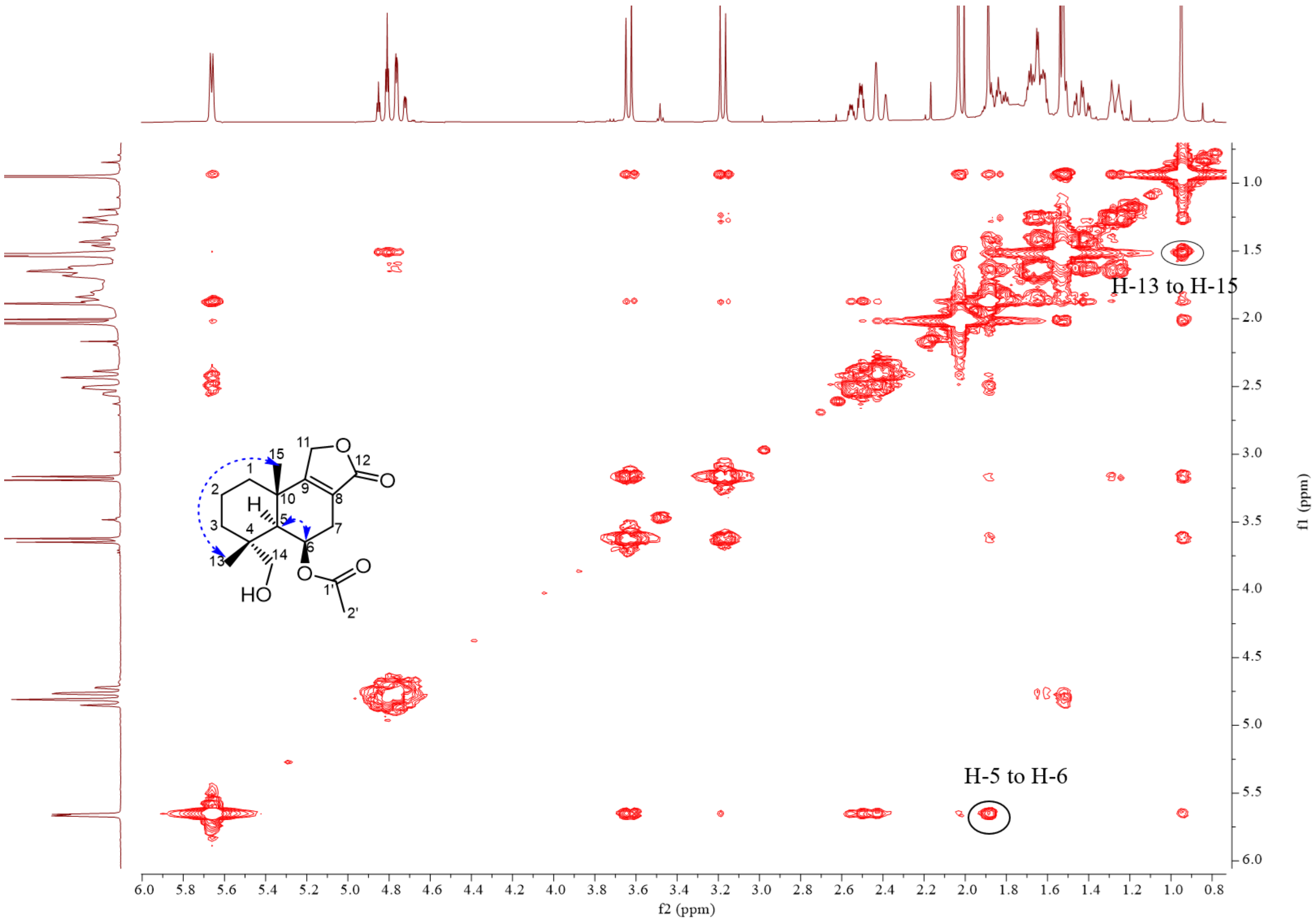

Figure S62. The NOESY spectrum of ustidrimane F (**12**) in $CDCl_3$.

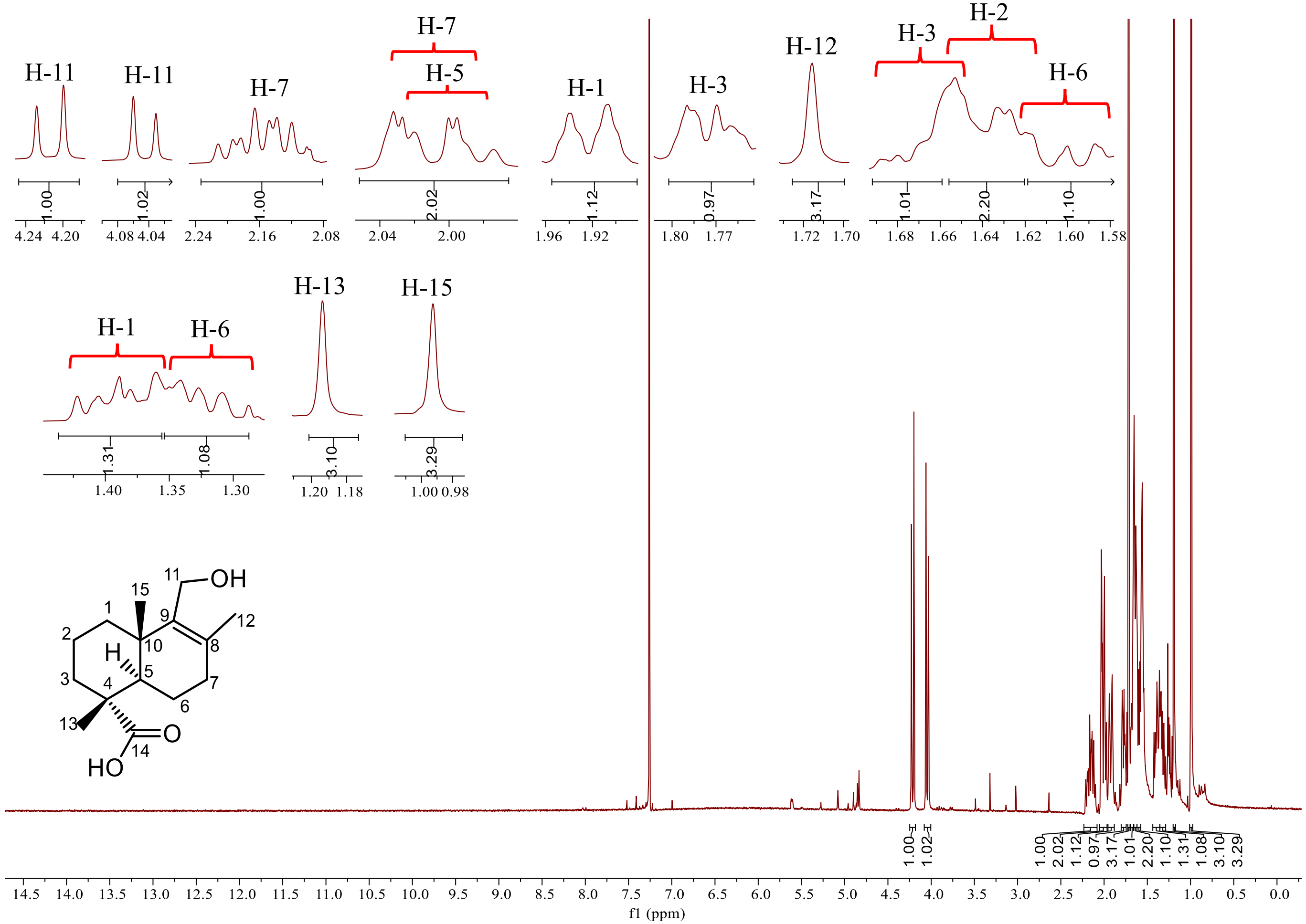

Figure S63. The ^{1}H NMR spectrum of ustidrimane G (**13**) in $CDCl_3$ (400 MHz).

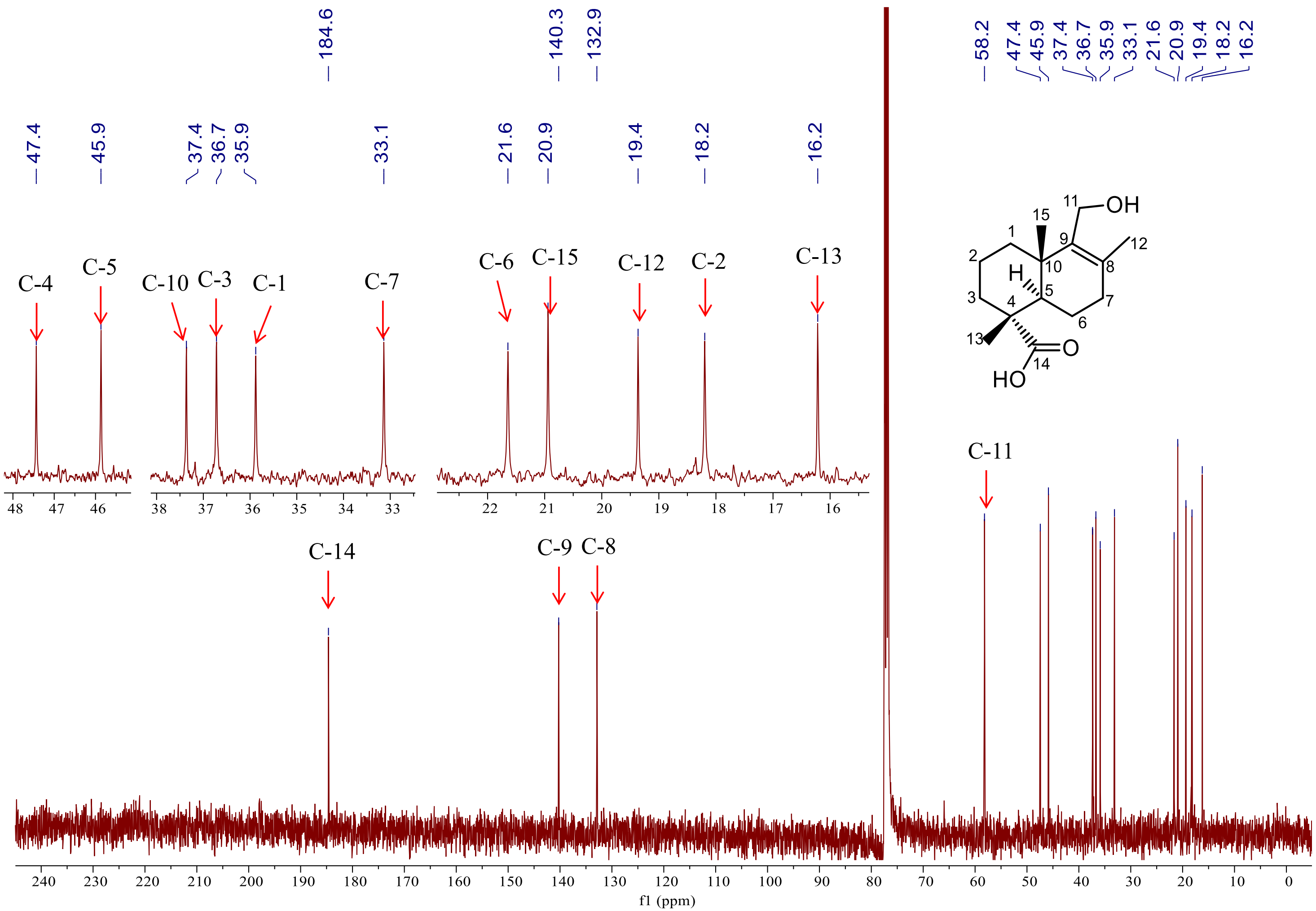

Figure S64. The ^{13}C NMR spectrum of ustidrimane G (**13**) in $CDCl_3$ (100 MHz).

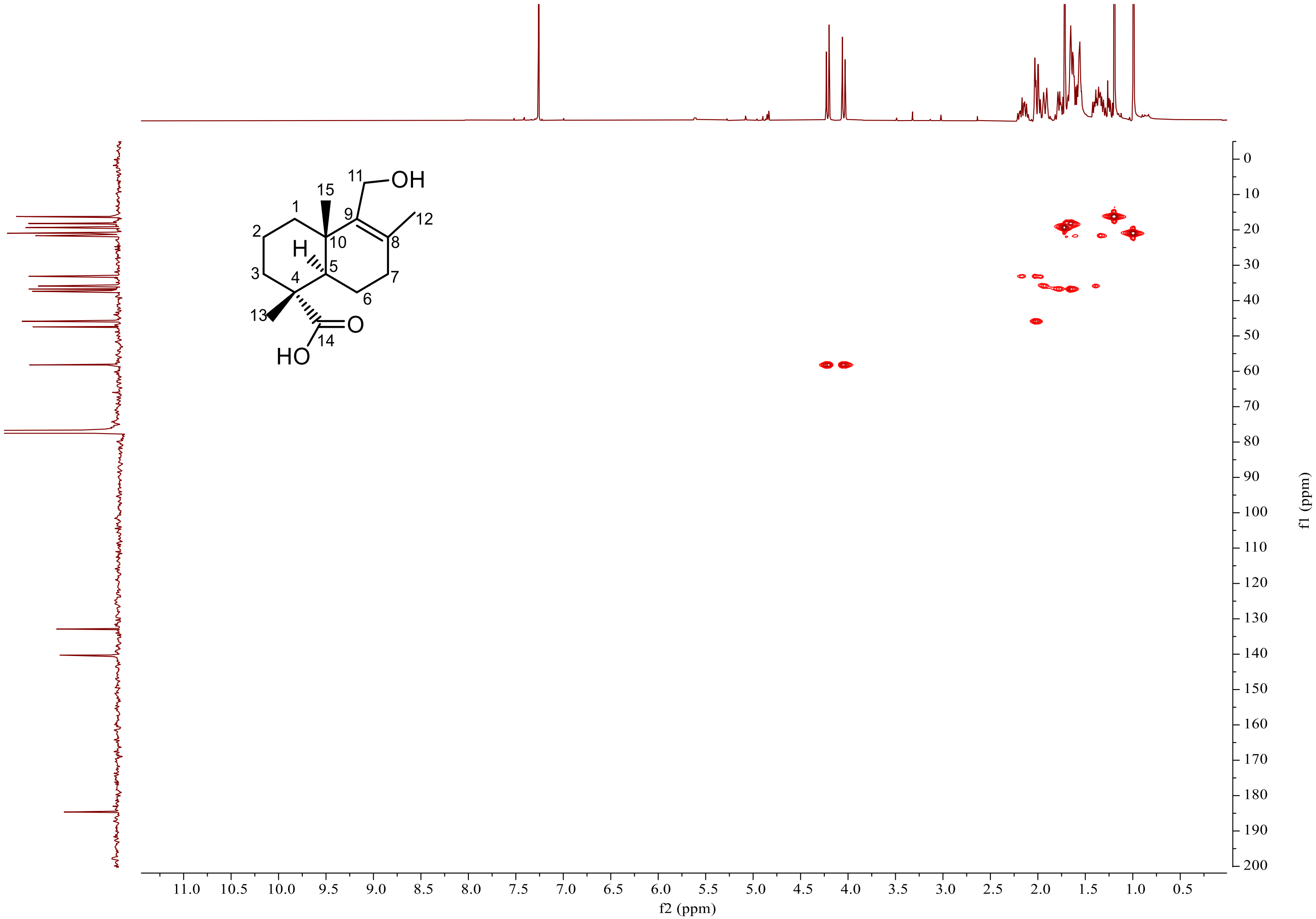

Figure S65. The HSQC spectrum of ustidrimane G (**13**) in $CDCl_3$.

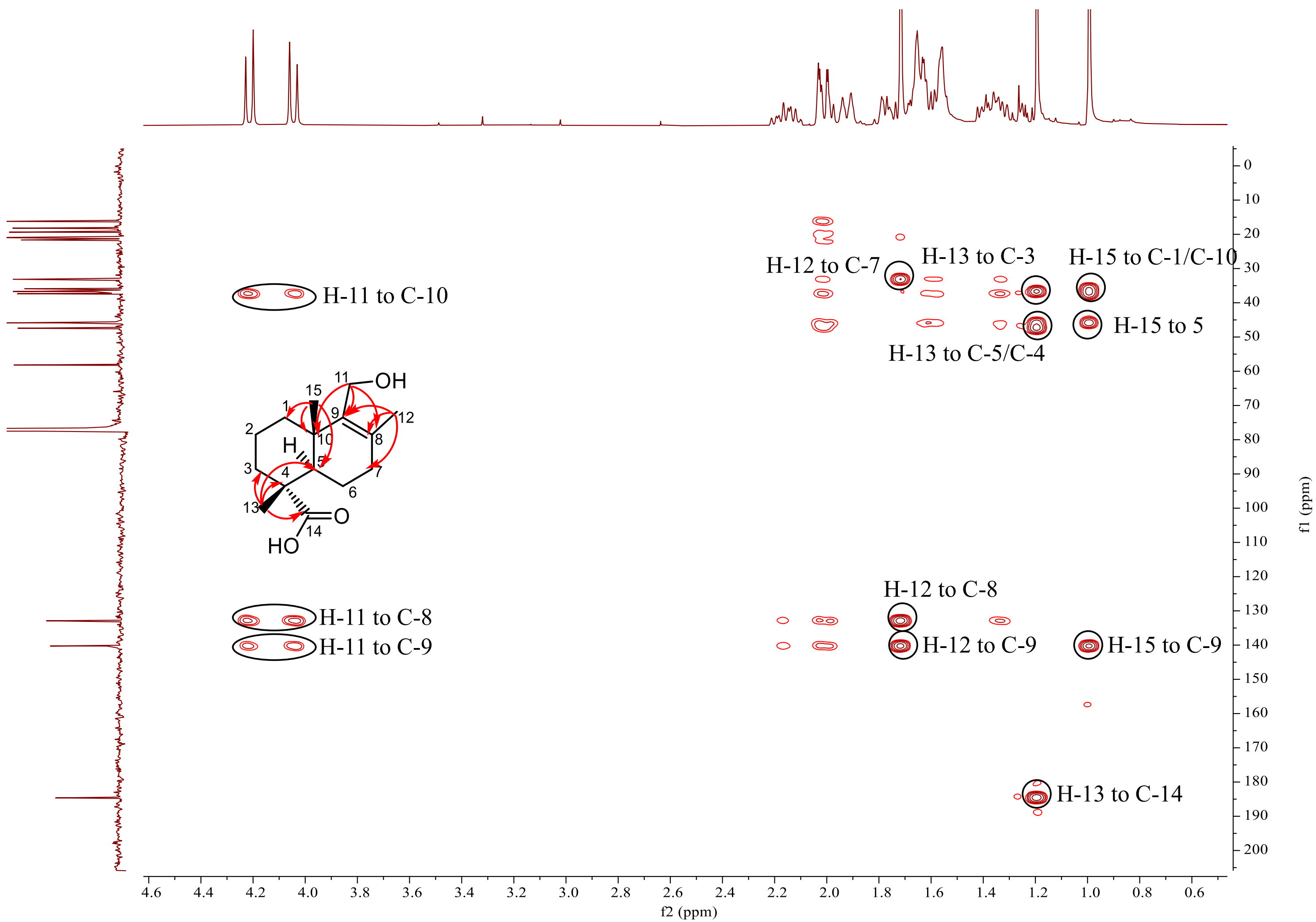

Figure S66. The HMBC spectrum of ustidrimane G (**13**) in $CDCl_3$.

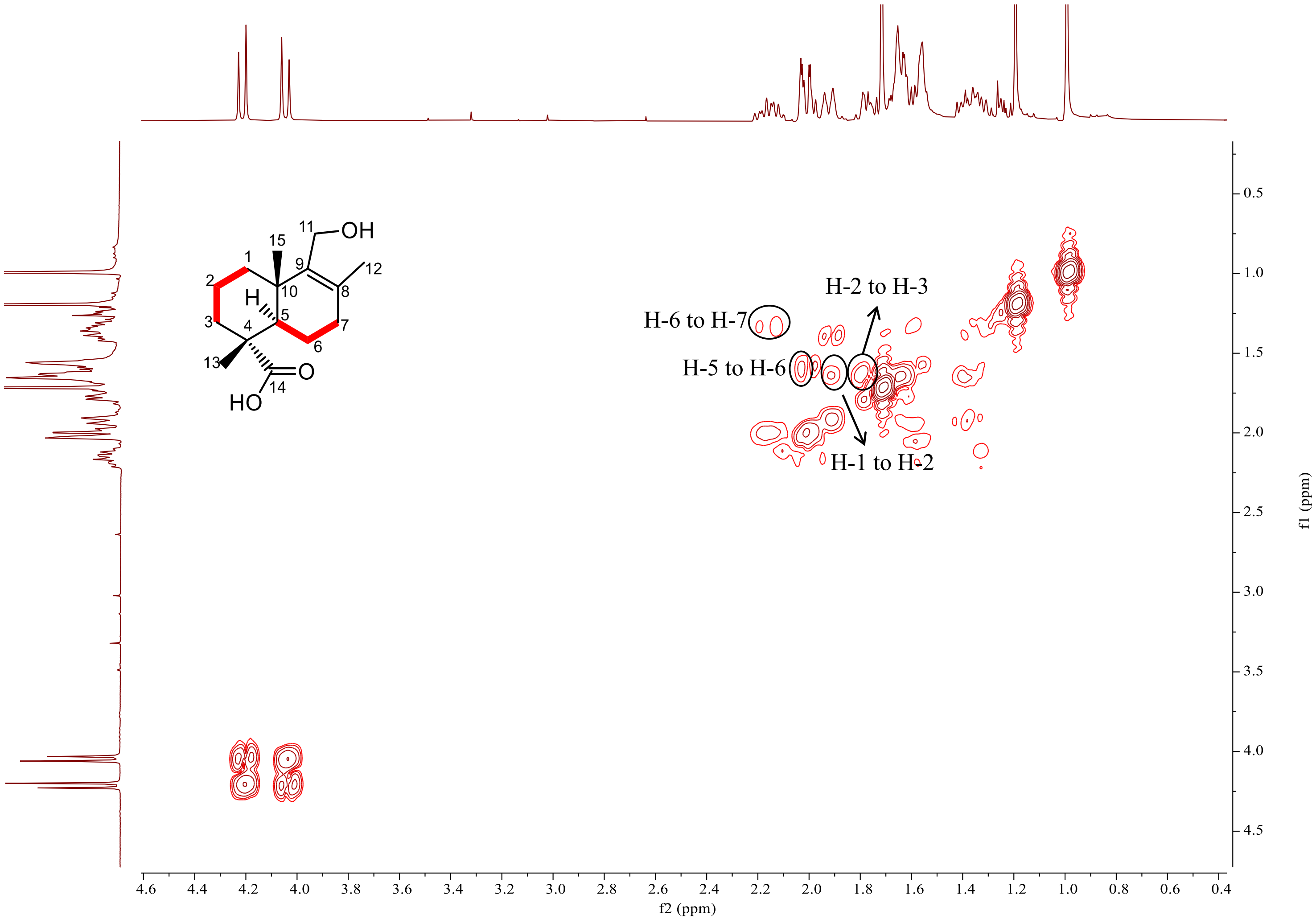

Figure S67. The ^{1}H-^{1}H COSY spectrum of ustidrimane G (**13**) in $CDCl_3$.

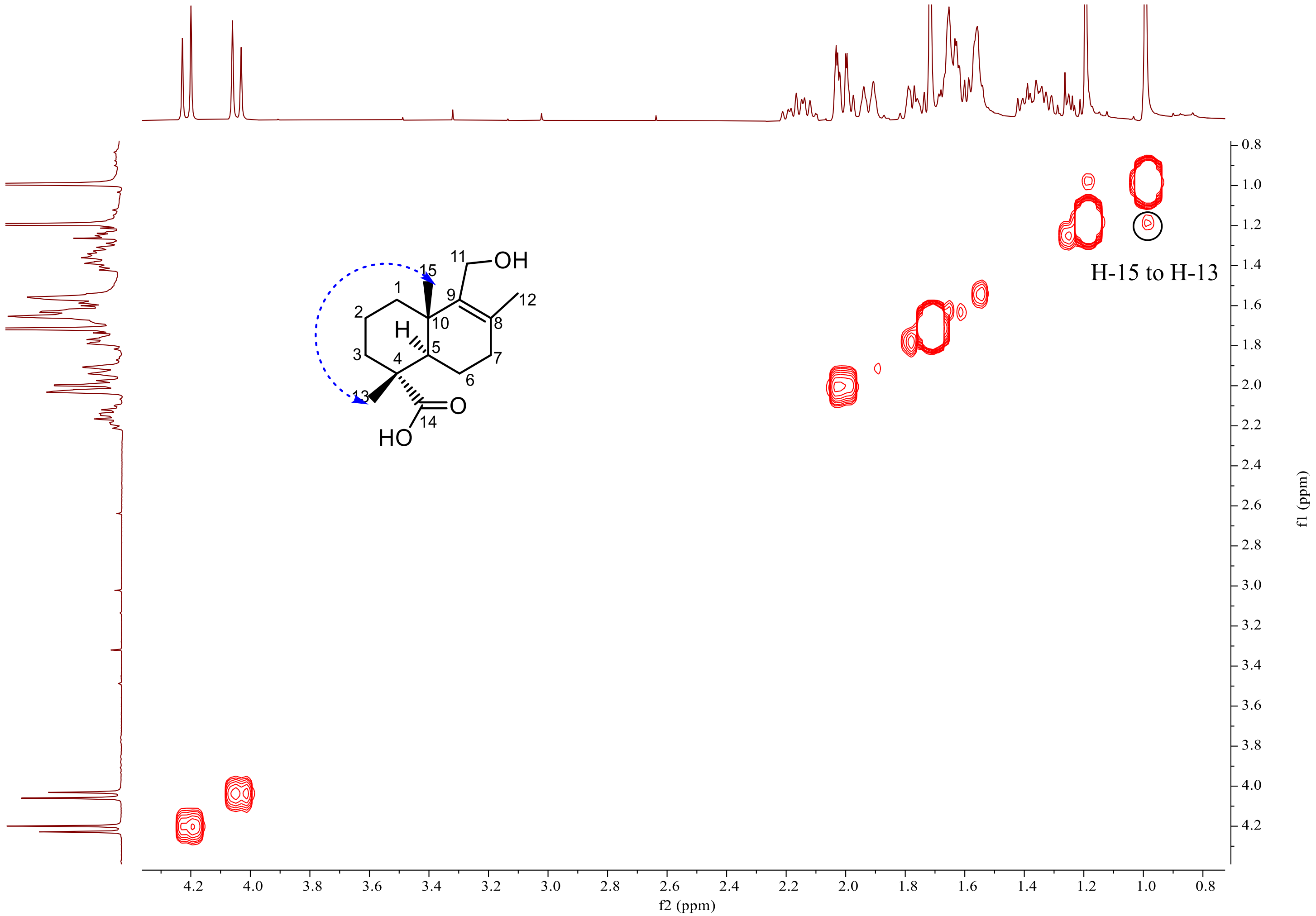

Figure S68. The NOESY spectrum of ustidrimane G (**13**) in $CDCl_3$.

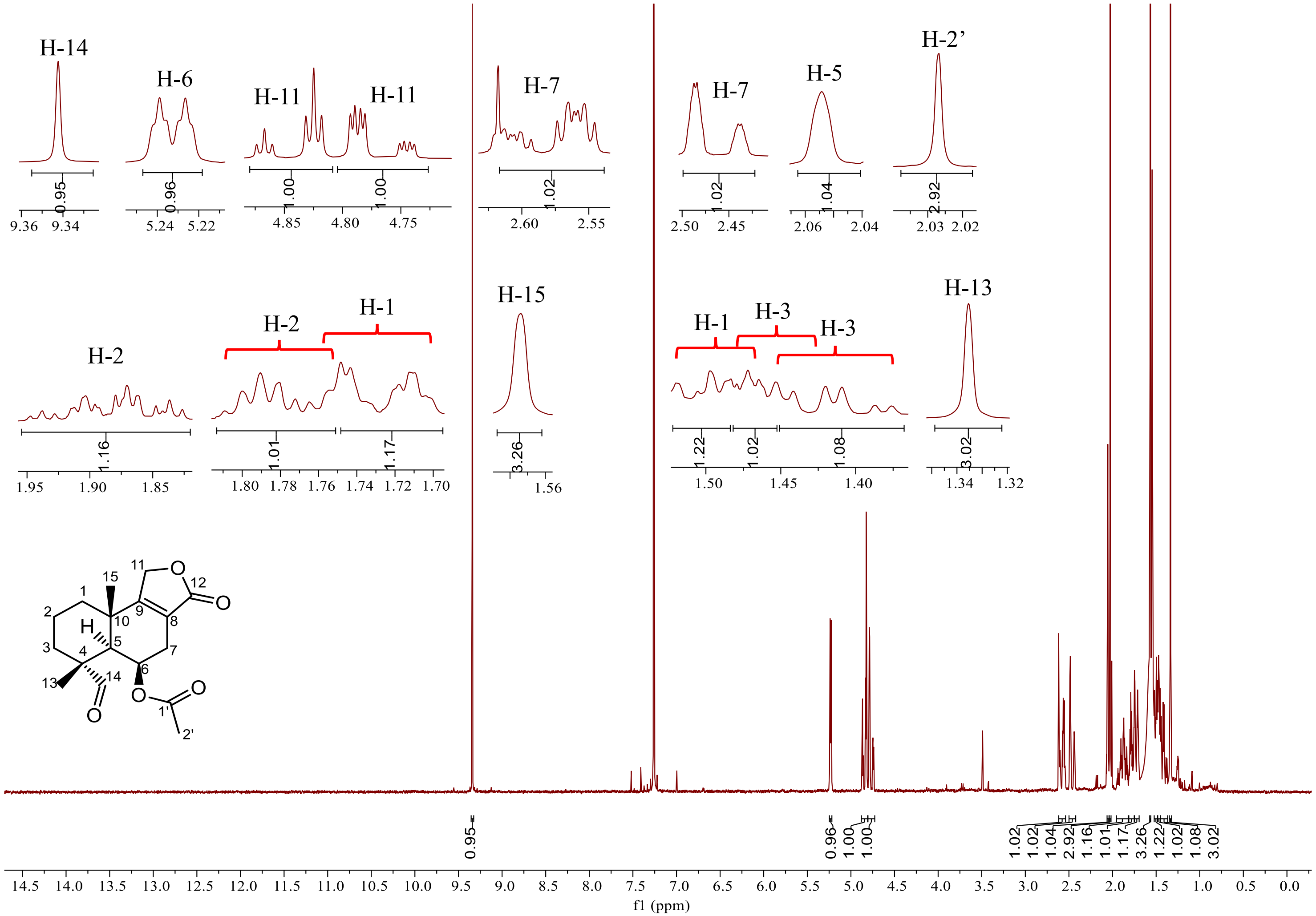

Figure S69. The ^{1}H NMR spectrum of ustidrimane H (**14**) in $CDCl_3$ (400 MHz).

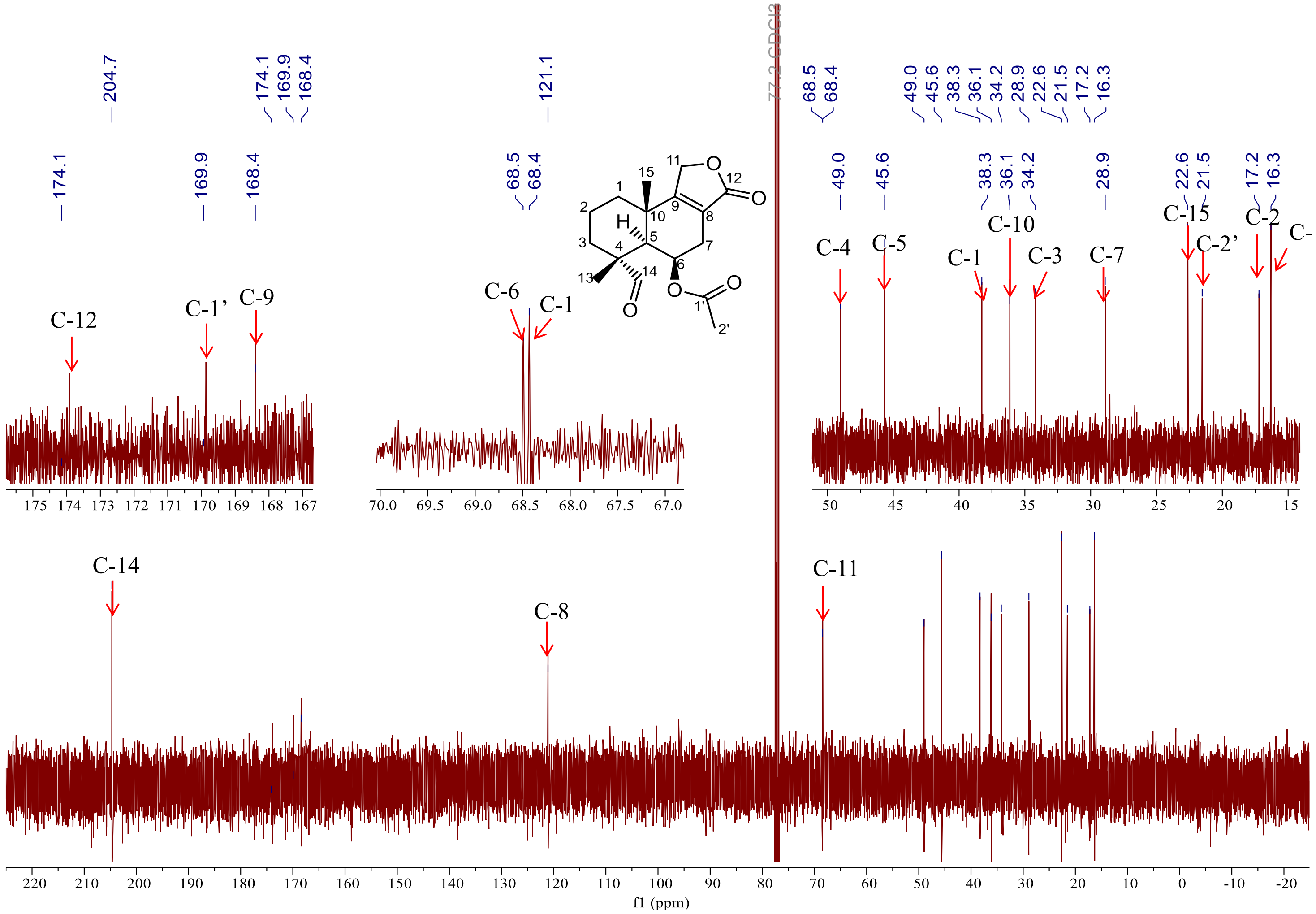

Figure S70. The ^{13}C NMR spectrum of ustidrimane H (**14**) in $CDCl_3$ (100 MHz).

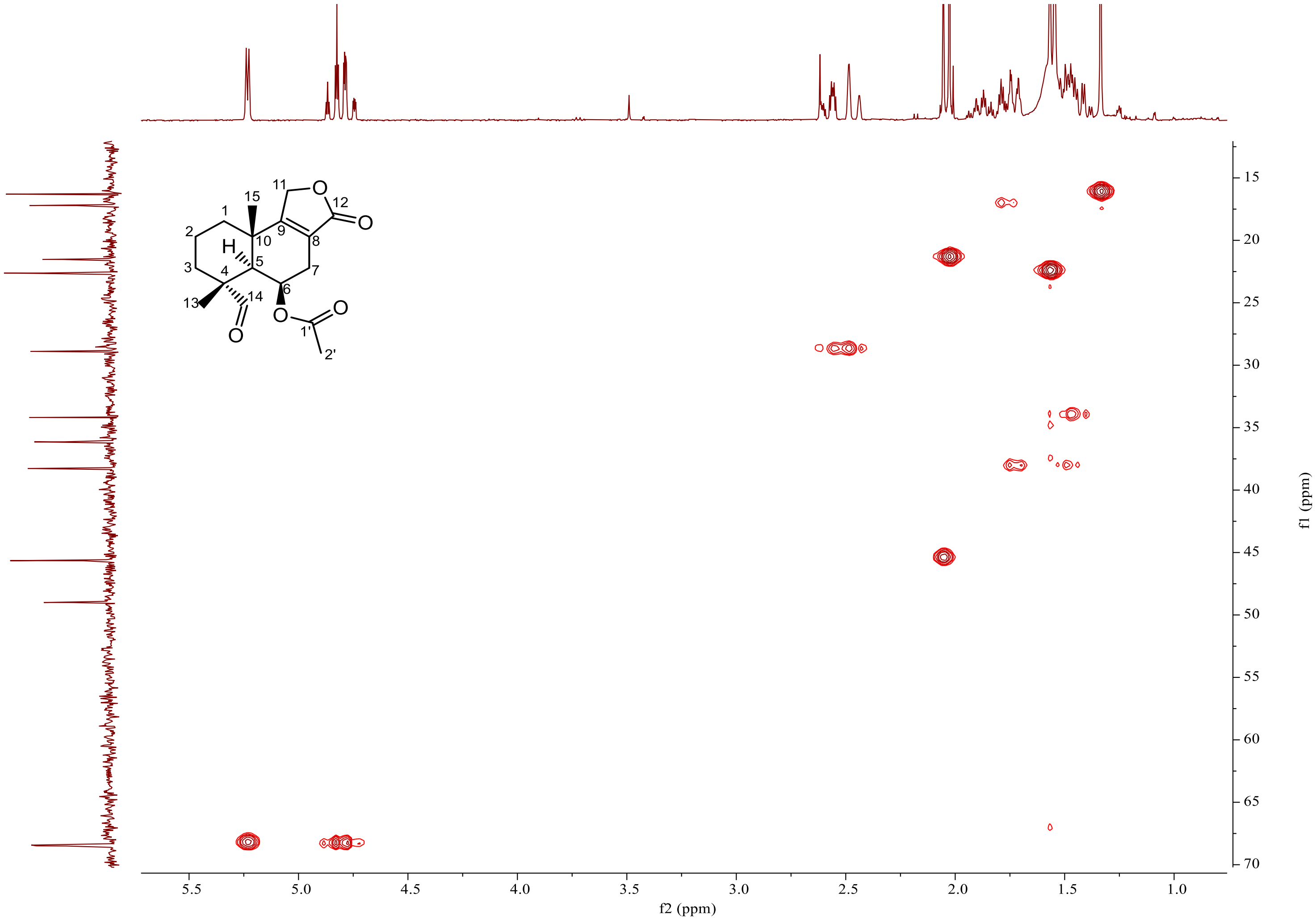

Figure S71. The HSQC spectrum of ustidrimane H (**14**) in $CDCl_3$.

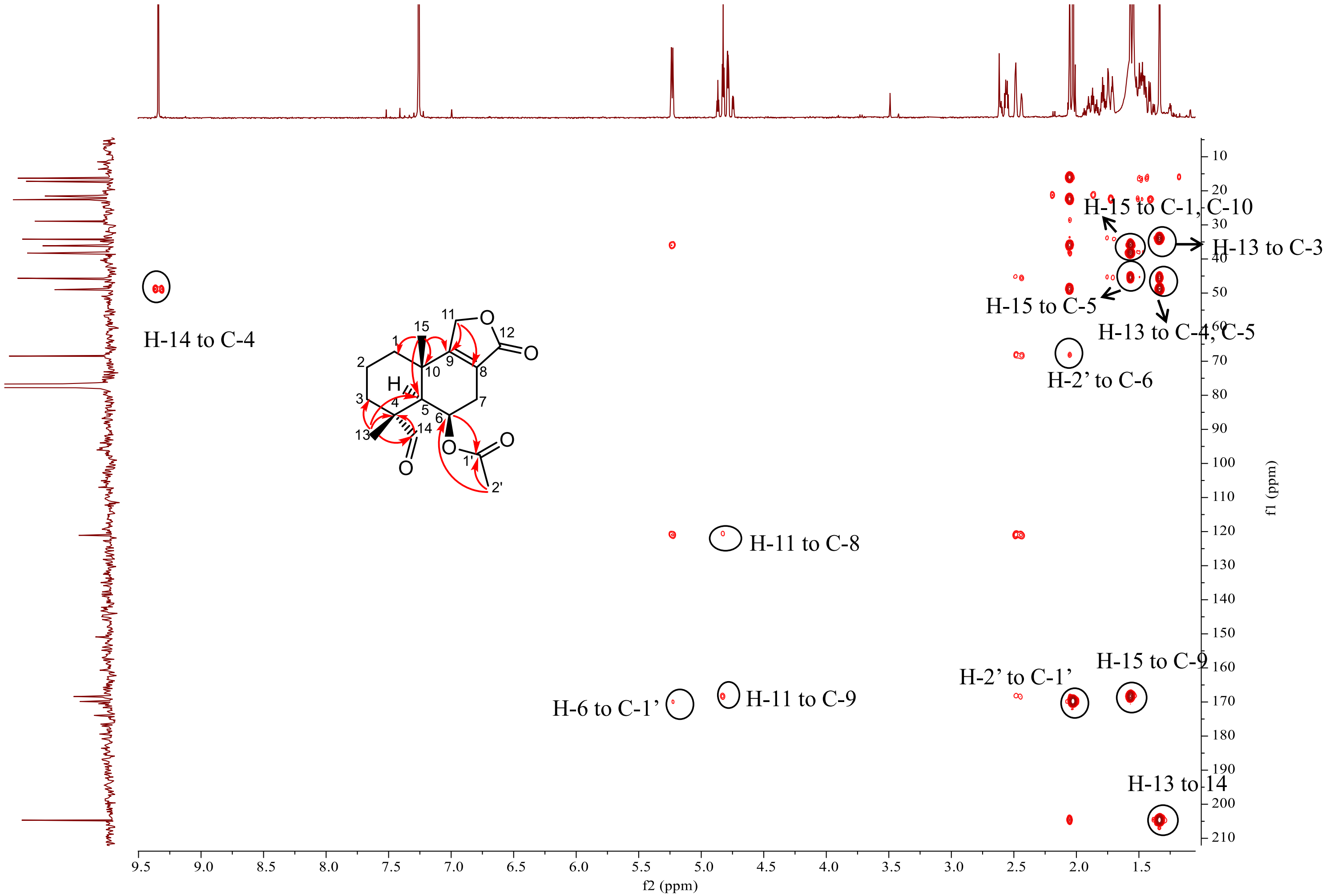

Figure S72. The HMBC spectrum of ustidrimane H (**14**) in $CDCl_3$.

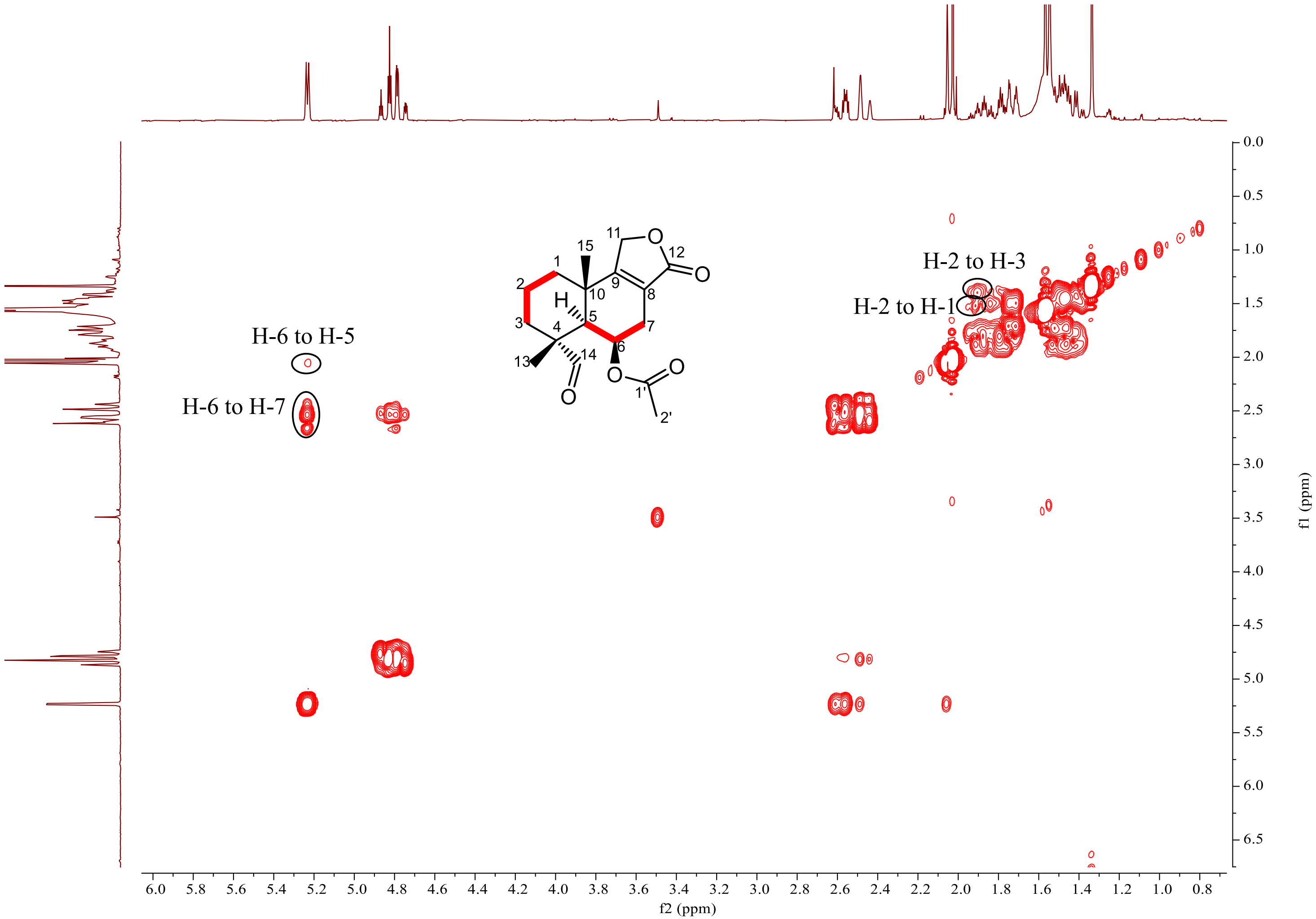

Figure S73. The ^{1}H-^{1}H COSY spectrum of ustidrimane H (**14**) in $CDCl_3$.

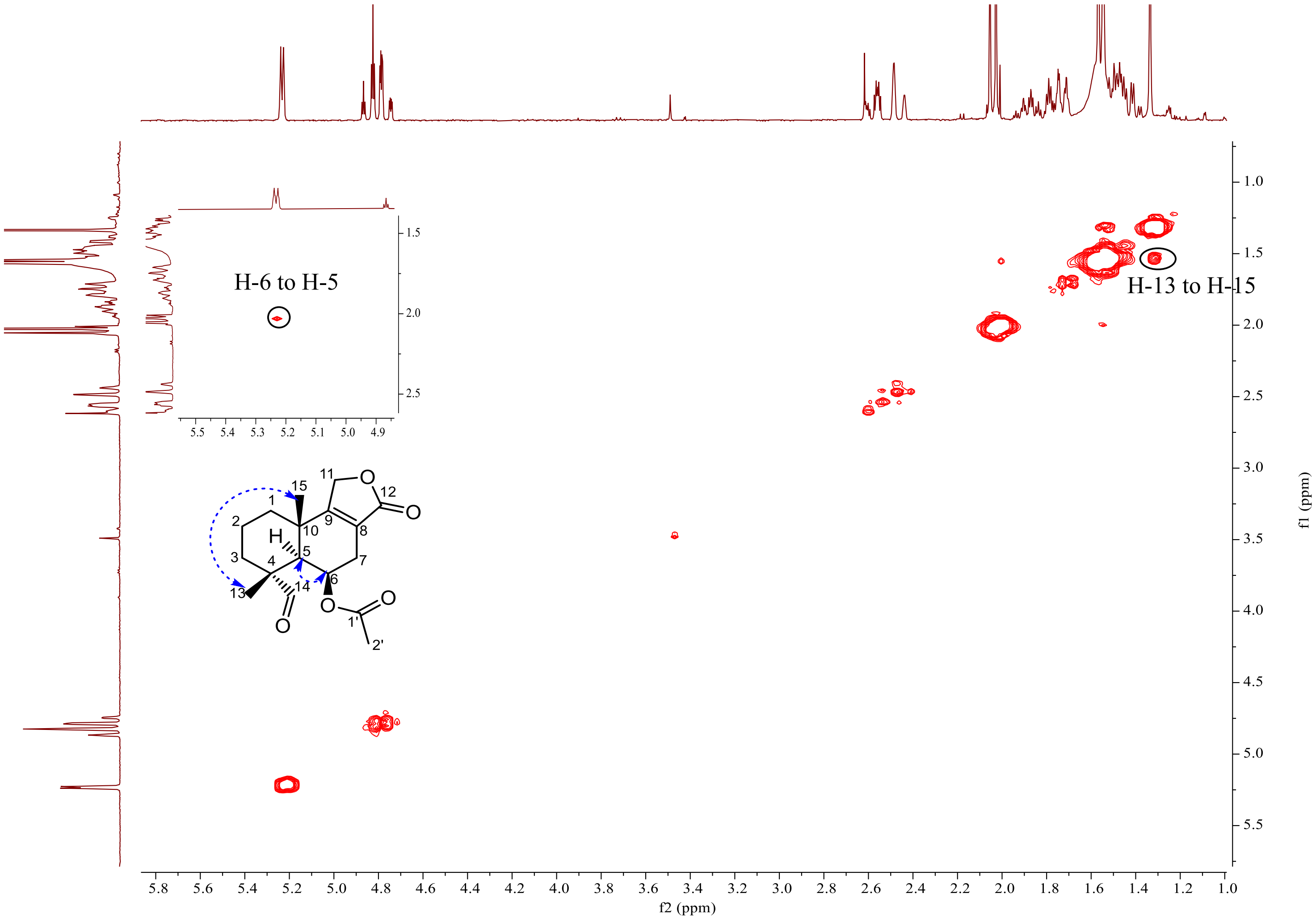

Figure S74. The NOESY spectrum of ustidrimane H (**14**) in $CDCl_3$.

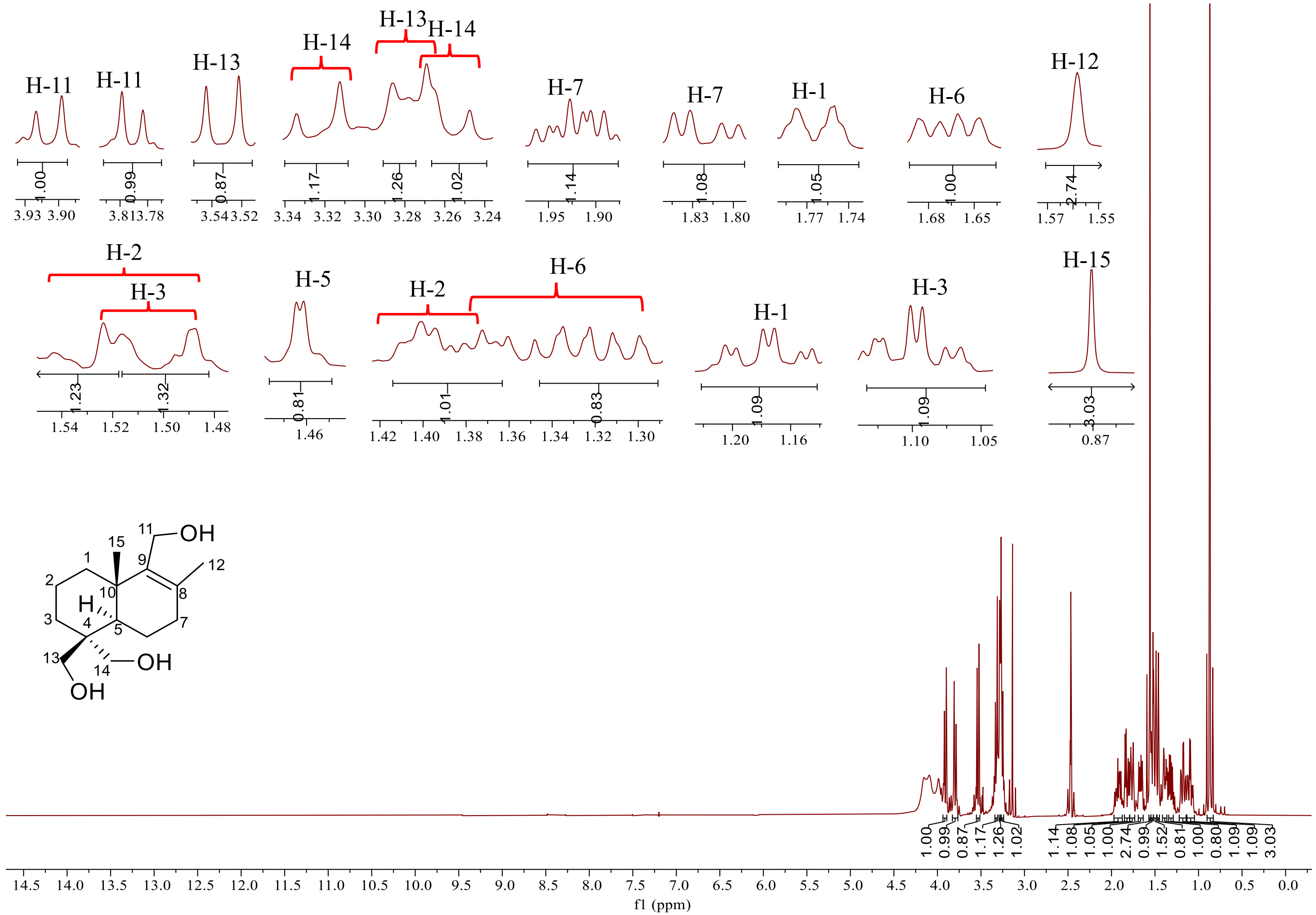

Figure S75. The ^{1}H NMR spectrum of ustidrimane I (**15**) in DMSO-d_6 (400 MHz).

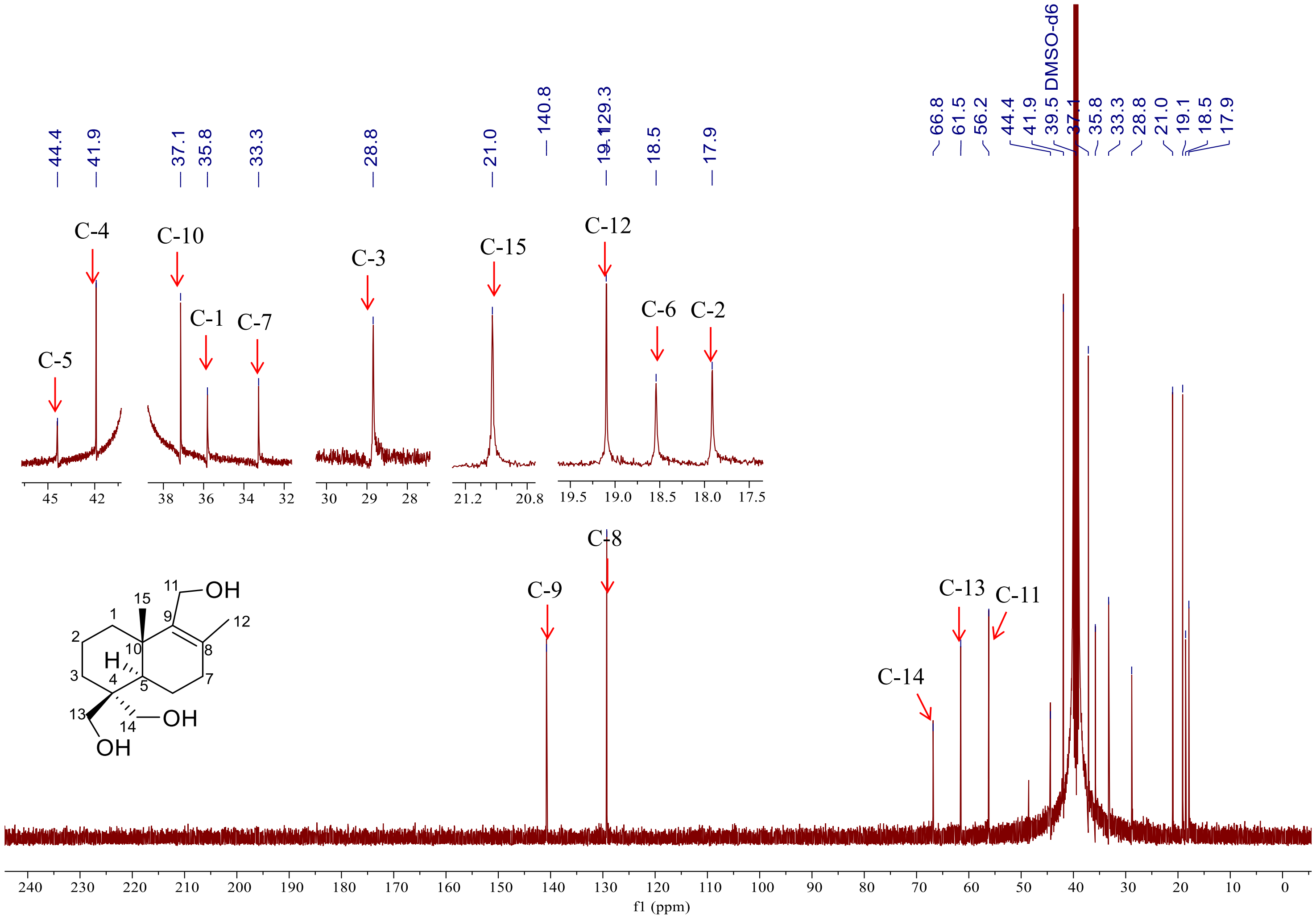

Figure S76. The ^{13}C NMR spectrum of ustidrimane I (**15**) in DMSO-d_6 (100 MHz).

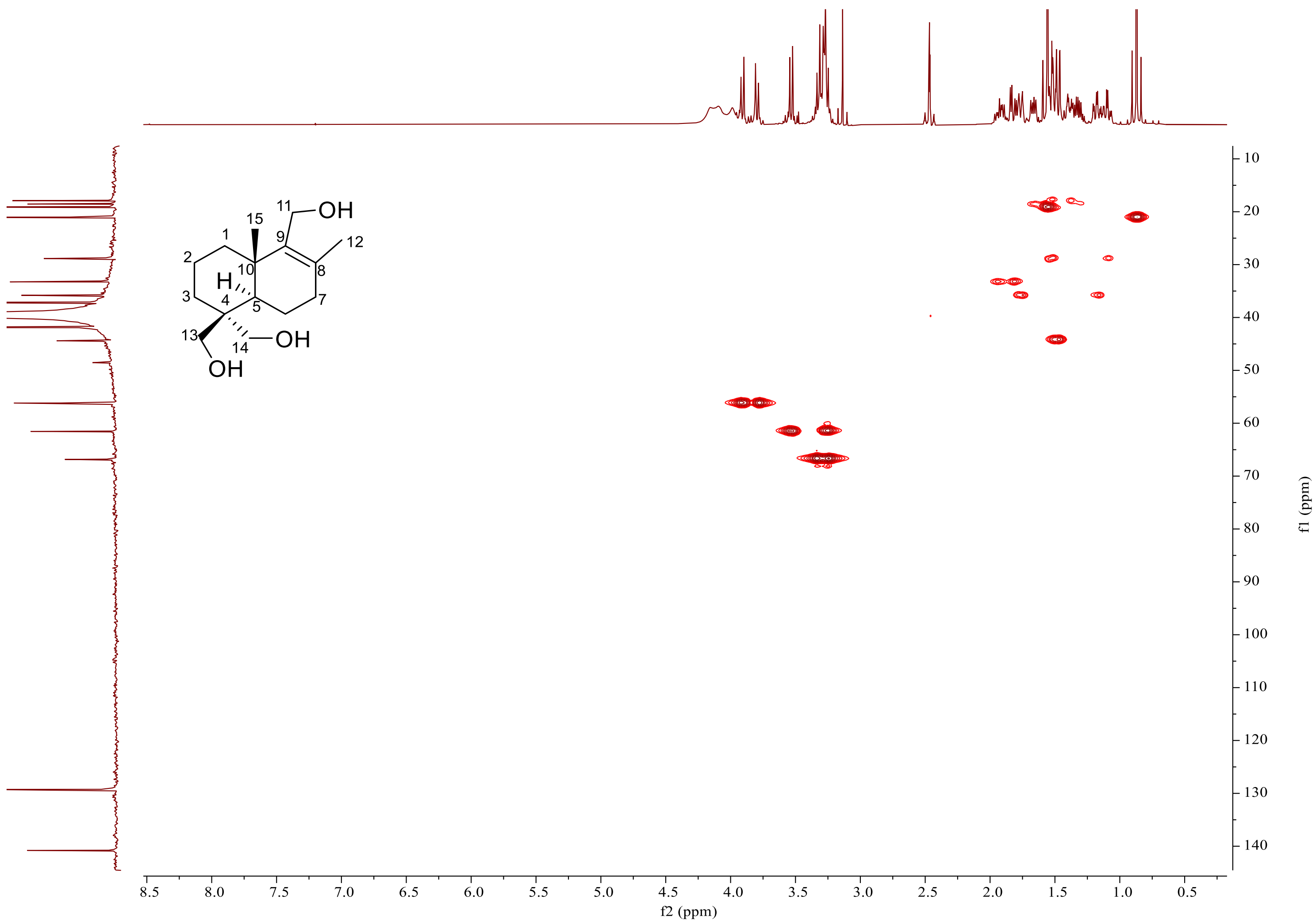

Figure S77. The HSQC spectrum of ustidrimane I (**15**) in DMSO-d_6.

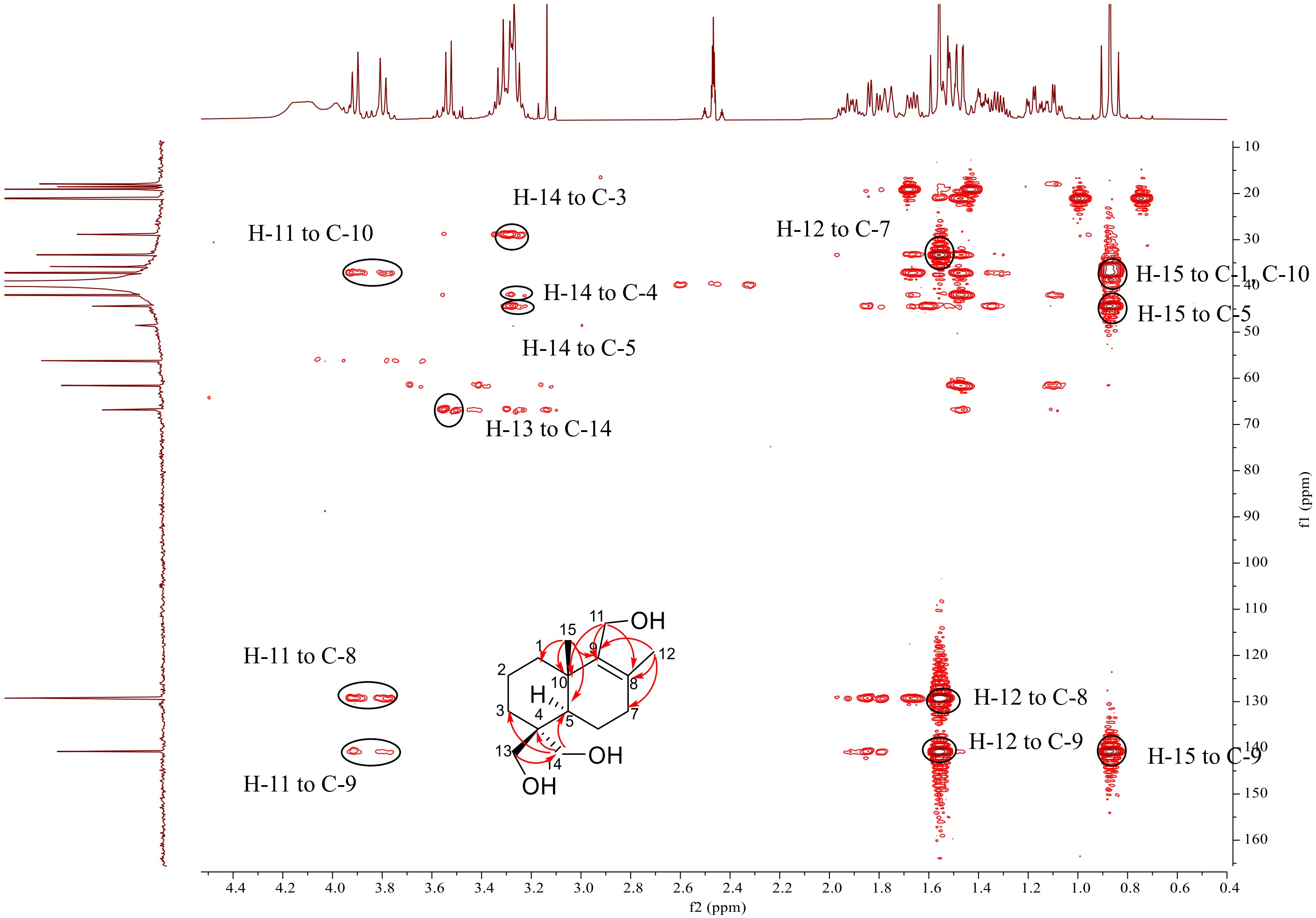

Figure S78. The HMBC spectrum of ustidrimane I (**15**) in DMSO-d_6.

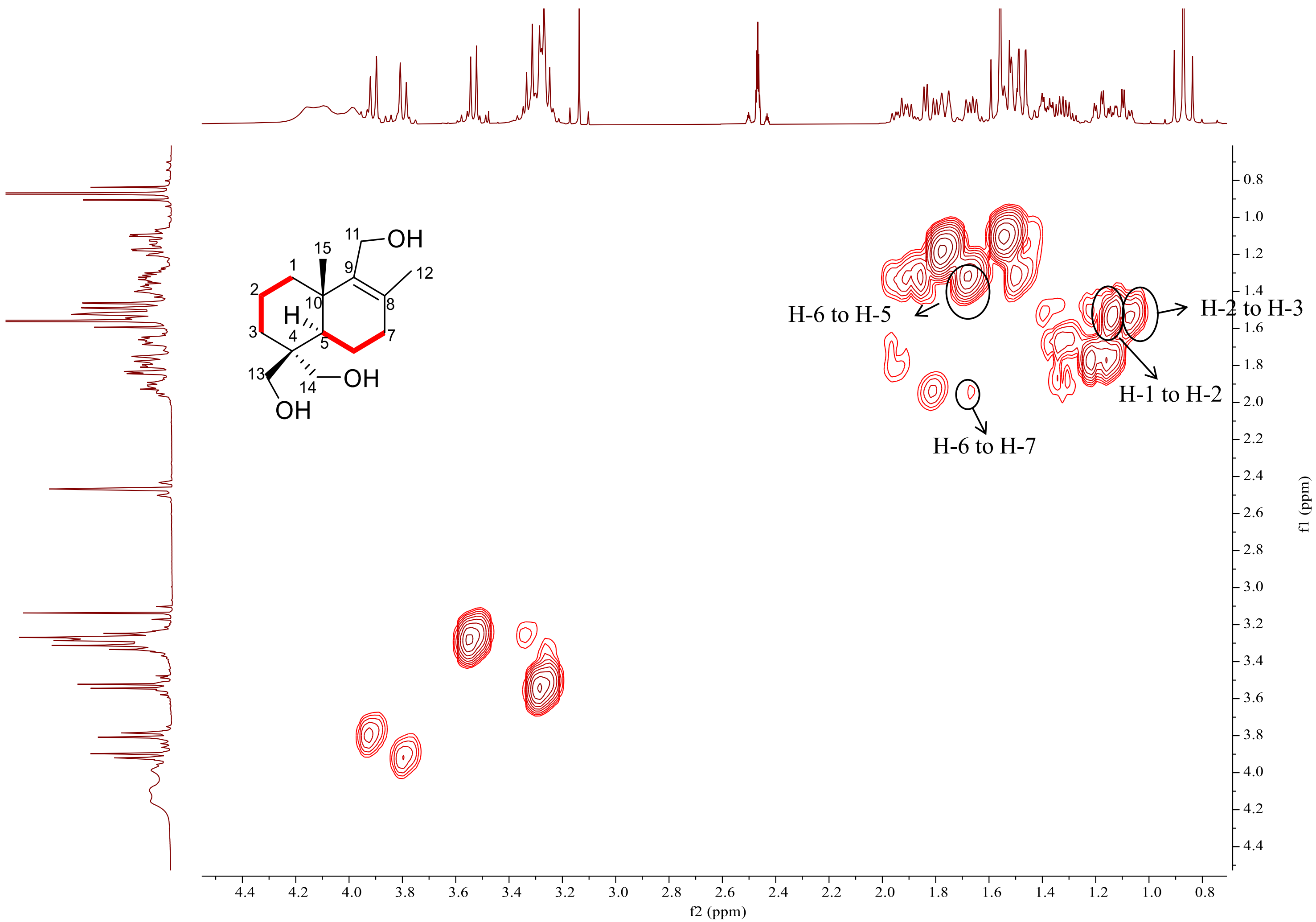

Figure S79. The ^{1}H-^{1}H COSY spectrum of ustidrimane I (**15**) in DMSO-d_6.

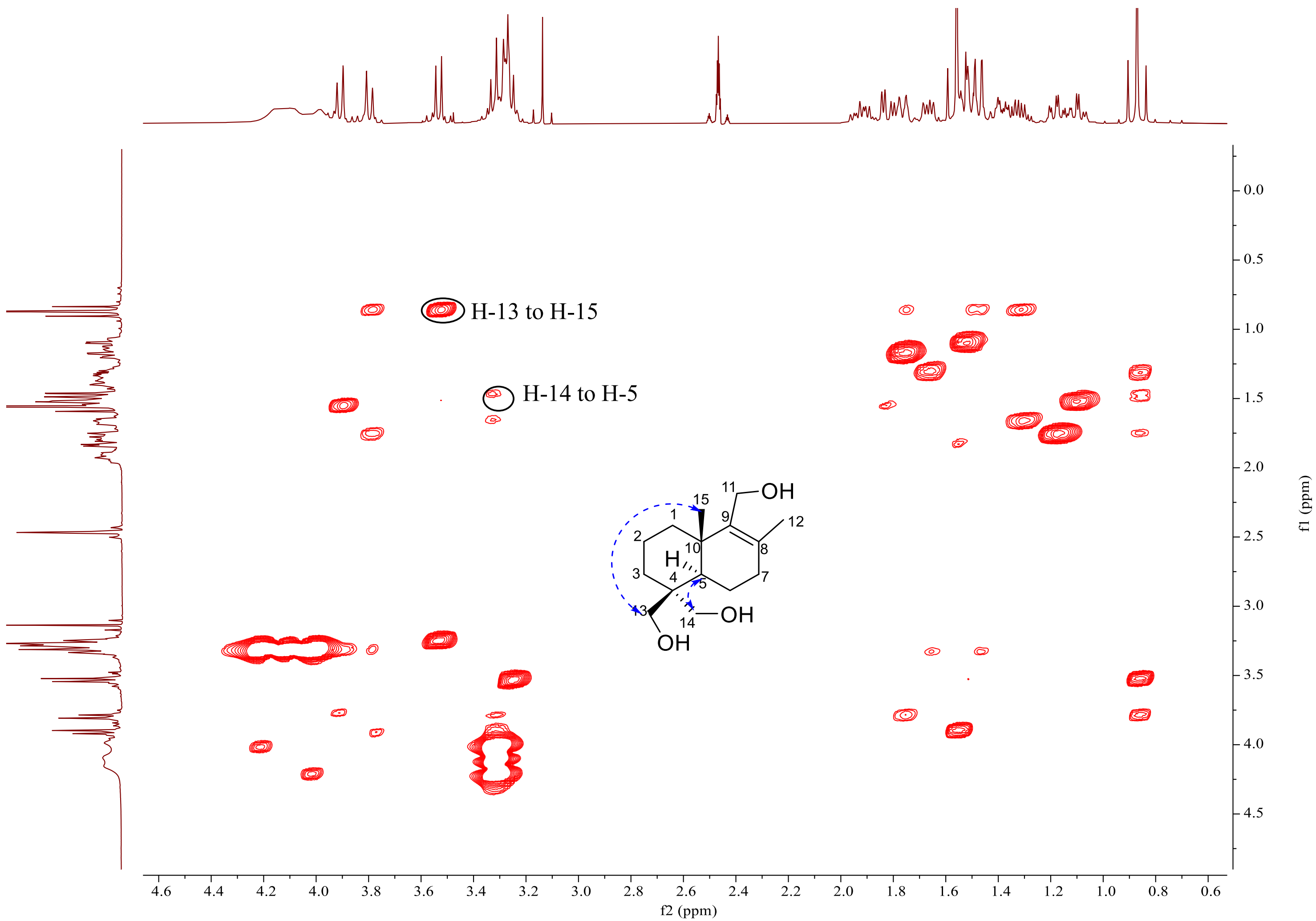

Figure S80. The NOESY spectrum of ustidrimane I (**15**) in DMSO-d_6.

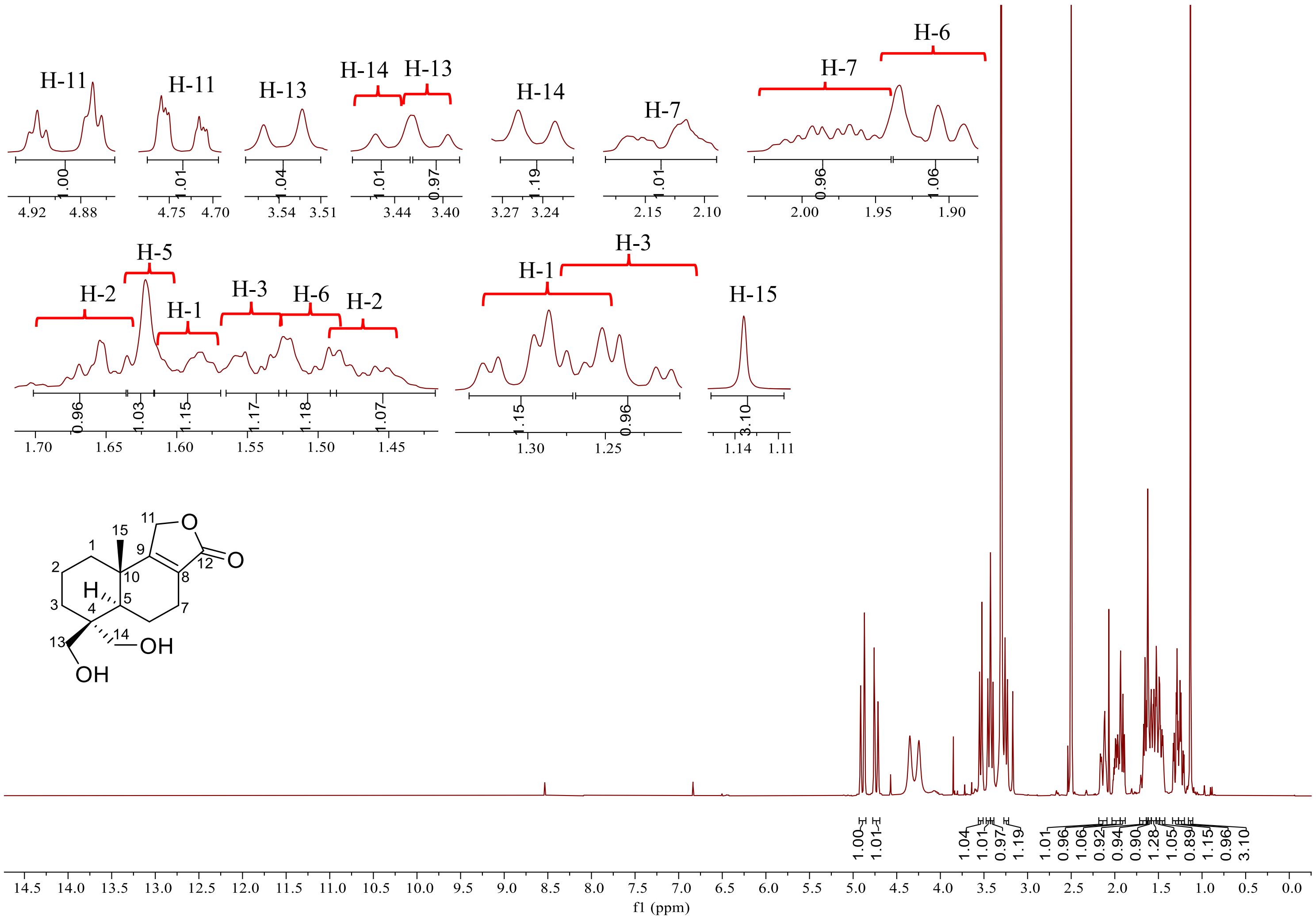

Figure S81. The ^{1}H NMR spectrum of ustidrimane J (**16**) in DMSO-d_6 (400 MHz).

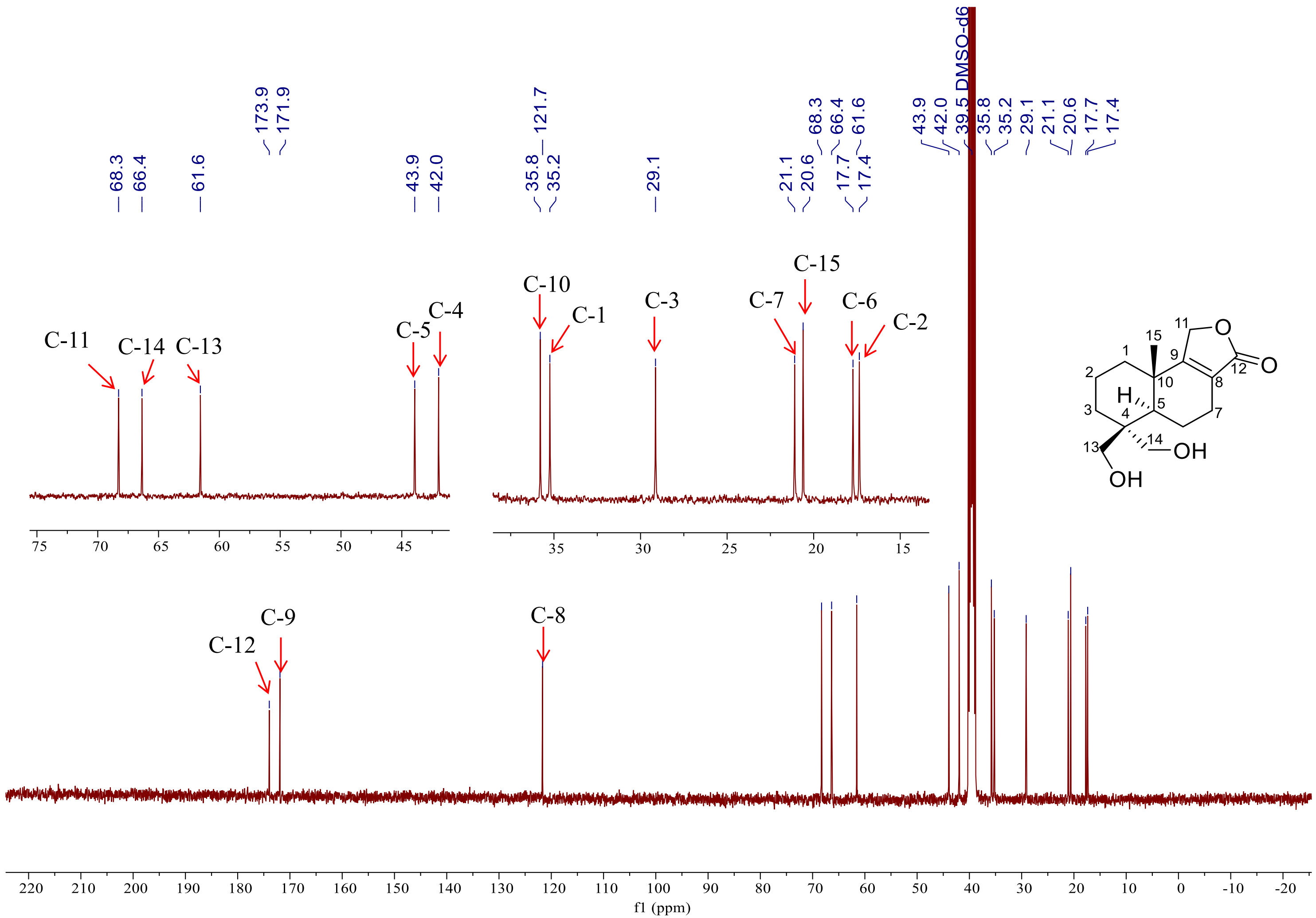

Figure S82. The ^{13}C NMR spectrum of ustidrimane J (**16**) in DMSO-d_6 (100 MHz).

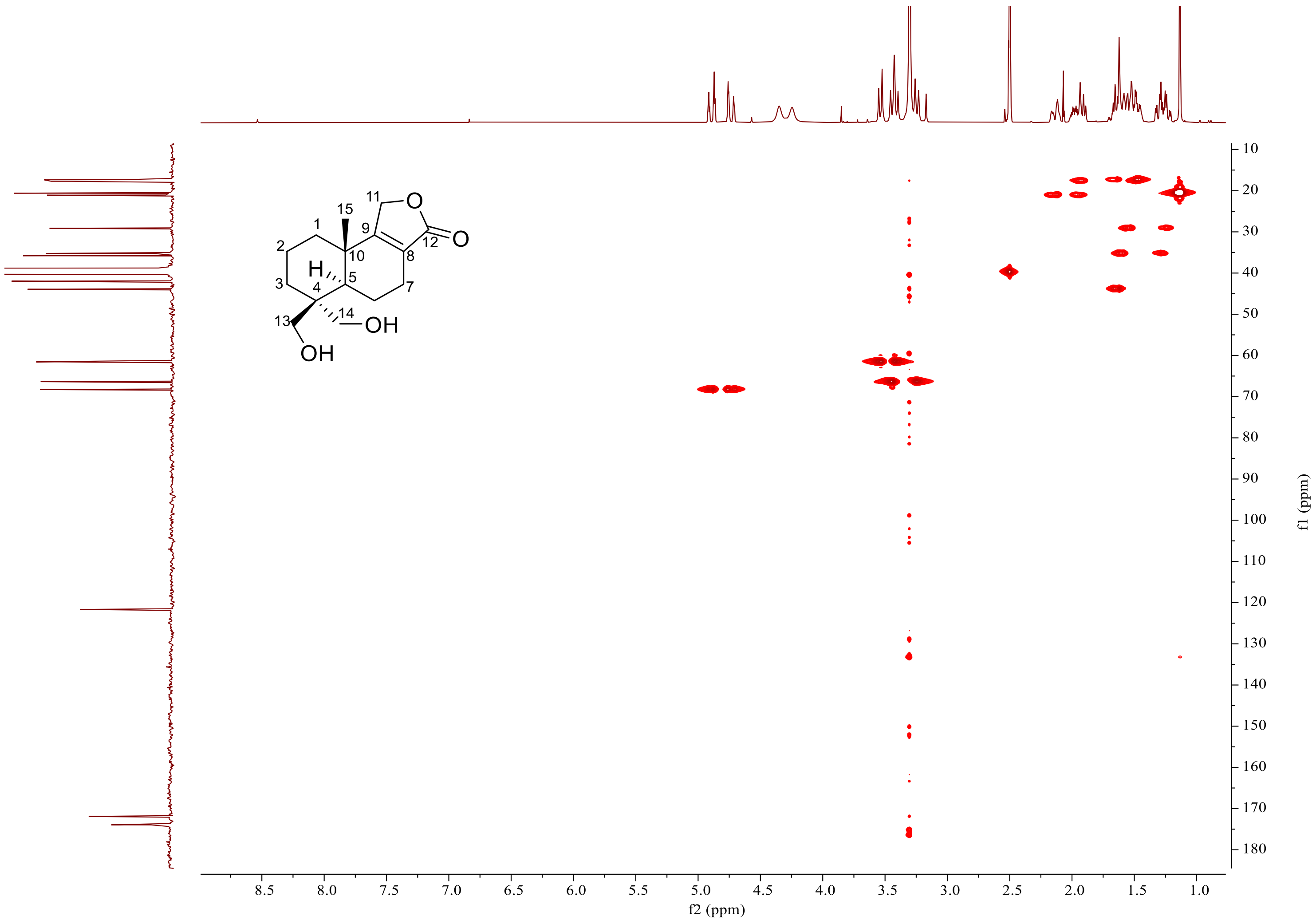

Figure S83. The HSQC spectrum of ustidrimane J (**16**) in DMSO-d_6.

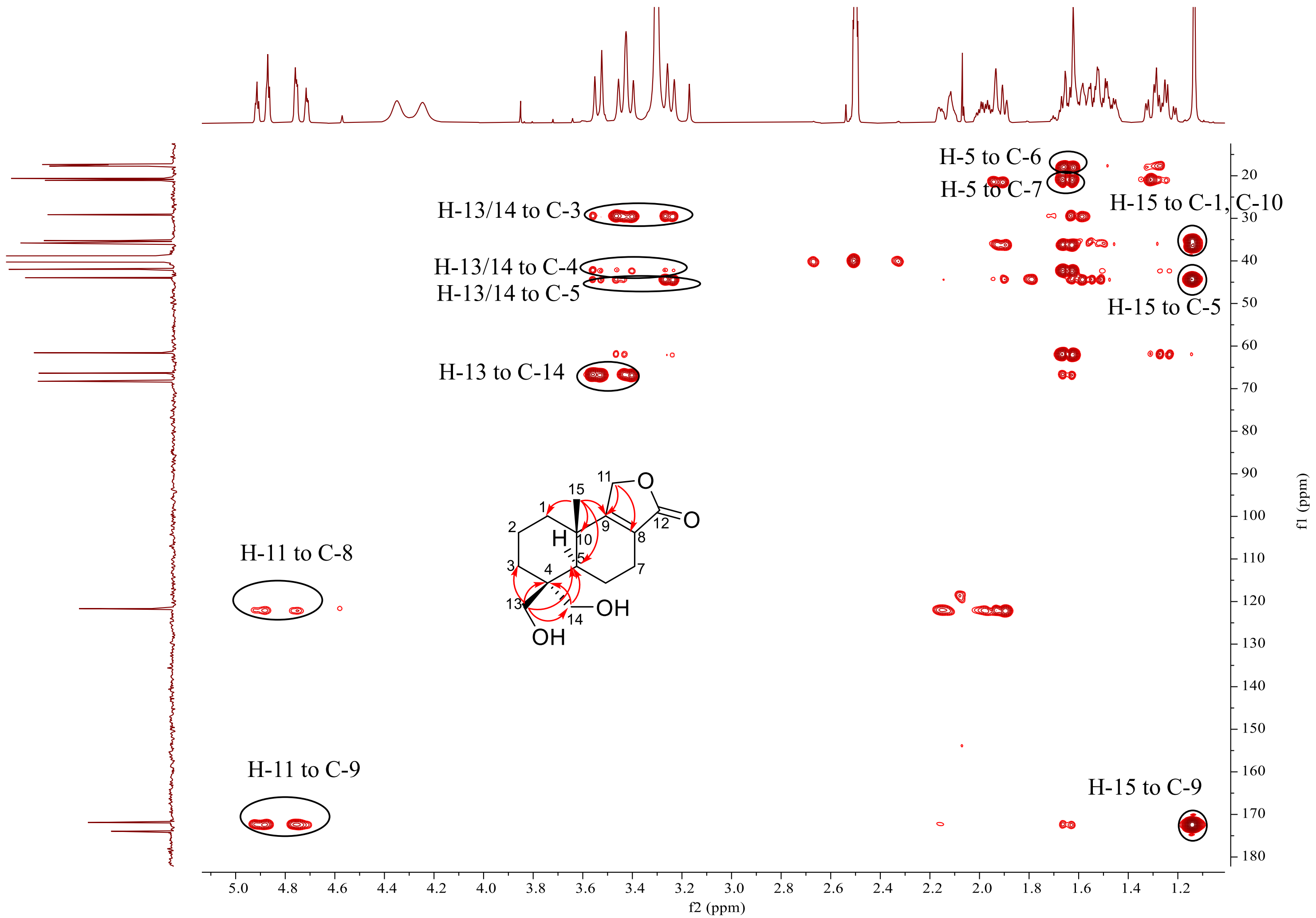

Figure S84. The HMBC spectrum of ustidrimane J (**16**) in DMSO-d_6.

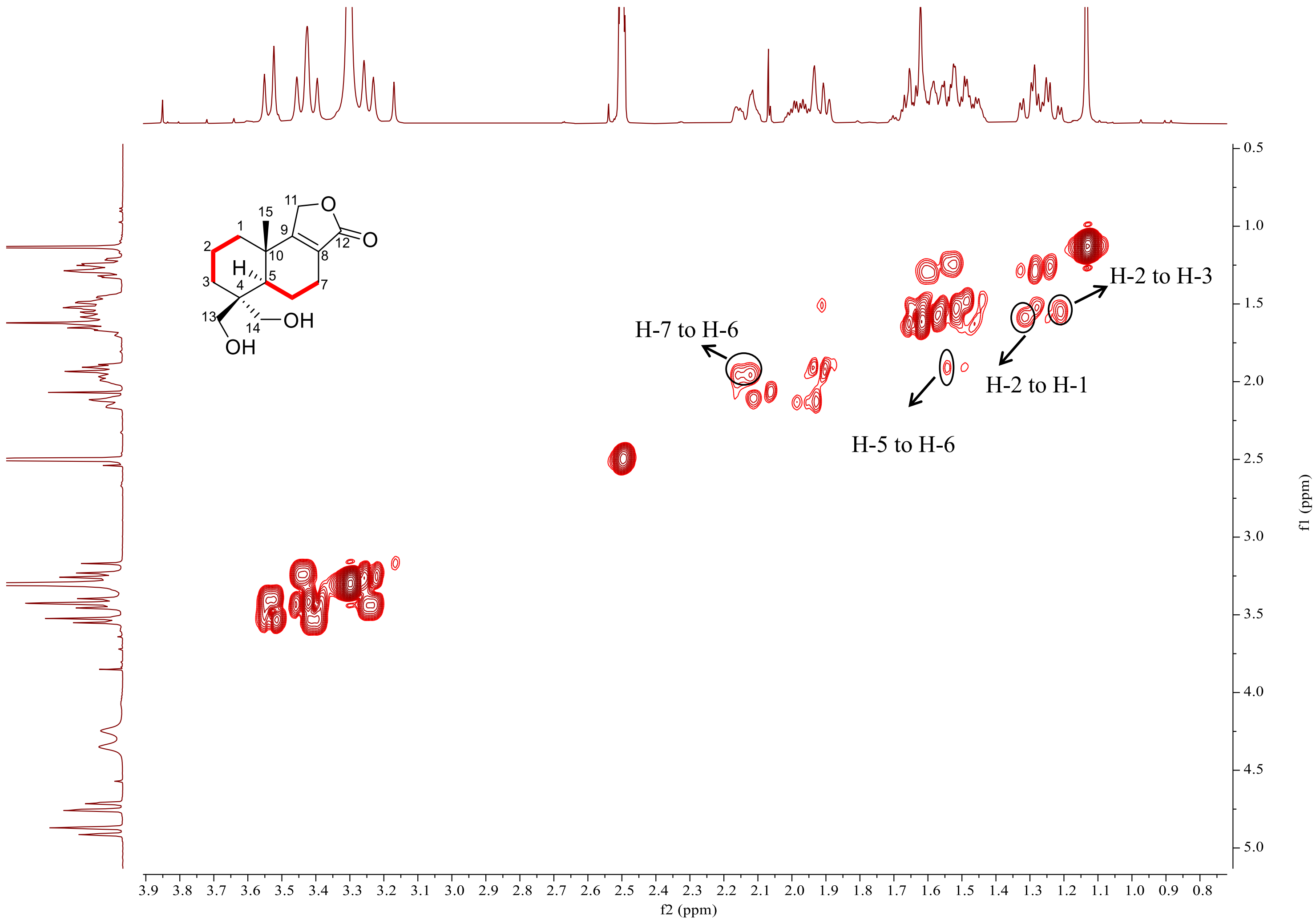

Figure S85. The ^{1}H-^{1}H COSY spectrum of ustidrimane J (**16**) in DMSO-d_6.

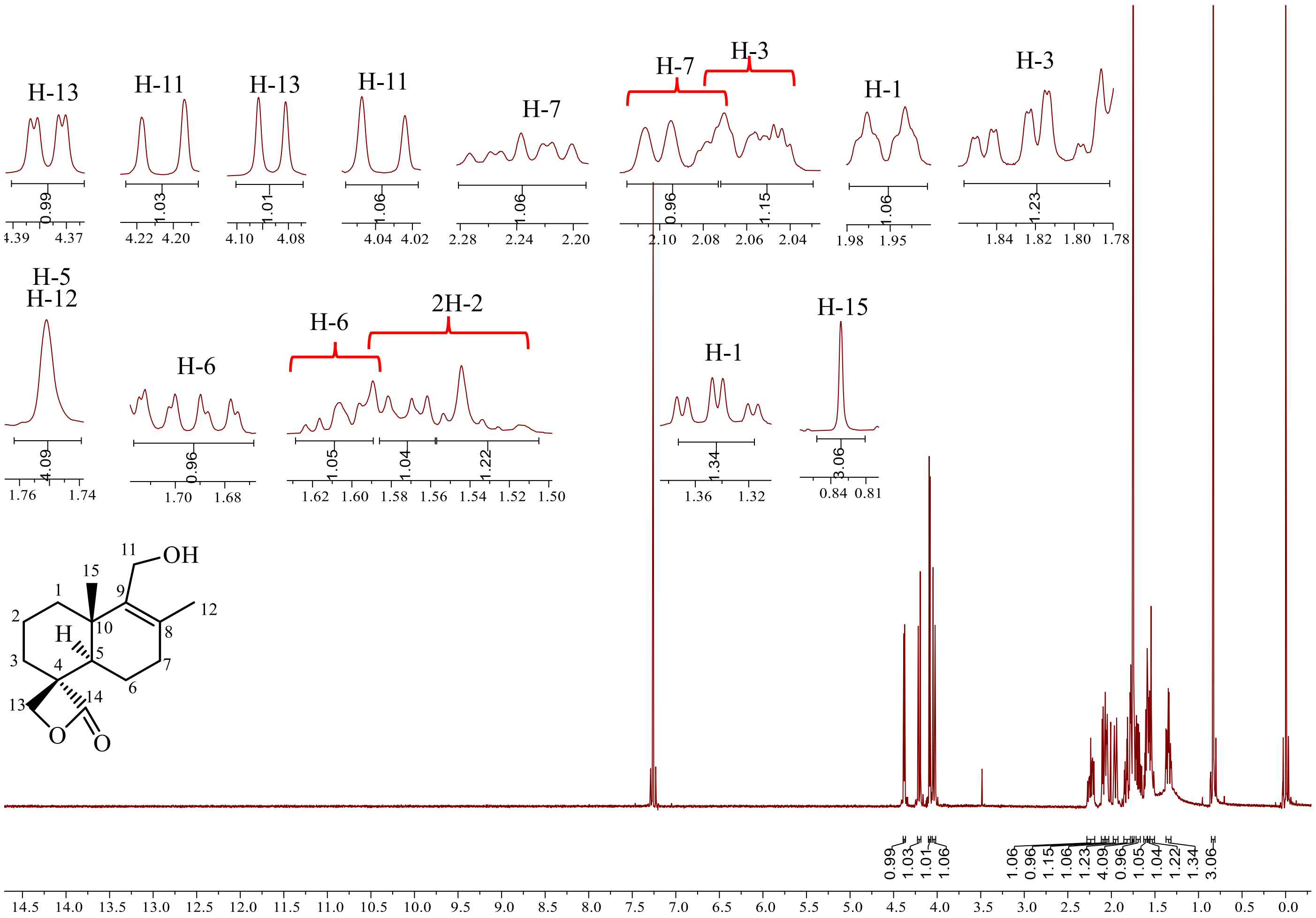

Figure S86. The ^{1}H NMR spectrum of ustidrimane K (**17**) in $CDCl_3$ (400 MHz).

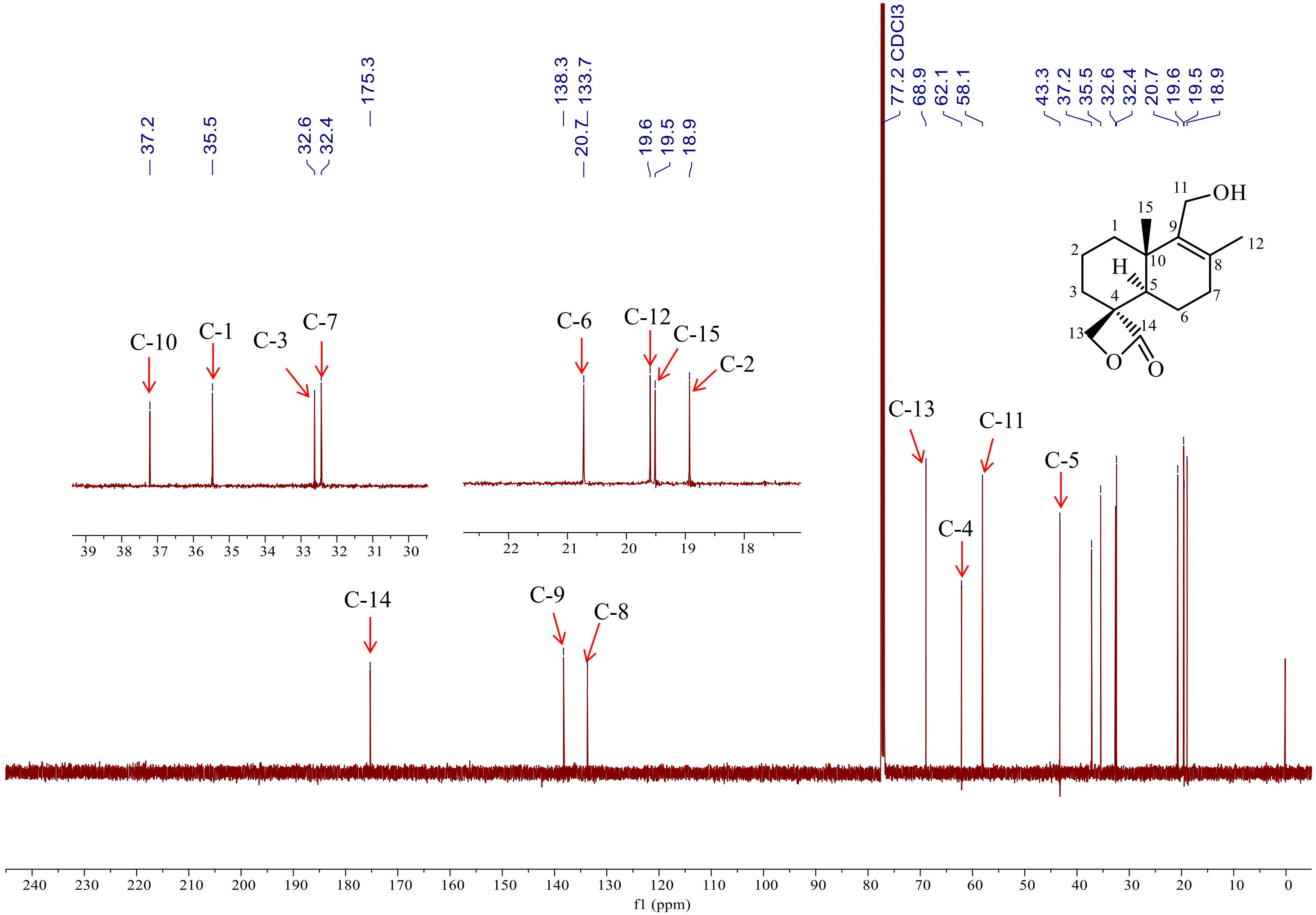

Figure S87. The ^{13}C NMR spectrum of ustidrimane K (**17**) in $CDCl_3$ (100 MHz).

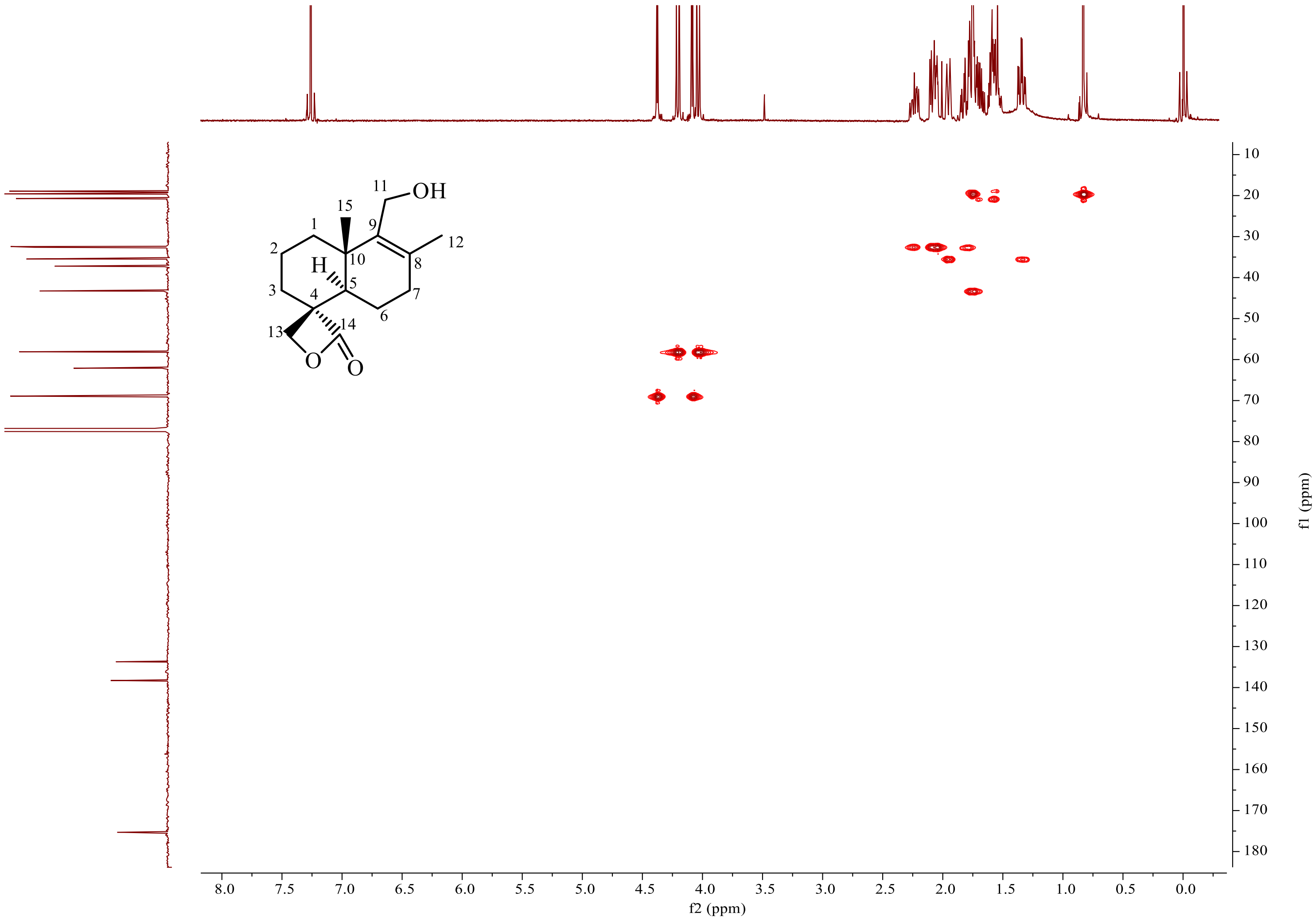

Figure S88. The HSQC spectrum of ustidrimane K (**17**) in $CDCl_3$.

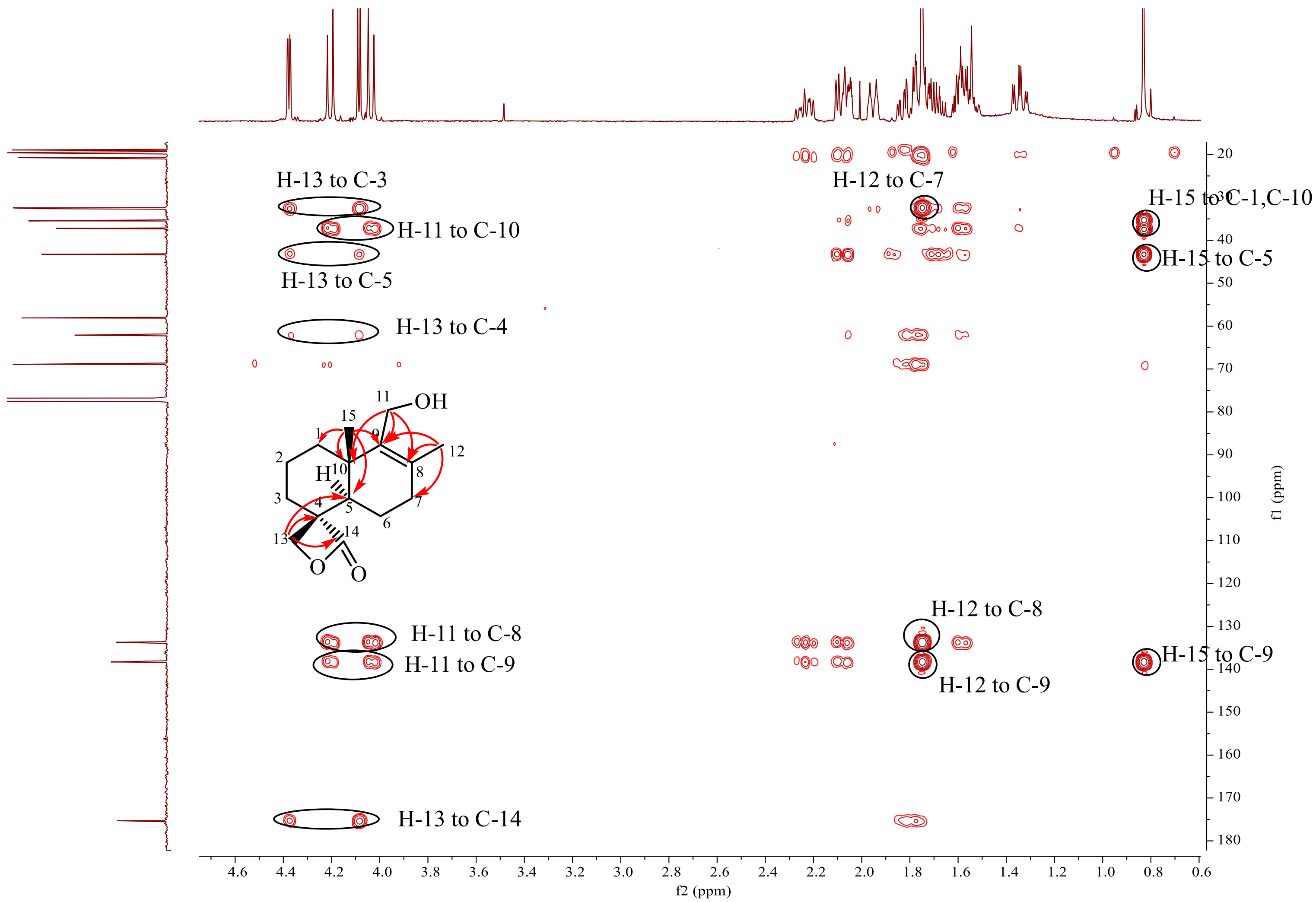

Figure S89. The HMBC spectrum of ustidrimane K (**17**) in $CDCl_3$.

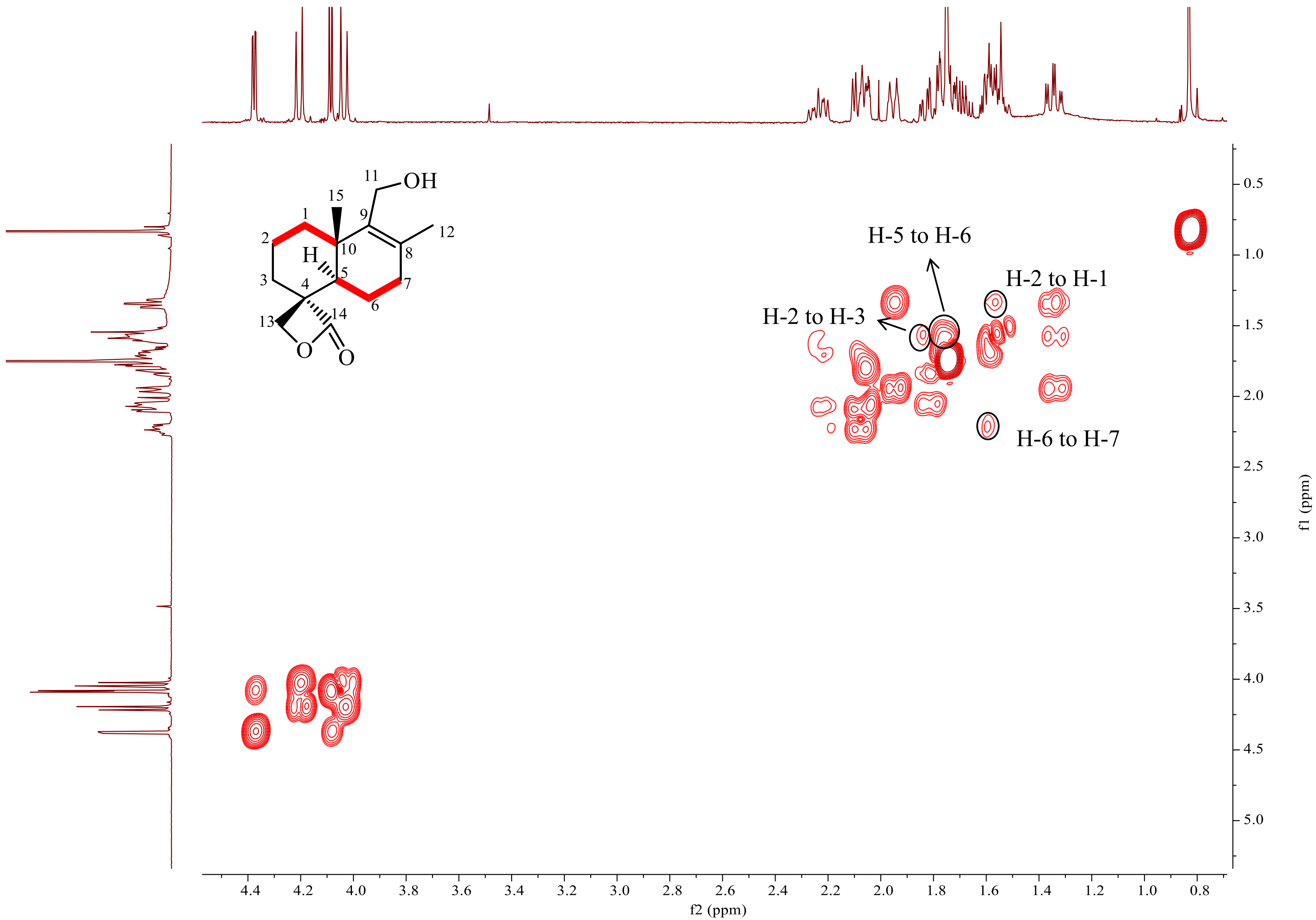

Figure S90. The ^{1}H-^{1}H COSY spectrum of ustidrimane K (**17**) in $CDCl_3$.

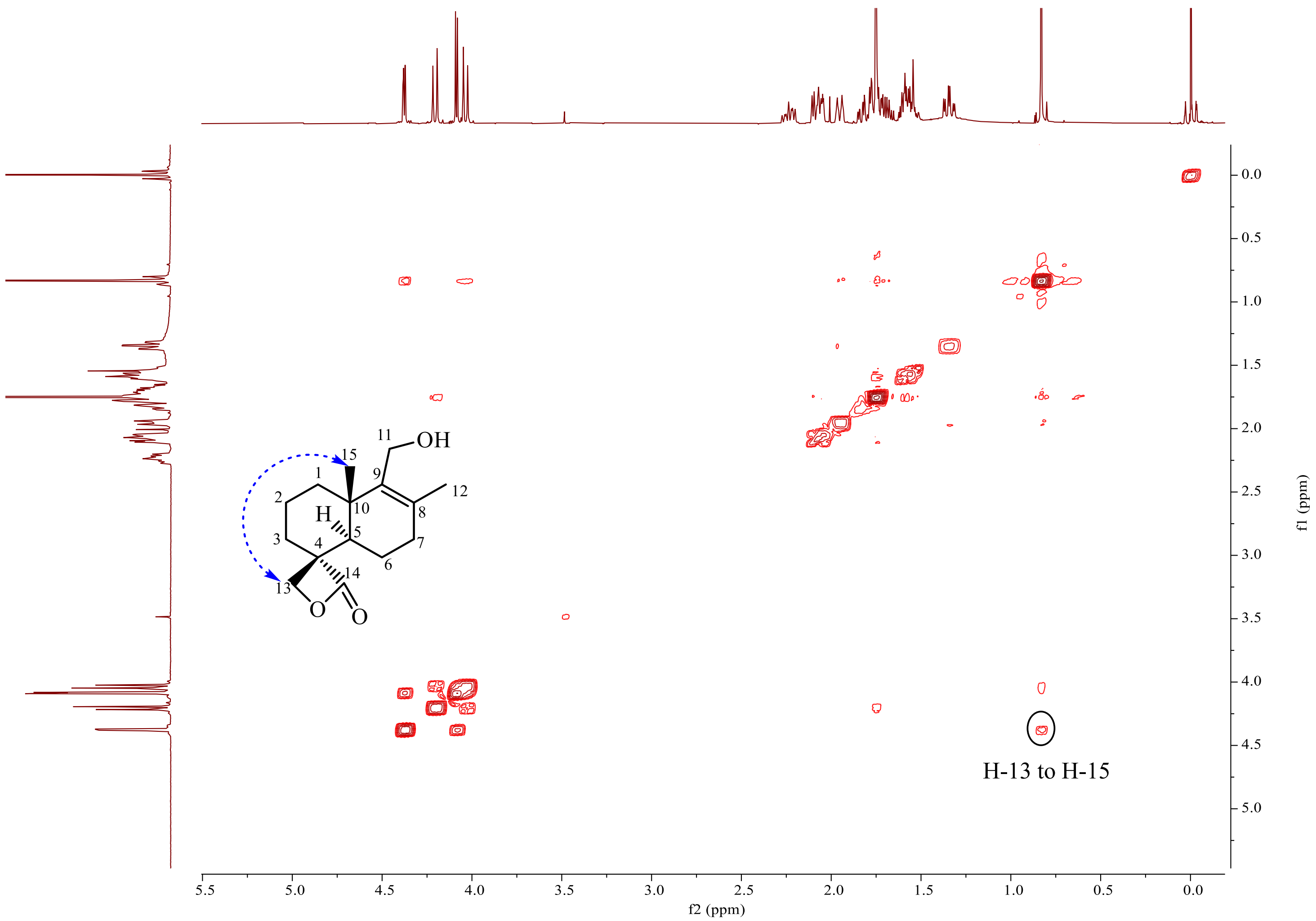

Figure S91. The NOESY spectrum of ustidrimane K (**17**) in $CDCl_3$.

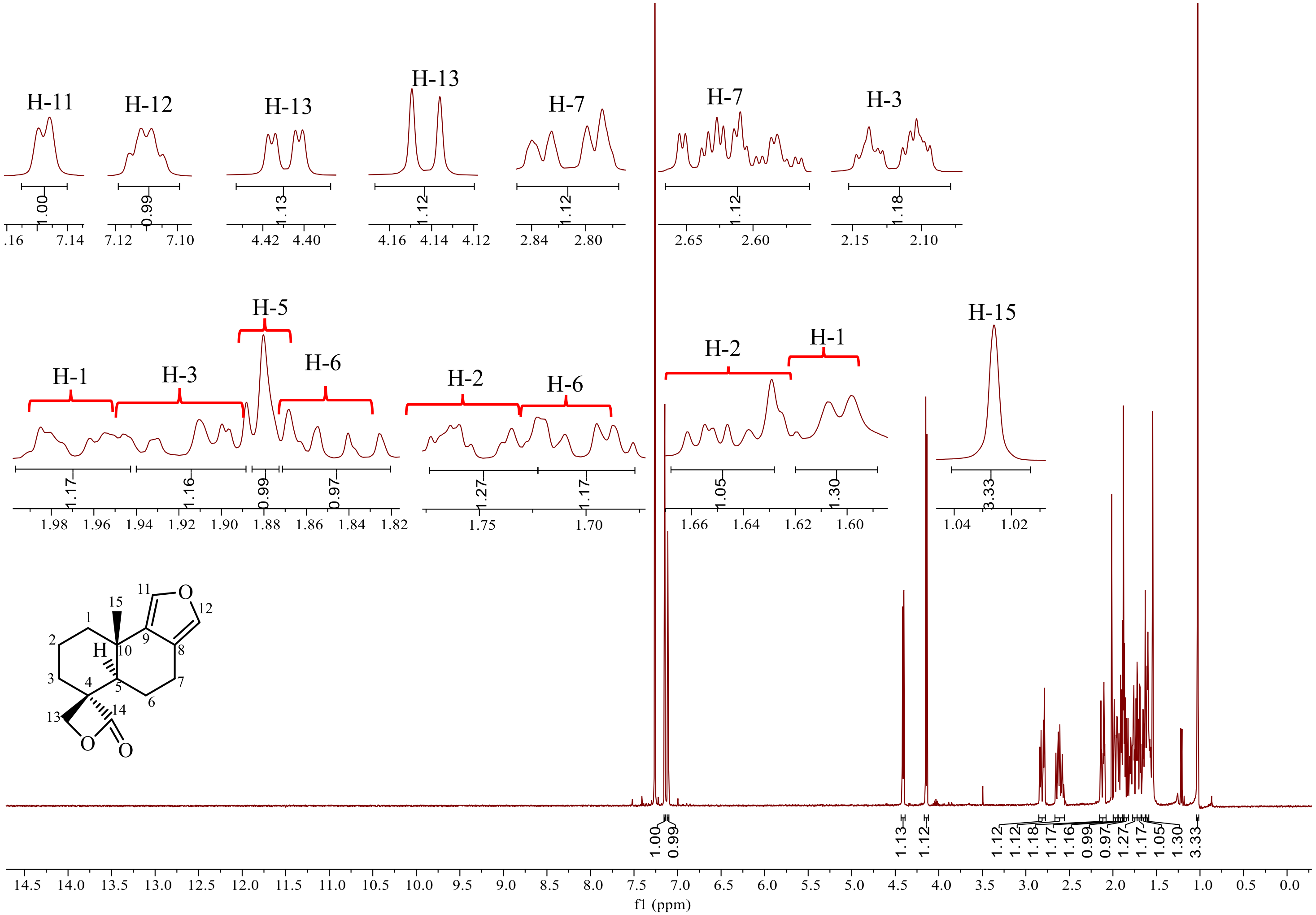

Figure S92. The ^{1}H NMR spectrum of ustidrimane L (**18**) in $CDCl_3$ (400 MHz).

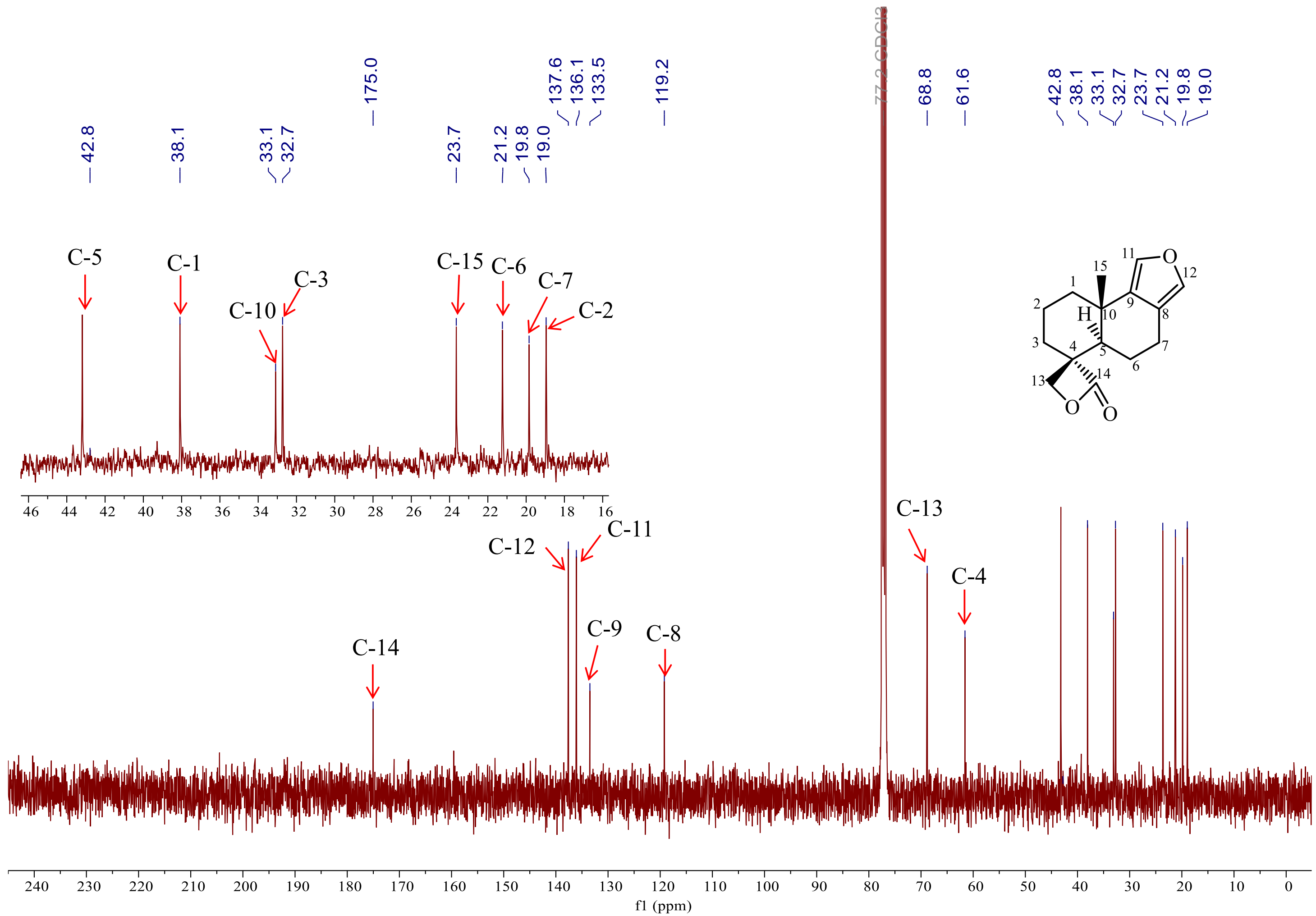

Figure S93. The ^{13}C NMR spectrum of ustidrimane L (**18**) in $CDCl_3$ (100 MHz).

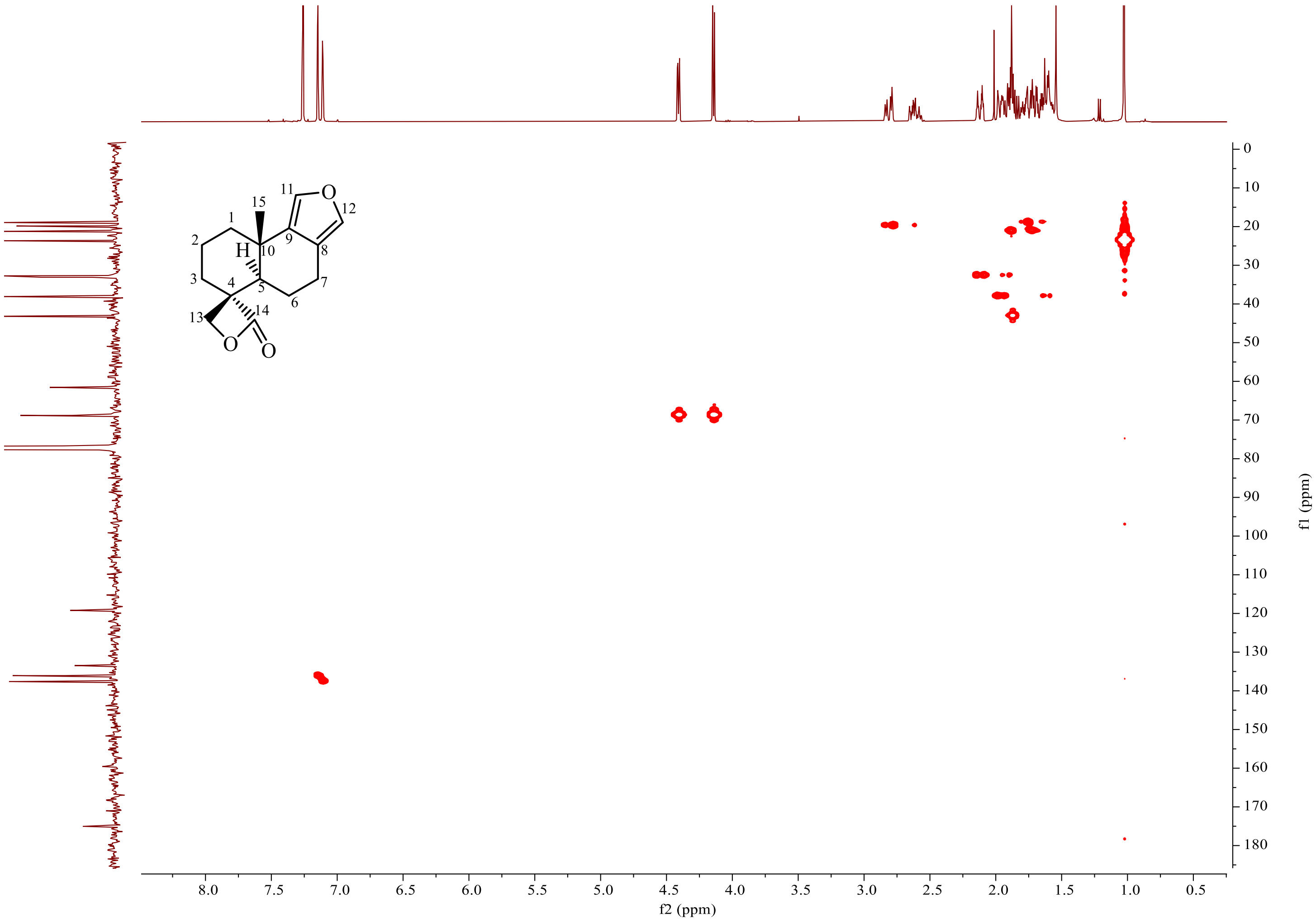

Figure S94. The HSQC spectrum of ustidrimane L (**18**) in $CDCl_3$.

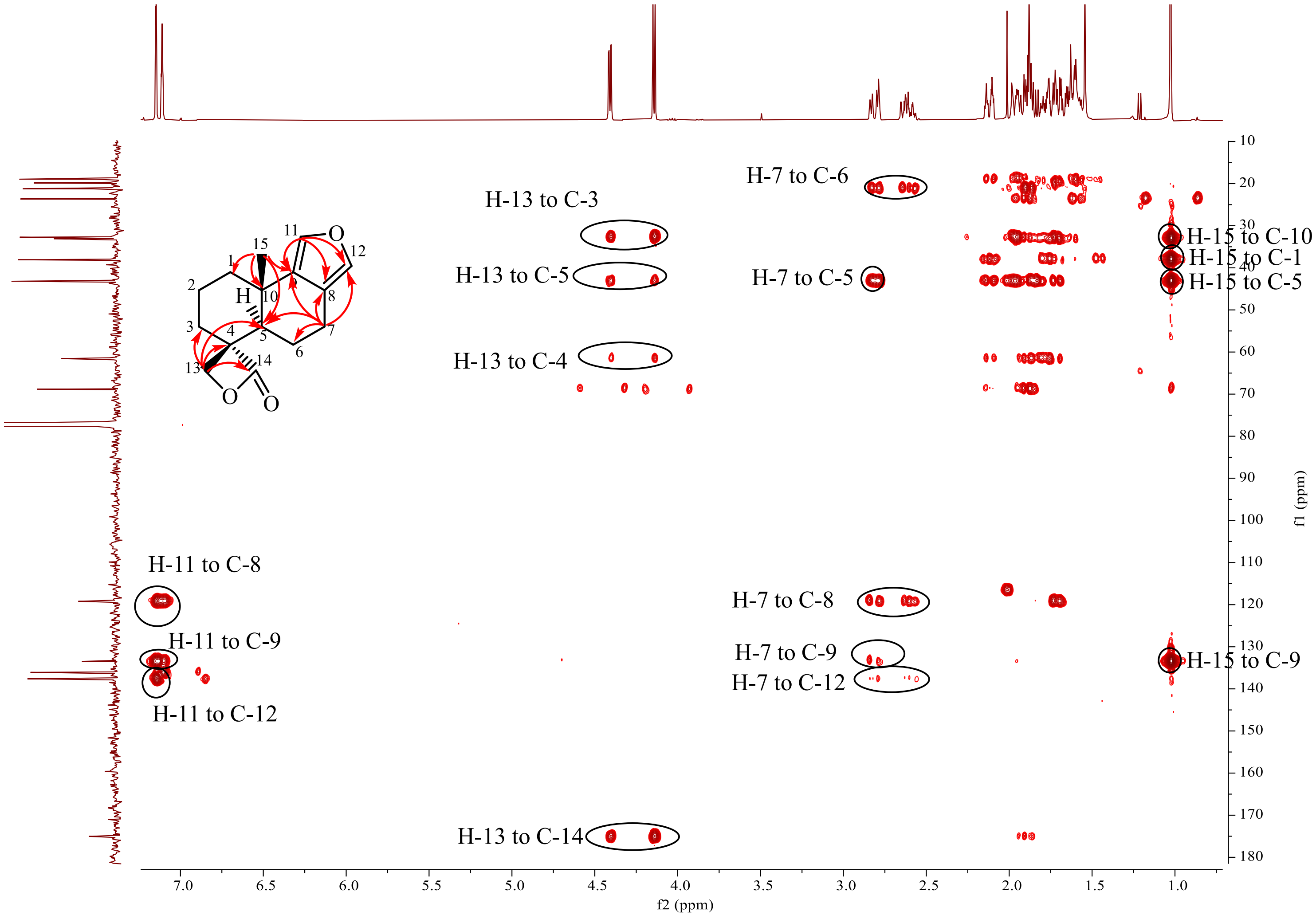

Figure S95. The HMBC spectrum of ustidrimane L (**18**) in $CDCl_3$.

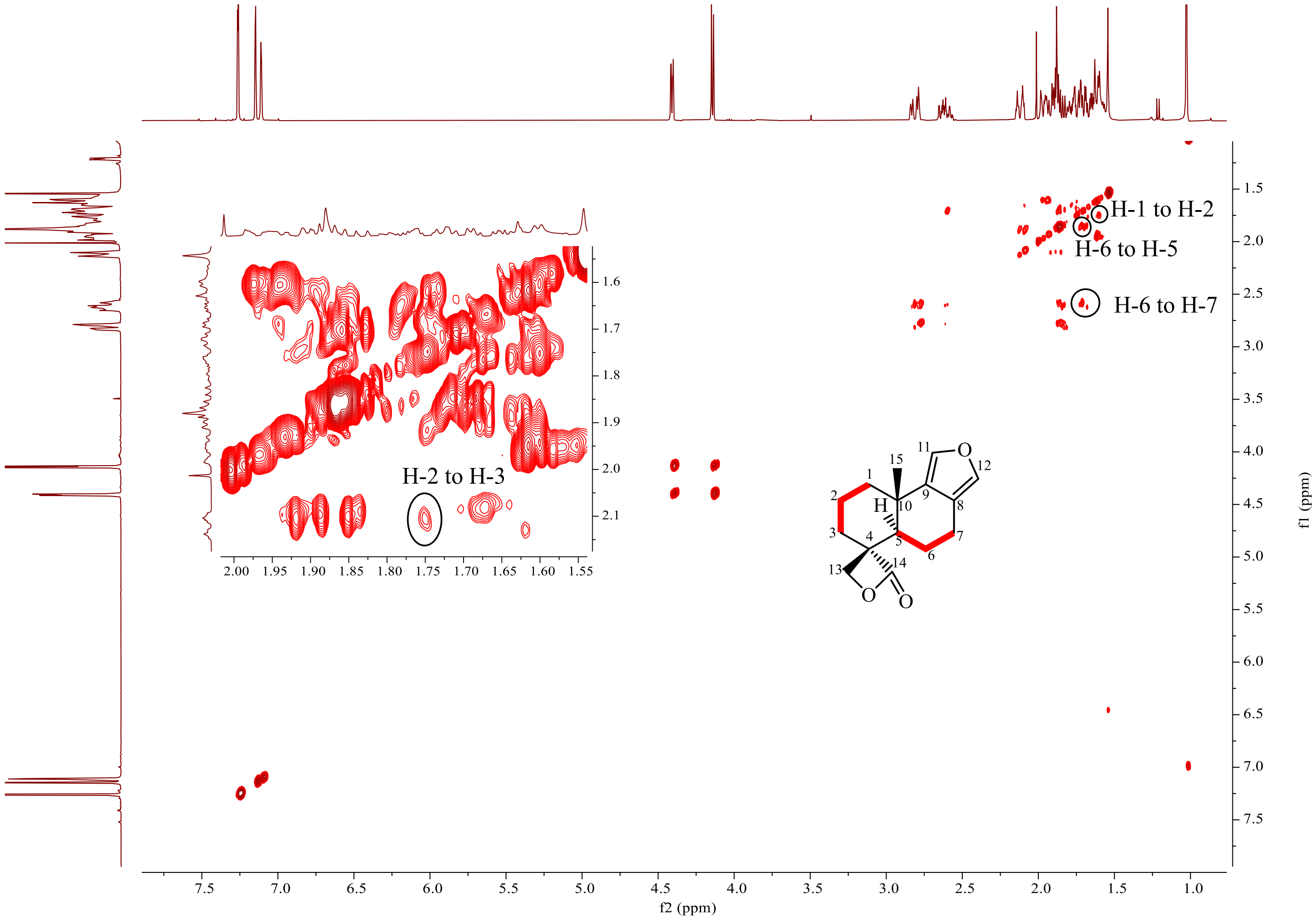

Figure S96. The ^{1}H-^{1}H COSY spectrum of ustidrimane L (**18**) in $CDCl_3$.

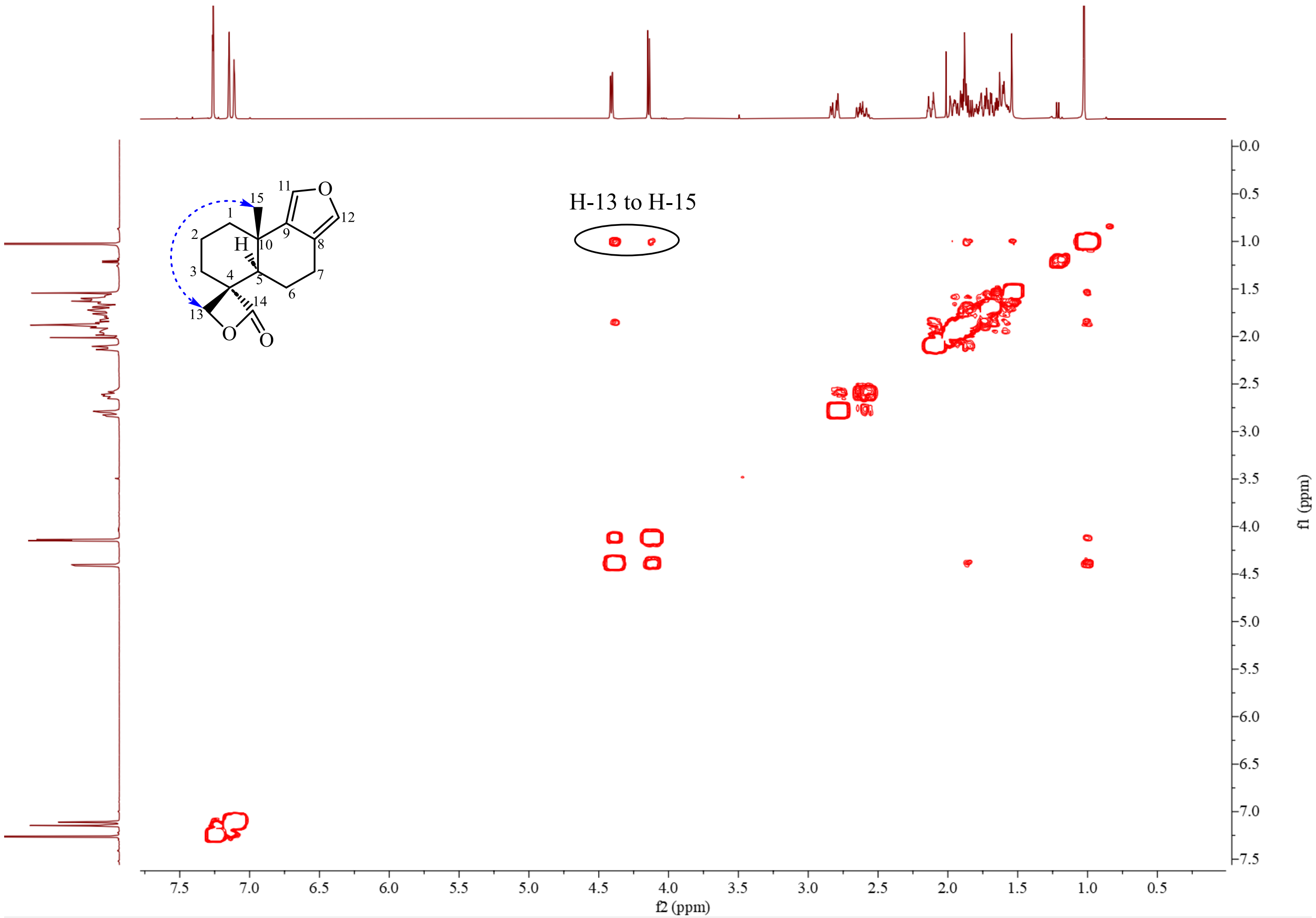

Figure S97. The NOESY spectrum of ustidrimane L (**18**) in $CDCl_3$.

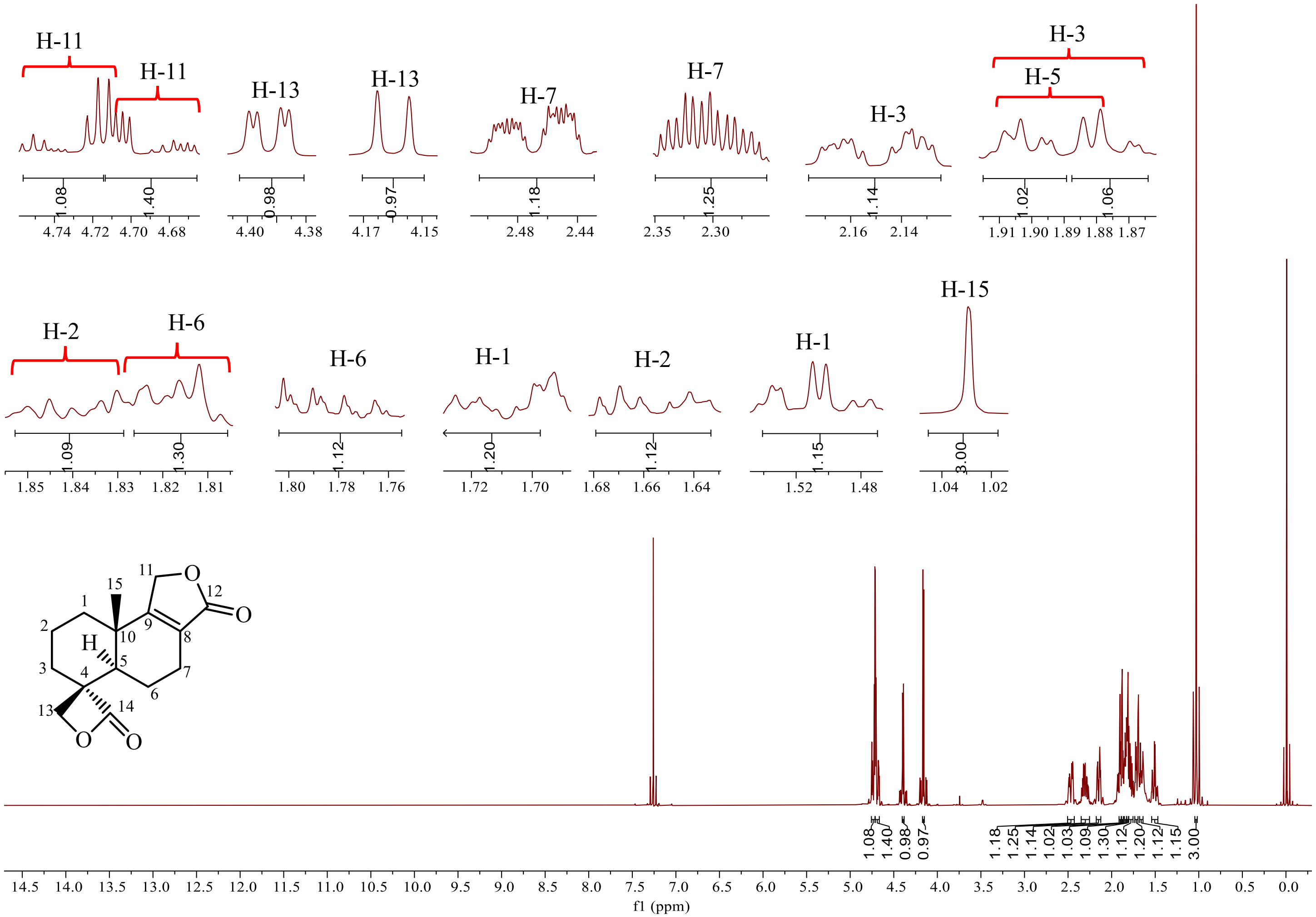

Figure S98. The ^{1}H NMR spectrum of ustidrimane M (**19**) in $CDCl_3$ (400 MHz).

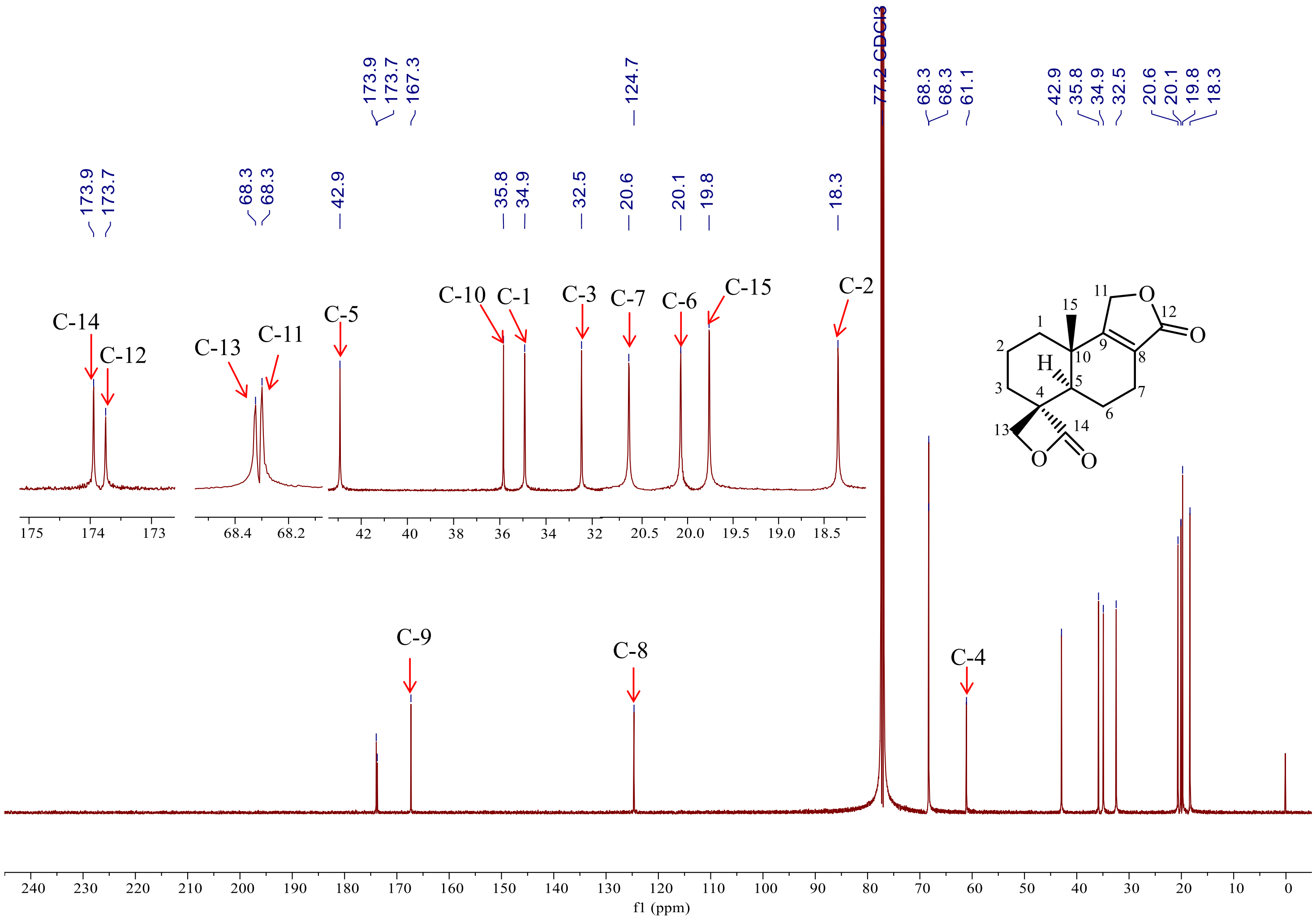

Figure S99. The ^{13}C NMR spectrum of ustidrimane M (**19**) in $CDCl_3$ (100 MHz).

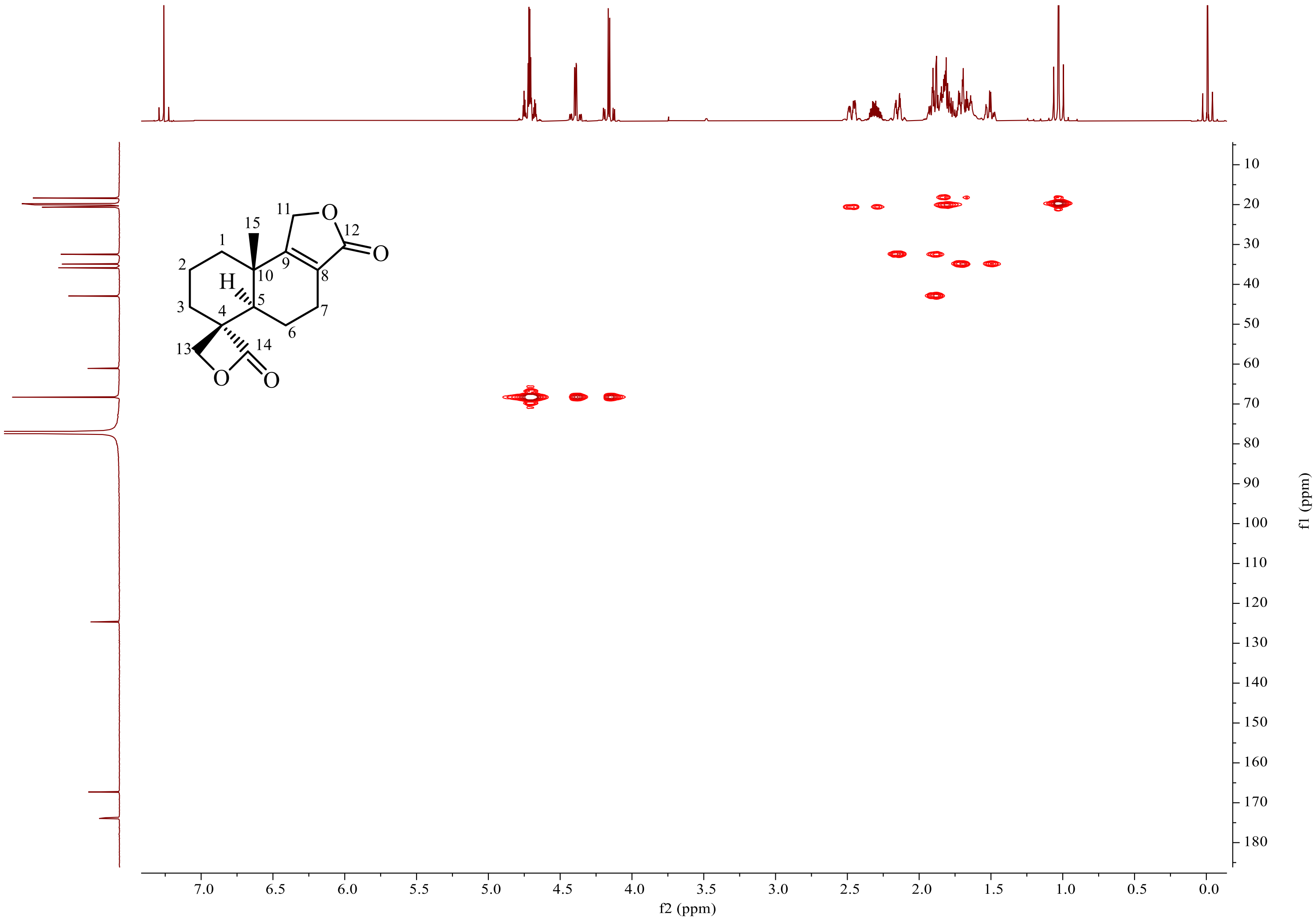

Figure S100. The HSQC spectrum of ustidrimane M (**19**) in $CDCl_3$.

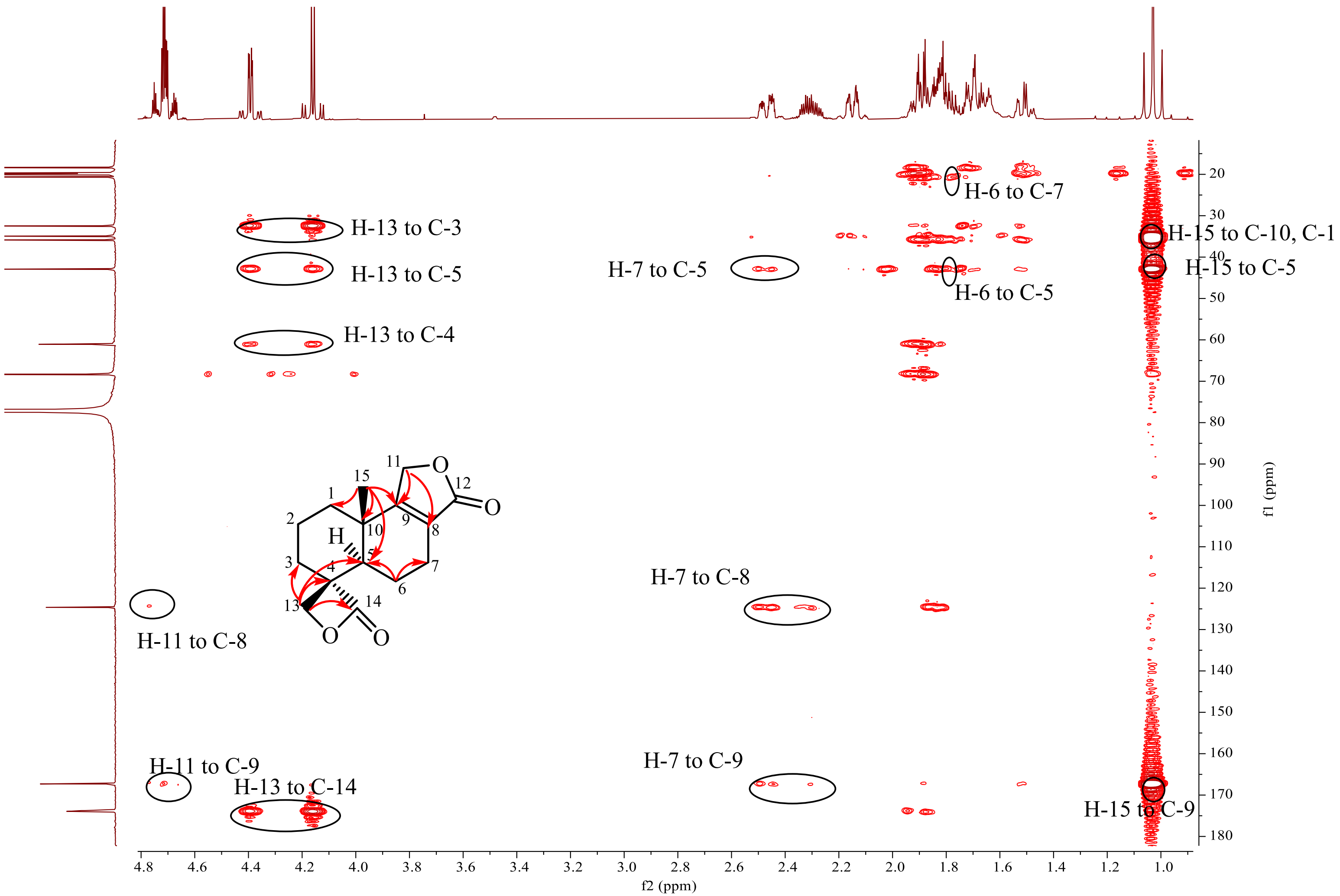

Figure S101. The HMBC spectrum of ustidrimane M (**19**) in $CDCl_3$.

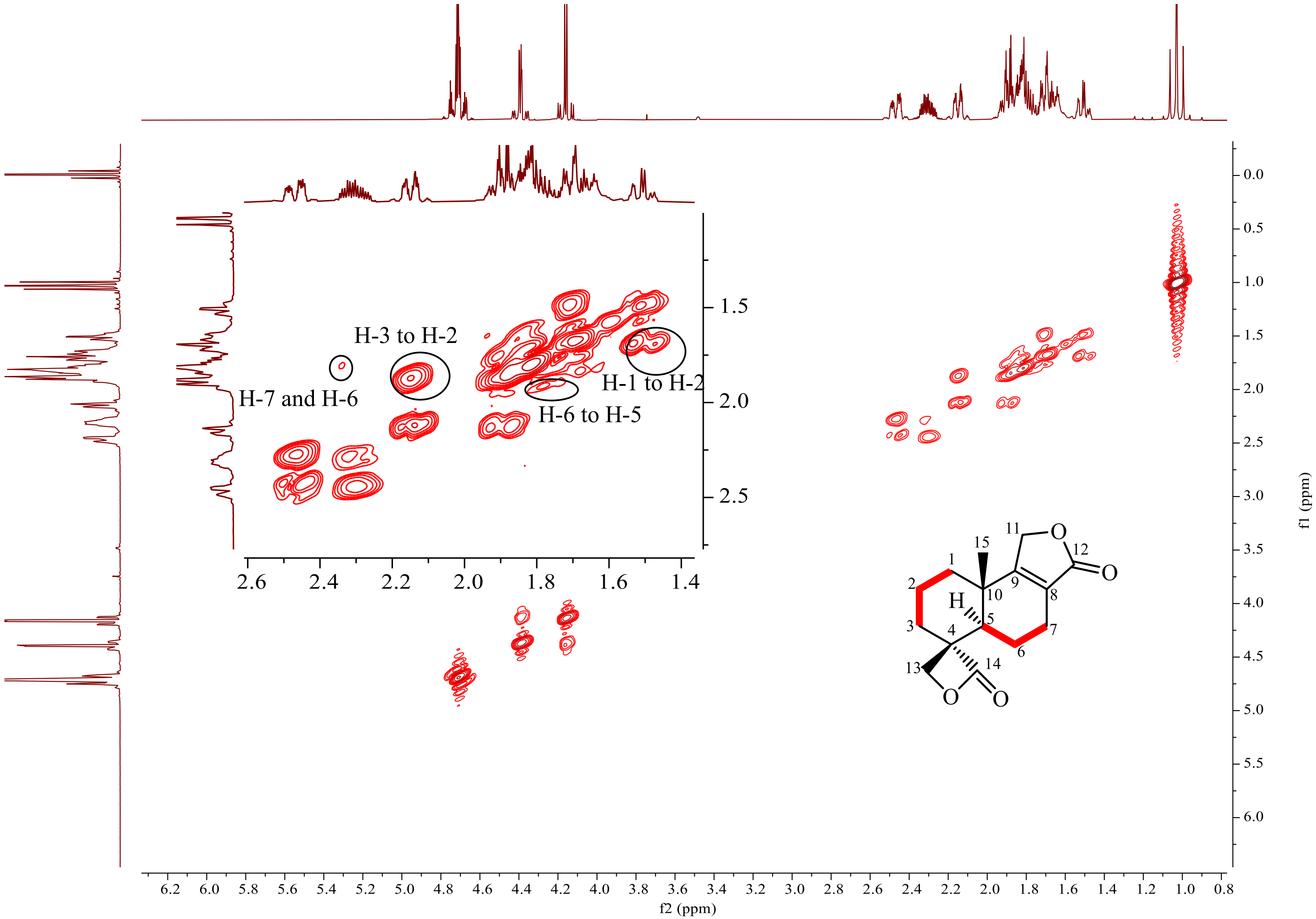

Figure S102. The ^{1}H-^{1}H COSY spectrum of ustidrimane M (**19**) in $CDCl_3$.

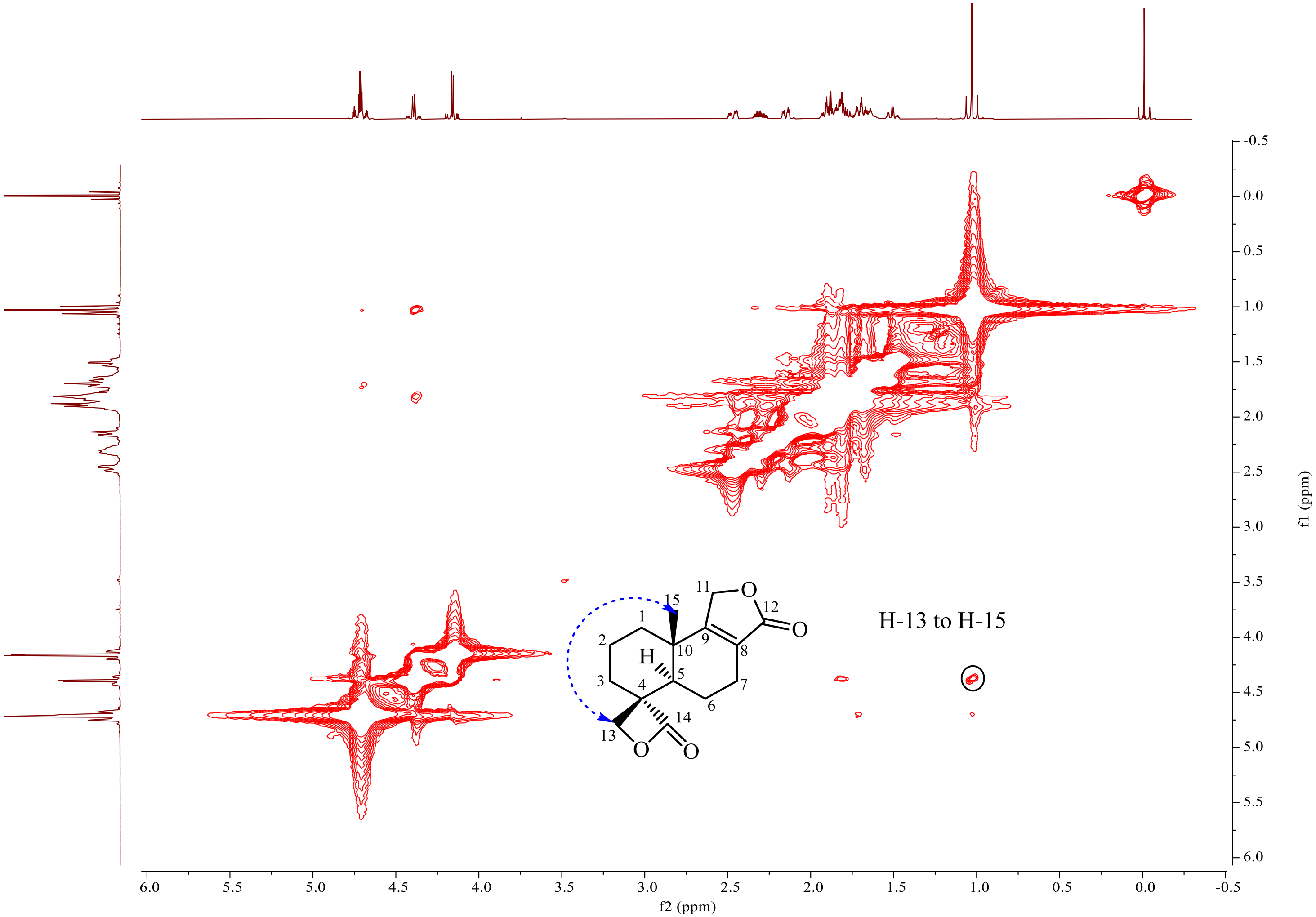

Figure S103. The NOESY spectrum of ustidrimane M (**19**) in $CDCl_3$.

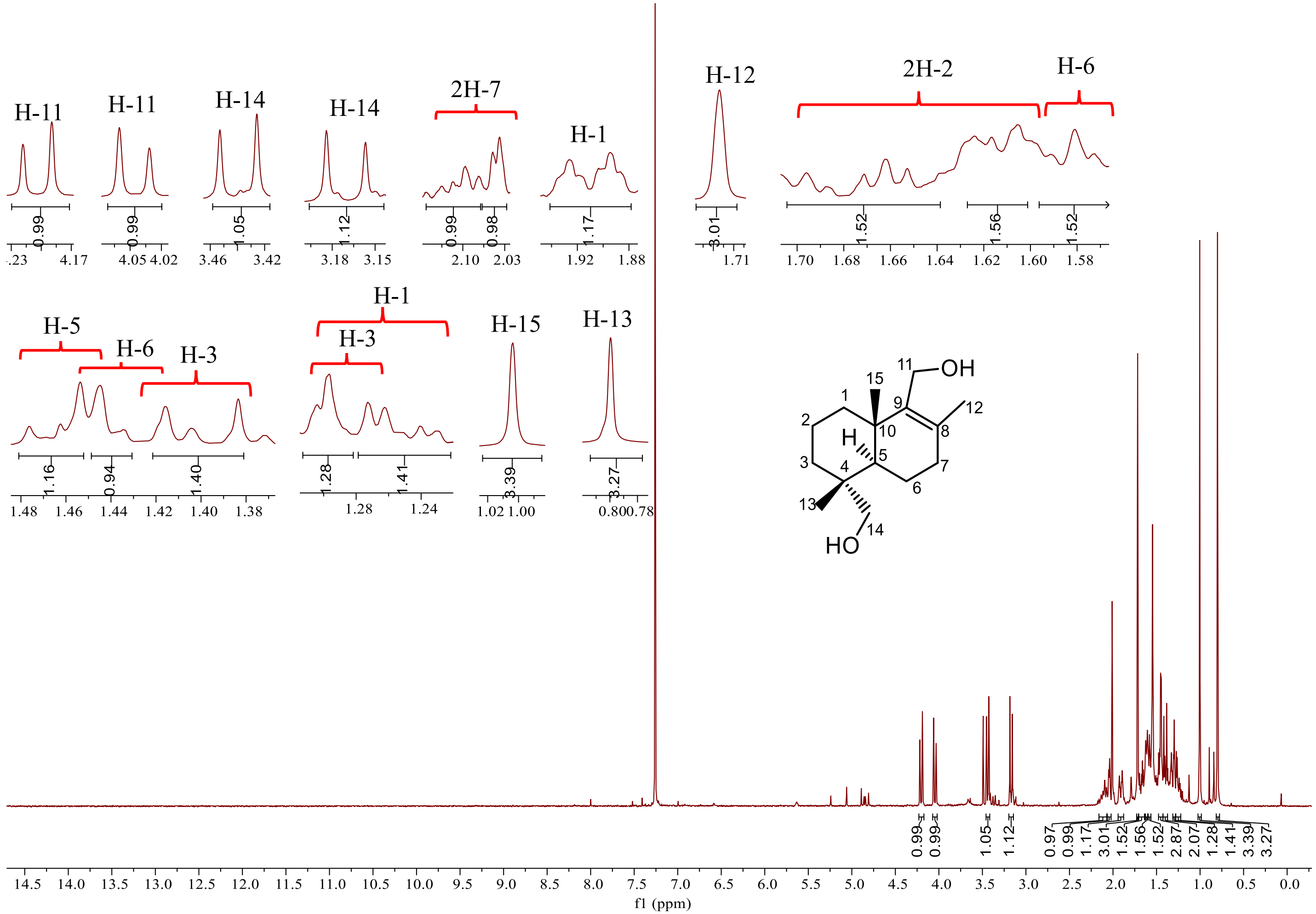

Figure S104. The ^{1}H NMR spectrum of ustidrimane N (**20**) in $CDCl_3$ (400 MHz).

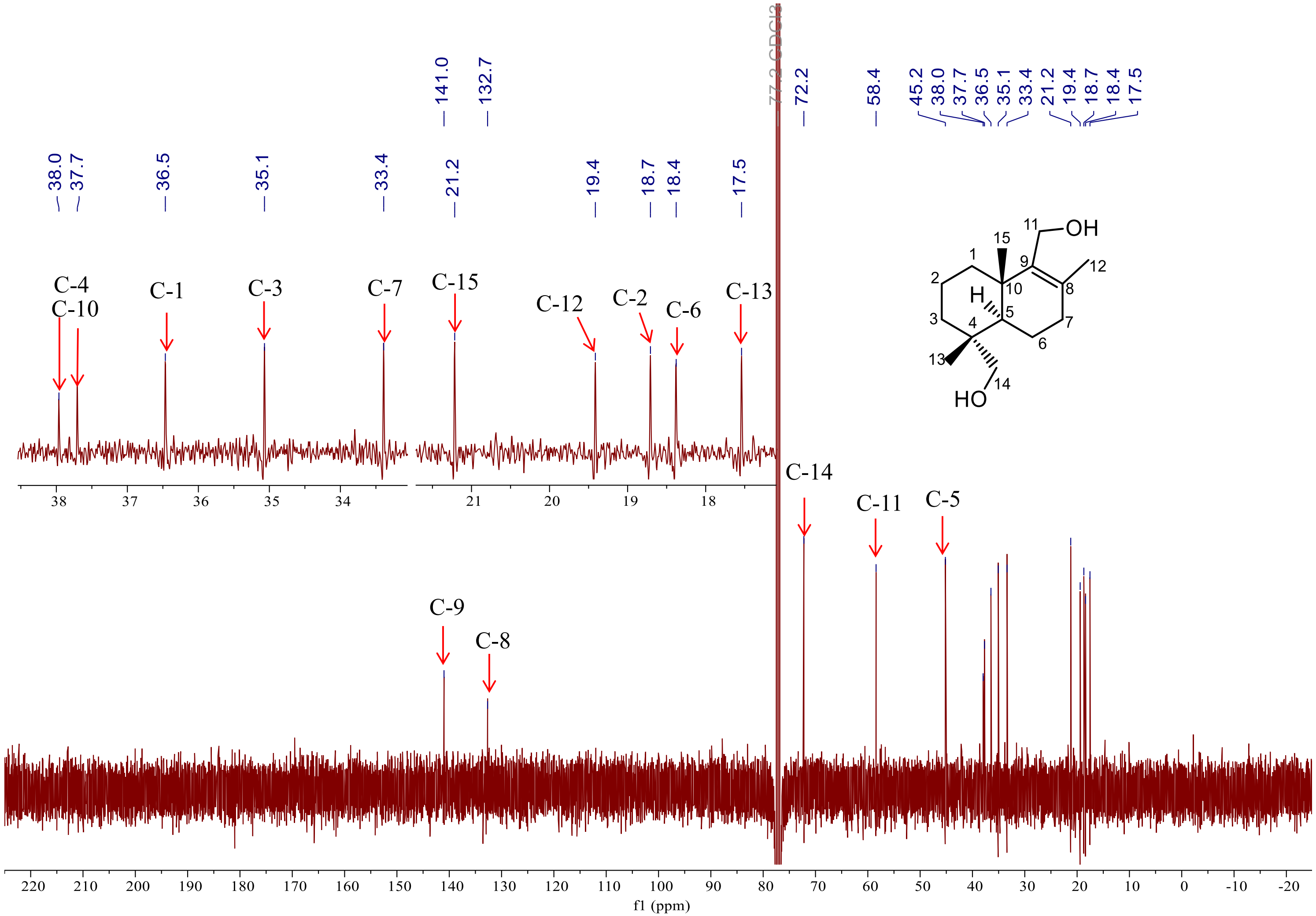

Figure S105. The ^{13}C NMR spectrum of ustidrimane N (**20**) in $CDCl_3$ (100 MHz).

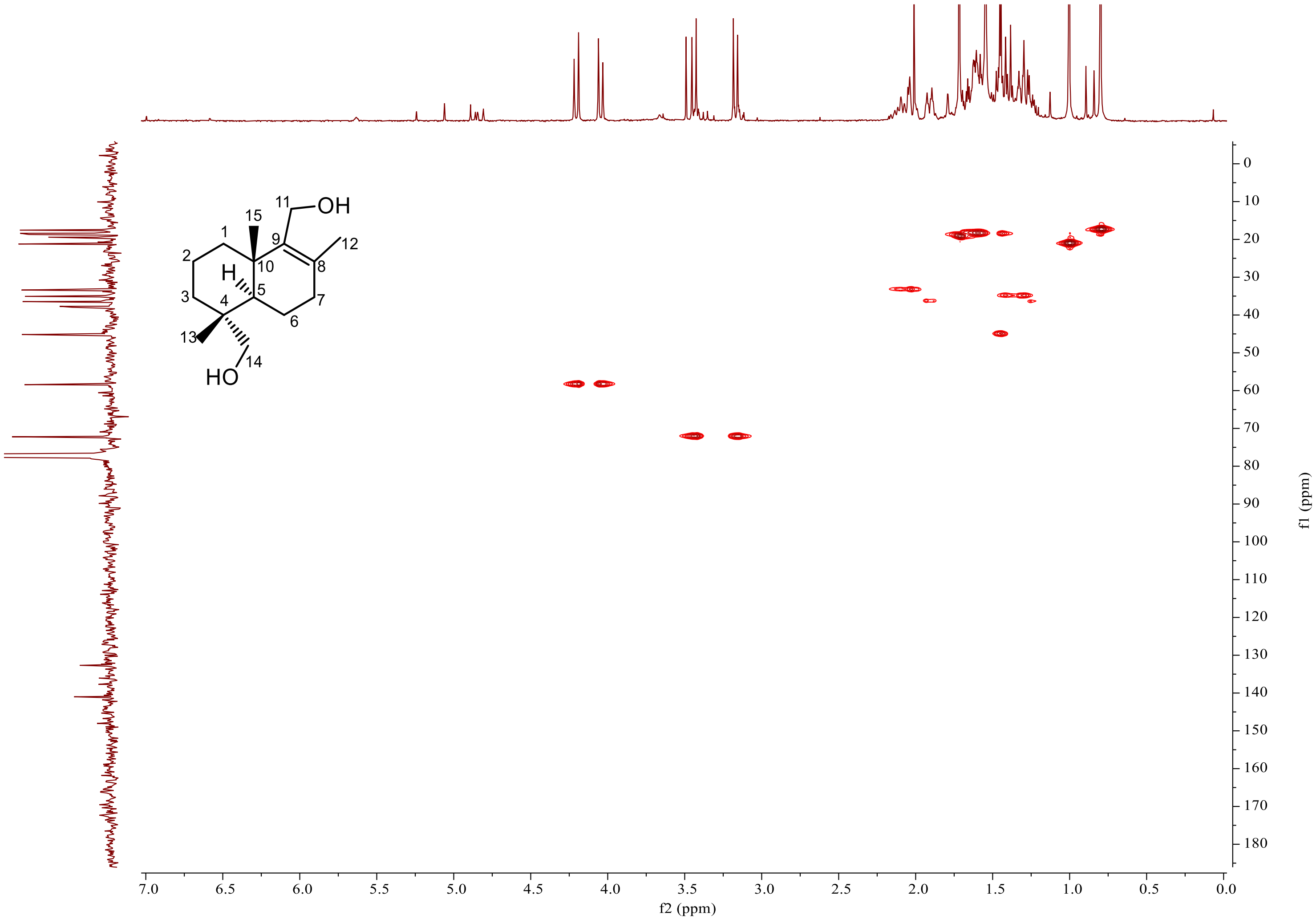

Figure S106. The HSQC spectrum of ustidrimane N (**20**) in $CDCl_3$.

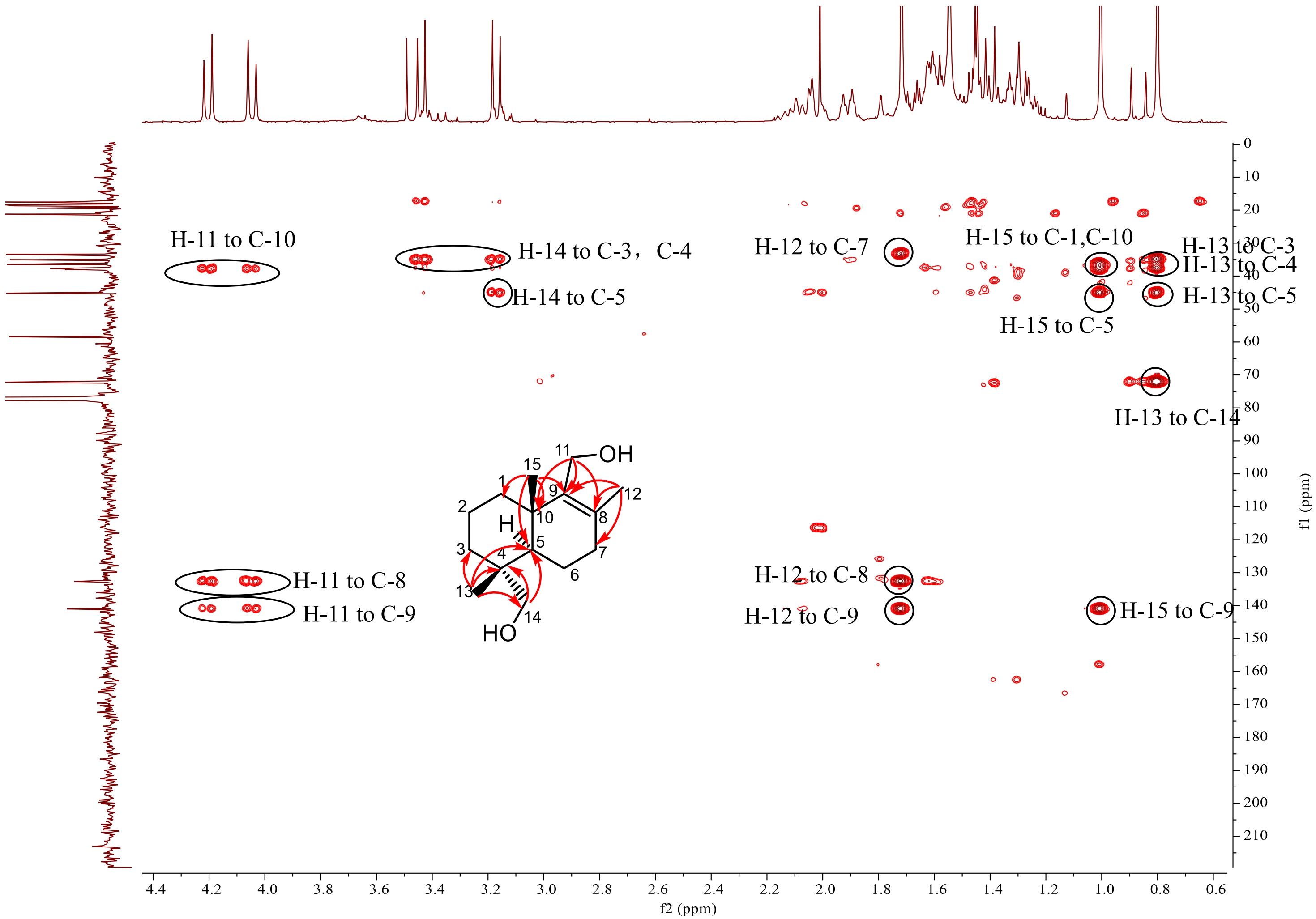

Figure S107. The HMBC spectrum of ustidrimane N (**20**) in $CDCl_3$.

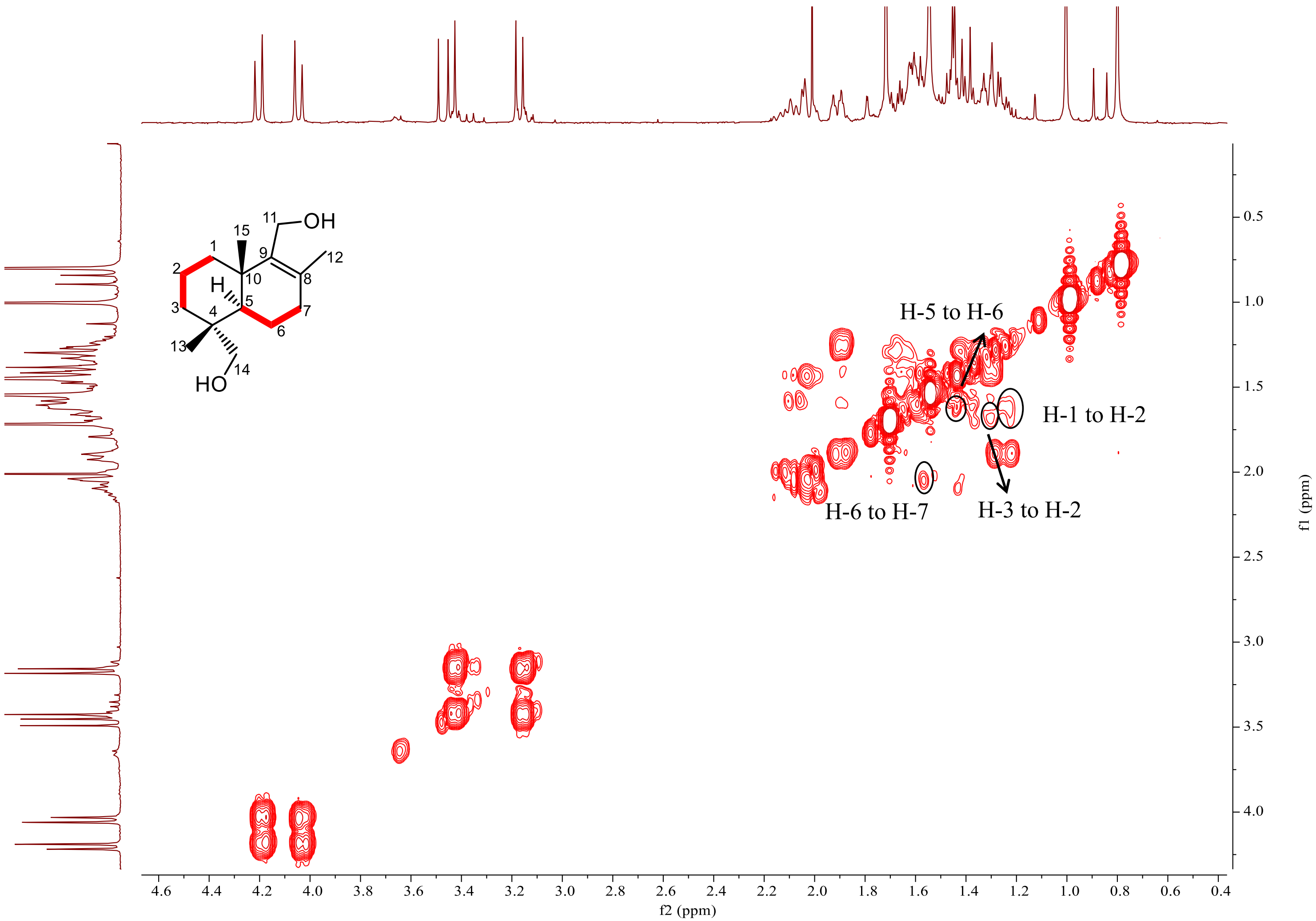

Figure S108. The ^{1}H-^{1}H COSY spectrum of ustidrimane N (**20**) in $CDCl_3$.

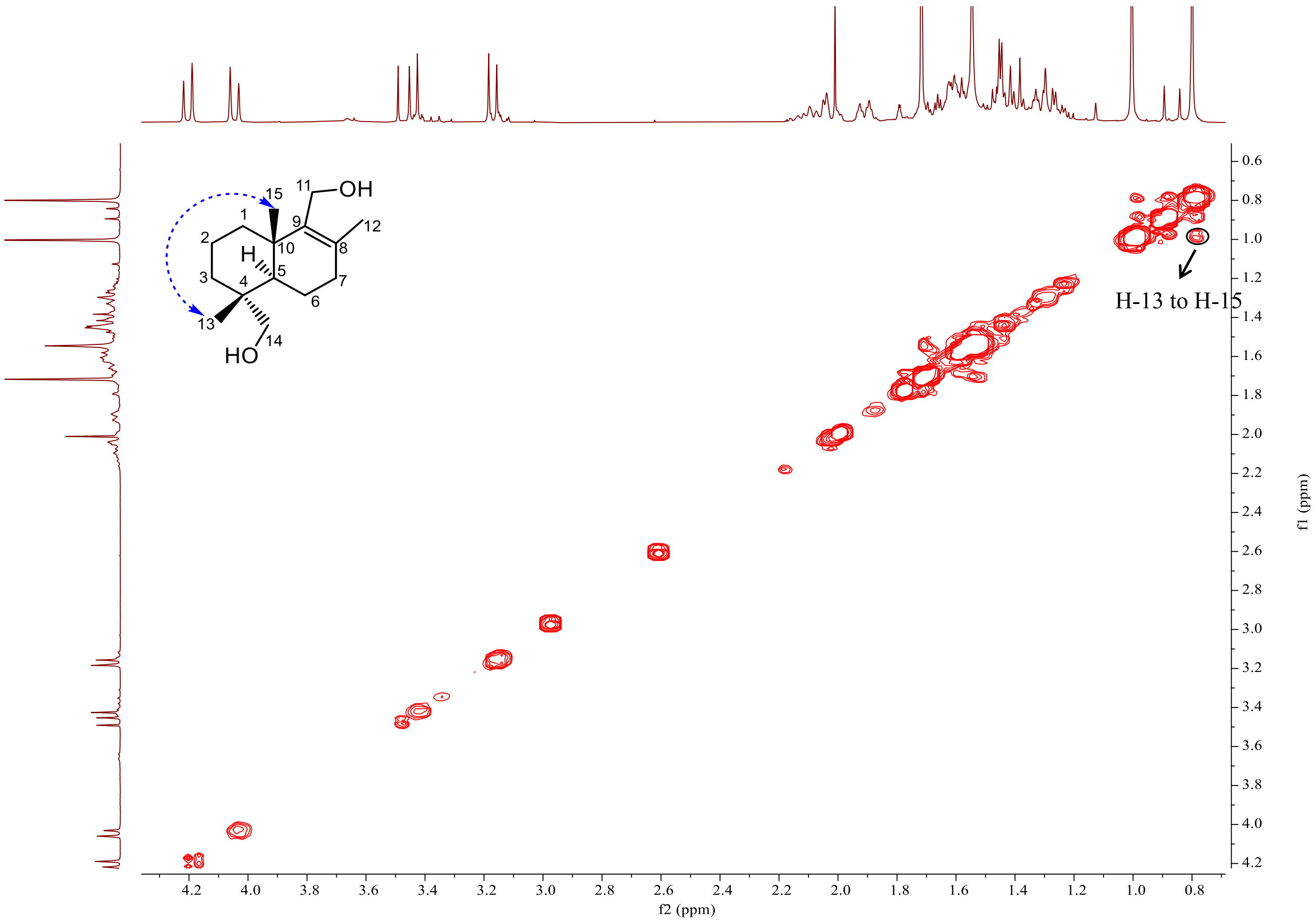

Figure S109. The NOESY spectrum of ustidrimane N (**20**) in $CDCl_3$.

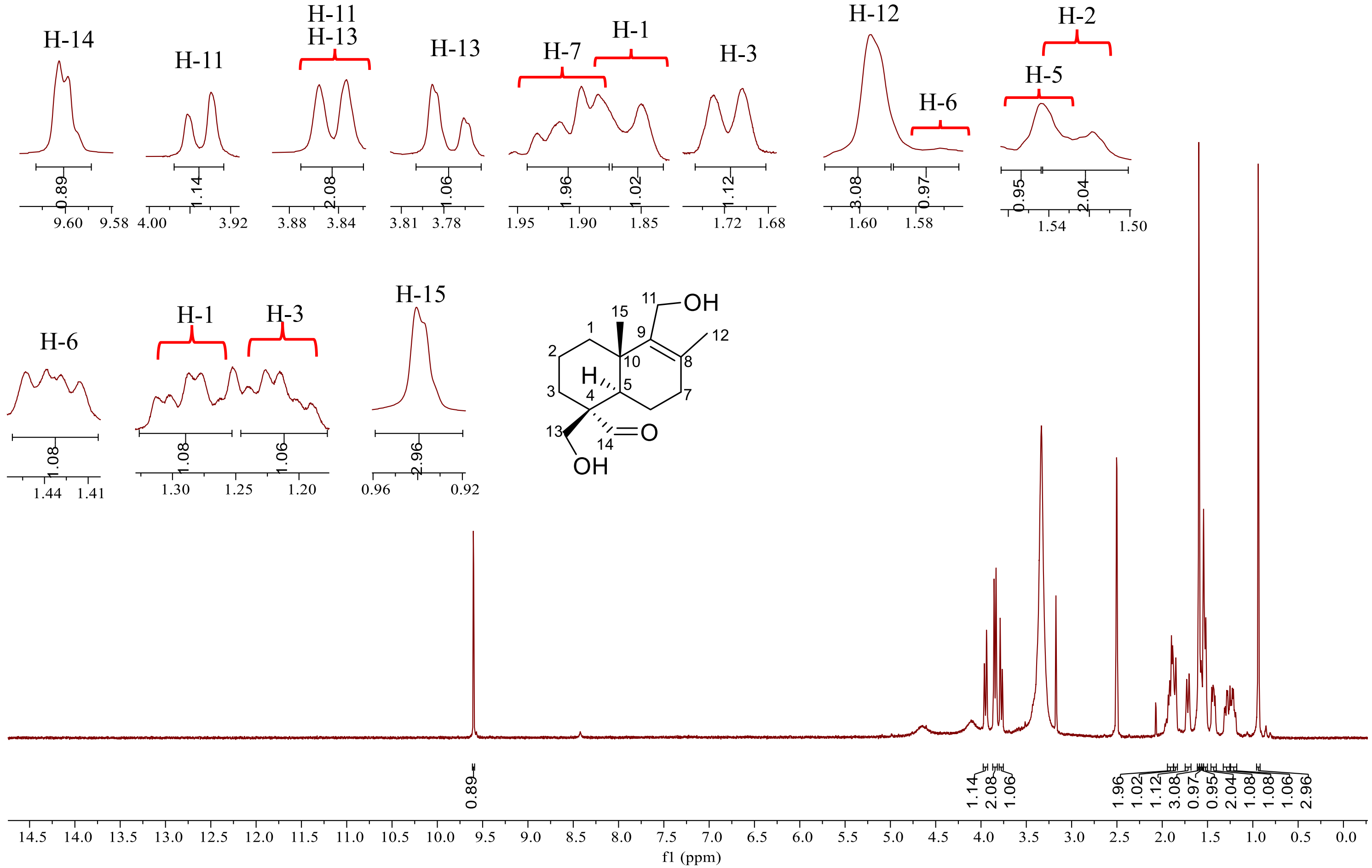

Figure S110. The ^{1}H NMR spectrum of ustidrimane O (**21**) in DMSO-d_6 (400 MHz).

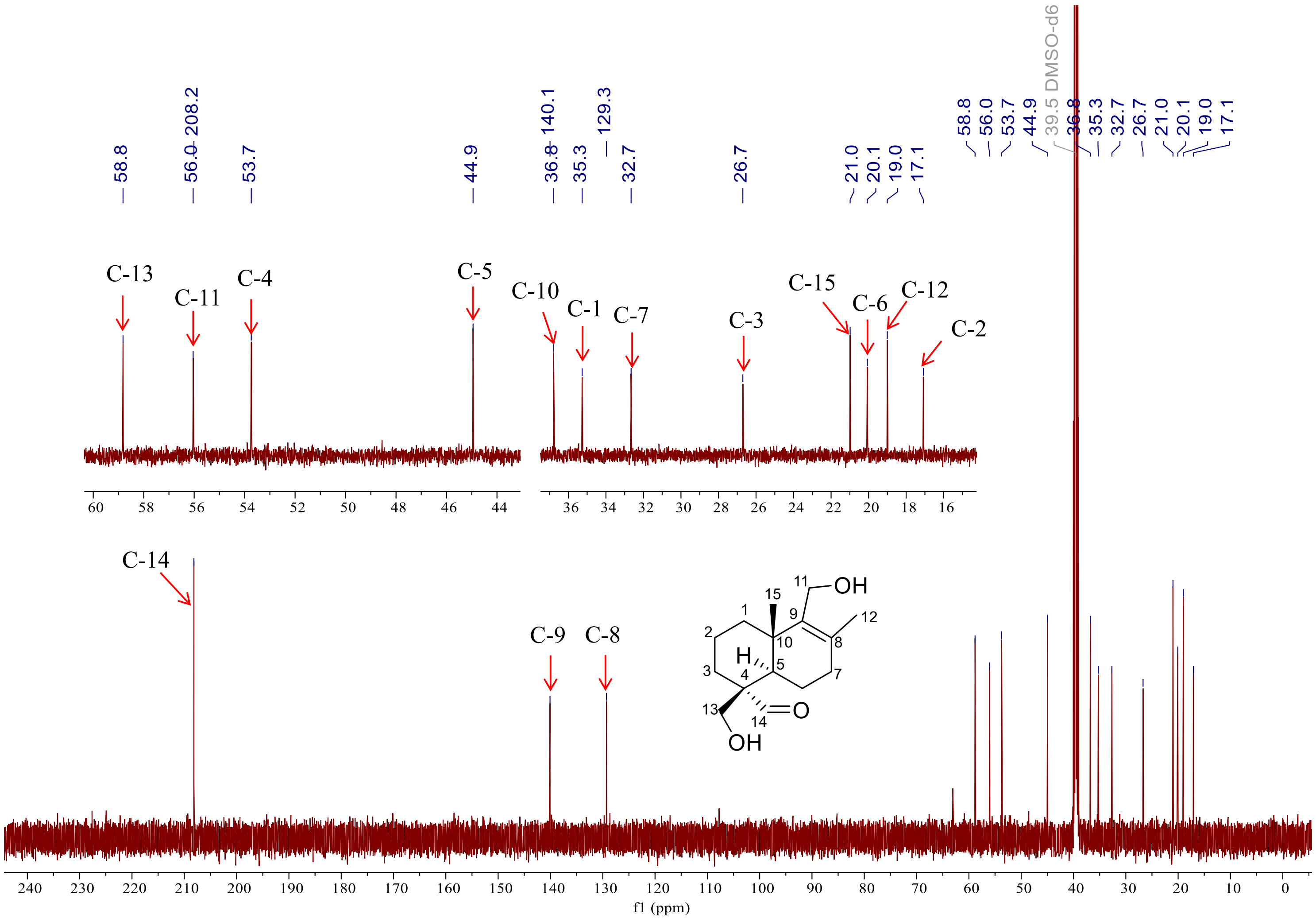

Figure S111. The ^{13}C NMR spectrum of ustidrimane O (**21**) in DMSO-d_6 (100 MHz).

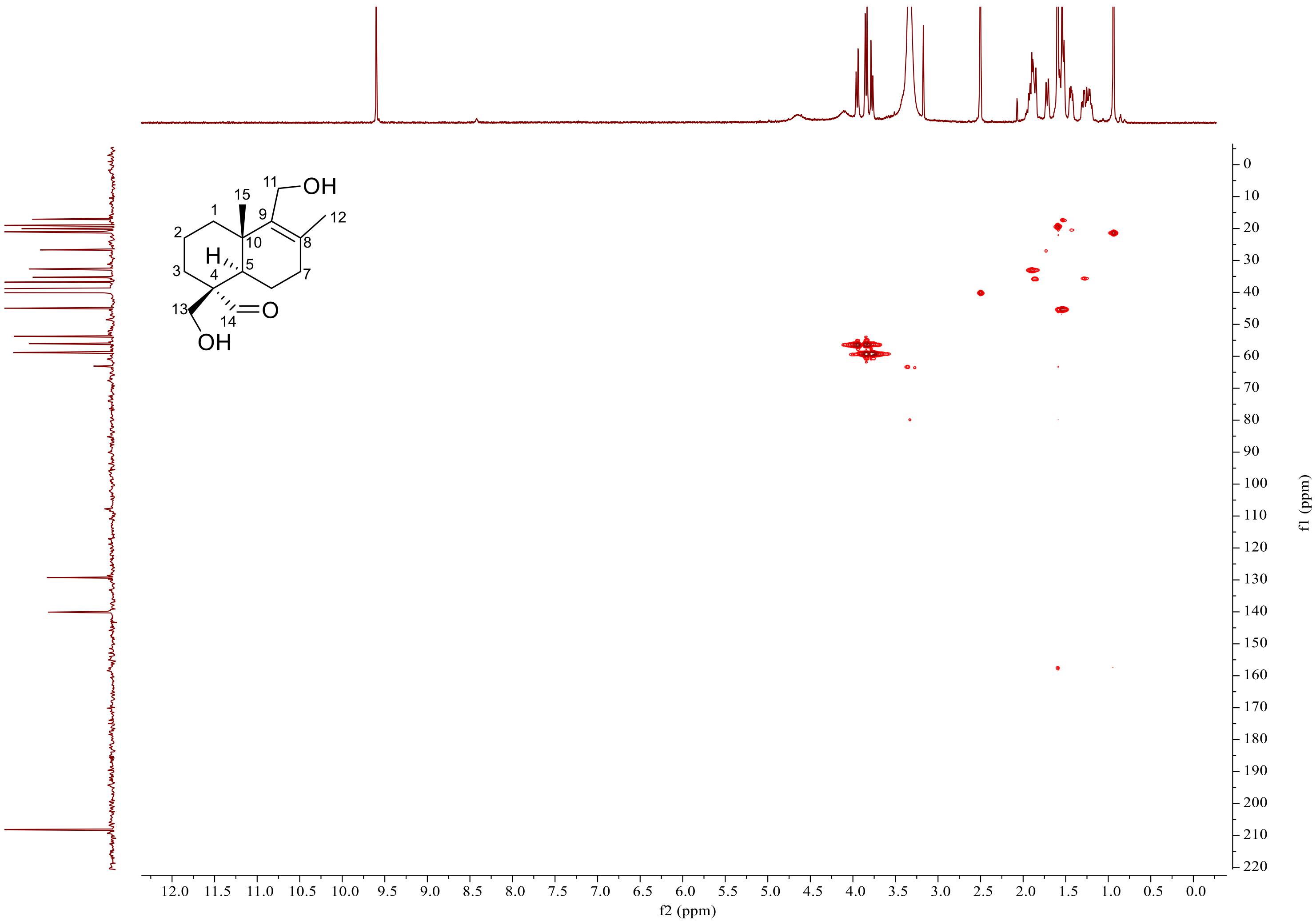

Figure S112. The HSQC spectrum of ustidrimane O (**21**) in DMSO-d_6.

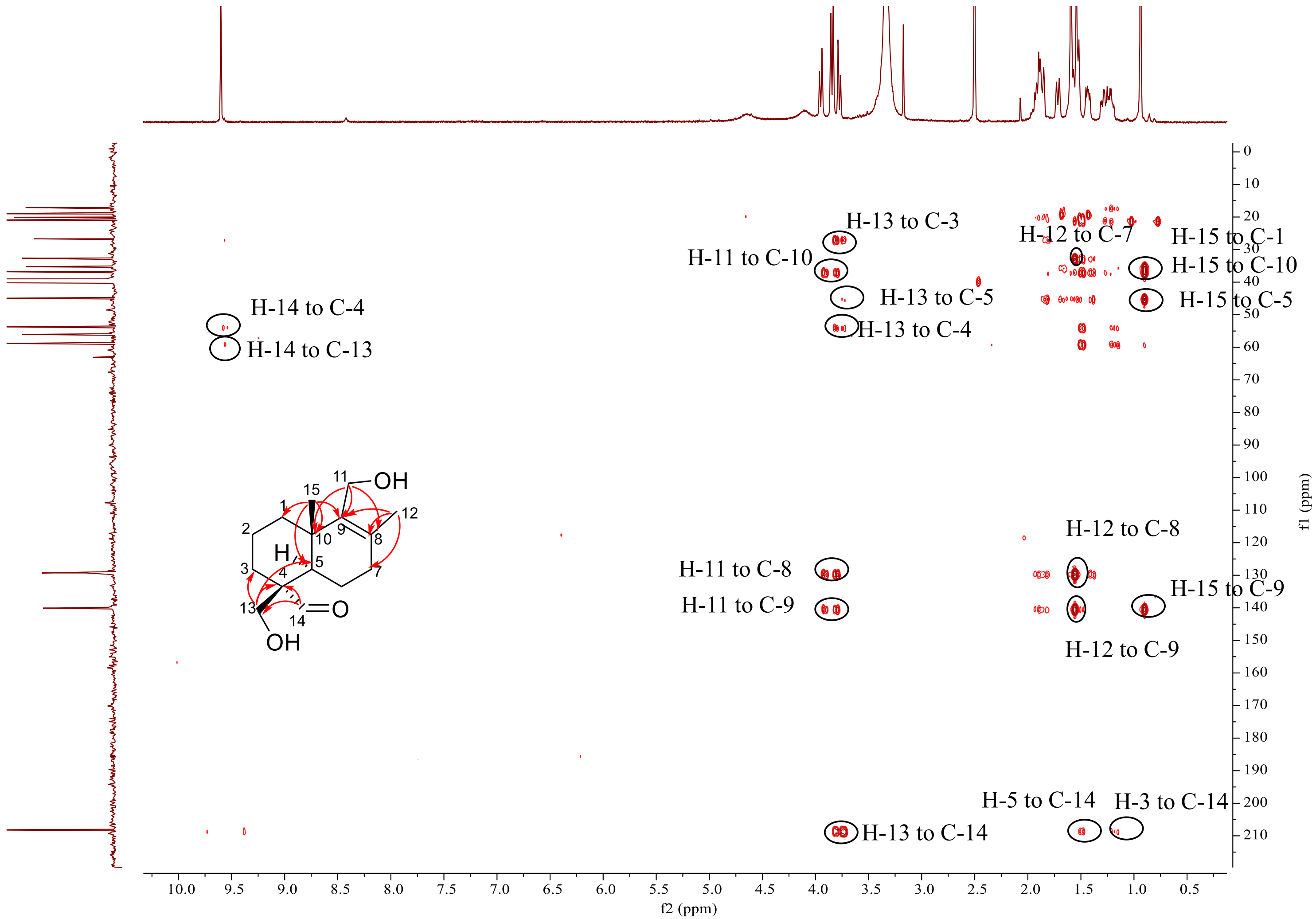

Figure S113. The HMBC spectrum of ustidrimane O (**21**) in DMSO-d_6.

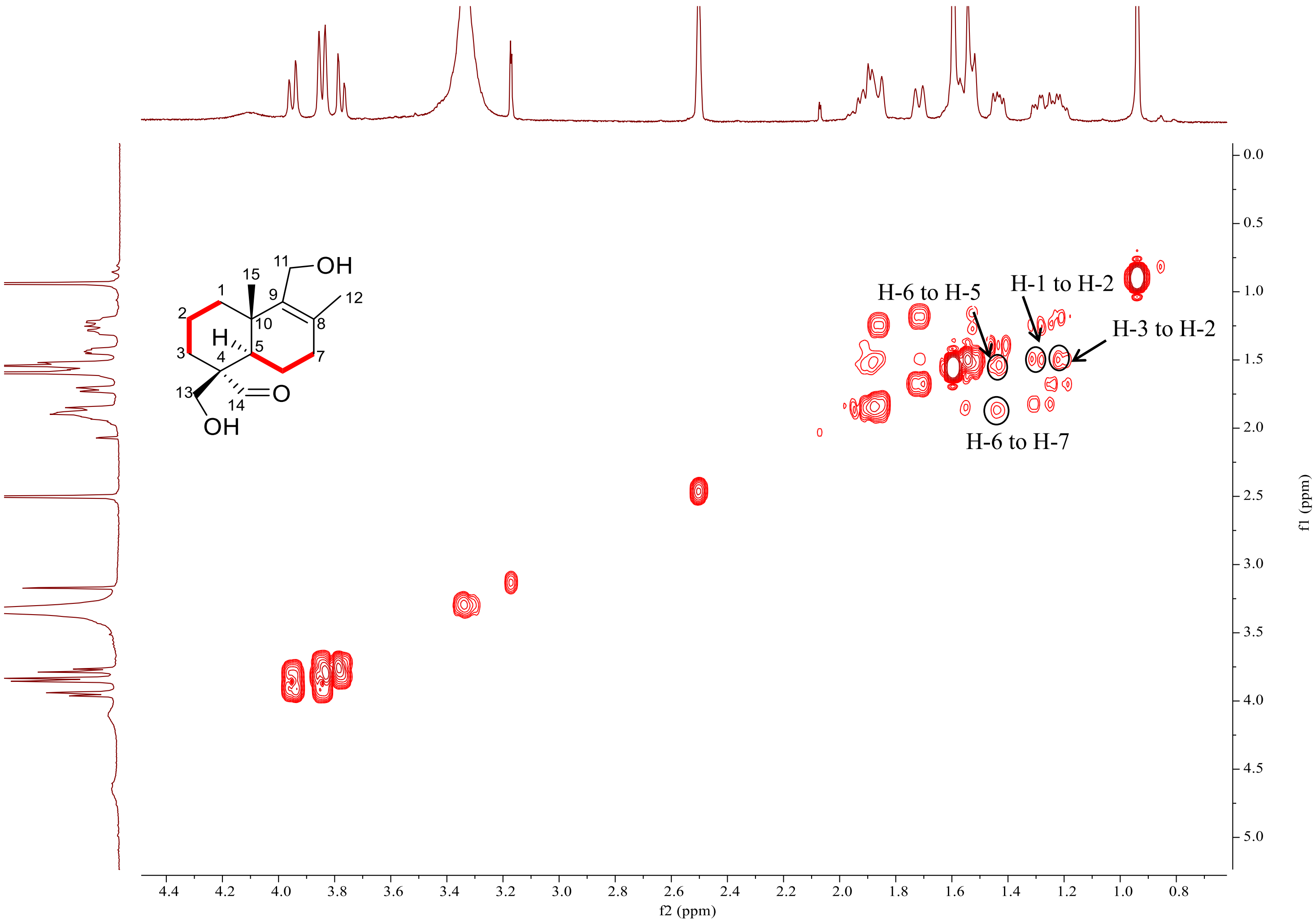

Figure S114. The ^{1}H-^{1}H COSY spectrum of ustidrimane O (**21**) in DMSO-d_6.

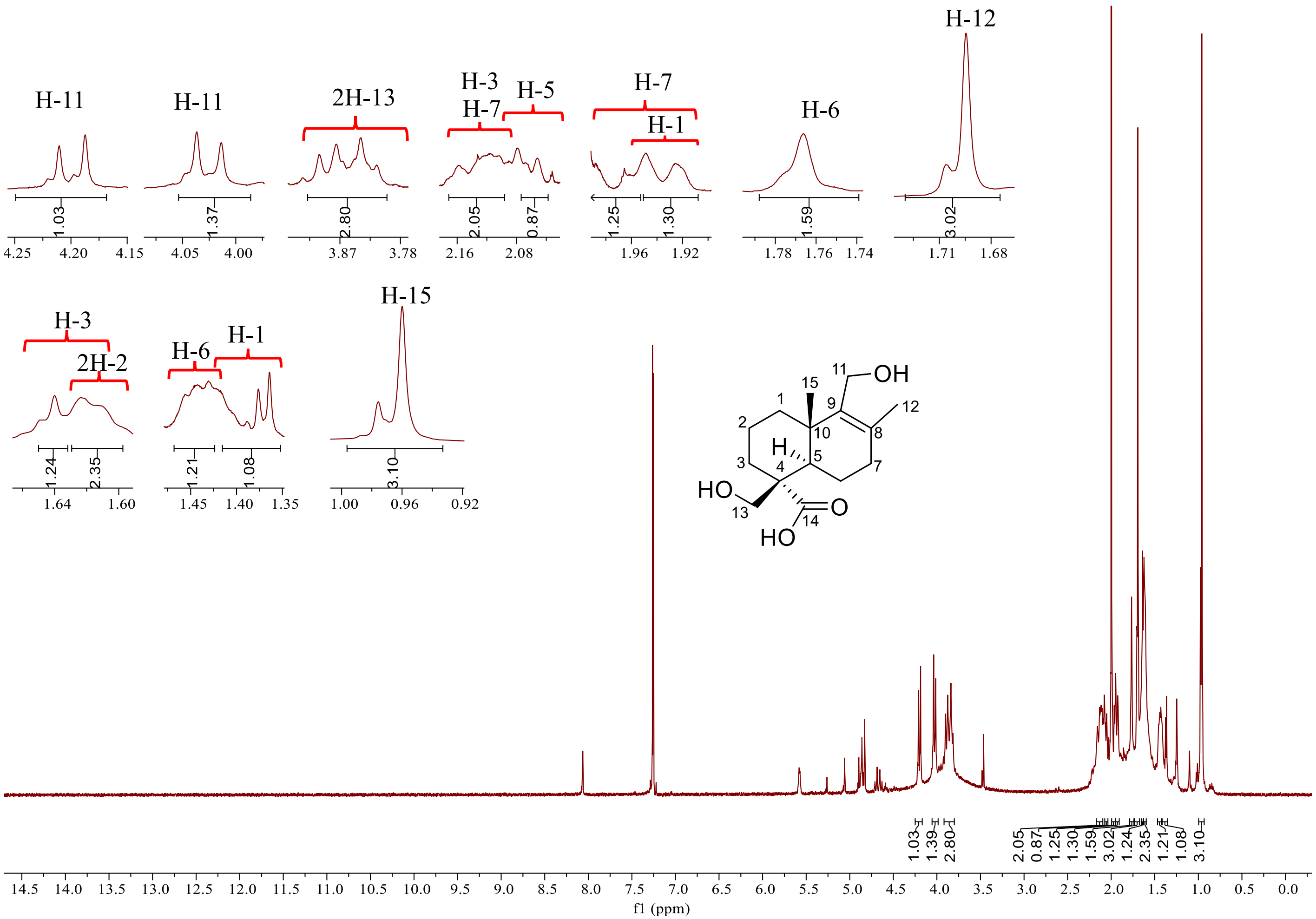

Figure S115. The ^{1}H NMR spectrum of ustidrimane P (**22**) in $CDCl_3$ (400 MHz).

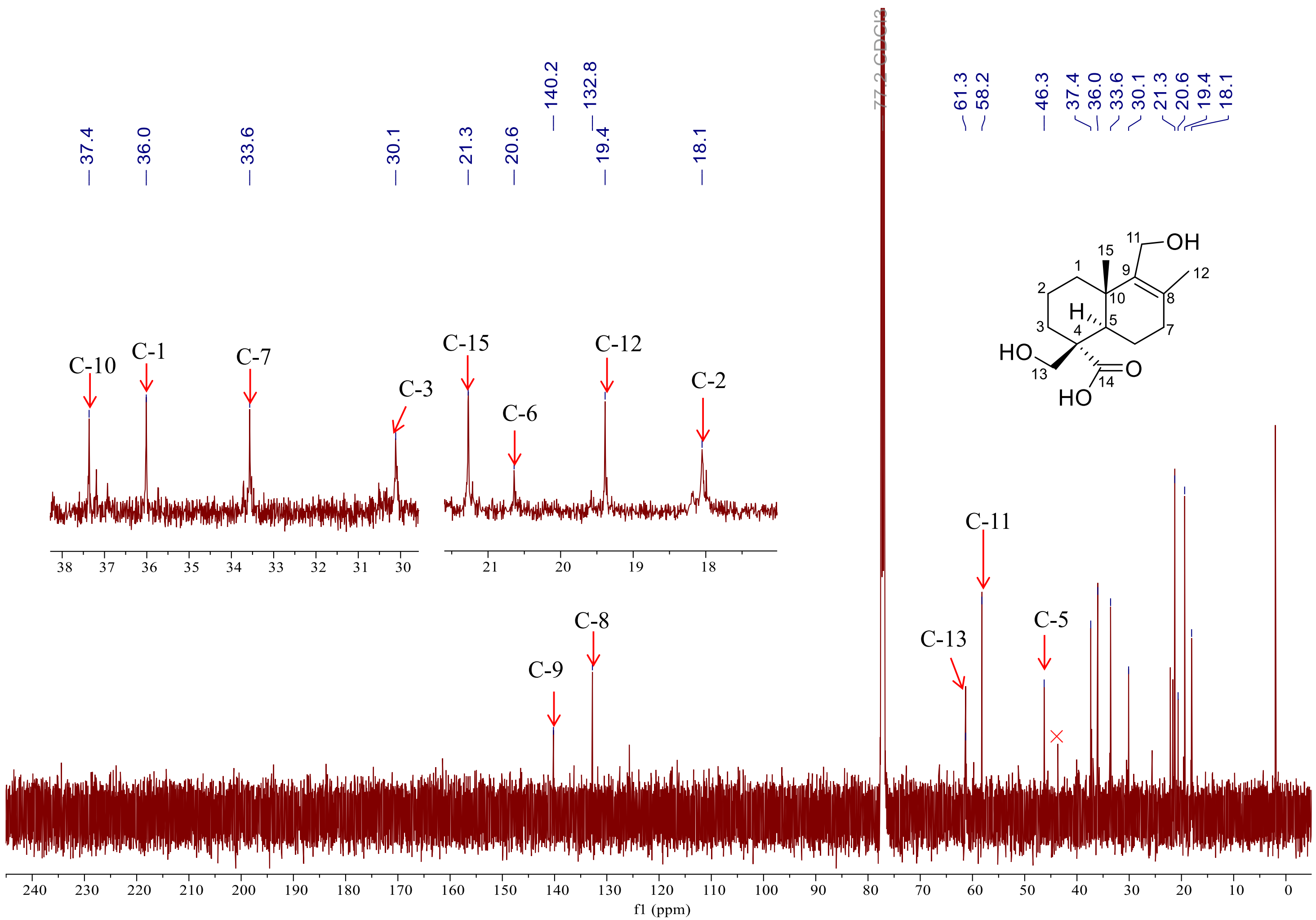

Figure S116. The ^{13}C NMR spectrum of ustidrimane P (**22**) in $CDCl_3$ (100 MHz).

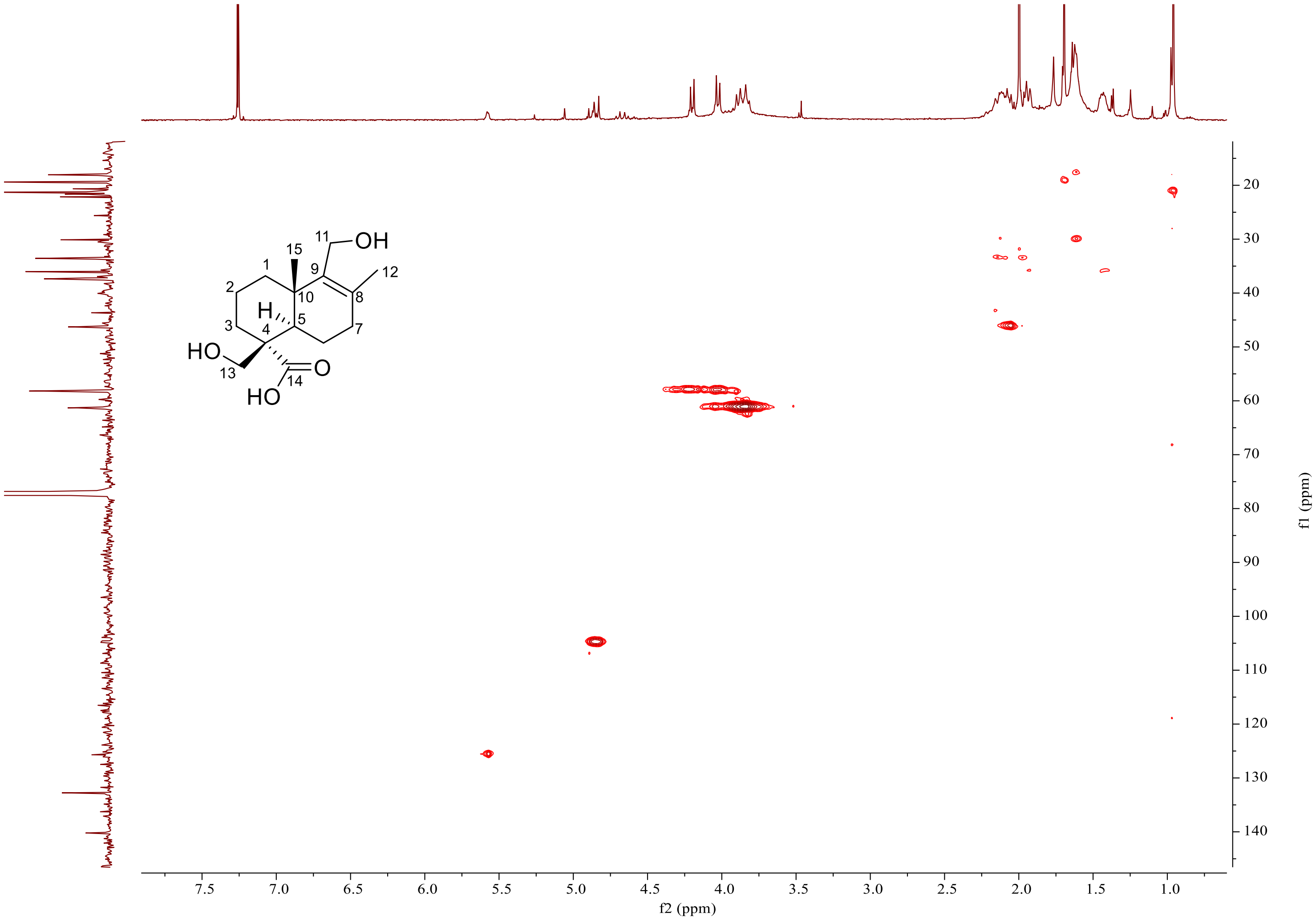

Figure S117. The HSQC spectrum of ustidrimane P (**22**) in $CDCl_3$.

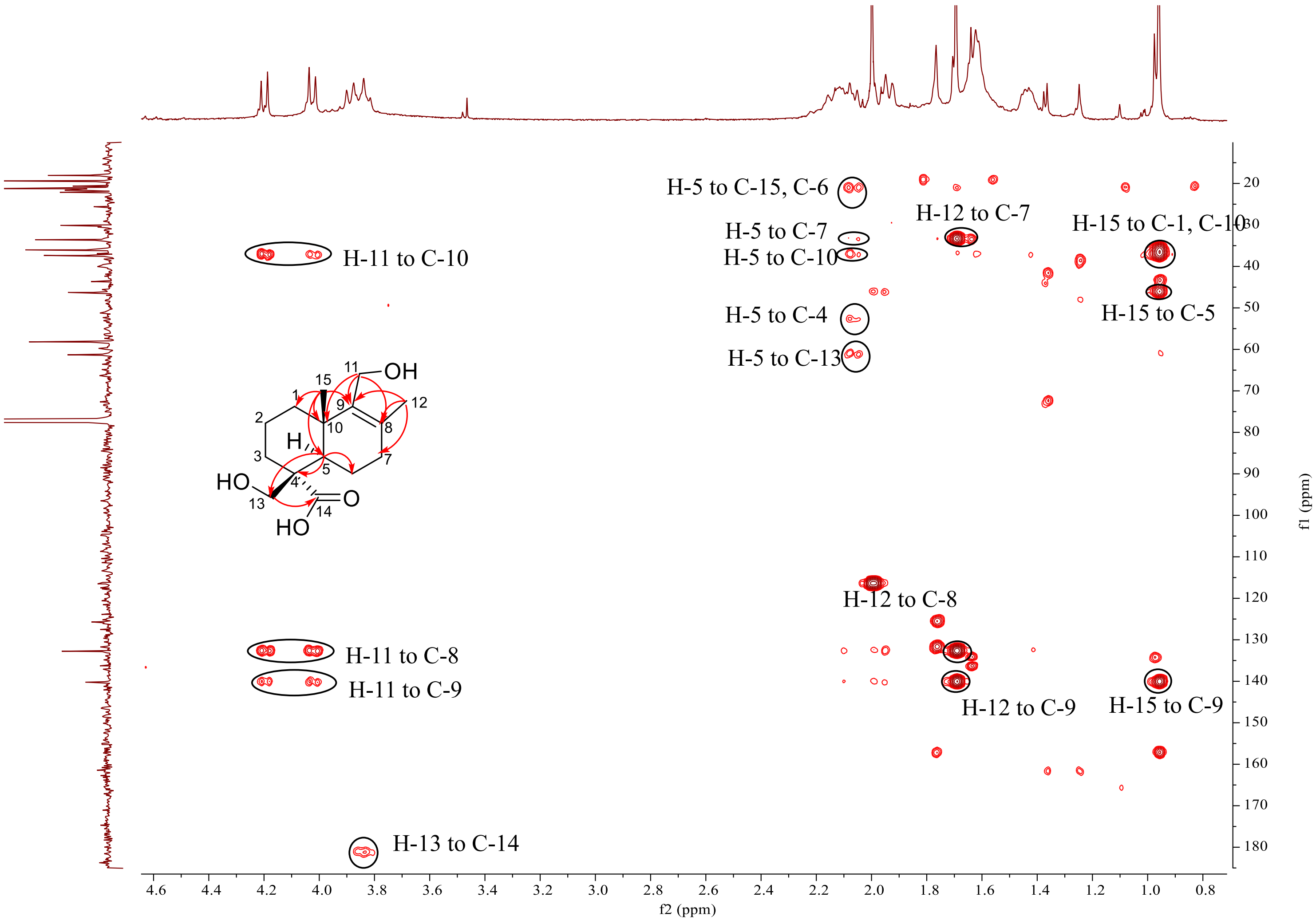

Figure S118. The HMBC spectrum of ustidrimane P (**22**) in $CDCl_3$.

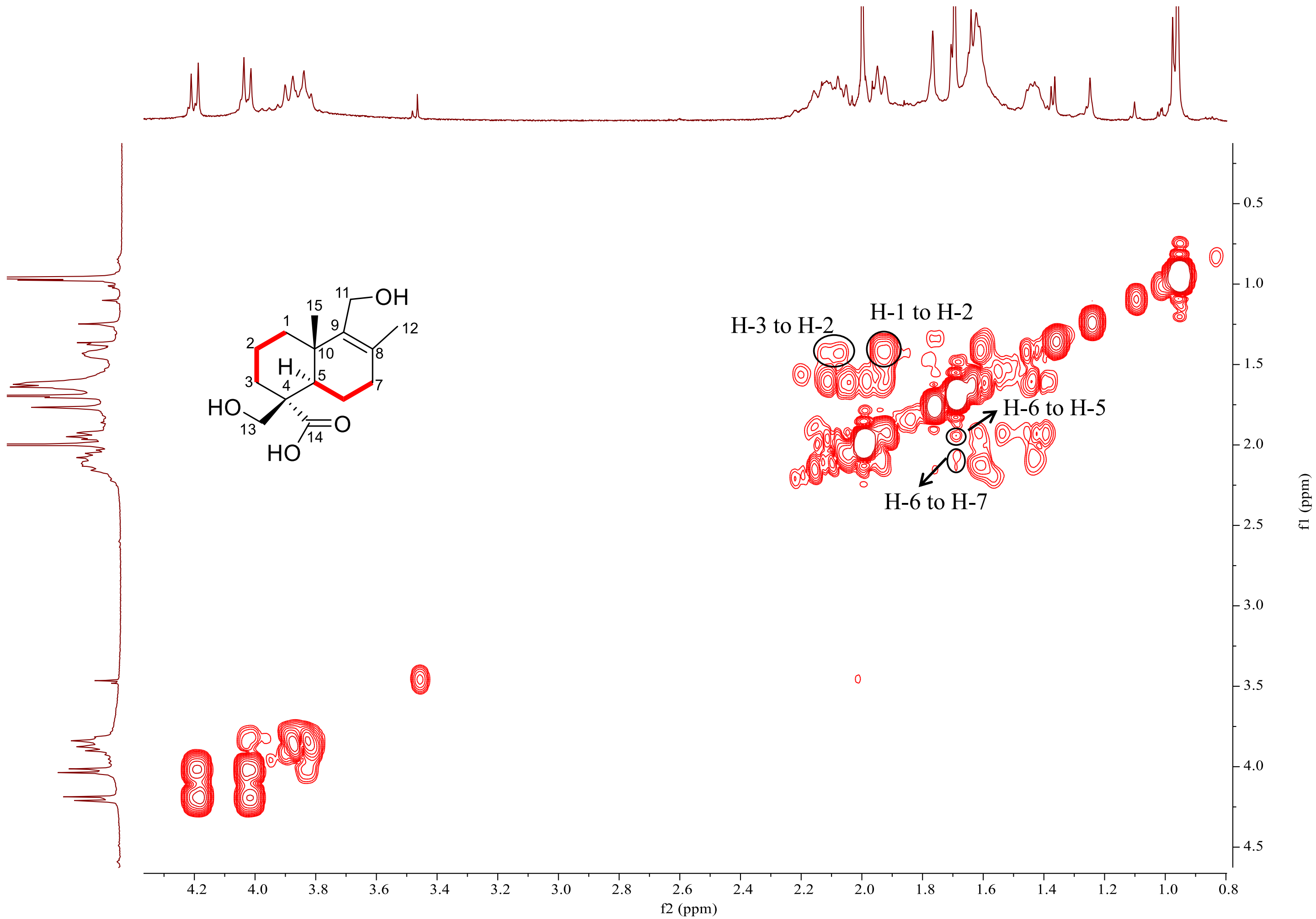

Figure S119. The ^{1}H-^{1}H COSY spectrum of ustidrimane P (**22**) in $CDCl_3$.

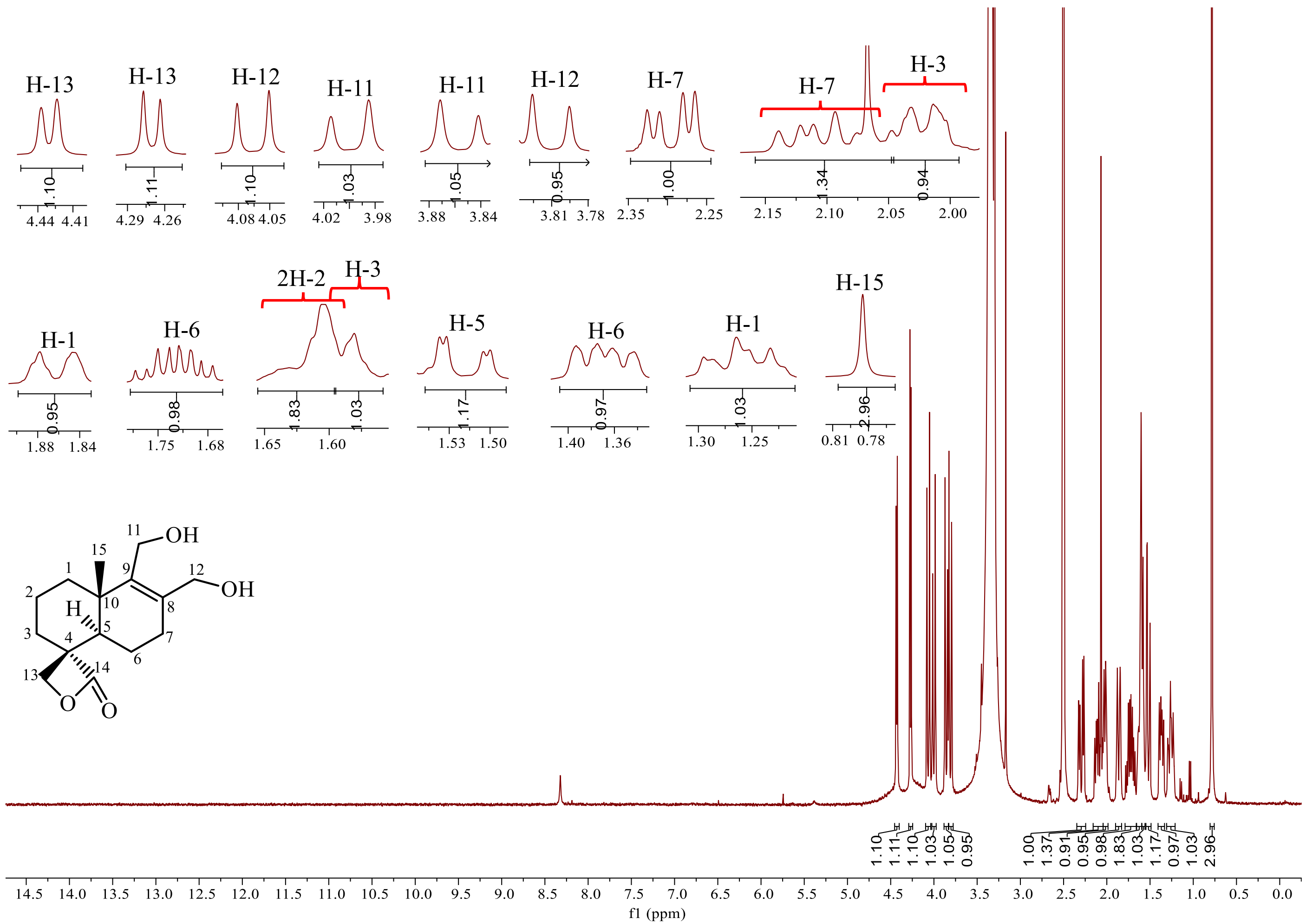

Figure S120. The ^{1}H NMR spectrum of ustidrimane Q (**23**) in DMSO-d_6 (400 MHz).

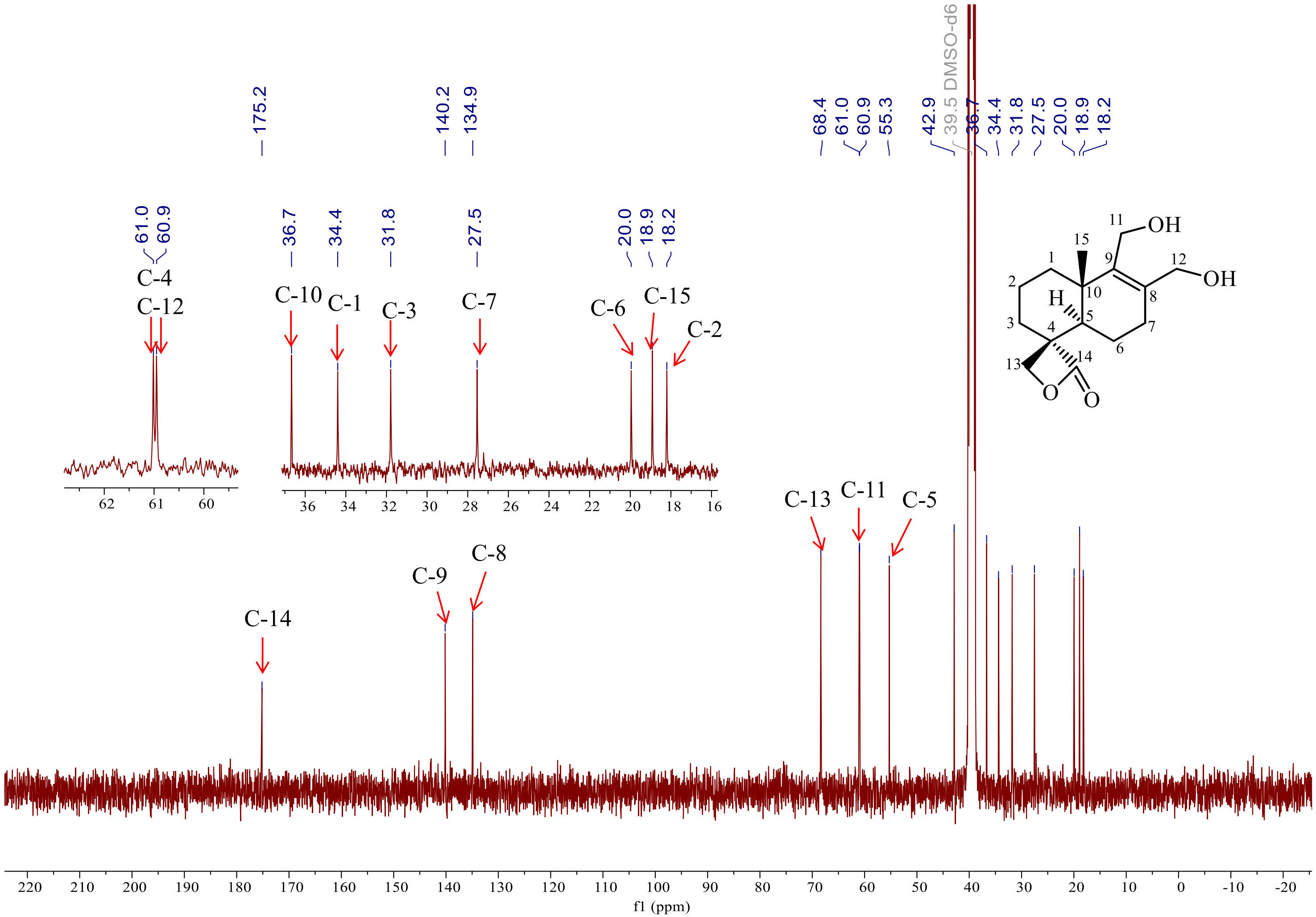

Figure S121. The ^{13}C NMR spectrum of ustidrimane Q (**23**) in DMSO-d_6 (100 MHz).

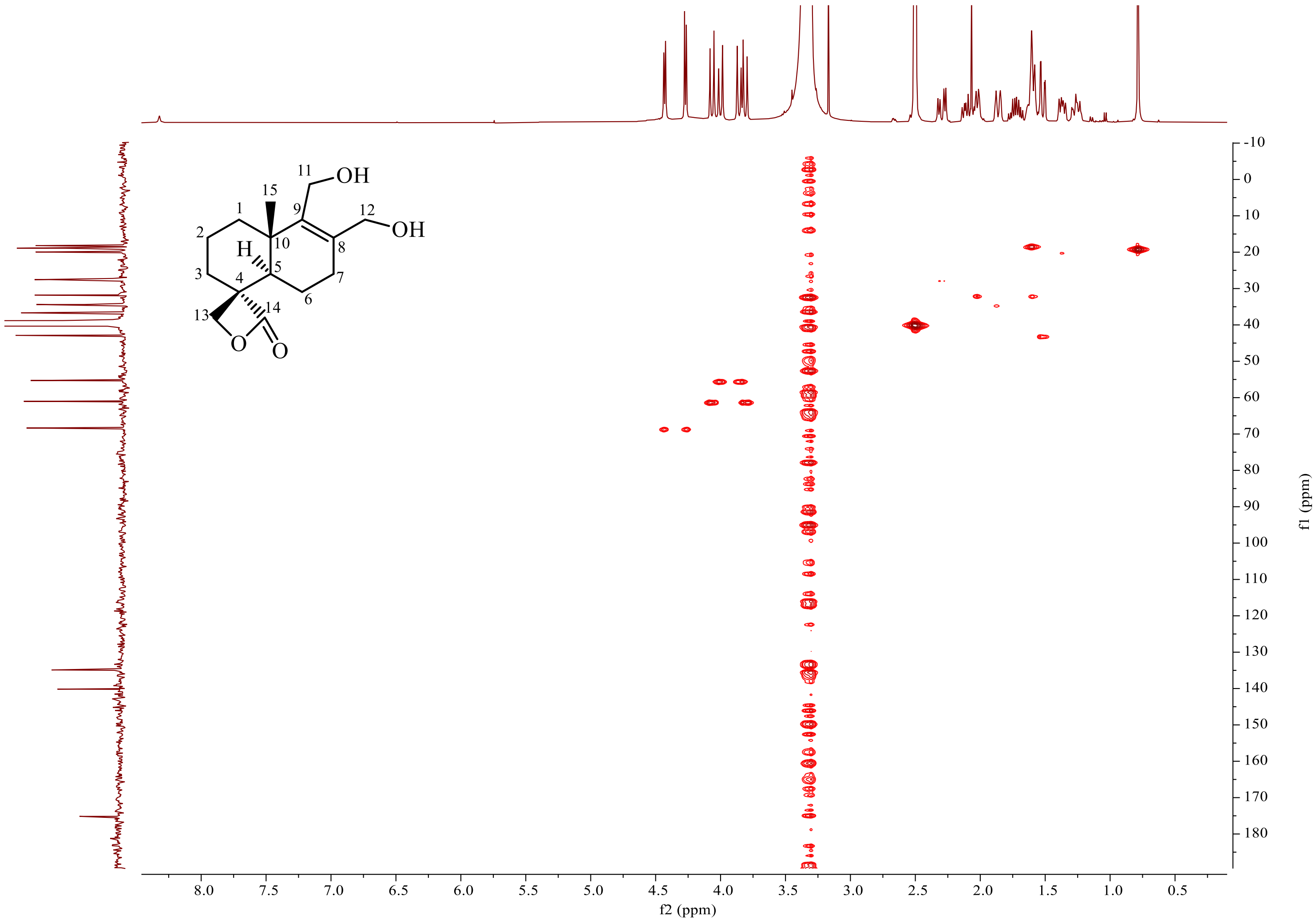

Figure S122. The HSQC spectrum of ustidrimane Q (**23**) in DMSO-d_6.

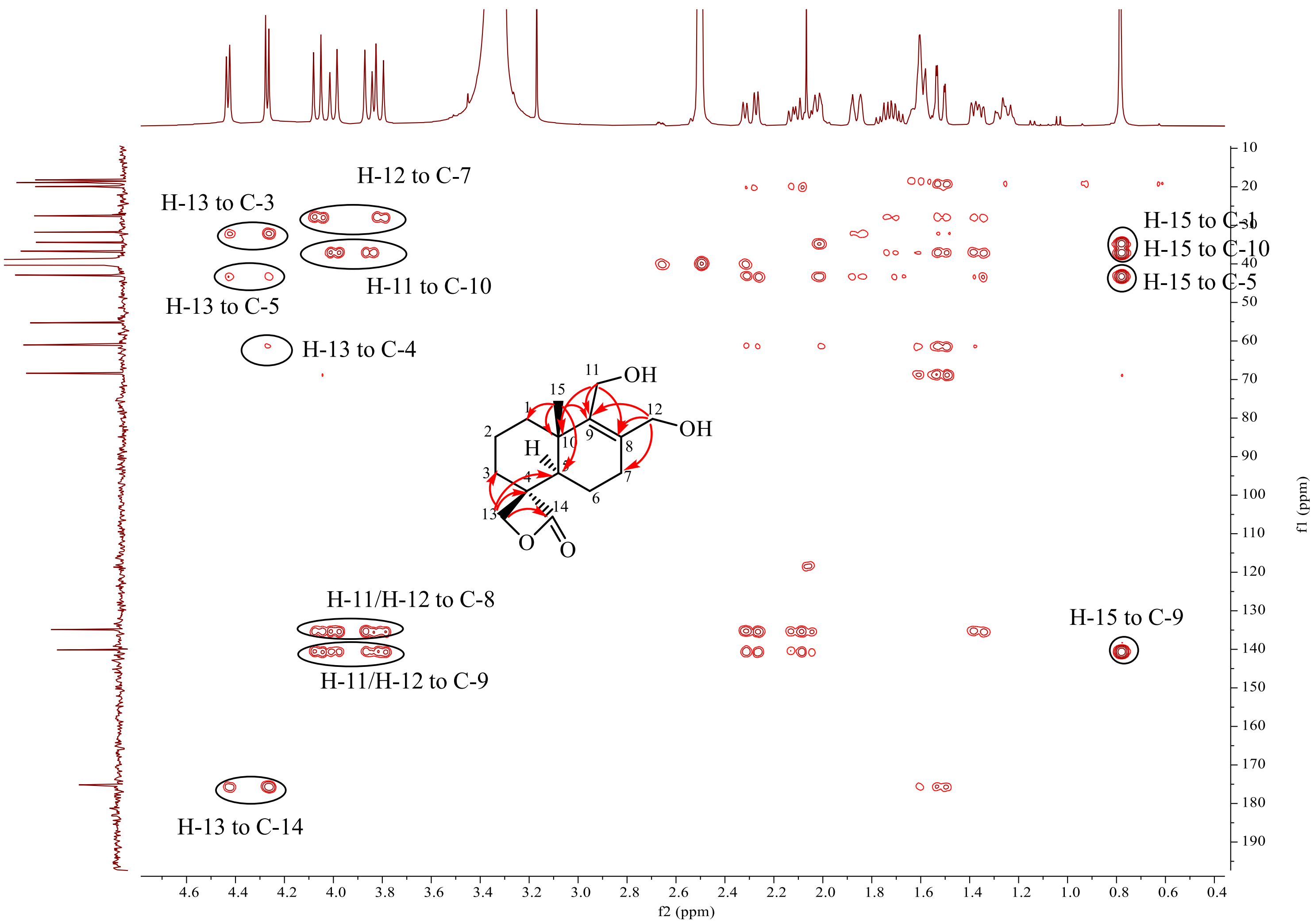

Figure S123. The HMBC spectrum of ustidrimane Q (**23**) in DMSO-d_6.

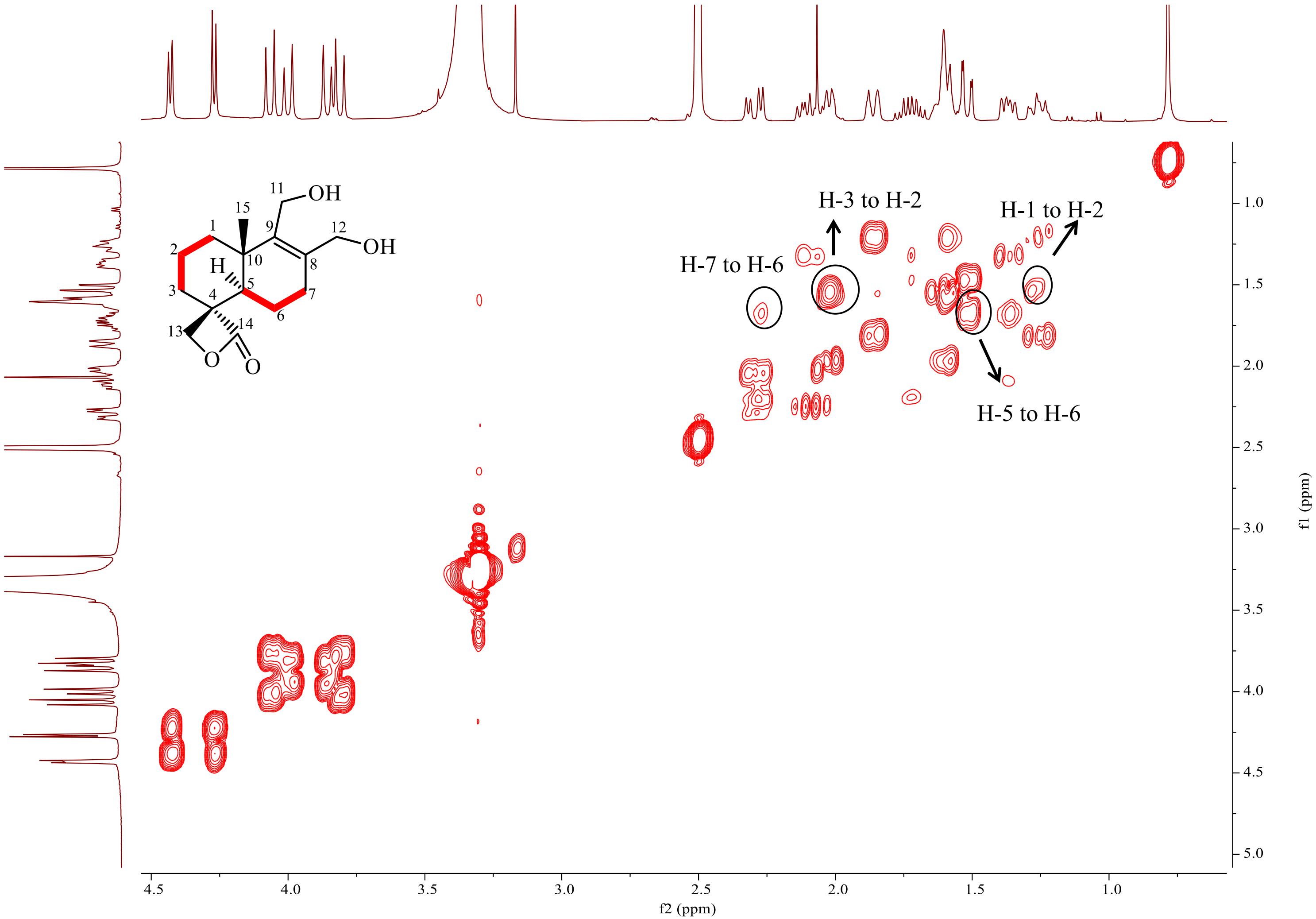

Figure S124. The ^{1}H-^{1}H COSY spectrum of ustidrimane Q (**23**) in DMSO-d_6.

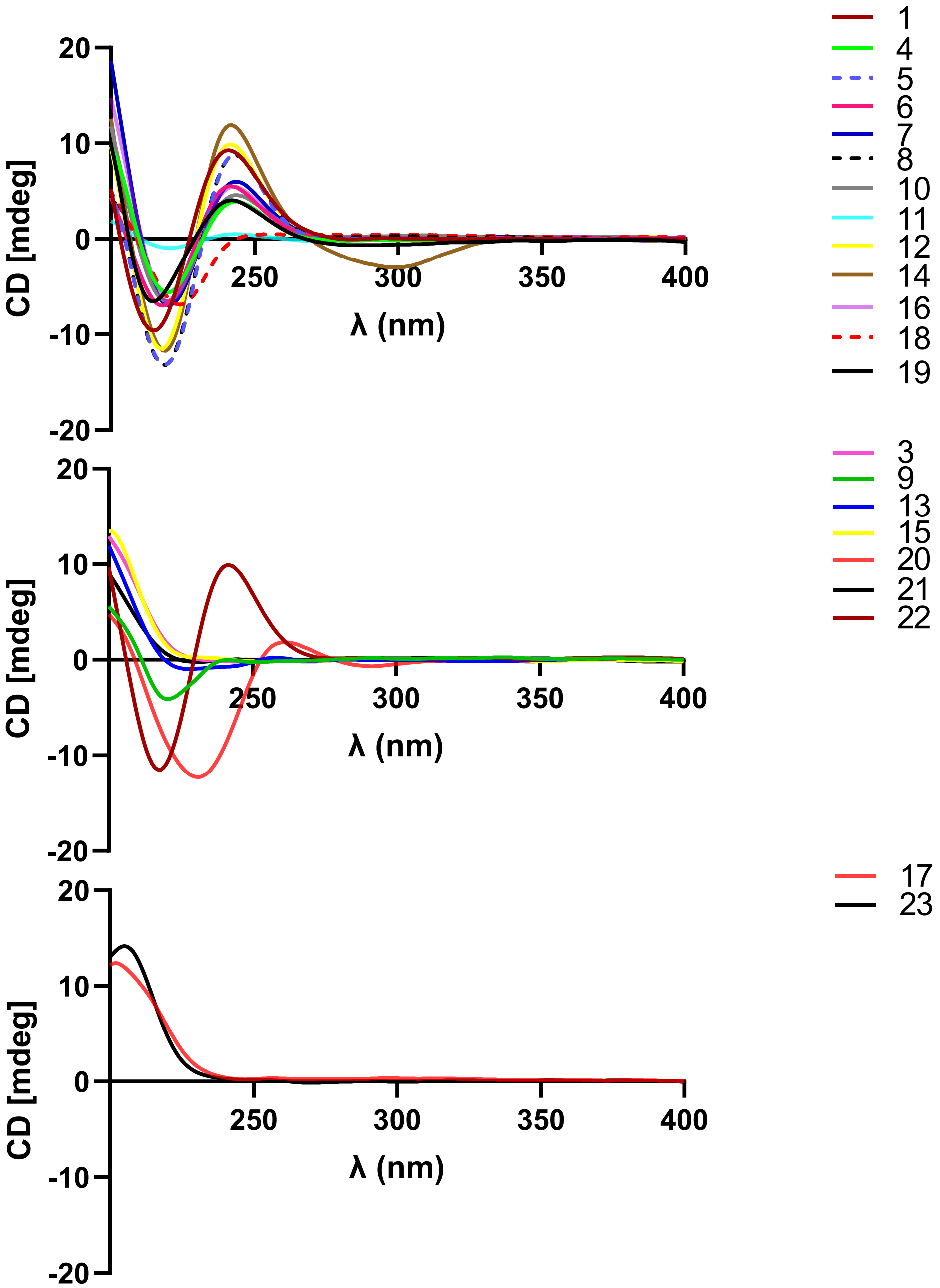

Figure S125. The ECD spectra of the isolated compounds **1** and **3–23** in MeOH.

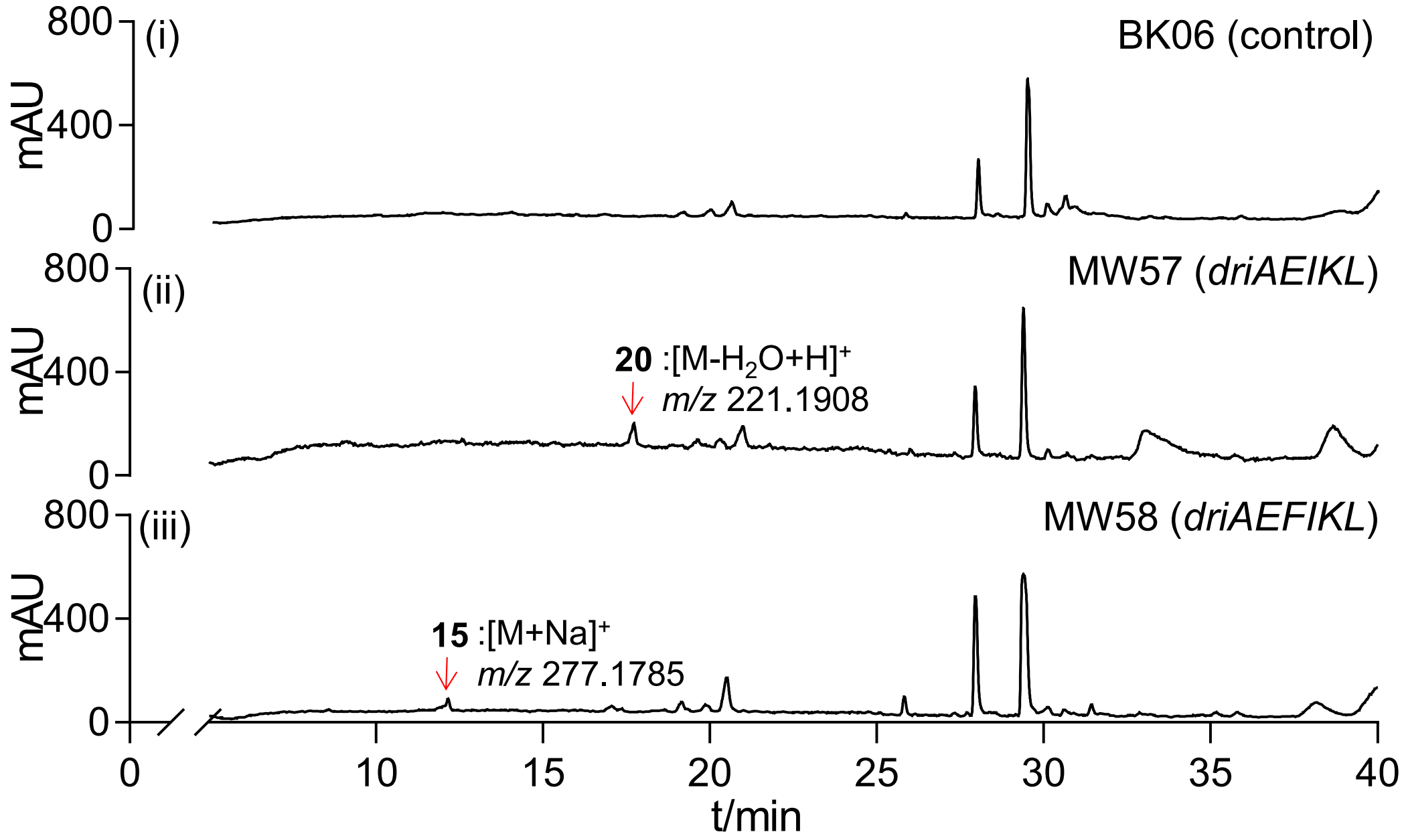

Figure S126. LC-MS chromatograms of the metabolites from *A. nidulans* MW57 and MW58. Absorptions at UV 210 nm are displayed.

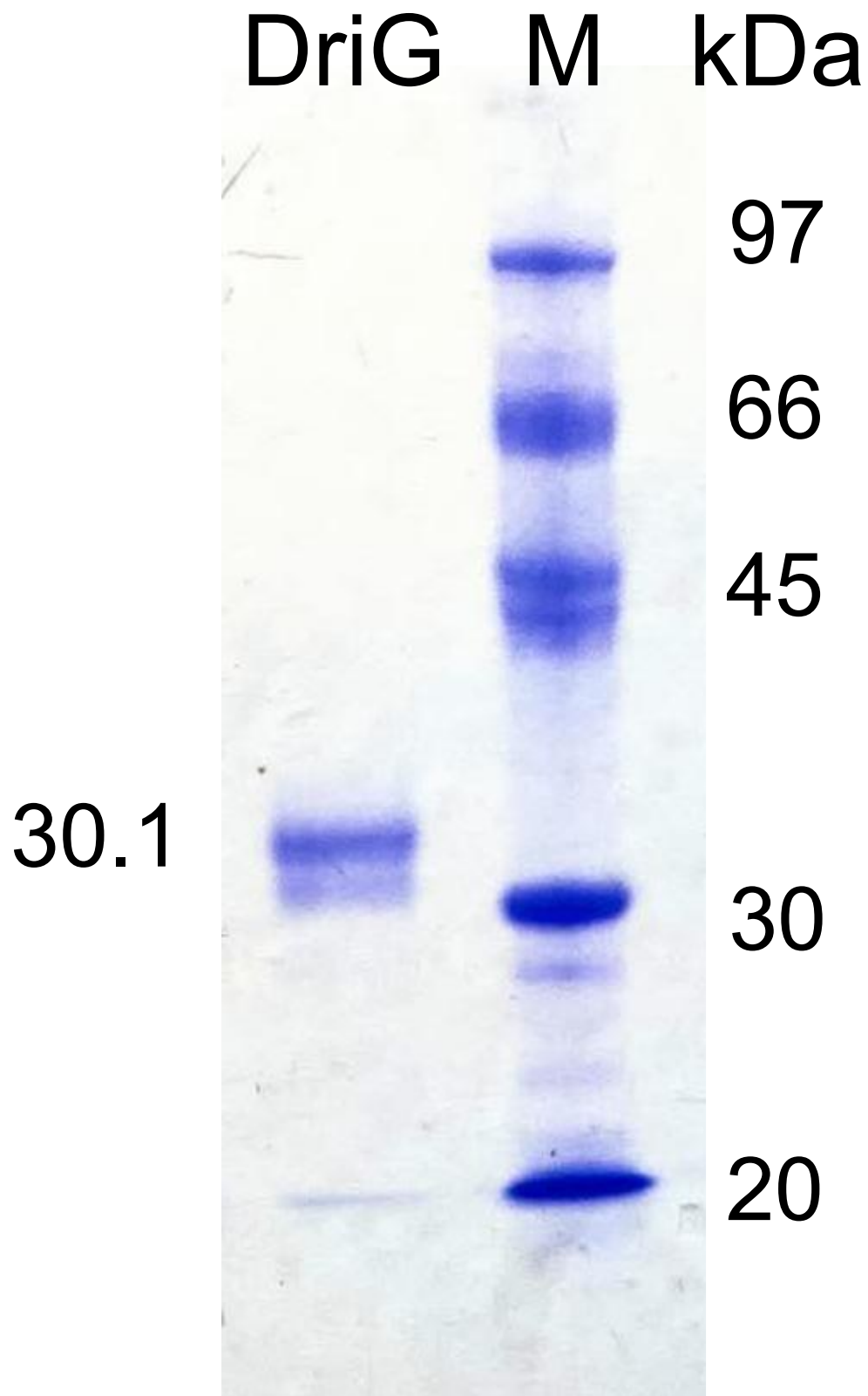

Figure S127. SDS-PAGE analysis of purified recombinant DriG. The overproduced and purified DriG was separated by SDS-PAGE (12%), stained with Coomassie brilliant blue R-250.

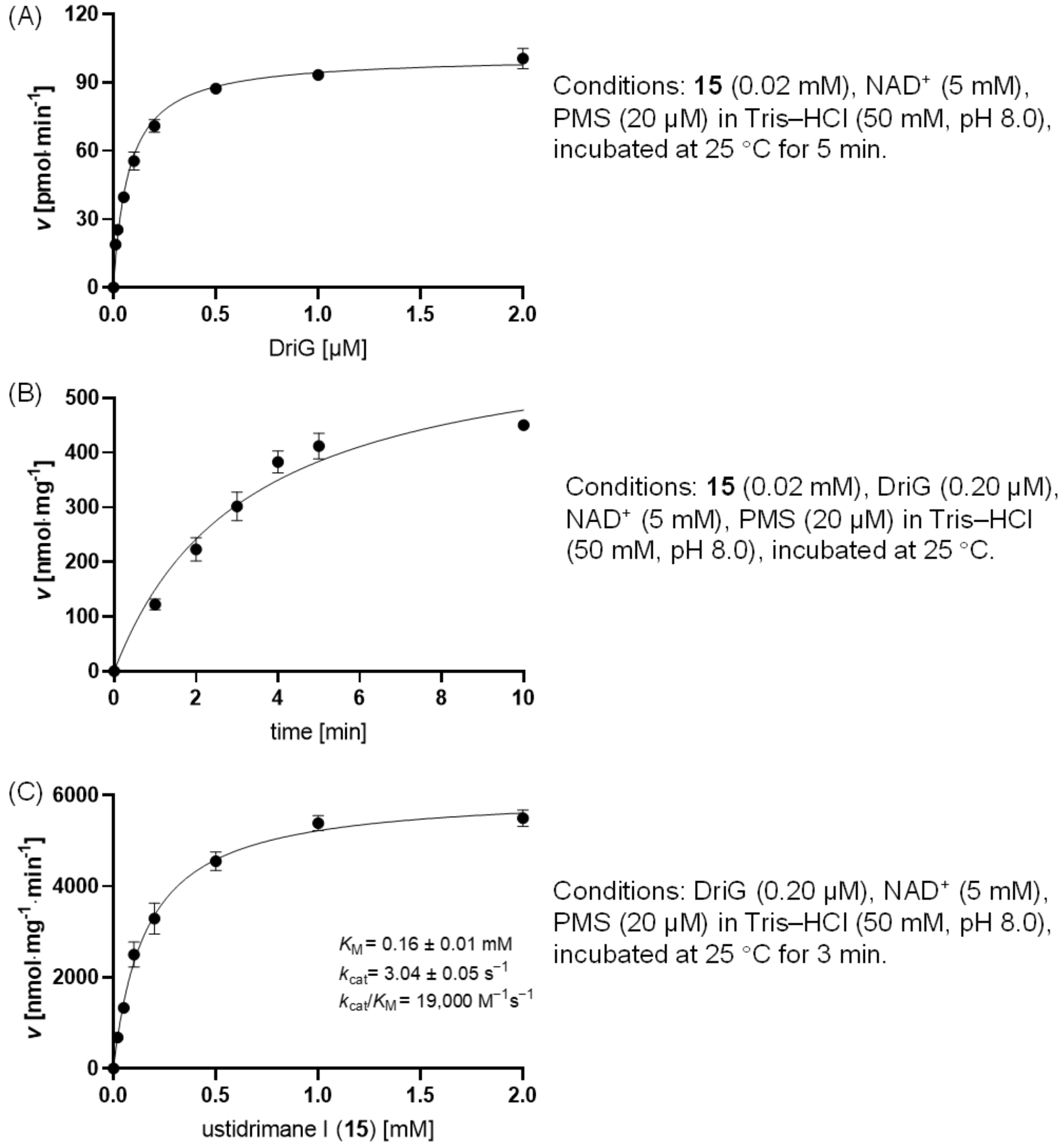

Figure S128. Determination of kinetic parameters of DriG toward ustidrimane I (**15**).

(A) Dependence of DriG activity on protein concentration (B) Dependence of DriG activity on reaction time (C) Dependence of DriG activity on substrate concentration.

References

(1) Janzen, D. J.; Zhou, J.; Li, S.-M. Biosynthesis of *p*-terphenyls in *Aspergillus ustus* implies enzymatic reductive dehydration and spontaneous dibenzofuran formation. *Org. Lett.* **2023**, *25*, 6311–6316. DOI: 10.1021/acs.orglett.3c02234.

(2) Chiang, Y.-M.; Ahuja, M.; Oakley, C. E.; Entwistle, R.; Asokan, A.; Zutz, C.; Wang, C. C. C.; Oakley, B. R. Development of genetic dereplication strains in *Aspergillus nidulans* results in the discovery of aspercryptin. *Angew. Chem., Int. Ed.* **2016**, *55*, 1662–1665. DOI: 10.1002/anie.201507097.

(3) Xiang, P.; Ludwig-Radtke, L.; Yin, W.-B.; Li, S.-M. Isocoumarin formation by heterologous gene expression and modification by host enzymes. *Org. Biomol. Chem.* **2020**, *18*, 4946–4948. DOI: 10.1039/d0ob00989j.

(4) Wei, H.-L.; Chen, X.-L.; Dai, Y.; Yang, L.; Li, S.-M. A unique three-enzyme cascade mediates efficient regioselective and stereospecific epoxytetrahydrofuran ring formation in deoxyverrucosidin biosynthesis. *Chem. Sci.* **2025**, *16* (34), 15564–15570. DOI: 10.1039/d5sc03423j.

(5) Zhang, P.; Wang, X.; Fan, A.; Zheng, Y.; Liu, X.; Wang, S.; Zou, H.; Oakley, B. R.; Keller, N. P.; Yin, W. B. A cryptic pigment biosynthetic pathway uncovered by heterologous expression is essential for conidial development in *Pestalotiopsis fici*. *Mol. Microbiol.* **2017**, *105*, 469–483. DOI: 10.1111/mmi.13711.

(6) Kindinger, F.; Nies, J.; Becker, A.; Zhu, T.; Li, S.-M. Genomic locus of a *Penicillium crustosum* pigment as an integration site for secondary metabolite gene expression. *ACS Chem. Biol.* **2019**, *14* (6), 1227–1234. DOI: 10.1021/acschembio.9b00164.

(7) Li, W.; Dai, Y.; Wei, H.-L.; Zhou, J.; Fan, J.; Li, Z.-H.; Xie, X.; Li, S.-M. Biosynthesis of tetramate derivatives in *Penicillium crustosum* reveals the involvement of *ortho*-quinone methide in crosstalk of multiple pathways. *Org. Lett.* **2024**, *26* (49), 10464–10469. DOI: 10.1021/acs.orglett.4c03698.

(8) Fan, J.; Liao, G.; Kindinger, F.; Ludwig-Radtke, L.; Yin, W.-B.; Li, S.-M. Peniphenone and penilactone formation in *Penicillium crustosum* via 1,4-Michael additions of *ortho*-quinone methide from hydroxyclavatol to *γ*-butyrolactones from crustosic acid. *J. Am. Chem. Soc.* **2019**, *141*, 4225–4229. DOI: 10.1021/jacs.9b00110.

(9) Stec, E.; Steffan, N.; Kremer, A.; Zou, H.; Zheng, X.; Li, S.-M. Two Lysine residues are responsible for the enzymatic activities of indole prenyltransferases from fungi. *ChemBioChem* **2008**, *9*, 2055–2058.

(10) Zhang, X.-Q.; Liu, J.; Xu, W.-L.; Men, W.-H.; He, H.-B.; Guo, Z.-Y.; Proksch, P. Spinulactones A-F: bioactive drimane-type sesquiterpenes from the endophytic *Penicillium spinulosum* 9-1. *J. Nat. Prod.* **2025**, *88*, 1491–1498. DOI: 10.1021/acs.jnatprod.5c00431.

(11) Tang, Y.-J.; Zou, Y. Cyclo-farnesyl diphosphate-dependent prenylation in fungi. *Org. Lett.*

2024, *26* (39), 8366–8370. DOI: 10.1021/acs.orglett.4c03154.
(12) Maurs, M.; Azerad, R.; Cortés, M.; Aranda, G.; Delahaye, M. B.; Ricard, L. Microbial hydroxylation of natural drimenic lactones. *Phytochemistry* **1999**, *52* (2), 291–296. DOI: 10.1016/s0031-9422(99)00199-5.
(13) Nakano, T.; Maillo, M. A. A novel, short synthesis of confertifolin. *Synth. Commun.* **1981**, *11* (6), 463–473. DOI: 10.1080/00397918108061878.
(14) Akita, H.; Anazawa, A.; Oishi, T. Syntheses of optically active (+)-fragrolide and (+)-bemadienolide from dehydroabietic acid. *Chem. Pharm. Bull.* **1981**, *29* (6), 1588–1593. DOI: 10.1248/cpb.29.1588.
(15) Canonica, L.; Corbella, A.; Gariboldi, P.; Jommi, G.; Krˇepinský, J.; Ferrari, G.; Casagrande, C. Sesquiterpenoids of *Cinnamosma fragrans* baillon. *Tetrahedron* **1969**, *25* (17), 3903–3908. DOI: 10.1016/S0040-4020(01)82922-7.
(16) Aranda, G.; Moreno, L.; Cortés, M.; Prangé, T.; Maurs, M.; Azerad, R. A new example of 1α-hydroxylation of drimanic terpenes through combined microbial and chemical processes. *Tetrahedron* **2001**, *57* (28), 6051–6056. DOI: 10.1016/S0040-4020(01)00595-6.
(17) Bond, C.; Tang, Y.; Li, L. *Saccharomyces cerevisiae* as a tool for mining, studying and engineering fungal polyketide synthases. *Fungal Genet. Biol.* **2016**, *89*, 52–61. DOI: 10.1016/j.fgb.2016.01.005.
(18) Zhou, J.; Li, S.-M. Conversion of viridicatic acid to crustosic acid by cytochrome P450 enzyme-catalysed hydroxylation and spontaneous cyclisation. *Appl. Microbiol. Biotechnol.* **2021**, *105*, 9181–9189. DOI: 10.1007/s00253-021-11674-4.
